# Supplementary material for: Detection and characterization of the SARS-CoV-2 lineage B.1.526 in New York
Source: Nat Commun. 2021 Aug 9;12:4886. doi: 10.1038/s41467-021-25168-4 (PMC8352861; doi:10.1038/s41467-021-25168-4)
Supplement: Supplementary file 8 — Supplementary Data 4 [file 41467_2021_25168_MOESM8_ESM.zip › GISAID_acknowledements_tables/gisaid_hcov-19_acknowledgement_table_2021_02_13_00-9.pdf]

We gratefully acknowledge the following Authors from the Originating laboratories responsible for obtaining the specimens, as well as the Submitting laboratories where the genome data were generated and shared via GISAID, on which this research is based.

All Submitters of data may be contacted directly via www.gisaid.org

Authors are sorted alphabetically.

Accession ID	Originating Laboratory	Submitting Laboratory	Authors
EPI_ISL_645133, EPI_ISL_645134, EPI_ISL_649108, EPI_ISL_649110	Respiratory Virus Unit, Microbiology Services Colindale, Public Health England	COVID-19 Genomics UK (COG-UK) Consortium	PHE Covid Sequencing Team
EPI_ISL_649125	Wellington SCL (WN)	Institute of Environmental Science and Research (ESR)	Xiaoyun Ren, Matt Storey, Nikki Freed, Muhammad Faisal, Jing Wang, Hermes Perez, Anja Werno, Antje van der Linden, Arlo Upton, Chris Mansell, David Hammer, Dragana Drinkovic, Gary McAuliffe, Hana Sofia Andersson, James Ussher, Jill Sherwood, Josh Freeman, Julia Howard, Juliet Elvy, Mary DeAlmeida, Matt Blakiston, Matthew Rogers, Max Bloomfield, Michael Addidle, Michelle Balm, Sally Roberts, Sarah Jefferies, Sharmini Muttaiyah, Susan Morpeth, Susan Taylor, Timothy Blackmore, Vani Sathyendran, Veronica Playle, Virginia Hope, Erasmus Smit, Lauren Jelly, Olin Silander, Joep de Ligt
EPI_ISL_649936, EPI_ISL_649937	Respiratory Virus Unit, Microbiology Services Colindale, Public Health England	COVID-19 Genomics UK (COG-UK) Consortium	PHE Covid Sequencing Team
EPI_ISL_650178, EPI_ISL_650187, EPI_ISL_650266, EPI_ISL_650276, EPI_ISL_650314, EPI_ISL_650443	Queens Medical Centre, Clinical Microbiology Department / DeepSeq Nottingham	COVID-19 Genomics UK (COG-UK) Consortium	Gemma Clark, Wendy Smith, Manjinder Khakh, Vicki M Fleming, Michelle M Lister, Hannah Howson-Wells, Jonathan Ball, Patrick McClure, Joseph Chappell, Theocharis Tsoleridis, Nadine Holmes, Matthew Carlisle, Christopher Moore, Fei Sang, Johnny Debebe, Victoria Wright, Matthew Loose
EPI_ISL_650459	Quadram Institute Bioscience	COVID-19 Genomics UK (COG-UK) Consortium	Dave J. Baker, Gemma L. Kay, Alp Aydin, Thanh Le-Viet, Steven Rudder, Ana P. Tedim, Anastasia Kolyva, Maria Diaz, Leonardo de Oliveira Martins, Nabil-Fareed Alikhan, Lizzie Meadows, Rachael Stanley, Ngozi Elumogo, Muhammed Yasir, Nicholas M. Thomson, Alexander J Trotter, Rachel Gilroy, Samuel Bloomfield, Claire Stuart, Andrew Bell, Reenesh Prakash, Samir Dervisevic, Alison E. Mather, John Wain, Mark Webber, Andrew J. Page, Justin O'Grady
EPI_ISL_650786, EPI_ISL_650830, EPI_ISL_650835, EPI_ISL_650979, EPI_ISL_650987, EPI_ISL_651065, EPI_ISL_651107, EPI_ISL_651189, EPI_ISL_651286, EPI_ISL_651401, EPI_ISL_651408, EPI_ISL_652401, EPI_ISL_652402, EPI_ISL_652403, EPI_ISL_652404, EPI_ISL_652405, EPI_ISL_652406, EPI_ISL_652407, EPI_ISL_652408, EPI_ISL_652409, EPI_ISL_652410, EPI_ISL_652411, EPI_ISL_652412, EPI_ISL_652413			
see above	Queens Medical Centre, Clinical Microbiology Department / DeepSeq Nottingham	COVID-19 Genomics UK (COG-UK) Consortium	Gemma Clark, Wendy Smith, Manjinder Khakh, Vicki M Fleming, Michelle M Lister, Hannah Howson-Wells, Jonathan Ball, Patrick McClure, Joseph Chappell, Theocharis Tsoleridis, Nadine Holmes, Matthew Carlisle, Christopher Moore, Fei Sang, Johnny Debebe, Victoria Wright, Matthew Loose
EPI_ISL_654799, EPI_ISL_654800, EPI_ISL_654801, EPI_ISL_654802, EPI_ISL_654803, EPI_ISL_654804, EPI_ISL_654805, EPI_ISL_654806, EPI_ISL_654807, EPI_ISL_654808, EPI_ISL_654809			
see above	SA Pathology	SA Pathology	Lex Leong, Julien Soubrier, Chuan Kok Lim, Song Gao, Mark Turra, Karin Kassahn, Ivan Bastian, Geoff Higgins
EPI_ISL_660228, EPI_ISL_660229, EPI_ISL_660230, EPI_ISL_660231, EPI_ISL_660232, EPI_ISL_660256, EPI_ISL_660257, EPI_ISL_660258	KRISP, KZN Research Innovation and Sequencing Platform	KRISP, KZN Research Innovation and Sequencing Platform	Giandhari J, Pillay S, Lessells R, Mdlalose K, York D, Khan S, Tegally H, Wilkinson E, de Oliveira T
EPI_ISL_661173	Gundersen Molecular Diagnostics Laboratory	Kabara Cancer Research Institute	Craig S. Richmond, Paraic A. Kenny
EPI_ISL_661201, EPI_ISL_661204, EPI_ISL_661205, EPI_ISL_661206, EPI_ISL_661207, EPI_ISL_661208, EPI_ISL_661209, EPI_ISL_661210, EPI_ISL_661211, EPI_ISL_661212, EPI_ISL_661213, EPI_ISL_661214, EPI_ISL_661218, EPI_ISL_661219, EPI_ISL_661220, EPI_ISL_661221, EPI_ISL_661222, EPI_ISL_661223, EPI_ISL_661224, EPI_ISL_661225, EPI_ISL_661226, EPI_ISL_661227, EPI_ISL_661228, EPI_ISL_661229, EPI_ISL_661230, EPI_ISL_661231, EPI_ISL_661232, EPI_ISL_661233, EPI_ISL_661234, EPI_ISL_661235, EPI_ISL_661236, EPI_ISL_661237, EPI_ISL_661238, EPI_ISL_661239, EPI_ISL_661240, EPI_ISL_661241, EPI_ISL_661242, EPI_ISL_661243, EPI_ISL_661244, EPI_ISL_661245			
see above	Department of Clinical Microbiology	GIGA Medical Genomics	Keith Durkin, Maria Artesi, Sébastien Bontems, Raphaël Boreux, Bouchra Boujemla, Cécile Meex, Pierrette Melin, Marie-Pierre Hayette, Vincent Bours
EPI_ISL_661257, EPI_ISL_661258, EPI_ISL_661259, EPI_ISL_661262, EPI_ISL_661263	LabPLUS	Institute of Environmental Science and Research (ESR)	Xiaoyun Ren, Matt Storey, Nikki Freed, Muhammad Faisal, Jing Wang, Hermes Perez, Anja Werno, Antje van der Linden, Arlo Upton, Chris Mansell, David Hammer, Dragana Drinkovic, Gary McAuliffe, Hana Sofia Andersson, James Ussher, Jill Sherwood, Josh Freeman, Julia Howard, Juliet Elvy, Mary DeAlmeida, Matt Blakiston, Matthew Rogers, Max Bloomfield, Michael Addidle, Michelle Balm, Sally Roberts, Sarah Jefferies, Sharmini Muttaiyah, Susan Morpeth, Susan Taylor, Timothy Blackmore, Vani Sathyendran, Veronica Playle, Virginia Hope, Erasmus Smit, Lauren Jelly, Olin Silander, Joep de Ligt
EPI_ISL_664120, EPI_ISL_664139, EPI_ISL_664199, EPI_ISL_664200, EPI_ISL_664234, EPI_ISL_664291, EPI_ISL_664304, EPI_ISL_664316, EPI_ISL_664317, EPI_ISL_664380, EPI_ISL_664382, EPI_ISL_664383, EPI_ISL_664384, EPI_ISL_664446, EPI_ISL_664455, EPI_ISL_664470, EPI_ISL_664516, EPI_ISL_664527, EPI_ISL_664529, EPI_ISL_664535, EPI_ISL_664536, EPI_ISL_664537, EPI_ISL_664538, EPI_ISL_664588, EPI_ISL_664589, EPI_ISL_664601, EPI_ISL_664616, EPI_ISL_664635, EPI_ISL_664656, EPI_ISL_664731, EPI_ISL_664749, EPI_ISL_664995, EPI_ISL_665005, EPI_ISL_665006, EPI_ISL_665007, EPI_ISL_665008, EPI_ISL_665009, EPI_ISL_665010, EPI_ISL_665011, EPI_ISL_665012, EPI_ISL_665020, EPI_ISL_665021, EPI_ISL_665022			
see above	Department of Pathology, University of Cambridge	COVID-19 Genomics UK (COG-UK) Consortium	Aminu S. Jahun, Yasmin Chaudhry, Grant Hall, Iliana Georgana, Myra Hosmillo, Martin D. Curran, Malte Pinckert, Surendra Parmar, Ian Goodfellow
EPI_ISL_665268	Quadram Institute Bioscience	COVID-19 Genomics UK (COG-UK) Consortium	Dave J. Baker, Gemma L. Kay, Alp Aydin, Thanh Le-Viet, Steven Rudder, Ana P. Tedim, Anastasia Kolyva, Maria Diaz, Leonardo de Oliveira Martins, Nabil-Fareed Alikhan, Lizzie Meadows, Rachael Stanley, Ngozi Elumogo, Muhammed Yasir, Nicholas M. Thomson, Alexander J Trotter, Rachel Gilroy, Samuel Bloomfield, Claire Stuart, Andrew Bell, Reenesh Prakash, Samir Dervisevic, Alison E. Mather, John Wain, Mark Webber, Andrew J. Page, Justin O'Grady
EPI_ISL_665291	Queens Medical Centre, Clinical Microbiology Department / DeepSeq Nottingham	COVID-19 Genomics UK (COG-UK) Consortium	Gemma Clark, Wendy Smith, Manjinder Khakh, Vicki M Fleming, Michelle M Lister, Hannah Howson-Wells, Jonathan Ball, Patrick McClure, Joseph Chappell, Theocharis Tsoleridis, Nadine Holmes, Matthew Carlisle, Christopher Moore, Fei Sang, Johnny Debebe, Victoria Wright, Matthew Loose
EPI_ISL_665316, EPI_ISL_665336	Quadram Institute Bioscience	COVID-19 Genomics UK (COG-UK) Consortium	Dave J. Baker, Gemma L. Kay, Alp Aydin, Thanh Le-Viet, Steven Rudder, Ana P. Tedim, Anastasia Kolyva, Maria Diaz, Leonardo de Oliveira Martins, Nabil-Fareed Alikhan, Lizzie Meadows, Rachael Stanley, Ngozi Elumogo, Muhammed Yasir, Nicholas M. Thomson, Alexander J Trotter, Rachel Gilroy, Samuel Bloomfield, Claire Stuart, Andrew Bell, Reenesh Prakash, Samir Dervisevic, Alison E. Mather, John Wain, Mark Webber, Andrew J. Page, Justin O'Grady
EPI_ISL_665345	Queens Medical Centre, Clinical Microbiology Department / DeepSeq Nottingham	COVID-19 Genomics UK (COG-UK) Consortium	Gemma Clark, Wendy Smith, Manjinder Khakh, Vicki M Fleming, Michelle M Lister, Hannah Howson-Wells, Jonathan Ball, Patrick McClure, Joseph Chappell, Theocharis Tsoleridis, Nadine Holmes, Matthew Carlisle, Christopher Moore, Fei Sang, Johnny Debebe, Victoria Wright, Matthew Loose
EPI_ISL_665362, EPI_ISL_665369, EPI_ISL_665370	Quadram Institute Bioscience	COVID-19 Genomics UK (COG-UK) Consortium	Dave J. Baker, Gemma L. Kay, Alp Aydin, Thanh Le-Viet, Steven Rudder, Ana P. Tedim, Anastasia Kolyva, Maria Diaz, Leonardo de Oliveira Martins, Nabil-Fareed Alikhan, Lizzie Meadows, Rachael Stanley, Ngozi Elumogo, Muhammed Yasir, Nicholas M. Thomson, Alexander J Trotter, Rachel Gilroy, Samuel Bloomfield, Claire Stuart, Andrew Bell, Reenesh Prakash, Samir Dervisevic, Alison E. Mather, John Wain, Mark Webber, Andrew J. Page, Justin O'Grady
EPI_ISL_665420, EPI_ISL_665421, EPI_ISL_665424, EPI_ISL_665426	Queens Medical Centre, Clinical Microbiology Department / DeepSeq Nottingham	COVID-19 Genomics UK (COG-UK) Consortium	Gemma Clark, Wendy Smith, Manjinder Khakh, Vicki M Fleming, Michelle M Lister, Hannah Howson-Wells, Jonathan Ball, Patrick McClure, Joseph Chappell, Theocharis Tsoleridis, Nadine Holmes, Matthew Carlisle, Christopher Moore, Fei Sang, Johnny Debebe, Victoria Wright, Matthew Loose
EPI_ISL_665427, EPI_ISL_665429	Quadram Institute Bioscience	COVID-19 Genomics UK (COG-UK) Consortium	Dave J. Baker, Gemma L. Kay, Alp Aydin, Thanh Le-Viet, Steven Rudder, Ana P. Tedim, Anastasia Kolyva, Maria Diaz, Leonardo de Oliveira Martins, Nabil-Fareed Alikhan, Lizzie Meadows, Rachael Stanley, Ngozi Elumogo, Muhammed Yasir, Nicholas M. Thomson, Alexander J Trotter, Rachel Gilroy, Samuel Bloomfield, Claire Stuart, Andrew Bell, Reenesh Prakash, Samir Dervisevic, Alison E. Mather, John Wain, Mark Webber, Andrew J. Page, Justin O'Grady
EPI_ISL_665430	Queens Medical Centre, Clinical Microbiology Department / DeepSeq Nottingham	COVID-19 Genomics UK (COG-UK) Consortium	Gemma Clark, Wendy Smith, Manjinder Khakh, Vicki M Fleming, Michelle M Lister, Hannah Howson-Wells, Jonathan Ball, Patrick McClure, Joseph Chappell, Theocharis Tsoleridis, Nadine Holmes, Matthew Carlisle, Christopher Moore, Fei Sang, Johnny Debebe, Victoria Wright, Matthew Loose
EPI_ISL_665436	West of Scotland Specialist Virology Centre, NHSGGC /	COVID-19 Genomics UK (COG-UK) Consortium	Ana da Silva Filipe, Natasha Johnson, Kathy Smollett, Daniel Mair, Stephen Carmichael, Alice Broos, Lily Tong, Jenna Nichols, Kyriaki Nomikou; Sarah

	MRC-University of Glasgow Centre for Virus Research		McDonald; Richard Orton, Joseph Hughes, Sreenu Vattipally, David L Robertson; Alasdair MacLean, Rory Gunson; Sharif Shaaban, Matthew Holden; Rachel Blacow, Guy Mollett, Kathy Li, James Shepherd, Antonia Ho, Emma Thomson
EPI_ISL_665438	Quadram Institute Bioscience	COVID-19 Genomics UK (COG-UK) Consortium	Dave J. Baker, Gemma L. Kay, Alp Aydin, Thanh Le-Viet, Steven Rudder, Ana P. Tedim, Anastasia Kolyva, Maria Diaz, Leonardo de Oliveira Martins, Nabil-Fareed Alikhan, Lizzie Meadows, Rachael Stanley, Ngozi Elumogo, Muhammed Yasir, Nicholas M. Thomson, Alexander J Trotter, Rachel Gilroy, Samuel Bloomfield, Claire Stuart, Andrew Bell, Reenesh Prakash, Samir Dervisevic, Alison E. Mather, John Wain, Mark Webber, Andrew J. Page, Justin O'Grady
EPI_ISL_665443, EPI_ISL_665452	Queens Medical Centre, Clinical Microbiology Department / DeepSeq Nottingham	COVID-19 Genomics UK (COG-UK) Consortium	Gemma Clark, Wendy Smith, Manjinder Khakh, Vicki M Fleming, Michelle M Lister, Hannah Howson-Wells, Jonathan Ball, Patrick McClure, Joseph Chappell, Theocharis Tsoleridis, Nadine Holmes, Matthew Carlisle, Christopher Moore, Fei Sang, Johnny Debebe, Victoria Wright, Matthew Loose
EPI_ISL_665453, EPI_ISL_665463, EPI_ISL_665465, EPI_ISL_665475, EPI_ISL_665519	Quadram Institute Bioscience	COVID-19 Genomics UK (COG-UK) Consortium	Dave J. Baker, Gemma L. Kay, Alp Aydin, Thanh Le-Viet, Steven Rudder, Ana P. Tedim, Anastasia Kolyva, Maria Diaz, Leonardo de Oliveira Martins, Nabil-Fareed Alikhan, Lizzie Meadows, Rachael Stanley, Ngozi Elumogo, Muhammed Yasir, Nicholas M. Thomson, Alexander J Trotter, Rachel Gilroy, Samuel Bloomfield, Claire Stuart, Andrew Bell, Reenesh Prakash, Samir Dervisevic, Alison E. Mather, John Wain, Mark Webber, Andrew J. Page, Justin O'Grady
EPI_ISL_665521	West of Scotland Specialist Virology Centre, NHSGGC / MRC-University of Glasgow Centre for Virus Research	COVID-19 Genomics UK (COG-UK) Consortium	Ana da Silva Filipe, Natasha Johnson, Kathy Smollett, Daniel Mair, Stephen Carmichael, Alice Broos, Lily Tong, Jenna Nichols, Kyriaki Nomikou; Sarah McDonald; Richard Orton, Joseph Hughes, Sreenu Vattipally, David L Robertson; Alasdair MacLean, Rory Gunson; Sharif Shaaban, Matthew Holden; Rachel Blacow, Guy Mollett, Kathy Li, James Shepherd, Antonia Ho, Emma Thomson
EPI_ISL_665534	Quadram Institute Bioscience	COVID-19 Genomics UK (COG-UK) Consortium	Dave J. Baker, Gemma L. Kay, Alp Aydin, Thanh Le-Viet, Steven Rudder, Ana P. Tedim, Anastasia Kolyva, Maria Diaz, Leonardo de Oliveira Martins, Nabil-Fareed Alikhan, Lizzie Meadows, Rachael Stanley, Ngozi Elumogo, Muhammed Yasir, Nicholas M. Thomson, Alexander J Trotter, Rachel Gilroy, Samuel Bloomfield, Claire Stuart, Andrew Bell, Reenesh Prakash, Samir Dervisevic, Alison E. Mather, John Wain, Mark Webber, Andrew J. Page, Justin O'Grady
EPI_ISL_665538	West of Scotland Specialist Virology Centre, NHSGGC / MRC-University of Glasgow Centre for Virus Research	COVID-19 Genomics UK (COG-UK) Consortium	Ana da Silva Filipe, Natasha Johnson, Kathy Smollett, Daniel Mair, Stephen Carmichael, Alice Broos, Lily Tong, Jenna Nichols, Kyriaki Nomikou; Sarah McDonald; Richard Orton, Joseph Hughes, Sreenu Vattipally, David L Robertson; Alasdair MacLean, Rory Gunson; Sharif Shaaban, Matthew Holden; Rachel Blacow, Guy Mollett, Kathy Li, James Shepherd, Antonia Ho, Emma Thomson
EPI_ISL_665543, EPI_ISL_665544, EPI_ISL_665550, EPI_ISL_665551	Queens Medical Centre, Clinical Microbiology Department / DeepSeq Nottingham	COVID-19 Genomics UK (COG-UK) Consortium	Gemma Clark, Wendy Smith, Manjinder Khakh, Vicki M Fleming, Michelle M Lister, Hannah Howson-Wells, Jonathan Ball, Patrick McClure, Joseph Chappell, Theocharis Tsoleridis, Nadine Holmes, Matthew Carlisle, Christopher Moore, Fei Sang, Johnny Debebe, Victoria Wright, Matthew Loose
EPI_ISL_665560	Quadram Institute Bioscience	COVID-19 Genomics UK (COG-UK) Consortium	Dave J. Baker, Gemma L. Kay, Alp Aydin, Thanh Le-Viet, Steven Rudder, Ana P. Tedim, Anastasia Kolyva, Maria Diaz, Leonardo de Oliveira Martins, Nabil-Fareed Alikhan, Lizzie Meadows, Rachael Stanley, Ngozi Elumogo, Muhammed Yasir, Nicholas M. Thomson, Alexander J Trotter, Rachel Gilroy, Samuel Bloomfield, Claire Stuart, Andrew Bell, Reenesh Prakash, Samir Dervisevic, Alison E. Mather, John Wain, Mark Webber, Andrew J. Page, Justin O'Grady
EPI_ISL_665561, EPI_ISL_665562	Queens Medical Centre, Clinical Microbiology Department / DeepSeq Nottingham	COVID-19 Genomics UK (COG-UK) Consortium	Gemma Clark, Wendy Smith, Manjinder Khakh, Vicki M Fleming, Michelle M Lister, Hannah Howson-Wells, Jonathan Ball, Patrick McClure, Joseph Chappell, Theocharis Tsoleridis, Nadine Holmes, Matthew Carlisle, Christopher Moore, Fei Sang, Johnny Debebe, Victoria Wright, Matthew Loose
EPI_ISL_665599	Quadram Institute Bioscience	COVID-19 Genomics UK (COG-UK) Consortium	Dave J. Baker, Gemma L. Kay, Alp Aydin, Thanh Le-Viet, Steven Rudder, Ana P. Tedim, Anastasia Kolyva, Maria Diaz, Leonardo de Oliveira Martins, Nabil-Fareed Alikhan, Lizzie Meadows, Rachael Stanley, Ngozi Elumogo, Muhammed Yasir, Nicholas M. Thomson, Alexander J Trotter, Rachel Gilroy, Samuel Bloomfield, Claire Stuart, Andrew Bell, Reenesh Prakash, Samir Dervisevic, Alison E. Mather, John Wain, Mark Webber, Andrew J. Page, Justin O'Grady
EPI_ISL_665601	Queens Medical Centre, Clinical Microbiology Department / DeepSeq Nottingham	COVID-19 Genomics UK (COG-UK) Consortium	Gemma Clark, Wendy Smith, Manjinder Khakh, Vicki M Fleming, Michelle M Lister, Hannah Howson-Wells, Jonathan Ball, Patrick McClure, Joseph Chappell, Theocharis Tsoleridis, Nadine Holmes, Matthew Carlisle, Christopher Moore, Fei Sang, Johnny Debebe, Victoria Wright, Matthew Loose
EPI_ISL_665609	West of Scotland Specialist Virology Centre, NHSGGC / MRC-University of Glasgow Centre for Virus Research	COVID-19 Genomics UK (COG-UK) Consortium	Ana da Silva Filipe, Natasha Johnson, Kathy Smollett, Daniel Mair, Stephen Carmichael, Alice Broos, Lily Tong, Jenna Nichols, Kyriaki Nomikou; Sarah McDonald; Richard Orton, Joseph Hughes, Sreenu Vattipally, David L Robertson; Alasdair MacLean, Rory Gunson; Sharif Shaaban, Matthew Holden; Rachel Blacow, Guy Mollett, Kathy Li, James Shepherd, Antonia Ho, Emma Thomson
EPI_ISL_665612	Queens Medical Centre, Clinical Microbiology Department / DeepSeq Nottingham	COVID-19 Genomics UK (COG-UK) Consortium	Gemma Clark, Wendy Smith, Manjinder Khakh, Vicki M Fleming, Michelle M Lister, Hannah Howson-Wells, Jonathan Ball, Patrick McClure, Joseph Chappell, Theocharis Tsoleridis, Nadine Holmes, Matthew Carlisle, Christopher Moore, Fei Sang, Johnny Debebe, Victoria Wright, Matthew Loose
EPI_ISL_665616	Centre for Enzyme Innovation, University of Portsmouth / Translational Research Laboratory, Portsmouth Hospitals NHS Trust	COVID-19 Genomics UK (COG-UK) Consortium	Angela Beckett,Yann Bourgeois,Garry Scarlett,Sharon Glaysher,Scott Elliott,Kelly Bicknell,Robert Impey,Allyson Lloyd,Sarah Wyllie,Ethan Butcher,Anoop Chauhan,Samuel Robson
EPI_ISL_665622	Queens Medical Centre, Clinical Microbiology Department / DeepSeq Nottingham	COVID-19 Genomics UK (COG-UK) Consortium	Gemma Clark, Wendy Smith, Manjinder Khakh, Vicki M Fleming, Michelle M Lister, Hannah Howson-Wells, Jonathan Ball, Patrick McClure, Joseph Chappell, Theocharis Tsoleridis, Nadine Holmes, Matthew Carlisle, Christopher Moore, Fei Sang, Johnny Debebe, Victoria Wright, Matthew Loose
EPI_ISL_665625	Centre for Enzyme Innovation, University of Portsmouth / Translational Research Laboratory, Portsmouth Hospitals NHS Trust	COVID-19 Genomics UK (COG-UK) Consortium	Angela Beckett,Yann Bourgeois,Garry Scarlett,Sharon Glaysher,Scott Elliott,Kelly Bicknell,Robert Impey,Allyson Lloyd,Sarah Wyllie,Ethan Butcher,Anoop Chauhan,Samuel Robson
EPI_ISL_665636	University College London Hospital	COVID-19 Genomics UK (COG-UK) Consortium	Judith Heaney, Matthew Byott, Catherine Houlihan, Dan Frampton, Stuart Kirk, Moira Spyer and Eleni Nastouli
EPI_ISL_665638	Quadram Institute Bioscience	COVID-19 Genomics UK (COG-UK) Consortium	Dave J. Baker, Gemma L. Kay, Alp Aydin, Thanh Le-Viet, Steven Rudder, Ana P. Tedim, Anastasia Kolyva, Maria Diaz, Leonardo de Oliveira Martins, Nabil-Fareed Alikhan, Lizzie Meadows, Rachael Stanley, Ngozi Elumogo, Muhammed Yasir, Nicholas M. Thomson, Alexander J Trotter, Rachel Gilroy, Samuel Bloomfield, Claire Stuart, Andrew Bell, Reenesh Prakash, Samir Dervisevic, Alison E. Mather, John Wain, Mark Webber, Andrew J. Page, Justin O'Grady
EPI_ISL_665641	West of Scotland Specialist Virology Centre, NHSGGC / MRC-University of Glasgow Centre for Virus Research	COVID-19 Genomics UK (COG-UK) Consortium	Ana da Silva Filipe, Natasha Johnson, Kathy Smollett, Daniel Mair, Stephen Carmichael, Alice Broos, Lily Tong, Jenna Nichols, Kyriaki Nomikou; Sarah McDonald; Richard Orton, Joseph Hughes, Sreenu Vattipally, David L Robertson; Alasdair MacLean, Rory Gunson; Sharif Shaaban, Matthew Holden; Rachel Blacow, Guy Mollett, Kathy Li, James Shepherd, Antonia Ho, Emma Thomson
EPI_ISL_665643	University College London Hospital	COVID-19 Genomics UK (COG-UK) Consortium	Judith Heaney, Matthew Byott, Catherine Houlihan, Dan Frampton, Stuart Kirk, Moira Spyer and Eleni Nastouli
EPI_ISL_665668, EPI_ISL_665678, EPI_ISL_665692, EPI_ISL_665693, EPI_ISL_665697, EPI_ISL_665714, EPI_ISL_665730	Quadram Institute Bioscience	COVID-19 Genomics UK (COG-UK) Consortium	Dave J. Baker, Gemma L. Kay, Alp Aydin, Thanh Le-Viet, Steven Rudder, Ana P. Tedim, Anastasia Kolyva, Maria Diaz, Leonardo de Oliveira Martins, Nabil-Fareed Alikhan, Lizzie Meadows, Rachael Stanley, Ngozi Elumogo, Muhammed Yasir, Nicholas M. Thomson, Alexander J Trotter, Rachel Gilroy, Samuel Bloomfield, Claire Stuart, Andrew Bell, Reenesh Prakash, Samir Dervisevic, Alison E. Mather, John Wain, Mark Webber, Andrew J. Page, Justin O'Grady
EPI_ISL_665740	Queens Medical Centre, Clinical Microbiology Department / DeepSeq Nottingham	COVID-19 Genomics UK (COG-UK) Consortium	Gemma Clark, Wendy Smith, Manjinder Khakh, Vicki M Fleming, Michelle M Lister, Hannah Howson-Wells, Jonathan Ball, Patrick McClure, Joseph Chappell, Theocharis Tsoleridis, Nadine Holmes, Matthew Carlisle, Christopher Moore, Fei Sang, Johnny Debebe, Victoria Wright, Matthew Loose
EPI_ISL_665746	Quadram Institute Bioscience	COVID-19 Genomics UK (COG-UK) Consortium	Dave J. Baker, Gemma L. Kay, Alp Aydin, Thanh Le-Viet, Steven Rudder, Ana P. Tedim, Anastasia Kolyva, Maria Diaz, Leonardo de Oliveira Martins, Nabil-Fareed Alikhan, Lizzie Meadows, Rachael Stanley, Ngozi Elumogo, Muhammed Yasir, Nicholas M. Thomson, Alexander J Trotter, Rachel Gilroy, Samuel Bloomfield, Claire Stuart, Andrew Bell, Reenesh Prakash, Samir Dervisevic, Alison E. Mather, John Wain, Mark Webber, Andrew J. Page, Justin O'Grady
EPI_ISL_665789	Queens Medical Centre, Clinical Microbiology Department / DeepSeq Nottingham	COVID-19 Genomics UK (COG-UK) Consortium	Gemma Clark, Wendy Smith, Manjinder Khakh, Vicki M Fleming, Michelle M Lister, Hannah Howson-Wells, Jonathan Ball, Patrick McClure, Joseph Chappell, Theocharis Tsoleridis, Nadine Holmes, Matthew Carlisle, Christopher Moore, Fei Sang, Johnny Debebe, Victoria Wright, Matthew Loose
EPI_ISL_665825, EPI_ISL_665839, EPI_ISL_665840, EPI_ISL_665841, EPI_ISL_665847, EPI_ISL_665848	University College London Hospital	COVID-19 Genomics UK (COG-UK) Consortium	Judith Heaney, Matthew Byott, Catherine Houlihan, Dan Frampton, Stuart Kirk, Moira Spyer and Eleni Nastouli
EPI_ISL_665876	Quadram Institute Bioscience	COVID-19 Genomics UK (COG-UK) Consortium	Dave J. Baker, Gemma L. Kay, Alp Aydin, Thanh Le-Viet, Steven Rudder, Ana P. Tedim, Anastasia Kolyva, Maria Diaz, Leonardo de Oliveira Martins,

			Nabil-Fareed Alikhan, Lizzie Meadows, Rachael Stanley, Ngozi Elumogo, Muhammed Yasir, Nicholas M. Thomson, Alexander J Trotter, Rachel Gilroy, Samuel Bloomfield, Claire Stuart, Andrew Bell, Reenesh Prakash, Samir Dervisevic, Alison E. Mather, John Wain, Mark Webber, Andrew J. Page, Justin O'Grady
EPI_ISL_665901, EPI_ISL_665902, EPI_ISL_665903, EPI_ISL_665904, EPI_ISL_665905, EPI_ISL_665906, EPI_ISL_665907, EPI_ISL_665908, EPI_ISL_665942	Queens Medical Centre, Clinical Microbiology Department / DeepSeq Nottingham	COVID-19 Genomics UK (COG-UK) Consortium	Gemma Clark, Wendy Smith, Manjinder Khakh, Vicki M Fleming, Michelle M Lister, Hannah Howson-Wells, Jonathan Ball, Patrick McClure, Joseph Chappell, Theocharis Tsoleridis, Nadine Holmes, Matthew Carlisle, Christopher Moore, Fei Sang, Johnny Debebe, Victoria Wright, Matthew Loose
EPI_ISL_666041, EPI_ISL_666042, EPI_ISL_666043, EPI_ISL_666044, EPI_ISL_666045, EPI_ISL_666046, EPI_ISL_666047, EPI_ISL_666048, EPI_ISL_666049, EPI_ISL_666050, EPI_ISL_666051, EPI_ISL_666052, EPI_ISL_666053, EPI_ISL_666054, EPI_ISL_666055, EPI_ISL_666056, EPI_ISL_666057, EPI_ISL_666058, EPI_ISL_666059			
see above	West of Scotland Specialist Virology Centre, NHSGGC / MRC-University of Glasgow Centre for Virus Research	COVID-19 Genomics UK (COG-UK) Consortium	Ana da Silva Filipe, Natasha Johnson, Kathy Smollett, Daniel Mair, Stephen Carmichael, Alice Broos, Lily Tong, Jenna Nichols, Kyriaki Nomikou; Sarah McDonald; Richard Orton, Joseph Hughes, Sreenu Vattipally, David L Robertson; Alasdair MacLean, Rory Gunson; Sharif Shaaban, Matthew Holden; Rachel Blacow, Guy Mollett, Kathy Li, James Shepherd, Antonia Ho, Emma Thomson
EPI_ISL_667549	OHSU Lab Services Molecular Microbiology Lab	Oregon SARS-CoV-2 Genome Sequencing Center	Brendan L. O'Connell, Ruth V. Nichols, Sally Grindstaff, Alec J. Hirsch, Donna Hansel, Guang Fan, Daniel N. Streblow, William B. Messer, Andrew C. Adey, Benjamin N. Bimber, Brian J. O'Roak
EPI_ISL_668465, EPI_ISL_668467, EPI_ISL_668468, EPI_ISL_668501, EPI_ISL_668502, EPI_ISL_668510, EPI_ISL_668517, EPI_ISL_668518, EPI_ISL_668519, EPI_ISL_668521, EPI_ISL_668522, EPI_ISL_668523, EPI_ISL_668524, EPI_ISL_668525, EPI_ISL_668526, EPI_ISL_668527, EPI_ISL_668530, EPI_ISL_668534, EPI_ISL_668535, EPI_ISL_668536, EPI_ISL_668537, EPI_ISL_668544, EPI_ISL_668546, EPI_ISL_668558, EPI_ISL_668559, EPI_ISL_668561, EPI_ISL_668562, EPI_ISL_668563, EPI_ISL_668572, EPI_ISL_668579, EPI_ISL_668580, EPI_ISL_668581, EPI_ISL_668592, EPI_ISL_668595, EPI_ISL_668596, EPI_ISL_668597, EPI_ISL_668601, EPI_ISL_668606, EPI_ISL_668609, EPI_ISL_668610, EPI_ISL_668612, EPI_ISL_668615, EPI_ISL_668633, EPI_ISL_668635, EPI_ISL_668636, EPI_ISL_668643, EPI_ISL_668652, EPI_ISL_668653, EPI_ISL_668663, EPI_ISL_668664, EPI_ISL_668665, EPI_ISL_668666, EPI_ISL_668667, EPI_ISL_668668, EPI_ISL_668669, EPI_ISL_668670, EPI_ISL_668671, EPI_ISL_668672, EPI_ISL_668673, EPI_ISL_668674, EPI_ISL_668718, EPI_ISL_668719, EPI_ISL_668727, EPI_ISL_668728, EPI_ISL_668730, EPI_ISL_668735, EPI_ISL_668738, EPI_ISL_668743, EPI_ISL_668744, EPI_ISL_668757, EPI_ISL_668758, EPI_ISL_668759, EPI_ISL_668760, EPI_ISL_668761, EPI_ISL_668762, EPI_ISL_668763, EPI_ISL_668764, EPI_ISL_668765, EPI_ISL_668766, EPI_ISL_668767, EPI_ISL_668768, EPI_ISL_668769, EPI_ISL_668770, EPI_ISL_668771, EPI_ISL_668772, EPI_ISL_668773, EPI_ISL_668774, EPI_ISL_668829, EPI_ISL_668830, EPI_ISL_668839, EPI_ISL_668840, EPI_ISL_668850, EPI_ISL_668853, EPI_ISL_668854, EPI_ISL_668855, EPI_ISL_668856, EPI_ISL_668857, EPI_ISL_668858, EPI_ISL_668859, EPI_ISL_668860, EPI_ISL_668861, EPI_ISL_668862, EPI_ISL_668863, EPI_ISL_668864, EPI_ISL_668865, EPI_ISL_668866, EPI_ISL_668867, EPI_ISL_668868, EPI_ISL_668869, EPI_ISL_668870, EPI_ISL_668871, EPI_ISL_668872, EPI_ISL_668873, EPI_ISL_668874, EPI_ISL_668875, EPI_ISL_668876, EPI_ISL_668877, EPI_ISL_668878, EPI_ISL_668879, EPI_ISL_668880, EPI_ISL_668881, EPI_ISL_668882, EPI_ISL_668883, EPI_ISL_668884, EPI_ISL_668885, EPI_ISL_668886, EPI_ISL_668887, EPI_ISL_668888, EPI_ISL_668889, EPI_ISL_668890, EPI_ISL_668891, EPI_ISL_668892, EPI_ISL_668893, EPI_ISL_668894, EPI_ISL_668895, EPI_ISL_668896, EPI_ISL_668897, EPI_ISL_668898, EPI_ISL_668899, EPI_ISL_668900, EPI_ISL_668901, EPI_ISL_668902, EPI_ISL_668903, EPI_ISL_668904, EPI_ISL_668905, EPI_ISL_668906, EPI_ISL_668907, EPI_ISL_668908, EPI_ISL_668909, EPI_ISL_668910, EPI_ISL_668911, EPI_ISL_668912, EPI_ISL_668913, EPI_ISL_668914, EPI_ISL_668915, EPI_ISL_668916, EPI_ISL_668917, EPI_ISL_668918, EPI_ISL_668919, EPI_ISL_668920, EPI_ISL_668921, EPI_ISL_668922, EPI_ISL_668923, EPI_ISL_668924, EPI_ISL_668925, EPI_ISL_668926, EPI_ISL_668927, EPI_ISL_668928, EPI_ISL_668929, EPI_ISL_668930, EPI_ISL_668931, EPI_ISL_668932, EPI_ISL_668933, EPI_ISL_668934, EPI_ISL_668935, EPI_ISL_668936, EPI_ISL_668937, EPI_ISL_668938, EPI_ISL_668939, EPI_ISL_668940, EPI_ISL_668941, EPI_ISL_668942, EPI_ISL_668943, EPI_ISL_668944, EPI_ISL_668945, EPI_ISL_668946, EPI_ISL_668947, EPI_ISL_668948, EPI_ISL_668949, EPI_ISL_668950, EPI_ISL_668951, EPI_ISL_668952, EPI_ISL_668953, EPI_ISL_668954, EPI_ISL_668955, EPI_ISL_668956			
see above	Department of Virus and Microbiological Special Diagnostics, Statens Serum Institut, Copenhagen, Denmark	Albertsen Lab, Department of Chemistry and Bioscience, Aalborg University, Denmark	Danish Covid-19 Genome Consortium
EPI_ISL_671883	National Virus Reference Laboratory	National Virus Reference Laboratory	Michael Carr, Gabriel Gonzalez, Jonathan Dean, Daniel Hare, Cillian F De Gascun
EPI_ISL_672655, EPI_ISL_672656, EPI_ISL_672657, EPI_ISL_672658, EPI_ISL_672659	South Eastern Area Laboratory Services (SEALS)	NSW Health Pathology - Institute of Clinical Pathology and Medical Research; Westmead Hospital; University of Sydney	CIDM-PH et al.
EPI_ISL_672752, EPI_ISL_672753, EPI_ISL_672754, EPI_ISL_672755, EPI_ISL_672756, EPI_ISL_672757, EPI_ISL_672758, EPI_ISL_672759, EPI_ISL_672760, EPI_ISL_672761, EPI_ISL_672762, EPI_ISL_672763, EPI_ISL_672764, EPI_ISL_672765, EPI_ISL_672766, EPI_ISL_672767, EPI_ISL_672768, EPI_ISL_672769, EPI_ISL_672770, EPI_ISL_672771, EPI_ISL_672772, EPI_ISL_672773, EPI_ISL_672774, EPI_ISL_672775, EPI_ISL_672776, EPI_ISL_672777, EPI_ISL_672778, EPI_ISL_672779, EPI_ISL_672780, EPI_ISL_672781, EPI_ISL_672782, EPI_ISL_672783, EPI_ISL_672784, EPI_ISL_672785, EPI_ISL_672786, EPI_ISL_672787, EPI_ISL_672788, EPI_ISL_672789, EPI_ISL_672790, EPI_ISL_672791, EPI_ISL_672792, EPI_ISL_672793, EPI_ISL_672794, EPI_ISL_672795, EPI_ISL_672796, EPI_ISL_672797, EPI_ISL_672798, EPI_ISL_672799, EPI_ISL_672800, EPI_ISL_672801, EPI_ISL_672802, EPI_ISL_672803, EPI_ISL_672804, EPI_ISL_672805, EPI_ISL_672806, EPI_ISL_672807, EPI_ISL_672808, EPI_ISL_672809, EPI_ISL_672810, EPI_ISL_672811, EPI_ISL_672812, EPI_ISL_672813, EPI_ISL_672814, EPI_ISL_672815, EPI_ISL_672816, EPI_ISL_672817, EPI_ISL_672818, EPI_ISL_672819, EPI_ISL_672820, EPI_ISL_672821, EPI_ISL_672822, EPI_ISL_672823, EPI_ISL_672824, EPI_ISL_672825, EPI_ISL_672826, EPI_ISL_672827, EPI_ISL_672828, EPI_ISL_672829, EPI_ISL_672830, EPI_ISL_672831, EPI_ISL_672832, EPI_ISL_672833, EPI_ISL_672834, EPI_ISL_672835, EPI_ISL_672836, EPI_ISL_672837, EPI_ISL_672838, EPI_ISL_672839, EPI_ISL_672840, EPI_ISL_672841, EPI_ISL_672842, EPI_ISL_672843, EPI_ISL_672844, EPI_ISL_672845, EPI_ISL_672846, EPI_ISL_672847, EPI_ISL_672848, EPI_ISL_672849, EPI_ISL_672850, EPI_ISL_672851, EPI_ISL_672852, EPI_ISL_672853, EPI_ISL_672854, EPI_ISL_672855, EPI_ISL_672856, EPI_ISL_672857, EPI_ISL_672858, EPI_ISL_672859, EPI_ISL_672860, EPI_ISL_672861, EPI_ISL_672862, EPI_ISL_672863, EPI_ISL_672864, EPI_ISL_672865, EPI_ISL_672866, EPI_ISL_672867, EPI_ISL_672868, EPI_ISL_672869, EPI_ISL_672870, EPI_ISL_672871, EPI_ISL_672872, EPI_ISL_672873, EPI_ISL_672874, EPI_ISL_672875, EPI_ISL_672876, EPI_ISL_672877, EPI_ISL_672878, EPI_ISL_672879, EPI_ISL_672880, EPI_ISL_672881, EPI_ISL_672882, EPI_ISL_672883, EPI_ISL_672884, EPI_ISL_672885, EPI_ISL_672886, EPI_ISL_672887, EPI_ISL_672888, EPI_ISL_672889, EPI_ISL_672890, EPI_ISL_672891, EPI_ISL_672892, EPI_ISL_672893, EPI_ISL_672894, EPI_ISL_672895, EPI_ISL_672896, EPI_ISL_672897, EPI_ISL_672898, EPI_ISL_672899, EPI_ISL_672900, EPI_ISL_672901, EPI_ISL_672902, EPI_ISL_672903, EPI_ISL_672904, EPI_ISL_672905, EPI_ISL_672906, EPI_ISL_672907, EPI_ISL_672908, EPI_ISL_672909, EPI_ISL_672910, EPI_ISL_672911			
see above	Lighthouse Lab in Glasgow	Wellcome Sanger Institute for the COVID-19 Genomics UK (COG-UK) Consortium	Harper VanSteenhouse, Yumi Kasai, David Gray, Carol Clugston, Anna Dominiczak and Alex Alderton, Roberto Amato, Sonia Goncalves, Ewan Harrison, David K. Jackson, Ian Johnston, Dominic Kwiatkowski, Cordelia Langford, John Sillitoe on behalf of the Wellcome Sanger Institute COVID-19 Surveillance Team
EPI_ISL_673284, EPI_ISL_673288, EPI_ISL_673443, EPI_ISL_673483, EPI_ISL_673597, EPI_ISL_673629, EPI_ISL_673705, EPI_ISL_673771	Lighthouse Lab in Cambridge	Wellcome Sanger Institute for the COVID-19 Genomics UK (COG-UK) Consortium	Rob Howes, The Lighthouse Lab in Cambridge and Alex Alderton, Roberto Amato, Sonia Goncalves, Ewan Harrison, David K. Jackson, Ian Johnston, Dominic Kwiatkowski, Cordelia Langford, John Sillitoe on behalf of the Wellcome Sanger Institute COVID-19 Surveillance Team
EPI_ISL_676511	Respiratory Virus Unit, Microbiology Services Colindale, Public Health England	COVID-19 Genomics UK (COG-UK) Consortium	PHE Covid Sequencing Team
EPI_ISL_678383, EPI_ISL_678384, EPI_ISL_678385	Area of Virology, Serology and Virology Division (SAViD), New South Wales Health Pathology Randwick	Virology Research Laboratory; Area of Virology, Serology and Virology Division (SAViD), New South Wales Health Pathology Randwick	Foster, C.; Au, J.; Ruiz Silva, M.; Deveson, I.; Bull, R.; Van Hal, S.; Rawlinson, W.
EPI_ISL_678548, EPI_ISL_678549, EPI_ISL_678550, EPI_ISL_678551, EPI_ISL_678552, EPI_ISL_678553, EPI_ISL_678554, EPI_ISL_678555, EPI_ISL_678556, EPI_ISL_678557, EPI_ISL_678558, EPI_ISL_678559, EPI_ISL_678560, EPI_ISL_678561, EPI_ISL_678562, EPI_ISL_678563, EPI_ISL_678564, EPI_ISL_678565, EPI_ISL_678567, EPI_ISL_678568, EPI_ISL_678571, EPI_ISL_678572, EPI_ISL_678573, EPI_ISL_678576, EPI_ISL_678577, EPI_ISL_678578, EPI_ISL_678579, EPI_ISL_678580, EPI_ISL_678581, EPI_ISL_678582, EPI_ISL_678583, EPI_ISL_678584, EPI_ISL_678585, EPI_ISL_678586, EPI_ISL_678587, EPI_ISL_678588, EPI_ISL_678589, EPI_ISL_678590, EPI_ISL_678591, EPI_ISL_678592, EPI_ISL_678593, EPI_ISL_678594, EPI_ISL_678595, EPI_ISL_678596, EPI_ISL_678597			
see above	Netcare	KRISP, KZN Research Innovation and Sequencing Platform	Giandhari J, Pillay S, Lessells R, ChimukangaraB, Mdlalose K, York D, Khan S, Tegally H, Wilkinson E, de Oliveira T
EPI_ISL_678605, EPI_ISL_678607, EPI_ISL_678613, EPI_ISL_678614, EPI_ISL_678616, EPI_ISL_678617, EPI_ISL_678618	NHLS-IALCH	KRISP, KZN Research Innovation and Sequencing Platform	Giandhari J, Pillay S, Lessells R, ChimukangaraB, Mdlalose K, York D, Khan S, Tegally H, Wilkinson E, de Oliveira T
EPI_ISL_678706, EPI_ISL_678709, EPI_ISL_678710, EPI_ISL_678711, EPI_ISL_678713, EPI_ISL_678715, EPI_ISL_678716, EPI_ISL_678717, EPI_ISL_678718, EPI_ISL_678719, EPI_ISL_678720, EPI_ISL_678721, EPI_ISL_678722, EPI_ISL_678723, EPI_ISL_678724, EPI_ISL_678725, EPI_ISL_678726, EPI_ISL_678727, EPI_ISL_678728, EPI_ISL_678729, EPI_ISL_678730, EPI_ISL_678731, EPI_ISL_678732, EPI_ISL_678733, EPI_ISL_678734, EPI_ISL_678735, EPI_ISL_678736, EPI_ISL_678737, EPI_ISL_678738, EPI_ISL_678739, EPI_ISL_678740, EPI_ISL_678741, EPI_ISL_678742, EPI_ISL_678743, EPI_ISL_678744, EPI_ISL_678745, EPI_ISL_678746, EPI_ISL_678749, EPI_ISL_678750, EPI_ISL_678752, EPI_ISL_678753, EPI_ISL_678755, EPI_ISL_678760, EPI_ISL_678761			
see above	Respiratory Virus Unit, Microbiology Services Colindale, Public Health England	COVID-19 Genomics UK (COG-UK) Consortium	PHE Covid Sequencing Team
EPI_ISL_679162, EPI_ISL_679163, EPI_ISL_679164, EPI_ISL_679165, EPI_ISL_679166, EPI_ISL_679167, EPI_ISL_679168, EPI_ISL_679169, EPI_ISL_679170, EPI_ISL_679171, EPI_ISL_679172, EPI_ISL_679173, EPI_ISL_679174, EPI_ISL_679175, EPI_ISL_679176, EPI_ISL_679179, EPI_ISL_679181, EPI_ISL_679183, EPI_ISL_679185, EPI_ISL_679187, EPI_ISL_679189, EPI_ISL_679191, EPI_ISL_679196, EPI_ISL_679197, EPI_ISL_679198, EPI_ISL_679199, EPI_ISL_679200, EPI_ISL_679201, EPI_ISL_679202, EPI_ISL_679203, EPI_ISL_679204, EPI_ISL_679206, EPI_ISL_679208, EPI_ISL_679210, EPI_ISL_679212, EPI_ISL_679214, EPI_ISL_679216, EPI_ISL_679218			
see above	Department of Pathology, University of Cambridge	COVID-19 Genomics UK (COG-UK) Consortium	Aminu S. Jahun, Yasmin Chaudhry, Grant Hall, Iliana Georgana, Myra Hosmillo, Martin D. Curran, Malte Pinckert, Surendra Parmar, Ian Goodfellow
EPI_ISL_679292, EPI_ISL_679293, EPI_ISL_679294, EPI_ISL_679296, EPI_ISL_679308, EPI_ISL_679309, EPI_ISL_679310, EPI_ISL_679311	Queens Medical Centre, Clinical Microbiology Department / DeepSeq Nottingham	COVID-19 Genomics UK (COG-UK) Consortium	Gemma Clark, Wendy Smith, Manjinder Khakh, Vicki M Fleming, Michelle M Lister, Hannah Howson-Wells, Jonathan Ball, Patrick McClure, Joseph Chappell, Theocharis Tsoleridis, Nadine Holmes, Matthew Carlisle, Christopher Moore, Fei Sang, Johnny Debebe, Victoria Wright, Matthew Loose
EPI_ISL_679416, EPI_ISL_679417, EPI_ISL_679418, EPI_ISL_679419, EPI_ISL_679420, EPI_ISL_679421, EPI_ISL_679430, EPI_ISL_679431, EPI_ISL_679434, EPI_ISL_679435, EPI_ISL_679436, EPI_ISL_679437, EPI_ISL_679438, EPI_ISL_679439			
see above	University College London Hospital	COVID-19 Genomics UK (COG-UK) Consortium	Judith Heaney, Matthew Byott, Catherine Houlihan, Dan Frampton, Stuart Kirk, Moira Spyer and Eleni Nastouli

EPI_ISL_679545, EPI_ISL_679546, EPI_ISL_679547, EPI_ISL_679548, EPI_ISL_679549, EPI_ISL_679575, EPI_ISL_679576, EPI_ISL_679577, EPI_ISL_679578, EPI_ISL_679579, EPI_ISL_679580, EPI_ISL_679581, EPI_ISL_679582, EPI_ISL_679583, EPI_ISL_679585, EPI_ISL_679586, EPI_ISL_679588, EPI_ISL_679589, EPI_ISL_679590, EPI_ISL_679591, EPI_ISL_679592, EPI_ISL_679596			
see above	University College London, Great Ormond Street Hospital for Children NHS Foundation Trust, Imperial College Healthcare NHS Trust	COVID-19 Genomics UK (COG-UK) Consortium	Sergi Castellano, Rachel Williams, Mark Kristiansen, Paola Resende Silva, Sunando Roy, Tony Brooks, Helena Tutill, Paola Niola, Patricia Dyal, Charlotte Williams, Leysa Forrest, Yasmin Panchbhaya, Jacqueline Findlay, Samuel Weeks, Julianne Brown, Kathryn Harris, Paul Randell, James Price, Alison Holmes, Judith Breuer
EPI_ISL_679933	Queens Medical Centre, Clinical Microbiology Department / DeepSeq Nottingham	COVID-19 Genomics UK (COG-UK) Consortium	Gemma Clark, Wendy Smith, Manjinder Khakh, Vicki M Fleming, Michelle M Lister, Hannah Howson-Wells, Jonathan Ball, Patrick McClure, Joseph Chappell, Theocharis Tsoleridis, Nadine Holmes, Matthew Carlisle, Christopher Moore, Fei Sang, Johnny Debebe, Victoria Wright, Matthew Loose
EPI_ISL_680102, EPI_ISL_680103, EPI_ISL_680104, EPI_ISL_680105, EPI_ISL_680106, EPI_ISL_680107, EPI_ISL_680108, EPI_ISL_680109, EPI_ISL_680113, EPI_ISL_680114, EPI_ISL_680119, EPI_ISL_680128, EPI_ISL_680137, EPI_ISL_680187, EPI_ISL_680192			
see above	Virology Department, Sheffield Teaching Hospitals NHS Foundation Trust/Department of Infection, Immunity and Cardiovascular Disease, The Medical School, University of Sheffield	COVID-19 Genomics UK (COG-UK) Consortium	Thushan de Silva, Matthew Parker, Nikki Smith, Adri Angyal, Rebecca Brown, Luke Green, Rachel Tucker, Paul Parsons, Danielle Groves, Katie Johnson, Laura Carrilero, Alex Keeley, Dave Partridge, Matthew Wyles, Benjamin Lindsey, Mehmet Yavuz, Mohammad Raza, Cariad Evans
EPI_ISL_680449, EPI_ISL_680450, EPI_ISL_680451, EPI_ISL_680452	West of Scotland Specialist Virology Centre, NHSGGC / MRC-University of Glasgow Centre for Virus Research	COVID-19 Genomics UK (COG-UK) Consortium	Ana da Silva Filipe, Natasha Johnson, Kathy Smollett, Daniel Mair, Stephen Carmichael, Alice Broos, Lily Tong, Jenna Nichols, Kyriaki Nomikou; Sarah McDonald; Richard Orton, Joseph Hughes, Sreenu Vattipally, David L Robertson; Alasdair MacLean, Rory Gunson; Sharif Shaaban, Matthew Holden; Rachel Blacow, Guy Mollett, Kathy Li, James Shepherd, Antonia Ho, Emma Thomson
EPI_ISL_680461, EPI_ISL_680462, EPI_ISL_680463, EPI_ISL_680541, EPI_ISL_680542, EPI_ISL_680543, EPI_ISL_680548, EPI_ISL_680549, EPI_ISL_680550, EPI_ISL_680551, EPI_ISL_680552, EPI_ISL_680553, EPI_ISL_680554, EPI_ISL_680555			
see above	Virology Department, Royal Infirmary of Edinburgh, NHS Lothian / School of Biological Sciences, University of Edinburgh / Institute of Genetics and Molecular Medicine, University of Edinburgh	COVID-19 Genomics UK (COG-UK) Consortium	McHugh M, Dewar R, Rooke S, Gallagher M, Balcaza C, O'Toole Á, Scher E, Hill V, McCrone JT, Colquhoun R, Yu X, Jackson B, Rambaut A, Williams TC, Templeton K
EPI_ISL_680591, EPI_ISL_680610, EPI_ISL_680654, EPI_ISL_680662, EPI_ISL_680663, EPI_ISL_680664, EPI_ISL_680665, EPI_ISL_680666, EPI_ISL_680667, EPI_ISL_680668, EPI_ISL_680669, EPI_ISL_680670			
see above	Wales Specialist Virology Centre Sequencing lab: Pathogen Genomics Unit	COVID-19 Genomics UK (COG-UK) Consortium	Catherine Moore, Johnathan Evans, Laura Gifford, Malorie Perry, Simon Cottrell, Angela Marchbank, Alec Birchley, Alexander Adams, Amy Gaskin, Bree Gatica-Wilcox, Jason Coombes, Joel Southgate, Lauren Gilbert, Lee Graham, Nicole Pacchiarini, Sara Kumziene-Summerhayes, Sarah Taylor, Sophie Jones, Sara Rey, Matthew Bull, Joanne Watkins, Sally Corden, Tom Connor
EPI_ISL_681313, EPI_ISL_681314	Communicable Disease Laboratory, Public Health Directorate	Communicable Disease Laboratory, Public Health Directorate	Alwasti,H., Altaif,Z., AlHujairi,Z., AlAbbas,Z.
EPI_ISL_681325, EPI_ISL_681326, EPI_ISL_681327, EPI_ISL_681328, EPI_ISL_681329, EPI_ISL_681330, EPI_ISL_681331, EPI_ISL_681332, EPI_ISL_681333, EPI_ISL_681334, EPI_ISL_681335, EPI_ISL_681336, EPI_ISL_681337, EPI_ISL_681338, EPI_ISL_681339, EPI_ISL_681340, EPI_ISL_681341, EPI_ISL_681342, EPI_ISL_681343, EPI_ISL_681344, EPI_ISL_681345, EPI_ISL_681346, EPI_ISL_681347, EPI_ISL_681348, EPI_ISL_681349, EPI_ISL_681350, EPI_ISL_681351, EPI_ISL_681352, EPI_ISL_681353, EPI_ISL_681354, EPI_ISL_681355, EPI_ISL_681356, EPI_ISL_681357, EPI_ISL_681358, EPI_ISL_681359, EPI_ISL_681360, EPI_ISL_681361, EPI_ISL_681362, EPI_ISL_681363, EPI_ISL_681364, EPI_ISL_681365, EPI_ISL_681366, EPI_ISL_681367, EPI_ISL_681368, EPI_ISL_681369, EPI_ISL_681370, EPI_ISL_681371, EPI_ISL_681372, EPI_ISL_681373, EPI_ISL_681374, EPI_ISL_681375, EPI_ISL_681376, EPI_ISL_681377, EPI_ISL_681378, EPI_ISL_681379, EPI_ISL_681380, EPI_ISL_681381, EPI_ISL_681382, EPI_ISL_681383, EPI_ISL_681384, EPI_ISL_681385, EPI_ISL_681386, EPI_ISL_681387, EPI_ISL_681388, EPI_ISL_681389, EPI_ISL_681390, EPI_ISL_681391, EPI_ISL_681392, EPI_ISL_681393, EPI_ISL_681394, EPI_ISL_681395, EPI_ISL_681396, EPI_ISL_681397, EPI_ISL_681398, EPI_ISL_681399, EPI_ISL_681400, EPI_ISL_681401, EPI_ISL_681402, EPI_ISL_681403, EPI_ISL_681404, EPI_ISL_681405, EPI_ISL_681406, EPI_ISL_681407, EPI_ISL_681408, EPI_ISL_681409, EPI_ISL_681410, EPI_ISL_681411, EPI_ISL_681412, EPI_ISL_681413, EPI_ISL_681414, EPI_ISL_681415, EPI_ISL_681416, EPI_ISL_681417, EPI_ISL_681418, EPI_ISL_681419, EPI_ISL_681420, EPI_ISL_681421, EPI_ISL_681422, EPI_ISL_681423, EPI_ISL_681424, EPI_ISL_681425, EPI_ISL_681426, EPI_ISL_681427, EPI_ISL_681428, EPI_ISL_681429, EPI_ISL_681430, EPI_ISL_681431, EPI_ISL_681432, EPI_ISL_681433, EPI_ISL_681434, EPI_ISL_681435, EPI_ISL_681436, EPI_ISL_681437, EPI_ISL_681438, EPI_ISL_681439, EPI_ISL_681440, EPI_ISL_681441, EPI_ISL_681442, EPI_ISL_681443, EPI_ISL_681444, EPI_ISL_681445, EPI_ISL_681446, EPI_ISL_681447, EPI_ISL_681448, EPI_ISL_681449, EPI_ISL_681450, EPI_ISL_681451, EPI_ISL_681452, EPI_ISL_681453, EPI_ISL_681454, EPI_ISL_681455, EPI_ISL_681456, EPI_ISL_681457, EPI_ISL_681458, EPI_ISL_681459, EPI_ISL_681460, EPI_ISL_681461, EPI_ISL_681462, EPI_ISL_681463, EPI_ISL_681464, EPI_ISL_681465, EPI_ISL_681467, EPI_ISL_681468, EPI_ISL_681469, EPI_ISL_681470, EPI_ISL_681471, EPI_ISL_681472, EPI_ISL_681473, EPI_ISL_681474, EPI_ISL_681475, EPI_ISL_681476, EPI_ISL_681477, EPI_ISL_681478, EPI_ISL_681479, EPI_ISL_681480, EPI_ISL_681481, EPI_ISL_681482, EPI_ISL_681483, EPI_ISL_681484, EPI_ISL_681485, EPI_ISL_681486, EPI_ISL_681487, EPI_ISL_681488, EPI_ISL_681489, EPI_ISL_681490, EPI_ISL_681491, EPI_ISL_681492, EPI_ISL_681493, EPI_ISL_681494, EPI_ISL_681495, EPI_ISL_681496, EPI_ISL_681497, EPI_ISL_681498, EPI_ISL_681499, EPI_ISL_681500, EPI_ISL_681501, EPI_ISL_681502, EPI_ISL_681503, EPI_ISL_681504, EPI_ISL_681505, EPI_ISL_681506, EPI_ISL_681507, EPI_ISL_681508, EPI_ISL_681509, EPI_ISL_681510, EPI_ISL_681511, EPI_ISL_681512, EPI_ISL_681513, EPI_ISL_681514, EPI_ISL_681515, EPI_ISL_681516, EPI_ISL_681517, EPI_ISL_681518, EPI_ISL_681519, EPI_ISL_681520, EPI_ISL_681521, EPI_ISL_681522, EPI_ISL_681523, EPI_ISL_681524, EPI_ISL_681525, EPI_ISL_681526, EPI_ISL_681527, EPI_ISL_681528, EPI_ISL_681529, EPI_ISL_681530, EPI_ISL_681531, EPI_ISL_681532, EPI_ISL_681533, EPI_ISL_681534, EPI_ISL_681535, EPI_ISL_681536, EPI_ISL_681537, EPI_ISL_681538, EPI_ISL_681539, EPI_ISL_681540, EPI_ISL_681541, EPI_ISL_681542, EPI_ISL_681543, EPI_ISL_681544, EPI_ISL_681545, EPI_ISL_681546, EPI_ISL_681547, EPI_ISL_681548, EPI_ISL_681549, EPI_ISL_681550, EPI_ISL_681551, EPI_ISL_681552, EPI_ISL_681553, EPI_ISL_681554, EPI_ISL_681555, EPI_ISL_681556, EPI_ISL_681557, EPI_ISL_681558, EPI_ISL_681559, EPI_ISL_681560, EPI_ISL_681561, EPI_ISL_681562, EPI_ISL_681563, EPI_ISL_681564, EPI_ISL_681565, EPI_ISL_681566, EPI_ISL_681567, EPI_ISL_681568, EPI_ISL_681569, EPI_ISL_681570, EPI_ISL_681571, EPI_ISL_681572, EPI_ISL_681573, EPI_ISL_681574, EPI_ISL_681575, EPI_ISL_681576, EPI_ISL_681577, EPI_ISL_681578, EPI_ISL_681579, EPI_ISL_681580, EPI_ISL_681581, EPI_ISL_681582, EPI_ISL_681583, EPI_ISL_681584, EPI_ISL_681585, EPI_ISL_681586, EPI_ISL_681587, EPI_ISL_681588, EPI_ISL_681589, EPI_ISL_681590, EPI_ISL_681591, EPI_ISL_681592, EPI_ISL_681593, EPI_ISL_681594, EPI_ISL_681595, EPI_ISL_681596, EPI_ISL_681597, EPI_ISL_681598, EPI_ISL_681599, EPI_ISL_681600, EPI_ISL_681601, EPI_ISL_681602, EPI_ISL_681603, EPI_ISL_681604, EPI_ISL_681605, EPI_ISL_681606, EPI_ISL_681607, EPI_ISL_681608, EPI_ISL_681609, EPI_ISL_681610, EPI_ISL_681611, EPI_ISL_681612, EPI_ISL_681613, EPI_ISL_681614, EPI_ISL_681615, EPI_ISL_681616, EPI_ISL_681617, EPI_ISL_681618, EPI_ISL_681619, EPI_ISL_681620, EPI_ISL_681621, EPI_ISL_681622, EPI_ISL_681623, EPI_ISL_681624, EPI_ISL_681625, EPI_ISL_681626, EPI_ISL_681627, EPI_ISL_681628, EPI_ISL_681629, EPI_ISL_681630, EPI_ISL_681631, EPI_ISL_681632, EPI_ISL_681633, EPI_ISL_681634, EPI_ISL_681635, EPI_ISL_681636, EPI_ISL_681637, EPI_ISL_681638, EPI_ISL_681639, EPI_ISL_681640, EPI_ISL_681641, EPI_ISL_681642, EPI_ISL_681643, EPI_ISL_681644, EPI_ISL_681645, EPI_ISL_681646, EPI_ISL_681647, EPI_ISL_681648, EPI_ISL_681649, EPI_ISL_681650, EPI_ISL_681651, EPI_ISL_681652, EPI_ISL_681653, EPI_ISL_681654, EPI_ISL_681655			
see above	Lighthouse Lab in Milton Keynes	Wellcome Sanger Institute for the COVID-19 Genomics UK (COG-UK) Consortium	The Lighthouse Lab in Milton Keynes and Alex Alderton, Roberto Amato, Sonia Goncalves, Ewan Harrison, David K. Jackson, Ian Johnston, Dominic Kwiatkowski, Cordelia Langford, John Sillitoe on behalf of the Wellcome Sanger Institute COVID-19 Surveillance Team
EPI_ISL_682279	Middlemore Hospital	Institute of Environmental Science and Research (ESR)	Xiaoyun Ren, Matt Storey, Nikki Freed, Muhammad Faisal, Jing Wang, Hermes Perez, Anja Werno, Antje van der Linden, Arlo Upton, Chris Mansell, David Hammer, Dragana Drinkovic, Gary McAuliffe, Hana Sofia Andersson, James Ussher, Jill Sherwood, Josh Freeman, Julia Howard, Juliet Elvy, Mary DeAlmeida, Matt Blakiston, Matthew Rogers, Max Bloomfield, Michael Addidle, Michelle Balm, Sally Roberts, Sarah Jefferies, Sharmini Muttaiyah, Susan Morpeth, Susan Taylor, Timothy Blackmore, Vani Sathyendran, Veronica Playle, Virginia Hope, Erasmus Smit, Lauren Jelly, Olin Silander, Joep de Ligt
EPI_ISL_682315, EPI_ISL_682316, EPI_ISL_682319, EPI_ISL_682320, EPI_ISL_682321	Communicable Disease Laboratory, Public Health Directorate	Communicable Disease Laboratory, Public Health Directorate	Alwasti,H., Altaif,Z., AlHujairi,Z., AlAbbas,Z.
EPI_ISL_682363, EPI_ISL_682364, EPI_ISL_682365, EPI_ISL_682366, EPI_ISL_682367, EPI_ISL_682368, EPI_ISL_682369, EPI_ISL_682370, EPI_ISL_682371, EPI_ISL_682372, EPI_ISL_682373, EPI_ISL_682374, EPI_ISL_682375, EPI_ISL_682376, EPI_ISL_682377, EPI_ISL_682378, EPI_ISL_682379, EPI_ISL_682380, EPI_ISL_682381, EPI_ISL_682382, EPI_ISL_682383, EPI_ISL_682384, EPI_ISL_682385, EPI_ISL_682386, EPI_ISL_682387, EPI_ISL_682388, EPI_ISL_682389, EPI_ISL_682390, EPI_ISL_682391, EPI_ISL_682392, EPI_ISL_682393, EPI_ISL_682394, EPI_ISL_682395, EPI_ISL_682396, EPI_ISL_682397, EPI_ISL_682398, EPI_ISL_682399, EPI_ISL_682400, EPI_ISL_682401, EPI_ISL_682402, EPI_ISL_682403, EPI_ISL_682404, EPI_ISL_682405, EPI_ISL_682406, EPI_ISL_682407, EPI_ISL_682408, EPI_ISL_682409, EPI_ISL_682410, EPI_ISL_682411, EPI_ISL_682412, EPI_ISL_682413, EPI_ISL_682414, EPI_ISL_682415, EPI_ISL_682416, EPI_ISL_682417, EPI_ISL_682418, EPI_ISL_682419, EPI_ISL_682420, EPI_ISL_682421, EPI_ISL_682422, EPI_ISL_682423, EPI_ISL_682424, EPI_ISL_682425, EPI_ISL_682426, EPI_ISL_682427, EPI_ISL_682428, EPI_ISL_682429, EPI_ISL_682430, EPI_ISL_682431, EPI_ISL_682432, EPI_ISL_682433, EPI_ISL_682434, EPI_ISL_682435, EPI_ISL_682436, EPI_ISL_682437, EPI_ISL_682438, EPI_ISL_682439, EPI_ISL_682440, EPI_ISL_682441, EPI_ISL_682442, EPI_ISL_682443, EPI_ISL_682444, EPI_ISL_682445, EPI_ISL_682446, EPI_ISL_682447, EPI_ISL_682448, EPI_ISL_682449, EPI_ISL_682450, EPI_ISL_682451, EPI_ISL_682452, EPI_ISL_682453, EPI_ISL_682454, EPI_ISL_682455, EPI_ISL_682456, EPI_ISL_682457, EPI_ISL_682458, EPI_ISL_682459, EPI_ISL_682460, EPI_ISL_682461, EPI_ISL_682462, EPI_ISL_682463, EPI_ISL_682464, EPI_ISL_682465, EPI_ISL_682466, EPI_ISL_682467, EPI_ISL_682468, EPI_ISL_682469, EPI_ISL_682470, EPI_ISL_682471, EPI_ISL_682472, EPI_ISL_682473, EPI_ISL_682474, EPI_ISL_682475, EPI_ISL_682476, EPI_ISL_682477, EPI_ISL_682478, EPI_ISL_682479, EPI_ISL_682480, EPI_ISL_682481, EPI_ISL_682482, EPI_ISL_682483, EPI_ISL_682484, EPI_ISL_682485, EPI_ISL_682486, EPI_ISL_682487, EPI_ISL_682488, EPI_ISL_682489, EPI_ISL_682490, EPI_ISL_682491, EPI_ISL_682492, EPI_ISL_682493, EPI_ISL_682494, EPI_ISL_682495, EPI_ISL_682496, EPI_ISL_682497, EPI_ISL_682498, EPI_ISL_682499, EPI_ISL_682500, EPI_ISL_682501, EPI_ISL_682502, EPI_ISL_682503, EPI_ISL_682504, EPI_ISL_682505, EPI_ISL_682506, EPI_ISL_682507, EPI_ISL_682508, EPI_ISL_682509, EPI_ISL_682510, EPI_ISL_682511, EPI_ISL_682512, EPI_ISL_682513, EPI_ISL_682514, EPI_ISL_682515, EPI_ISL_682516, EPI_ISL_682517, EPI_ISL_682518, EPI_ISL_682519, EPI_ISL_682520, EPI_ISL_682521, EPI_ISL_682522, EPI_ISL_682523, EPI_ISL_682524, EPI_ISL_682525, EPI_ISL_682526, EPI_ISL_682527, EPI_ISL_682528, EPI_ISL_682529, EPI_ISL_682530, EPI_ISL_682531, EPI_ISL_682532, EPI_ISL_682533, EPI_ISL_682534, EPI_ISL_682535, EPI_ISL_682536, EPI_ISL_682537, EPI_ISL_682538, EPI_ISL_682539, EPI_ISL_682540, EPI_ISL_682541, EPI_ISL_682542, EPI_ISL_682543, EPI_ISL_682544, EPI_ISL_682545, EPI_ISL_682546, EPI_ISL_682547, EPI_ISL_682548, EPI_ISL_682549, EPI_ISL_682550, EPI_ISL_682551, EPI_ISL_682552, EPI_ISL_682553, EPI_ISL_682554, EPI_ISL_682555, EPI_ISL_682556, EPI_ISL_682557, EPI_ISL_682558, EPI_ISL_682559, EPI_ISL_682560, EPI_ISL_682561, EPI_ISL_682562, EPI_ISL_682563, EPI_ISL_682564, EPI_ISL_682565, EPI_ISL_682566, EPI_ISL_682567, EPI_ISL_682568, EPI_ISL_682569, EPI_ISL_682570, EPI_ISL_682571, EPI_ISL_682572, EPI_ISL_682573, EPI_ISL_682574, EPI_ISL_682575, EPI_ISL_682576, EPI_ISL_682577, EPI_ISL_682578, EPI_ISL_682579, EPI_ISL_682580, EPI_ISL_682581, EPI_ISL_682582, EPI_ISL_682583, EPI_ISL_682584, EPI_ISL_682585, EPI_ISL_682586, EPI_ISL_682587, EPI_ISL_682588, EPI_ISL_682589, EPI_ISL_682590, EPI_ISL_682591, EPI_ISL_682592, EPI_ISL_682593, EPI_ISL_682594, EPI_ISL_682595, EPI_ISL_682596, EPI_ISL_682597, EPI_ISL_682598, EPI_ISL_682599, EPI_ISL_682600, EPI_ISL_682601, EPI_ISL_682602, EPI_ISL_682603, EPI_ISL_682604, EPI_ISL_682605, EPI_ISL_682606, EPI_ISL_682607, EPI_ISL_682608, EPI_ISL_682609, EPI_ISL_682610, EPI_ISL_682611, EPI_ISL_682612, EPI_ISL_682613, EPI_ISL_682614, EPI_ISL_682615, EPI_ISL_682616, EPI_ISL_682617, EPI_ISL_682618, EPI_ISL_682619, EPI_ISL_682620, EPI_ISL_682621, EPI_ISL_682622, EPI_ISL_682623, EPI_ISL_682624, EPI_ISL_682625, EPI_ISL_682626, EPI_ISL_682627, EPI_ISL_682628, EPI_ISL_682629, EPI_ISL_682630, EPI_ISL_682631, EPI_ISL_682632, EPI_ISL_682633, EPI_ISL_682634, EPI_ISL_682635, EPI_ISL_682636, EPI_ISL_682637, EPI_ISL_682638, EPI_ISL_682639, EPI_ISL_682640, EPI_ISL_682641, EPI_ISL_682642, EPI_ISL_682643, EPI_ISL_682644, EPI_ISL_682645, EPI_ISL_682646, EPI_ISL_682647, EPI_ISL_682648, EPI_ISL_682649, EPI_ISL_682650, EPI_ISL_682651, EPI_ISL_682652, EPI_ISL_682653, EPI_ISL_682654, EPI_ISL_682655, EPI_ISL_682656, EPI_ISL_682657, EPI_ISL_682658, EPI_ISL_682659, EPI_ISL_682660, EPI_ISL_682661, EPI_ISL_682662, EPI_ISL_682663, EPI_ISL_682664, EPI_ISL_682665, EPI_ISL_682666, EPI_ISL_682667, EPI_ISL_682668, EPI_ISL_682669, EPI_ISL_682670, EPI_ISL_682671, EPI_ISL_682672, EPI_ISL_682673, EPI_ISL_682674, EPI_ISL_682675, EPI_ISL_682676, EPI_ISL_682677, EPI_ISL_682678, EPI_ISL_682679, EPI_ISL_682680, EPI_ISL_682681, EPI_ISL_682682, EPI_ISL_682683, EPI_ISL_682684, EPI_ISL_682685, EPI_ISL_682686, EPI_ISL_682687, EPI_ISL_682688, EPI_ISL_682689, EPI_ISL_682690, EPI_ISL_682691, EPI_ISL_682692, EPI_ISL_682693, EPI_ISL_682694, EPI_ISL_682695, EPI_ISL_682696, EPI_ISL_682697, EPI_ISL_682698, EPI_ISL_682699, EPI_ISL_682700, EPI_ISL_682701, EPI_ISL_682702, EPI_ISL_682703, EPI_ISL_682704, EPI_ISL_682705, EPI_ISL_682706, EPI_ISL_682707, EPI_ISL_682708, EPI_ISL_682709, EPI_ISL_682710, EPI_ISL_682711, EPI_ISL_682712, EPI_ISL_682713, EPI_ISL_682714, EPI_ISL_682715, EPI_ISL_682716, EPI_ISL_682717, EPI_ISL_682718, EPI_ISL_682719, EPI_ISL_682720, EPI_ISL_682721, EPI_ISL_682722, EPI_ISL_682723, EPI_ISL_682724, EPI_ISL_682725, EPI_ISL_682726, EPI_ISL_682727, EPI_ISL_682728, EPI_ISL_682729, EPI_ISL_682730, EPI_ISL_682731, EPI_ISL_682732, EPI_ISL_682733, EPI_ISL_682734, EPI_ISL_682735, EPI_ISL_682736, EPI_ISL_682737, EPI_ISL_682738, EPI_ISL_682739, EPI_ISL_682740, EPI_ISL_682741, EPI_ISL_682742, EPI_ISL_682743, EPI_ISL_682744, EPI_ISL_682745, EPI_ISL_682746, EPI_ISL_682747, EPI_ISL_682748, EPI_ISL_682749, EPI_ISL_682750, EPI_ISL_682751, EPI_ISL_682752, EPI_ISL_682753, EPI_ISL_682754, EPI_ISL_682755, EPI_ISL_682756, EPI_ISL_682757, EPI_ISL_682758, EPI_ISL_682759, EPI_ISL_682760, EPI_ISL_682761, EPI_ISL_682762, EPI_ISL_682763, EPI_ISL_682764, EPI_ISL_682765, EPI_ISL_682766, EPI_ISL_682767, EPI_ISL_682768, EPI_ISL_682769, EPI_ISL_682770, EPI_ISL_682771, EPI_ISL_682772, EPI_ISL_682773, EPI_ISL_682774, EPI_ISL_682775, EPI_ISL_682776, EPI_ISL_682777, EPI_ISL_682778, EPI_ISL_682779, EPI_ISL_682780, EPI_ISL_682781, EPI_ISL_682782, EPI_ISL_682783, EPI_ISL_682784, EPI_ISL_682785, EPI_ISL_682786, EPI_ISL_682787, EPI_ISL_682788, EPI_ISL_682789, EPI_ISL_682790, EPI_ISL_682791, EPI_ISL_682792, EPI_ISL_682793, EPI_ISL_682794, EPI_ISL_682795, EPI_ISL_682796, EPI_ISL_682797, EPI_ISL_682798, EPI_ISL_682799, EPI_ISL_682800, EPI_ISL_682801, EPI_ISL_682802, EPI_ISL_682803, EPI_ISL_682804, EPI_ISL_682805, EPI_ISL_682806, EPI_ISL_682807, EPI_ISL_682808, EPI_ISL_682809, EPI_ISL_682810, EPI_ISL_682811, EPI_ISL_682812, EPI_ISL_682813, EPI_ISL_682814, EPI_ISL_682815, EPI_ISL_682816, EPI_ISL_682817, EPI_ISL_682818, EPI_ISL_682819, EPI_ISL_682820, EPI_ISL_682821, EPI_ISL_682822, EPI_ISL_682823, EPI_ISL_682824, EPI_ISL_682825, EPI_ISL_682826, EPI_ISL_682827, EPI_ISL_682828, EPI_ISL_682829, EPI_ISL_682830, EPI_ISL_682831, EPI_ISL_682832, EPI_ISL_682833, EPI_ISL_682834, EPI_ISL_682835, EPI_ISL_682836, EPI_ISL_682837, EPI_ISL_682838, EPI_ISL_682839, EPI_ISL_682840, EPI_ISL_682841, EPI_ISL_682842, EPI_ISL_682843, EPI_ISL_682844, EPI_ISL_682845, EPI_ISL_682846, EPI_ISL_682847, EPI_ISL_682848,			

EPI_ISL_682849, EPI_ISL_682850, EPI_ISL_682851, EPI_ISL_682852, EPI_ISL_682853, EPI_ISL_682854, EPI_ISL_682855, EPI_ISL_682856, EPI_ISL_682857, EPI_ISL_682858, EPI_ISL_682859, EPI_ISL_682860, EPI_ISL_682861, EPI_ISL_682862, EPI_ISL_682863, EPI_ISL_682864, EPI_ISL_682865, EPI_ISL_682866, EPI_ISL_682867, EPI_ISL_682868, EPI_ISL_682869, EPI_ISL_682870, EPI_ISL_682871, EPI_ISL_682872, EPI_ISL_682873, EPI_ISL_682874, EPI_ISL_682875, EPI_ISL_682876, EPI_ISL_682877, EPI_ISL_682878, EPI_ISL_682879, EPI_ISL_682880, EPI_ISL_682881, EPI_ISL_682882, EPI_ISL_682883, EPI_ISL_682884, EPI_ISL_682885, EPI_ISL_682886, EPI_ISL_682887, EPI_ISL_682888, EPI_ISL_682889, EPI_ISL_682890, EPI_ISL_682891, EPI_ISL_682892, EPI_ISL_682893, EPI_ISL_682894, EPI_ISL_682895, EPI_ISL_682896, EPI_ISL_682897, EPI_ISL_682898, EPI_ISL_682899, EPI_ISL_682900, EPI_ISL_682901, EPI_ISL_682902, EPI_ISL_682903, EPI_ISL_682904, EPI_ISL_682905, EPI_ISL_682906, EPI_ISL_682907, EPI_ISL_682908, EPI_ISL_682909, EPI_ISL_682910, EPI_ISL_682911, EPI_ISL_682912, EPI_ISL_682913, EPI_ISL_682914, EPI_ISL_682915, EPI_ISL_682916, EPI_ISL_682917, EPI_ISL_682918, EPI_ISL_682919, EPI_ISL_682920, EPI_ISL_682921, EPI_ISL_682922, EPI_ISL_682923, EPI_ISL_682924, EPI_ISL_682925, EPI_ISL_682926, EPI_ISL_682927, EPI_ISL_682928, EPI_ISL_682929, EPI_ISL_683161, EPI_ISL_683162, EPI_ISL_683163, EPI_ISL_683164, EPI_ISL_683165, EPI_ISL_683166, EPI_ISL_683255, EPI_ISL_683256, EPI_ISL_683257, EPI_ISL_683258, EPI_ISL_683259, EPI_ISL_683260			
see above	Department of Virus and Microbiological Special Diagnostics, Statens Serum Institut, Copenhagen, Denmark	Albertsen Lab, Department of Chemistry and Bioscience, Aalborg University, Denmark	Danish Covid-19 Genome Consortium
EPI_ISL_683457, EPI_ISL_683475, EPI_ISL_683476, EPI_ISL_683478, EPI_ISL_683484, EPI_ISL_683489, EPI_ISL_683490, EPI_ISL_683534, EPI_ISL_683535, EPI_ISL_683536, EPI_ISL_683537, EPI_ISL_683538, EPI_ISL_683539, EPI_ISL_683540, EPI_ISL_683541, EPI_ISL_683542, EPI_ISL_683543, EPI_ISL_683544, EPI_ISL_683545, EPI_ISL_683546, EPI_ISL_683547, EPI_ISL_683548, EPI_ISL_683549, EPI_ISL_683550, EPI_ISL_683551, EPI_ISL_683552, EPI_ISL_683553, EPI_ISL_683554, EPI_ISL_683555, EPI_ISL_683556, EPI_ISL_683557, EPI_ISL_683558, EPI_ISL_683559, EPI_ISL_683560, EPI_ISL_683561, EPI_ISL_683562, EPI_ISL_683563, EPI_ISL_683564, EPI_ISL_683565			
see above	Respiratory Virus Unit, Microbiology Services Colindale, Public Health England	COVID-19 Genomics UK (COG-UK) Consortium	PHE Covid Sequencing Team
EPI_ISL_683608, EPI_ISL_683609, EPI_ISL_683623, EPI_ISL_683636	Servicio de Microbiología, Laboratori Clínic Metropolitana Nord. Hospital Universitari Germans Trias i Pujol. Institut d'Investigació en Ciències de la Salut Germans Trias i Pujol (IGTP)	SeqCOVID-SPAIN consortium/IBV(CSIC)	Elisa Martró, Antoni E. Bordoy, Anna Not, Adrián Antuori, Anabel Fernández, Nona Romaní, Verónica Saludes, Cristina Casañ and SeqCOVID-SPAIN consortium
EPI_ISL_683689	Hennepin County Medical Center	Minnesota Department of Health, Public Health Laboratory	Alexandra Lorentz, Jacob Garfin, Matt Plumb, and Xiong Wang
EPI_ISL_683729	Minnesota Department of Health, Public Health Laboratory	Minnesota Department of Health, Public Health Laboratory	Alexandra Lorentz, Jacob Garfin, Matt Plumb, and Xiong Wang
EPI_ISL_683753	DOHMH Corona	New York City Public Health Laboratory	Jade Wang, et al.
EPI_ISL_683755	DOHMH PHL	New York City Public Health Laboratory	Jade Wang, et al.
EPI_ISL_683756	DOHMH Central Harlem	New York City Public Health Laboratory	Jade Wang, et al.
EPI_ISL_683757, EPI_ISL_683758	DOHMH Corona	New York City Public Health Laboratory	Jade Wang, et al.
EPI_ISL_683759	DOHMH PHL	New York City Public Health Laboratory	Jade Wang, et al.
EPI_ISL_683764, EPI_ISL_683779	DOHMH Corona	New York City Public Health Laboratory	Jade Wang, et al.
EPI_ISL_683780	DOHMH Chelsea	New York City Public Health Laboratory	Jade Wang, et al.
EPI_ISL_683781, EPI_ISL_683782	DOHMH Jamaica	New York City Public Health Laboratory	Jade Wang, et al.
EPI_ISL_683783	DOHMH PHL	New York City Public Health Laboratory	Jade Wang, et al.
EPI_ISL_683784	DOHMH Riverside	New York City Public Health Laboratory	Jade Wang, et al.
EPI_ISL_683785	DOHMH Corona	New York City Public Health Laboratory	Jade Wang, et al.
EPI_ISL_683786	DOHMH Jamaica	New York City Public Health Laboratory	Jade Wang, et al.
EPI_ISL_683787	DOHMH PHL	New York City Public Health Laboratory	Jade Wang, et al.
EPI_ISL_683788	DOHMH Chelsea	New York City Public Health Laboratory	Jade Wang, et al.
EPI_ISL_683789	DOHMH PHL	New York City Public Health Laboratory	Jade Wang, et al.
EPI_ISL_683790	DOHMH Morrisania	New York City Public Health Laboratory	Jade Wang, et al.
EPI_ISL_683791	DOHMH Jamaica	New York City Public Health Laboratory	Jade Wang, et al.
EPI_ISL_683792	DOHMH Chelsea	New York City Public Health Laboratory	Jade Wang, et al.
EPI_ISL_683837	DOHMH Jamaica	New York City Public Health Laboratory	Jade Wang, et al.
EPI_ISL_683842	DOHMH Central Harlem	New York City Public Health Laboratory	Jade Wang, et al.
EPI_ISL_683847	DOHMH Corona	New York City Public Health Laboratory	Jade Wang, et al.
EPI_ISL_683869	DOHMH Morrisania	New York City Public Health Laboratory	Jade Wang, et al.
EPI_ISL_683871, EPI_ISL_683875	DOHMH PHL	New York City Public Health Laboratory	Jade Wang, et al.
EPI_ISL_683881	DOHMH Corona	New York City Public Health Laboratory	Jade Wang, et al.
EPI_ISL_683927, EPI_ISL_683928	DOHMH Central Harlem	New York City Public Health Laboratory	Jade Wang, et al.
EPI_ISL_683929	DOHMH Corona	New York City Public Health Laboratory	Jade Wang, et al.
EPI_ISL_683930	DOHMH Jamaica	New York City Public Health Laboratory	Jade Wang, et al.
EPI_ISL_683931	DOHMH Chelsea	New York City Public Health Laboratory	Jade Wang, et al.
EPI_ISL_683932	DOHMH Corona	New York City Public Health Laboratory	Jade Wang, et al.
EPI_ISL_683933	DOHMH Central Harlem	New York City Public Health Laboratory	Jade Wang, et al.
EPI_ISL_683934	DOHMH Corona	New York City Public Health Laboratory	Jade Wang, et al.
EPI_ISL_683935	DOHMH Crown Heights	New York City Public Health Laboratory	Jade Wang, et al.
EPI_ISL_683936	DOHMH Chelsea	New York City Public Health Laboratory	Jade Wang, et al.
EPI_ISL_683978, EPI_ISL_683979	DOHMH Corona	New York City Public Health Laboratory	Jade Wang, et al.
EPI_ISL_683980, EPI_ISL_683981	DOHMH Chelsea	New York City Public Health Laboratory	Jade Wang, et al.
EPI_ISL_683982, EPI_ISL_683983, EPI_ISL_683984, EPI_ISL_683985	DOHMH Central Harlem	New York City Public Health Laboratory	Jade Wang, et al.
EPI_ISL_683986	DOHMH Jamaica	New York City Public Health Laboratory	Jade Wang, et al.
EPI_ISL_683987, EPI_ISL_683988	DOHMH PHL	New York City Public Health Laboratory	Jade Wang, et al.
EPI_ISL_683989	DOHMH Chelsea	New York City Public Health Laboratory	Jade Wang, et al.
EPI_ISL_683990	DOHMH Jamaica	New York City Public Health Laboratory	Jade Wang, et al.
EPI_ISL_683991	DOHMH Central Harlem	New York City Public Health Laboratory	Jade Wang, et al.
EPI_ISL_683992	DOHMH Morrisania	New York City Public Health Laboratory	Jade Wang, et al.
EPI_ISL_683993	DOHMH PHL	New York City Public Health Laboratory	Jade Wang, et al.

EPI_ISL_683994	DOHMH Riverside	New York City Public Health Laboratory	Jade Wang, et al.
EPI_ISL_683995	DOHMH PHL	New York City Public Health Laboratory	Jade Wang, et al.
EPI_ISL_683996	DOHMH Fort Greene	New York City Public Health Laboratory	Jade Wang, et al.
EPI_ISL_684028, EPI_ISL_684029, EPI_ISL_684036	Communicable Disease Laboratory, Public Health Directorate	Communicable Disease Laboratory, Public Health Directorate	Alwasti,H., Altaif,Z., AlHujairi,Z., AlAbbas,Z.
EPI_ISL_686601, EPI_ISL_686602, EPI_ISL_686603, EPI_ISL_686604, EPI_ISL_686605, EPI_ISL_686606, EPI_ISL_686607, EPI_ISL_686608, EPI_ISL_686609, EPI_ISL_686610	Respiratory Virus Unit, Microbiology Services Colindale, Public Health England	COVID-19 Genomics UK (COG-UK) Consortium	PHE Covid Sequencing Team
EPI_ISL_692774, EPI_ISL_692776, EPI_ISL_692777, EPI_ISL_692778, EPI_ISL_692779, EPI_ISL_692780, EPI_ISL_692782, EPI_ISL_692785	Massachusetts State Public Health Laboratory	Massachusetts State Public Health Laboratory	Andrew Lang, Timelia Fink, Glen Gallagher, Sandra Smole
EPI_ISL_693303	Department of Laboratory Medicine, National Taiwan University Hospital	Microbial Genomics Core Lab, National Taiwan University Centers of Genomic and Precision Medicine	Shiou-Hwei Yeh, You-Yu Lin, Ya-Yun Lai, Chiao-Ling Li, Shan-Chwen Chang, Pei-Jer Chen, Sui-Yuan Chang
EPI_ISL_693307, EPI_ISL_693308, EPI_ISL_693309, EPI_ISL_693310, EPI_ISL_693311, EPI_ISL_693312, EPI_ISL_693314, EPI_ISL_693317	National Public Health Laboratory, National Centre for Infectious Diseases	National Public Health Laboratory, National Centre for Infectious Diseases	Tze Minn Mak, Sophie Octavia, Zhenyang Zhou, Lin Cui, Raymond Tzer Pin Lin
EPI_ISL_693332, EPI_ISL_693333	Lighthouse Lab in Alderley Park	Wellcome Sanger Institute for the COVID-19 Genomics UK (COG-UK) Consortium	Jacquelyn Wynn, Mairead Hyland, The Lighthouse Lab in Alderley Park and Alex Alderton, Roberto Amato, Sonia Goncalves, Ewan Harrison, David K. Jackson, Ian Johnston, Dominic Kwiatkowski, Cordelia Langford, John Sillitoe on behalf of the Wellcome Sanger Institute COVID-19 Surveillance Team
EPI_ISL_693397, EPI_ISL_693404, EPI_ISL_693406, EPI_ISL_693407, EPI_ISL_693461, EPI_ISL_693462, EPI_ISL_693463, EPI_ISL_693464, EPI_ISL_693465, EPI_ISL_693466, EPI_ISL_693468			
see above	Respiratory Virus Unit, Microbiology Services Colindale, Public Health England	COVID-19 Genomics UK (COG-UK) Consortium	PHE Covid Sequencing Team
EPI_ISL_693770	General practitioner	National Reference Center for Viruses of Respiratory Infections, Institut Pasteur, Paris	Marion Barbet, Sylvie Behillil, Méline Bizard, Angela Brisebarre, Camille Capel, Etienne Simon-Lorière, Vincent Enouf, Maud Vanpeene, Sylvie van der Werf
EPI_ISL_693948, EPI_ISL_693949, EPI_ISL_693950, EPI_ISL_693951, EPI_ISL_693952, EPI_ISL_693953, EPI_ISL_693954, EPI_ISL_693955, EPI_ISL_693956, EPI_ISL_693957, EPI_ISL_693958, EPI_ISL_693959, EPI_ISL_693960, EPI_ISL_693961, EPI_ISL_693962, EPI_ISL_693963, EPI_ISL_693964, EPI_ISL_693965, EPI_ISL_693966, EPI_ISL_693967, EPI_ISL_693968, EPI_ISL_693969, EPI_ISL_693970, EPI_ISL_693971, EPI_ISL_693972, EPI_ISL_693973, EPI_ISL_693974, EPI_ISL_693975, EPI_ISL_693976, EPI_ISL_693977, EPI_ISL_693978, EPI_ISL_693979, EPI_ISL_693980, EPI_ISL_693981, EPI_ISL_693982, EPI_ISL_693983, EPI_ISL_693984, EPI_ISL_693985, EPI_ISL_693986, EPI_ISL_693987, EPI_ISL_693988, EPI_ISL_693989, EPI_ISL_693990, EPI_ISL_693991, EPI_ISL_693992, EPI_ISL_693993, EPI_ISL_693994, EPI_ISL_693995, EPI_ISL_693996, EPI_ISL_693997, EPI_ISL_693998, EPI_ISL_693999, EPI_ISL_694000, EPI_ISL_694001, EPI_ISL_694002, EPI_ISL_694003, EPI_ISL_694004, EPI_ISL_694005, EPI_ISL_694006, EPI_ISL_694007, EPI_ISL_694008			
see above	Viollier AG	Department of Biosystems Science and Engineering, ETH Zürich	Christian Beisel, Sarah Nadeau, Chaoran Chen, Ivan Topolsky, Pedro Ferreira, Philipp Jablonski, Susana Posada-Céspedes, Tobias Schär, Ina Nissen, Natascha Santacroce, Elodie Burcklen, Christiane Beckmann, Maurice Redondo, Olivier Kobel, Christoph Noppen, Sophie Seidel, Noemie Santamaria de Souza, Niko Beerenwinkel, Tanja Stadler
EPI_ISL_696452, EPI_ISL_696455	Thembalethu CDC wc THC & NHLS/UCT	KRISP, KZN Research Innovation and Sequencing Platform	Arash Iranzadeh, Deelan Doolabh, Lynn Tyers, Bruna Galvao, Innocent Mudau, Marvin Hsiao, Kruger Marais, Jennifer Giandhari, Sureshnee Pillay, Houriiyah Tegally, Emanuel James San, Tulio de Oliveira, Diana Hardie, Stephen Korsman, Carolyn Williamson
EPI_ISL_696457	Hornlee Clinic wc HLC & NHLS/UCT	KRISP, KZN Research Innovation and Sequencing Platform	Arash Iranzadeh, Deelan Doolabh, Lynn Tyers, Bruna Galvao, Innocent Mudau, Marvin Hsiao, Kruger Marais, Jennifer Giandhari, Sureshnee Pillay, Houriiyah Tegally, Emanuel James San, Tulio de Oliveira, Diana Hardie, Stephen Korsman, Carolyn Williamson
EPI_ISL_696461	Thembalethu CDC wc THC & NHLS/UCT	KRISP, KZN Research Innovation and Sequencing Platform	Arash Iranzadeh, Deelan Doolabh, Lynn Tyers, Bruna Galvao, Innocent Mudau, Marvin Hsiao, Kruger Marais, Jennifer Giandhari, Sureshnee Pillay, Houriiyah Tegally, Emanuel James San, Tulio de Oliveira, Diana Hardie, Stephen Korsman, Carolyn Williamson
EPI_ISL_696462	Sedgefield Clinic wc SGE & NHLS/UCT	KRISP, KZN Research Innovation and Sequencing Platform	Arash Iranzadeh, Deelan Doolabh, Lynn Tyers, Bruna Galvao, Innocent Mudau, Marvin Hsiao, Kruger Marais, Jennifer Giandhari, Sureshnee Pillay, Houriiyah Tegally, Emanuel James San, Tulio de Oliveira, Diana Hardie, Stephen Korsman, Carolyn Williamson
EPI_ISL_696463	Knysna Hospital wc KNY & NHLS/UCT	KRISP, KZN Research Innovation and Sequencing Platform	Arash Iranzadeh, Deelan Doolabh, Lynn Tyers, Bruna Galvao, Innocent Mudau, Marvin Hsiao, Kruger Marais, Jennifer Giandhari, Sureshnee Pillay, Houriiyah Tegally, Emanuel James San, Tulio de Oliveira, Diana Hardie, Stephen Korsman, Carolyn Williamson
EPI_ISL_696464	Groote Schuur Hospital wc GSH & NHLS/UCT	KRISP, KZN Research Innovation and Sequencing Platform	Arash Iranzadeh, Deelan Doolabh, Lynn Tyers, Bruna Galvao, Innocent Mudau, Marvin Hsiao, Kruger Marais, Jennifer Giandhari, Sureshnee Pillay, Houriiyah Tegally, Emanuel James San, Tulio de Oliveira, Diana Hardie, Stephen Korsman, Carolyn Williamson
EPI_ISL_696465	Kwanokuthula CDC wc KWA & NHLS/UCT	KRISP, KZN Research Innovation and Sequencing Platform	Arash Iranzadeh, Deelan Doolabh, Lynn Tyers, Bruna Galvao, Innocent Mudau, Marvin Hsiao, Kruger Marais, Jennifer Giandhari, Sureshnee Pillay, Houriiyah Tegally, Emanuel James San, Tulio de Oliveira, Diana Hardie, Stephen Korsman, Carolyn Williamson
EPI_ISL_696466	Great Brak River Clinic wc GBC & NHLS/UCT	KRISP, KZN Research Innovation and Sequencing Platform	Arash Iranzadeh, Deelan Doolabh, Lynn Tyers, Bruna Galvao, Innocent Mudau, Marvin Hsiao, Kruger Marais, Jennifer Giandhari, Sureshnee Pillay, Houriiyah Tegally, Emanuel James San, Tulio de Oliveira, Diana Hardie, Stephen Korsman, Carolyn Williamson
EPI_ISL_696467	Knysna CDC wc WLC & NHLS/UCT	KRISP, KZN Research Innovation and Sequencing Platform	Arash Iranzadeh, Deelan Doolabh, Lynn Tyers, Bruna Galvao, Innocent Mudau, Marvin Hsiao, Kruger Marais, Jennifer Giandhari, Sureshnee Pillay, Houriiyah Tegally, Emanuel James San, Tulio de Oliveira, Diana Hardie, Stephen Korsman, Carolyn Williamson
EPI_ISL_696468	New Horizon Clinic wc NZC & NHLS/UCT	KRISP, KZN Research Innovation and Sequencing Platform	Arash Iranzadeh, Deelan Doolabh, Lynn Tyers, Bruna Galvao, Innocent Mudau, Marvin Hsiao, Kruger Marais, Jennifer Giandhari, Sureshnee Pillay, Houriiyah Tegally, Emanuel James San, Tulio de Oliveira, Diana Hardie, Stephen Korsman, Carolyn Williamson
EPI_ISL_696469	Knysna Hospital wc KNY & NHLS/UCT	KRISP, KZN Research Innovation and Sequencing Platform	Arash Iranzadeh, Deelan Doolabh, Lynn Tyers, Bruna Galvao, Innocent Mudau, Marvin Hsiao, Kruger Marais, Jennifer Giandhari, Sureshnee Pillay, Houriiyah Tegally, Emanuel James San, Tulio de Oliveira, Diana Hardie, Stephen Korsman, Carolyn Williamson
EPI_ISL_696470	Pacaltsdorp Clinic wc PAC & NHLS/UCT	KRISP, KZN Research Innovation and Sequencing Platform	Arash Iranzadeh, Deelan Doolabh, Lynn Tyers, Bruna Galvao, Innocent Mudau, Marvin Hsiao, Kruger Marais, Jennifer Giandhari, Sureshnee Pillay, Houriiyah Tegally, Emanuel James San, Tulio de Oliveira, Diana Hardie, Stephen Korsman, Carolyn Williamson
EPI_ISL_696471	George Hospital wc GRH & NHLS/UCT	KRISP, KZN Research Innovation and Sequencing Platform	Arash Iranzadeh, Deelan Doolabh, Lynn Tyers, Bruna Galvao, Innocent Mudau, Marvin Hsiao, Kruger Marais, Jennifer Giandhari, Sureshnee Pillay, Houriiyah Tegally, Emanuel James San, Tulio de Oliveira, Diana Hardie, Stephen Korsman, Carolyn Williamson
EPI_ISL_696472	Conville CDC wc CVC & NHLS/UCT	KRISP, KZN Research Innovation and Sequencing Platform	Arash Iranzadeh, Deelan Doolabh, Lynn Tyers, Bruna Galvao, Innocent Mudau, Marvin Hsiao, Kruger Marais, Jennifer Giandhari, Sureshnee Pillay, Houriiyah Tegally, Emanuel James San, Tulio de Oliveira, Diana Hardie, Stephen Korsman, Carolyn Williamson
EPI_ISL_696473	George Hospital wc GRH & NHLS/UCT	KRISP, KZN Research Innovation and Sequencing Platform	Arash Iranzadeh, Deelan Doolabh, Lynn Tyers, Bruna Galvao, Innocent Mudau, Marvin Hsiao, Kruger Marais, Jennifer Giandhari, Sureshnee Pillay, Houriiyah Tegally, Emanuel James San, Tulio de Oliveira, Diana Hardie, Stephen Korsman, Carolyn Williamson
EPI_ISL_696474	Pacaltsdorp Clinic wc PAC & NHLS/UCT	KRISP, KZN Research Innovation and Sequencing Platform	Arash Iranzadeh, Deelan Doolabh, Lynn Tyers, Bruna Galvao, Innocent Mudau, Marvin Hsiao, Kruger Marais, Jennifer Giandhari, Sureshnee Pillay, Houriiyah Tegally, Emanuel James San, Tulio de Oliveira, Diana Hardie, Stephen Korsman, Carolyn Williamson
EPI_ISL_696475	Sedgefield Clinic wc SGE & NHLS/UCT	KRISP, KZN Research Innovation and Sequencing Platform	Arash Iranzadeh, Deelan Doolabh, Lynn Tyers, Bruna Galvao, Innocent Mudau, Marvin Hsiao, Kruger Marais, Jennifer Giandhari, Sureshnee Pillay, Houriiyah Tegally, Emanuel James San, Tulio de Oliveira, Diana Hardie, Stephen Korsman, Carolyn Williamson
EPI_ISL_696476	Plettenberg Bay Clinic wc PLC & NHLS/UCT	KRISP, KZN Research Innovation and Sequencing Platform	Arash Iranzadeh, Deelan Doolabh, Lynn Tyers, Bruna Galvao, Innocent Mudau, Marvin Hsiao, Kruger Marais, Jennifer Giandhari, Sureshnee Pillay, Houriiyah Tegally, Emanuel James San, Tulio de Oliveira, Diana Hardie, Stephen Korsman, Carolyn Williamson
EPI_ISL_696477, EPI_ISL_696478	Knysna Hospital wc KNY & NHLS/UCT	KRISP, KZN Research Innovation and Sequencing Platform	Arash Iranzadeh, Deelan Doolabh, Lynn Tyers, Bruna Galvao, Innocent Mudau, Marvin Hsiao, Kruger Marais, Jennifer Giandhari, Sureshnee Pillay, Houriiyah Tegally, Emanuel James San, Tulio de Oliveira, Diana Hardie, Stephen Korsman, Carolyn Williamson

EPI_ISL_696480	Crags Clinic wc CRG & NHLS/UCT	KRISP, KZN Research Innovation and Sequencing Platform	Arash Iranzadeh, Deelan Doolabh, Lynn Tyers, Bruna Galvao, Innocent Mudau, Marvin Hsiao, Kruger Marais, Jennifer Giandhari, Sureshnee Pillay, Houriiyah Tegally, Emanuel James San, Tulio de Oliveira, Diana Hardie, Stephen Korsman, Carolyn Williamson
EPI_ISL_696481	George Hospital wc GRH & NHLS/UCT	KRISP, KZN Research Innovation and Sequencing Platform	Arash Iranzadeh, Deelan Doolabh, Lynn Tyers, Bruna Galvao, Innocent Mudau, Marvin Hsiao, Kruger Marais, Jennifer Giandhari, Sureshnee Pillay, Houriiyah Tegally, Emanuel James San, Tulio de Oliveira, Diana Hardie, Stephen Korsman, Carolyn Williamson
EPI_ISL_696485	Touwsranten Clinic wc TST & NHLS/UCT	KRISP, KZN Research Innovation and Sequencing Platform	Arash Iranzadeh, Deelan Doolabh, Lynn Tyers, Bruna Galvao, Innocent Mudau, Marvin Hsiao, Kruger Marais, Jennifer Giandhari, Sureshnee Pillay, Houriiyah Tegally, Emanuel James San, Tulio de Oliveira, Diana Hardie, Stephen Korsman, Carolyn Williamson
EPI_ISL_696491	Knysna Hospital wc KNY & NHLS/UCT	KRISP, KZN Research Innovation and Sequencing Platform	Arash Iranzadeh, Deelan Doolabh, Lynn Tyers, Bruna Galvao, Innocent Mudau, Marvin Hsiao, Kruger Marais, Jennifer Giandhari, Sureshnee Pillay, Houriiyah Tegally, Emanuel James San, Tulio de Oliveira, Diana Hardie, Stephen Korsman, Carolyn Williamson
EPI_ISL_701675, EPI_ISL_701681, EPI_ISL_701683, EPI_ISL_701697, EPI_ISL_701698, EPI_ISL_701701, EPI_ISL_701713, EPI_ISL_701714, EPI_ISL_701722, EPI_ISL_701726, EPI_ISL_701729, EPI_ISL_701737, EPI_ISL_701739, EPI_ISL_701743, EPI_ISL_701746, EPI_ISL_701749, EPI_ISL_701754, EPI_ISL_701759, EPI_ISL_701762, EPI_ISL_701774, EPI_ISL_701775, EPI_ISL_701781, EPI_ISL_701788, EPI_ISL_701792, EPI_ISL_701795, EPI_ISL_701800, EPI_ISL_701803, EPI_ISL_701819, EPI_ISL_701829, EPI_ISL_701837, EPI_ISL_701838, EPI_ISL_701839, EPI_ISL_701841, EPI_ISL_701843, EPI_ISL_701847, EPI_ISL_701848, EPI_ISL_701850, EPI_ISL_701853, EPI_ISL_701870, EPI_ISL_701871, EPI_ISL_701875, EPI_ISL_701876, EPI_ISL_701878, EPI_ISL_701881, EPI_ISL_701913, EPI_ISL_701914, EPI_ISL_701916, EPI_ISL_701920, EPI_ISL_701923, EPI_ISL_701927, EPI_ISL_701930, EPI_ISL_701932, EPI_ISL_701943, EPI_ISL_701947, EPI_ISL_701963, EPI_ISL_701975, EPI_ISL_701976, EPI_ISL_701980, EPI_ISL_701987, EPI_ISL_701993, EPI_ISL_701994, EPI_ISL_701999, EPI_ISL_702001, EPI_ISL_702002, EPI_ISL_702014, EPI_ISL_702015, EPI_ISL_702016, EPI_ISL_702376, EPI_ISL_702381, EPI_ISL_702384, EPI_ISL_702413, EPI_ISL_702423, EPI_ISL_702424, EPI_ISL_702427, EPI_ISL_702443, EPI_ISL_702447, EPI_ISL_702453, EPI_ISL_702486			
see above	Lighthouse Lab in Alderley Park	Wellcome Sanger Institute for the COVID-19 Genomics UK (COG-UK) Consortium	Jacquelyn Wynn, Mairead Hyland, The Lighthouse Lab in Alderley Park and Alex Alderton, Roberto Amato, Sonia Goncalves, Ewan Harrison, David K. Jackson, Ian Johnston, Dominic Kwiatkowski, Cordelia Langford, John Sillitoe on behalf of the Wellcome Sanger Institute COVID-19 Surveillance Team
EPI_ISL_702495	Northumbria University / South Tees Hospitals NHS Foundation Trust / North Cumbria Integrated Care NHS Foundation Trust / North Tees and Hartlepool NHS Foundation Trust / Newcastle Hospitals NHS Foundation Trust	COVID-19 Genomics UK (COG-UK) Consortium	Darren L Smith,Andrew Nelson,Matthew Bashton,Greg R Young,Joshua Loh,John Allan,Mohammad A Tariq,Giles S Holt,Gary Black,Wen C Yew,Lynn Dover,Paul Baker,Steve Liggett,Sarah Essex,Jane Greenaway,Debra Padgett,Clive Graham,Garren Scott,Edward Barton,Emma Swindells,Brendan Payne,Jennifer Collins,Yusri Taha,Gary Eltringham
EPI_ISL_702519, EPI_ISL_702521, EPI_ISL_702537	Lighthouse Lab in Alderley Park	Wellcome Sanger Institute for the COVID-19 Genomics UK (COG-UK) Consortium	Jacquelyn Wynn, Mairead Hyland, The Lighthouse Lab in Alderley Park and Alex Alderton, Roberto Amato, Sonia Goncalves, Ewan Harrison, David K. Jackson, Ian Johnston, Dominic Kwiatkowski, Cordelia Langford, John Sillitoe on behalf of the Wellcome Sanger Institute COVID-19 Surveillance Team
EPI_ISL_702539	Northumbria University / South Tees Hospitals NHS Foundation Trust / North Cumbria Integrated Care NHS Foundation Trust / North Tees and Hartlepool NHS Foundation Trust / Newcastle Hospitals NHS Foundation Trust	COVID-19 Genomics UK (COG-UK) Consortium	Darren L Smith,Andrew Nelson,Matthew Bashton,Greg R Young,Joshua Loh,John Allan,Mohammad A Tariq,Giles S Holt,Gary Black,Wen C Yew,Lynn Dover,Paul Baker,Steve Liggett,Sarah Essex,Jane Greenaway,Debra Padgett,Clive Graham,Garren Scott,Edward Barton,Emma Swindells,Brendan Payne,Jennifer Collins,Yusri Taha,Gary Eltringham
EPI_ISL_702543	Lighthouse Lab in Alderley Park	Wellcome Sanger Institute for the COVID-19 Genomics UK (COG-UK) Consortium	Jacquelyn Wynn, Mairead Hyland, The Lighthouse Lab in Alderley Park and Alex Alderton, Roberto Amato, Sonia Goncalves, Ewan Harrison, David K. Jackson, Ian Johnston, Dominic Kwiatkowski, Cordelia Langford, John Sillitoe on behalf of the Wellcome Sanger Institute COVID-19 Surveillance Team
EPI_ISL_702544	University College London, Great Ormond Street Hospital for Children NHS Foundation Trust, Imperial College Healthcare NHS Trust	COVID-19 Genomics UK (COG-UK) Consortium	Sergi Castellano, Rachel Williams, Mark Kristiansen, Paola Resende Silva, Sunando Roy, Tony Brooks, Helena Tutill, Paola Niola, Patricia Dyal, Charlotte Williams, Leysa Forrest, Yasmin Panchbhaya, Jacqueline Findlay, Samuel Weeks, Julianne Brown, Kathryn Harris, Paul Randell, James Price, Alison Holmes, Judith Breuer
EPI_ISL_702547	Northumbria University / South Tees Hospitals NHS Foundation Trust / North Cumbria Integrated Care NHS Foundation Trust / North Tees and Hartlepool NHS Foundation Trust / Newcastle Hospitals NHS Foundation Trust	COVID-19 Genomics UK (COG-UK) Consortium	Darren L Smith,Andrew Nelson,Matthew Bashton,Greg R Young,Joshua Loh,John Allan,Mohammad A Tariq,Giles S Holt,Gary Black,Wen C Yew,Lynn Dover,Paul Baker,Steve Liggett,Sarah Essex,Jane Greenaway,Debra Padgett,Clive Graham,Garren Scott,Edward Barton,Emma Swindells,Brendan Payne,Jennifer Collins,Yusri Taha,Gary Eltringham
EPI_ISL_702553	Wales Specialist Virology Centre Sequencing lab: Pathogen Genomics Unit	COVID-19 Genomics UK (COG-UK) Consortium	Catherine Moore, Johnathan Evans, Laura Gifford, Malorie Perry, Simon Cottrell, Angela Marchbank, Alec Birchley, Alexander Adams, Amy Gaskin, Bree Gatica-Wilcox, Jason Coombes, Joel Southgate, Lauren Gilbert, Lee Graham, Nicole Pacchiarini, Sara Kumziene-Summerhayes, Sarah Taylor, Sophie Jones, Sara Rey, Matthew Bull, Joanne Watkins, Sally Corden, Tom Connor
EPI_ISL_702571, EPI_ISL_702591	Liverpool Clinical Laboratories	COVID-19 Genomics UK (COG-UK) Consortium	Sam Haldenby, Anita Lucaci, Steve Paterson, Julian Hiscox, Alistair Darby, M Almsaud, A Alrezaihi, Muhannad Alruwaili, Stuart D Armstrong, Jones Benjamin, Eleanor G Bentley, Anu Chawla, Jordan J Clark, Angela Cowell, Richard Eccles, Isabel García-Dorival, Matthew Gemmell, Alessandro Gerada, PKF Gilmore, Richard Gregory, Ximeng Han, Catherine Hartley, Margaret Hughes, Miren Iturriza-Gomara, James Johnson, L Luu, Jenifer Manson, Charlotte Nelson, Elaine O'Toole, Cassie Olateju, Rebekah Penrice-Randal , Lucille Rainbow, N.P Randle, Trevor Ian Robinson, Parul Sharma, Ghada T Shawli, James P Stewart, Neil Swainston, Ecaterina Vamos, Joanne Watts, Mark Whitehead
EPI_ISL_702610	University College London, Great Ormond Street Hospital for Children NHS Foundation Trust, Imperial College Healthcare NHS Trust	COVID-19 Genomics UK (COG-UK) Consortium	Sergi Castellano, Rachel Williams, Mark Kristiansen, Paola Resende Silva, Sunando Roy, Tony Brooks, Helena Tutill, Paola Niola, Patricia Dyal, Charlotte Williams, Leysa Forrest, Yasmin Panchbhaya, Jacqueline Findlay, Samuel Weeks, Julianne Brown, Kathryn Harris, Paul Randell, James Price, Alison Holmes, Judith Breuer
EPI_ISL_702615	Liverpool Clinical Laboratories	COVID-19 Genomics UK (COG-UK) Consortium	Sam Haldenby, Anita Lucaci, Steve Paterson, Julian Hiscox, Alistair Darby, M Almsaud, A Alrezaihi, Muhannad Alruwaili, Stuart D Armstrong, Jones Benjamin, Eleanor G Bentley, Anu Chawla, Jordan J Clark, Angela Cowell, Richard Eccles, Isabel García-Dorival, Matthew Gemmell, Alessandro Gerada, PKF Gilmore, Richard Gregory, Ximeng Han, Catherine Hartley, Margaret Hughes, Miren Iturriza-Gomara, James Johnson, L Luu, Jenifer Manson, Charlotte Nelson, Elaine O'Toole, Cassie Olateju, Rebekah Penrice-Randal , Lucille Rainbow, N.P Randle, Trevor Ian Robinson, Parul Sharma, Ghada T Shawli, James P Stewart, Neil Swainston, Ecaterina Vamos, Joanne Watts, Mark Whitehead
EPI_ISL_702619, EPI_ISL_702622	Lighthouse Lab in Alderley Park	Wellcome Sanger Institute for the COVID-19 Genomics UK (COG-UK) Consortium	Jacquelyn Wynn, Mairead Hyland, The Lighthouse Lab in Alderley Park and Alex Alderton, Roberto Amato, Sonia Goncalves, Ewan Harrison, David K. Jackson, Ian Johnston, Dominic Kwiatkowski, Cordelia Langford, John Sillitoe on behalf of the Wellcome Sanger Institute COVID-19 Surveillance Team
EPI_ISL_702623, EPI_ISL_702628, EPI_ISL_702631	Liverpool Clinical Laboratories	COVID-19 Genomics UK (COG-UK) Consortium	Sam Haldenby, Anita Lucaci, Steve Paterson, Julian Hiscox, Alistair Darby, M Almsaud, A Alrezaihi, Muhannad Alruwaili, Stuart D Armstrong, Jones Benjamin, Eleanor G Bentley, Anu Chawla, Jordan J Clark, Angela Cowell, Richard Eccles, Isabel García-Dorival, Matthew Gemmell, Alessandro Gerada, PKF Gilmore, Richard Gregory, Ximeng Han, Catherine Hartley, Margaret Hughes, Miren Iturriza-Gomara, James Johnson, L Luu, Jenifer Manson, Charlotte Nelson, Elaine O'Toole, Cassie Olateju, Rebekah Penrice-Randal , Lucille Rainbow, N.P Randle, Trevor Ian Robinson, Parul Sharma, Ghada T Shawli, James P Stewart, Neil Swainston, Ecaterina Vamos, Joanne Watts, Mark Whitehead
EPI_ISL_702634, EPI_ISL_702635, EPI_ISL_702637, EPI_ISL_702638, EPI_ISL_702640, EPI_ISL_702658	Lighthouse Lab in Alderley Park	Wellcome Sanger Institute for the COVID-19 Genomics UK (COG-UK) Consortium	Jacquelyn Wynn, Mairead Hyland, The Lighthouse Lab in Alderley Park and Alex Alderton, Roberto Amato, Sonia Goncalves, Ewan Harrison, David K. Jackson, Ian Johnston, Dominic Kwiatkowski, Cordelia Langford, John Sillitoe on behalf of the Wellcome Sanger Institute COVID-19 Surveillance Team
EPI_ISL_702659	West of Scotland Specialist Virology Centre, NHSGGC / MRC-University of Glasgow Centre for Virus Research	COVID-19 Genomics UK (COG-UK) Consortium	Ana da Silva Filipe, Natasha Johnson, Kathy Smollett, Daniel Mair, Stephen Carmichael, Alice Broos, Lily Tong, Jenna Nichols, Kyriaki Nomikou; Sarah McDonald; Richard Orton, Joseph Hughes, Sreenu Vattipally, David L Robertson; Alasdair MacLean, Rory Gunson; Sharif Shaaban, Matthew Holden; Rachel Blacow, Guy Mollett, Kathy Li, James Shepherd, Antonia Ho, Emma Thomson
EPI_ISL_702663, EPI_ISL_702668, EPI_ISL_702678, EPI_ISL_702681, EPI_ISL_702683, EPI_ISL_702701, EPI_ISL_702704, EPI_ISL_702707, EPI_ISL_702711, EPI_ISL_702712	Lighthouse Lab in Alderley Park	Wellcome Sanger Institute for the COVID-19 Genomics UK (COG-UK) Consortium	Jacquelyn Wynn, Mairead Hyland, The Lighthouse Lab in Alderley Park and Alex Alderton, Roberto Amato, Sonia Goncalves, Ewan Harrison, David K. Jackson, Ian Johnston, Dominic Kwiatkowski, Cordelia Langford, John Sillitoe on behalf of the Wellcome Sanger Institute COVID-19 Surveillance Team
EPI_ISL_702713	Wales Specialist Virology Centre Sequencing lab: Pathogen Genomics Unit	COVID-19 Genomics UK (COG-UK) Consortium	Catherine Moore, Johnathan Evans, Laura Gifford, Malorie Perry, Simon Cottrell, Angela Marchbank, Alec Birchley, Alexander Adams, Amy Gaskin, Bree Gatica-Wilcox, Jason Coombes, Joel Southgate, Lauren Gilbert, Lee Graham, Nicole Pacchiarini, Sara Kumziene-Summerhayes, Sarah Taylor, Sophie Jones, Sara Rey, Matthew Bull, Joanne Watkins, Sally Corden, Tom Connor
EPI_ISL_702719, EPI_ISL_702720, EPI_ISL_702725	Lighthouse Lab in Alderley Park	Wellcome Sanger Institute for the COVID-19 Genomics UK (COG-UK) Consortium	Jacquelyn Wynn, Mairead Hyland, The Lighthouse Lab in Alderley Park and Alex Alderton, Roberto Amato, Sonia Goncalves, Ewan Harrison, David K. Jackson, Ian Johnston, Dominic Kwiatkowski, Cordelia Langford, John Sillitoe on behalf of the Wellcome Sanger Institute COVID-19 Surveillance Team
EPI_ISL_702729	Liverpool Clinical Laboratories	COVID-19 Genomics UK (COG-UK) Consortium	Sam Haldenby, Anita Lucaci, Steve Paterson, Julian Hiscox, Alistair Darby, M Almsaud, A Alrezaihi, Muhannad Alruwaili, Stuart D Armstrong, Jones Benjamin, Eleanor G Bentley, Anu Chawla, Jordan J Clark, Angela Cowell, Richard Eccles, Isabel García-Dorival, Matthew Gemmell, Alessandro Gerada,

			PKF Gilmore, Richard Gregory, Ximeng Han, Catherine Hartley, Margaret Hughes, Miren Iturriza-Gomara, James Johnson, L Luu, Jenifer Manson, Charlotte Nelson, Elaine O'Toole, Cassie Olateju, Rebekah Penrice-Randal , Lucille Rainbow, N.P Randle, Trevor Ian Robinson, Parul Sharma, Ghada T Shawli, James P Stewart, Neil Swainston, Ecaterina Vamos, Joanne Watts, Mark Whitehead
EPI_ISL_702731	University College London, Great Ormond Street Hospital for Children NHS Foundation Trust, Imperial College Healthcare NHS Trust	COVID-19 Genomics UK (COG-UK) Consortium	Sergi Castellano, Rachel Williams, Mark Kristiansen, Paola Resende Silva, Sunando Roy, Tony Brooks, Helena Tutill, Paola Niola, Patricia Dyal, Charlotte Williams, Leysa Forrest, Yasmin Panchbhaya, Jacqueline Findlay, Samuel Weeks, Julianne Brown, Kathryn Harris, Paul Randell, James Price, Alison Holmes, Judith Breuer
EPI_ISL_702732, EPI_ISL_702733, EPI_ISL_702741	Lighthouse Lab in Alderley Park	Wellcome Sanger Institute for the COVID-19 Genomics UK (COG-UK) Consortium	Jacquelyn Wynn, Mairead Hyland, The Lighthouse Lab in Alderley Park and Alex Alderton, Roberto Amato, Sonia Goncalves, Ewan Harrison, David K. Jackson, Ian Johnston, Dominic Kwiatkowski, Cordelia Langford, John Sillitoe on behalf of the Wellcome Sanger Institute COVID-19 Surveillance Team
EPI_ISL_702742	Wales Specialist Virology Centre Sequencing lab: Pathogen Genomics Unit	COVID-19 Genomics UK (COG-UK) Consortium	Catherine Moore, Johnathan Evans, Laura Gifford, Malorie Perry, Simon Cottrell, Angela Marchbank, Alec Birchley, Alexander Adams, Amy Gaskin, Bree Gatica-Wilcox, Jason Coombes, Joel Southgate, Lauren Gilbert, Lee Graham, Nicole Pacchiarini, Sara Kumziene-Summerhayes, Sarah Taylor, Sophie Jones, Sara Rey, Matthew Bull, Joanne Watkins, Sally Corden, Tom Connor
EPI_ISL_702745, EPI_ISL_702752, EPI_ISL_702754, EPI_ISL_702755	Lighthouse Lab in Alderley Park	Wellcome Sanger Institute for the COVID-19 Genomics UK (COG-UK) Consortium	Jacquelyn Wynn, Mairead Hyland, The Lighthouse Lab in Alderley Park and Alex Alderton, Roberto Amato, Sonia Goncalves, Ewan Harrison, David K. Jackson, Ian Johnston, Dominic Kwiatkowski, Cordelia Langford, John Sillitoe on behalf of the Wellcome Sanger Institute COVID-19 Surveillance Team
EPI_ISL_702769	Liverpool Clinical Laboratories	COVID-19 Genomics UK (COG-UK) Consortium	Sam Haldenby, Anita Lucaci, Steve Paterson, Julian Hiscox, Alistair Darby, M Almsaud, A Alrezaihi, Muhannad Alruwaili, Stuart D Armstrong, Jones Benjamin, Eleanor G Bentley, Anu Chawla, Jordan J Clark, Angela Cowell, Richard Eccles, Isabel García-Dorival, Matthew Gemmell, Alessandro Gerada, PKF Gilmore, Richard Gregory, Ximeng Han, Catherine Hartley, Margaret Hughes, Miren Iturriza-Gomara, James Johnson, L Luu, Jenifer Manson, Charlotte Nelson, Elaine O'Toole, Cassie Olateju, Rebekah Penrice-Randal , Lucille Rainbow, N.P Randle, Trevor Ian Robinson, Parul Sharma, Ghada T Shawli, James P Stewart, Neil Swainston, Ecaterina Vamos, Joanne Watts, Mark Whitehead
EPI_ISL_702773, EPI_ISL_702785, EPI_ISL_702791, EPI_ISL_702796, EPI_ISL_702797, EPI_ISL_702800, EPI_ISL_702804	Lighthouse Lab in Alderley Park	Wellcome Sanger Institute for the COVID-19 Genomics UK (COG-UK) Consortium	Jacquelyn Wynn, Mairead Hyland, The Lighthouse Lab in Alderley Park and Alex Alderton, Roberto Amato, Sonia Goncalves, Ewan Harrison, David K. Jackson, Ian Johnston, Dominic Kwiatkowski, Cordelia Langford, John Sillitoe on behalf of the Wellcome Sanger Institute COVID-19 Surveillance Team
EPI_ISL_702812, EPI_ISL_702815	Northumbria University / South Tees Hospitals NHS Foundation Trust / North Cumbria Integrated Care NHS Foundation Trust / North Tees and Hartlepool NHS Foundation Trust / Newcastle Hospitals NHS Foundation Trust	COVID-19 Genomics UK (COG-UK) Consortium	Darren L Smith,Andrew Nelson,Matthew Bashton,Greg R Young,Joshua Loh,John Allan,Mohammad A Tariq,Giles S Holt,Gary Black,Wen C Yew,Lynn Dover,Paul Baker,Steve Liggett,Sarah Essex,Jane Greenaway,Debra Padgett,Clive Graham,Garren Scott,Edward Barton,Emma Swindells,Brendan Payne,Jennifer Collins,Yusri Taha,Gary Eltringham
EPI_ISL_702816	Lighthouse Lab in Alderley Park	Wellcome Sanger Institute for the COVID-19 Genomics UK (COG-UK) Consortium	Jacquelyn Wynn, Mairead Hyland, The Lighthouse Lab in Alderley Park and Alex Alderton, Roberto Amato, Sonia Goncalves, Ewan Harrison, David K. Jackson, Ian Johnston, Dominic Kwiatkowski, Cordelia Langford, John Sillitoe on behalf of the Wellcome Sanger Institute COVID-19 Surveillance Team
EPI_ISL_702817	Wales Specialist Virology Centre Sequencing lab: Pathogen Genomics Unit	COVID-19 Genomics UK (COG-UK) Consortium	Catherine Moore, Johnathan Evans, Laura Gifford, Malorie Perry, Simon Cottrell, Angela Marchbank, Alec Birchley, Alexander Adams, Amy Gaskin, Bree Gatica-Wilcox, Jason Coombes, Joel Southgate, Lauren Gilbert, Lee Graham, Nicole Pacchiarini, Sara Kumziene-Summerhayes, Sarah Taylor, Sophie Jones, Sara Rey, Matthew Bull, Joanne Watkins, Sally Corden, Tom Connor
EPI_ISL_702821	Lighthouse Lab in Alderley Park	Wellcome Sanger Institute for the COVID-19 Genomics UK (COG-UK) Consortium	Jacquelyn Wynn, Mairead Hyland, The Lighthouse Lab in Alderley Park and Alex Alderton, Roberto Amato, Sonia Goncalves, Ewan Harrison, David K. Jackson, Ian Johnston, Dominic Kwiatkowski, Cordelia Langford, John Sillitoe on behalf of the Wellcome Sanger Institute COVID-19 Surveillance Team
EPI_ISL_702823, EPI_ISL_702827	Northumbria University / South Tees Hospitals NHS Foundation Trust / North Cumbria Integrated Care NHS Foundation Trust / North Tees and Hartlepool NHS Foundation Trust / Newcastle Hospitals NHS Foundation Trust	COVID-19 Genomics UK (COG-UK) Consortium	Darren L Smith,Andrew Nelson,Matthew Bashton,Greg R Young,Joshua Loh,John Allan,Mohammad A Tariq,Giles S Holt,Gary Black,Wen C Yew,Lynn Dover,Paul Baker,Steve Liggett,Sarah Essex,Jane Greenaway,Debra Padgett,Clive Graham,Garren Scott,Edward Barton,Emma Swindells,Brendan Payne,Jennifer Collins,Yusri Taha,Gary Eltringham
EPI_ISL_702838	Liverpool Clinical Laboratories	COVID-19 Genomics UK (COG-UK) Consortium	Sam Haldenby, Anita Lucaci, Steve Paterson, Julian Hiscox, Alistair Darby, M Almsaud, A Alrezaihi, Muhannad Alruwaili, Stuart D Armstrong, Jones Benjamin, Eleanor G Bentley, Anu Chawla, Jordan J Clark, Angela Cowell, Richard Eccles, Isabel García-Dorival, Matthew Gemmell, Alessandro Gerada, PKF Gilmore, Richard Gregory, Ximeng Han, Catherine Hartley, Margaret Hughes, Miren Iturriza-Gomara, James Johnson, L Luu, Jenifer Manson, Charlotte Nelson, Elaine O'Toole, Cassie Olateju, Rebekah Penrice-Randal , Lucille Rainbow, N.P Randle, Trevor Ian Robinson, Parul Sharma, Ghada T Shawli, James P Stewart, Neil Swainston, Ecaterina Vamos, Joanne Watts, Mark Whitehead
EPI_ISL_702857, EPI_ISL_702859	Wales Specialist Virology Centre Sequencing lab: Pathogen Genomics Unit	COVID-19 Genomics UK (COG-UK) Consortium	Catherine Moore, Johnathan Evans, Laura Gifford, Malorie Perry, Simon Cottrell, Angela Marchbank, Alec Birchley, Alexander Adams, Amy Gaskin, Bree Gatica-Wilcox, Jason Coombes, Joel Southgate, Lauren Gilbert, Lee Graham, Nicole Pacchiarini, Sara Kumziene-Summerhayes, Sarah Taylor, Sophie Jones, Sara Rey, Matthew Bull, Joanne Watkins, Sally Corden, Tom Connor
EPI_ISL_702867	West of Scotland Specialist Virology Centre, NHSGGC / MRC-University of Glasgow Centre for Virus Research	COVID-19 Genomics UK (COG-UK) Consortium	Ana da Silva Filipe, Natasha Johnson, Kathy Smollett, Daniel Mair, Stephen Carmichael, Alice Broos, Lily Tong, Jenna Nichols, Kyriaki Nomikou; Sarah McDonald; Richard Orton, Joseph Hughes, Sreenu Vattipally, David L Robertson; Alasdair MacLean, Rory Gunson; Sharif Shaaban, Matthew Holden; Rachel Blacow, Guy Mollett, Kathy Li, James Shepherd, Antonia Ho, Emma Thomson
EPI_ISL_702895, EPI_ISL_702905	Wales Specialist Virology Centre Sequencing lab: Pathogen Genomics Unit	COVID-19 Genomics UK (COG-UK) Consortium	Catherine Moore, Johnathan Evans, Laura Gifford, Malorie Perry, Simon Cottrell, Angela Marchbank, Alec Birchley, Alexander Adams, Amy Gaskin, Bree Gatica-Wilcox, Jason Coombes, Joel Southgate, Lauren Gilbert, Lee Graham, Nicole Pacchiarini, Sara Kumziene-Summerhayes, Sarah Taylor, Sophie Jones, Sara Rey, Matthew Bull, Joanne Watkins, Sally Corden, Tom Connor
EPI_ISL_702906, EPI_ISL_702908, EPI_ISL_702916, EPI_ISL_702918, EPI_ISL_702923, EPI_ISL_702927, EPI_ISL_702929	Lighthouse Lab in Alderley Park	Wellcome Sanger Institute for the COVID-19 Genomics UK (COG-UK) Consortium	Jacquelyn Wynn, Mairead Hyland, The Lighthouse Lab in Alderley Park and Alex Alderton, Roberto Amato, Sonia Goncalves, Ewan Harrison, David K. Jackson, Ian Johnston, Dominic Kwiatkowski, Cordelia Langford, John Sillitoe on behalf of the Wellcome Sanger Institute COVID-19 Surveillance Team
EPI_ISL_702930	University College London, Great Ormond Street Hospital for Children NHS Foundation Trust, Imperial College Healthcare NHS Trust	COVID-19 Genomics UK (COG-UK) Consortium	Sergi Castellano, Rachel Williams, Mark Kristiansen, Paola Resende Silva, Sunando Roy, Tony Brooks, Helena Tutill, Paola Niola, Patricia Dyal, Charlotte Williams, Leysa Forrest, Yasmin Panchbhaya, Jacqueline Findlay, Samuel Weeks, Julianne Brown, Kathryn Harris, Paul Randell, James Price, Alison Holmes, Judith Breuer
EPI_ISL_702940, EPI_ISL_702942	Lighthouse Lab in Alderley Park	Wellcome Sanger Institute for the COVID-19 Genomics UK (COG-UK) Consortium	Jacquelyn Wynn, Mairead Hyland, The Lighthouse Lab in Alderley Park and Alex Alderton, Roberto Amato, Sonia Goncalves, Ewan Harrison, David K. Jackson, Ian Johnston, Dominic Kwiatkowski, Cordelia Langford, John Sillitoe on behalf of the Wellcome Sanger Institute COVID-19 Surveillance Team
EPI_ISL_702947	Liverpool Clinical Laboratories	COVID-19 Genomics UK (COG-UK) Consortium	Sam Haldenby, Anita Lucaci, Steve Paterson, Julian Hiscox, Alistair Darby, M Almsaud, A Alrezaihi, Muhannad Alruwaili, Stuart D Armstrong, Jones Benjamin, Eleanor G Bentley, Anu Chawla, Jordan J Clark, Angela Cowell, Richard Eccles, Isabel García-Dorival, Matthew Gemmell, Alessandro Gerada, PKF Gilmore, Richard Gregory, Ximeng Han, Catherine Hartley, Margaret Hughes, Miren Iturriza-Gomara, James Johnson, L Luu, Jenifer Manson, Charlotte Nelson, Elaine O'Toole, Cassie Olateju, Rebekah Penrice-Randal , Lucille Rainbow, N.P Randle, Trevor Ian Robinson, Parul Sharma, Ghada T Shawli, James P Stewart, Neil Swainston, Ecaterina Vamos, Joanne Watts, Mark Whitehead
EPI_ISL_702952, EPI_ISL_702954	Lighthouse Lab in Alderley Park	Wellcome Sanger Institute for the COVID-19 Genomics UK (COG-UK) Consortium	Jacquelyn Wynn, Mairead Hyland, The Lighthouse Lab in Alderley Park and Alex Alderton, Roberto Amato, Sonia Goncalves, Ewan Harrison, David K. Jackson, Ian Johnston, Dominic Kwiatkowski, Cordelia Langford, John Sillitoe on behalf of the Wellcome Sanger Institute COVID-19 Surveillance Team
EPI_ISL_702959	Northumbria University / South Tees Hospitals NHS Foundation Trust / North Cumbria Integrated Care NHS Foundation Trust / North Tees and Hartlepool NHS Foundation Trust / Newcastle Hospitals NHS Foundation Trust	COVID-19 Genomics UK (COG-UK) Consortium	Darren L Smith,Andrew Nelson,Matthew Bashton,Greg R Young,Joshua Loh,John Allan,Mohammad A Tariq,Giles S Holt,Gary Black,Wen C Yew,Lynn Dover,Paul Baker,Steve Liggett,Sarah Essex,Jane Greenaway,Debra Padgett,Clive Graham,Garren Scott,Edward Barton,Emma Swindells,Brendan Payne,Jennifer Collins,Yusri Taha,Gary Eltringham
EPI_ISL_702963, EPI_ISL_702965, EPI_ISL_702967, EPI_ISL_702968, EPI_ISL_702970, EPI_ISL_702972, EPI_ISL_702976, EPI_ISL_702979	Lighthouse Lab in Alderley Park	Wellcome Sanger Institute for the COVID-19 Genomics UK (COG-UK) Consortium	Jacquelyn Wynn, Mairead Hyland, The Lighthouse Lab in Alderley Park and Alex Alderton, Roberto Amato, Sonia Goncalves, Ewan Harrison, David K. Jackson, Ian Johnston, Dominic Kwiatkowski, Cordelia Langford, John Sillitoe on behalf of the Wellcome Sanger Institute COVID-19 Surveillance Team
EPI_ISL_702980	Liverpool Clinical Laboratories	COVID-19 Genomics UK (COG-UK) Consortium	Sam Haldenby, Anita Lucaci, Steve Paterson, Julian Hiscox, Alistair Darby, M Almsaud, A Alrezaihi, Muhannad Alruwaili, Stuart D Armstrong, Jones

			Benjamin, Eleanor G Bentley, Anu Chawla, Jordan J Clark, Angela Cowell, Richard Eccles, Isabel García-Dorival, Matthew Gemmell, Alessandro Gerada, PKF Gilmore, Richard Gregory, Ximeng Han, Catherine Hartley, Margaret Hughes, Miren Iturriza-Gomara, James Johnson, L Luu, Jenifer Manson, Charlotte Nelson, Elaine O'Toole, Cassie Olateju, Rebekah Penrice-Randal , Lucille Rainbow, N.P Randle, Trevor Ian Robinson, Parul Sharma, Ghada T Shawli, James P Stewart, Neil Swainston, Ecaterina Vamos, Joanne Watts, Mark Whitehead
EPI_ISL_702981, EPI_ISL_702983, EPI_ISL_702986, EPI_ISL_702988, EPI_ISL_702991, EPI_ISL_702993	Lighthouse Lab in Alderley Park	Wellcome Sanger Institute for the COVID-19 Genomics UK (COG-UK) Consortium	Jacquelyn Wynn, Mairead Hyland, The Lighthouse Lab in Alderley Park and Alex Alderton, Roberto Amato, Sonia Goncalves, Ewan Harrison, David K. Jackson, Ian Johnston, Dominic Kwiatkowski, Cordelia Langford, John Sillitoe on behalf of the Wellcome Sanger Institute COVID-19 Surveillance Team
EPI_ISL_702996	Liverpool Clinical Laboratories	COVID-19 Genomics UK (COG-UK) Consortium	Sam Haldenby, Anita Lucaci, Steve Paterson, Julian Hiscox, Alistair Darby, M Almsaud, A Alrezaihi, Muhannad Alruwaili, Stuart D Armstrong, Jones Benjamin, Eleanor G Bentley, Anu Chawla, Jordan J Clark, Angela Cowell, Richard Eccles, Isabel García-Dorival, Matthew Gemmell, Alessandro Gerada, PKF Gilmore, Richard Gregory, Ximeng Han, Catherine Hartley, Margaret Hughes, Miren Iturriza-Gomara, James Johnson, L Luu, Jenifer Manson, Charlotte Nelson, Elaine O'Toole, Cassie Olateju, Rebekah Penrice-Randal , Lucille Rainbow, N.P Randle, Trevor Ian Robinson, Parul Sharma, Ghada T Shawli, James P Stewart, Neil Swainston, Ecaterina Vamos, Joanne Watts, Mark Whitehead
EPI_ISL_702997	Lighthouse Lab in Alderley Park	Wellcome Sanger Institute for the COVID-19 Genomics UK (COG-UK) Consortium	Jacquelyn Wynn, Mairead Hyland, The Lighthouse Lab in Alderley Park and Alex Alderton, Roberto Amato, Sonia Goncalves, Ewan Harrison, David K. Jackson, Ian Johnston, Dominic Kwiatkowski, Cordelia Langford, John Sillitoe on behalf of the Wellcome Sanger Institute COVID-19 Surveillance Team
EPI_ISL_702999	University College London, Great Ormond Street Hospital for Children NHS Foundation Trust, Imperial College Healthcare NHS Trust	COVID-19 Genomics UK (COG-UK) Consortium	Sergi Castellano, Rachel Williams, Mark Kristiansen, Paola Resende Silva, Sunando Roy, Tony Brooks, Helena Tutill, Paola Niola, Patricia Dyal, Charlotte Williams, Leysa Forrest, Yasmin Panchbhaya, Jacqueline Findlay, Samuel Weeks, Julianne Brown, Kathryn Harris, Paul Randell, James Price, Alison Holmes, Judith Breuer
EPI_ISL_703010	Northumbria University / South Tees Hospitals NHS Foundation Trust / North Cumbria Integrated Care NHS Foundation Trust / North Tees and Hartlepool NHS Foundation Trust / Newcastle Hospitals NHS Foundation Trust	COVID-19 Genomics UK (COG-UK) Consortium	Darren L Smith,Andrew Nelson,Matthew Bashton,Greg R Young,Joshua Loh,John Allan,Mohammad A Tariq,Giles S Holt,Gary Black,Wen C Yew,Lynn Dover,Paul Baker,Steve Liggett,Sarah Essex,Jane Greenaway,Debra Padgett,Clive Graham,Garren Scott,Edward Barton,Emma Swindells,Brendan Payne,Jennifer Collins,Yusri Taha,Gary Eltringham
EPI_ISL_703012	Lighthouse Lab in Alderley Park	Wellcome Sanger Institute for the COVID-19 Genomics UK (COG-UK) Consortium	Jacquelyn Wynn, Mairead Hyland, The Lighthouse Lab in Alderley Park and Alex Alderton, Roberto Amato, Sonia Goncalves, Ewan Harrison, David K. Jackson, Ian Johnston, Dominic Kwiatkowski, Cordelia Langford, John Sillitoe on behalf of the Wellcome Sanger Institute COVID-19 Surveillance Team
EPI_ISL_703013	Northumbria University / South Tees Hospitals NHS Foundation Trust / North Cumbria Integrated Care NHS Foundation Trust / North Tees and Hartlepool NHS Foundation Trust / Newcastle Hospitals NHS Foundation Trust	COVID-19 Genomics UK (COG-UK) Consortium	Darren L Smith,Andrew Nelson,Matthew Bashton,Greg R Young,Joshua Loh,John Allan,Mohammad A Tariq,Giles S Holt,Gary Black,Wen C Yew,Lynn Dover,Paul Baker,Steve Liggett,Sarah Essex,Jane Greenaway,Debra Padgett,Clive Graham,Garren Scott,Edward Barton,Emma Swindells,Brendan Payne,Jennifer Collins,Yusri Taha,Gary Eltringham
EPI_ISL_703021, EPI_ISL_703022, EPI_ISL_703027, EPI_ISL_703030, EPI_ISL_703033	Lighthouse Lab in Alderley Park	Wellcome Sanger Institute for the COVID-19 Genomics UK (COG-UK) Consortium	Jacquelyn Wynn, Mairead Hyland, The Lighthouse Lab in Alderley Park and Alex Alderton, Roberto Amato, Sonia Goncalves, Ewan Harrison, David K. Jackson, Ian Johnston, Dominic Kwiatkowski, Cordelia Langford, John Sillitoe on behalf of the Wellcome Sanger Institute COVID-19 Surveillance Team
EPI_ISL_703034	Northumbria University / South Tees Hospitals NHS Foundation Trust / North Cumbria Integrated Care NHS Foundation Trust / North Tees and Hartlepool NHS Foundation Trust / Newcastle Hospitals NHS Foundation Trust	COVID-19 Genomics UK (COG-UK) Consortium	Darren L Smith,Andrew Nelson,Matthew Bashton,Greg R Young,Joshua Loh,John Allan,Mohammad A Tariq,Giles S Holt,Gary Black,Wen C Yew,Lynn Dover,Paul Baker,Steve Liggett,Sarah Essex,Jane Greenaway,Debra Padgett,Clive Graham,Garren Scott,Edward Barton,Emma Swindells,Brendan Payne,Jennifer Collins,Yusri Taha,Gary Eltringham
EPI_ISL_703035, EPI_ISL_703039, EPI_ISL_703044, EPI_ISL_703049	Lighthouse Lab in Alderley Park	Wellcome Sanger Institute for the COVID-19 Genomics UK (COG-UK) Consortium	Jacquelyn Wynn, Mairead Hyland, The Lighthouse Lab in Alderley Park and Alex Alderton, Roberto Amato, Sonia Goncalves, Ewan Harrison, David K. Jackson, Ian Johnston, Dominic Kwiatkowski, Cordelia Langford, John Sillitoe on behalf of the Wellcome Sanger Institute COVID-19 Surveillance Team
EPI_ISL_703051	Liverpool Clinical Laboratories	COVID-19 Genomics UK (COG-UK) Consortium	Sam Haldenby, Anita Lucaci, Steve Paterson, Julian Hiscox, Alistair Darby, M Almsaud, A Alrezaihi, Muhannad Alruwaili, Stuart D Armstrong, Jones Benjamin, Eleanor G Bentley, Anu Chawla, Jordan J Clark, Angela Cowell, Richard Eccles, Isabel García-Dorival, Matthew Gemmell, Alessandro Gerada, PKF Gilmore, Richard Gregory, Ximeng Han, Catherine Hartley, Margaret Hughes, Miren Iturriza-Gomara, James Johnson, L Luu, Jenifer Manson, Charlotte Nelson, Elaine O'Toole, Cassie Olateju, Rebekah Penrice-Randal , Lucille Rainbow, N.P Randle, Trevor Ian Robinson, Parul Sharma, Ghada T Shawli, James P Stewart, Neil Swainston, Ecaterina Vamos, Joanne Watts, Mark Whitehead
EPI_ISL_703054, EPI_ISL_703055, EPI_ISL_703057, EPI_ISL_703058	Lighthouse Lab in Alderley Park	Wellcome Sanger Institute for the COVID-19 Genomics UK (COG-UK) Consortium	Jacquelyn Wynn, Mairead Hyland, The Lighthouse Lab in Alderley Park and Alex Alderton, Roberto Amato, Sonia Goncalves, Ewan Harrison, David K. Jackson, Ian Johnston, Dominic Kwiatkowski, Cordelia Langford, John Sillitoe on behalf of the Wellcome Sanger Institute COVID-19 Surveillance Team
EPI_ISL_703064	Northumbria University / South Tees Hospitals NHS Foundation Trust / North Cumbria Integrated Care NHS Foundation Trust / North Tees and Hartlepool NHS Foundation Trust / Newcastle Hospitals NHS Foundation Trust	COVID-19 Genomics UK (COG-UK) Consortium	Darren L Smith,Andrew Nelson,Matthew Bashton,Greg R Young,Joshua Loh,John Allan,Mohammad A Tariq,Giles S Holt,Gary Black,Wen C Yew,Lynn Dover,Paul Baker,Steve Liggett,Sarah Essex,Jane Greenaway,Debra Padgett,Clive Graham,Garren Scott,Edward Barton,Emma Swindells,Brendan Payne,Jennifer Collins,Yusri Taha,Gary Eltringham
EPI_ISL_703066, EPI_ISL_703074	Wales Specialist Virology Centre Sequencing lab: Pathogen Genomics Unit	COVID-19 Genomics UK (COG-UK) Consortium	Catherine Moore, Johnathan Evans, Laura Gifford, Malorie Perry, Simon Cottrell, Angela Marchbank, Alec Birchley, Alexander Adams, Amy Gaskin, Bree Gatica-Wilcox, Jason Coombes, Joel Southgate, Lauren Gilbert, Lee Graham, Nicole Pacchiarini, Sara Kumziene-Summerhayes, Sarah Taylor, Sophie Jones, Sara Rey, Matthew Bull, Joanne Watkins, Sally Corden, Tom Connor
EPI_ISL_703075, EPI_ISL_703081, EPI_ISL_703087, EPI_ISL_703089, EPI_ISL_703094, EPI_ISL_703101	Lighthouse Lab in Alderley Park	Wellcome Sanger Institute for the COVID-19 Genomics UK (COG-UK) Consortium	Jacquelyn Wynn, Mairead Hyland, The Lighthouse Lab in Alderley Park and Alex Alderton, Roberto Amato, Sonia Goncalves, Ewan Harrison, David K. Jackson, Ian Johnston, Dominic Kwiatkowski, Cordelia Langford, John Sillitoe on behalf of the Wellcome Sanger Institute COVID-19 Surveillance Team
EPI_ISL_703105	Northumbria University / South Tees Hospitals NHS Foundation Trust / North Cumbria Integrated Care NHS Foundation Trust / North Tees and Hartlepool NHS Foundation Trust / Newcastle Hospitals NHS Foundation Trust	COVID-19 Genomics UK (COG-UK) Consortium	Darren L Smith,Andrew Nelson,Matthew Bashton,Greg R Young,Joshua Loh,John Allan,Mohammad A Tariq,Giles S Holt,Gary Black,Wen C Yew,Lynn Dover,Paul Baker,Steve Liggett,Sarah Essex,Jane Greenaway,Debra Padgett,Clive Graham,Garren Scott,Edward Barton,Emma Swindells,Brendan Payne,Jennifer Collins,Yusri Taha,Gary Eltringham
EPI_ISL_703109, EPI_ISL_703111, EPI_ISL_703112, EPI_ISL_703114, EPI_ISL_703116, EPI_ISL_703117, EPI_ISL_703119, EPI_ISL_703125, EPI_ISL_703134, EPI_ISL_703137, EPI_ISL_703140, EPI_ISL_703145, EPI_ISL_703148, EPI_ISL_703151, EPI_ISL_703157, EPI_ISL_703164, EPI_ISL_703166, EPI_ISL_703168, EPI_ISL_703171, EPI_ISL_703175			
see above	Lighthouse Lab in Alderley Park	Wellcome Sanger Institute for the COVID-19 Genomics UK (COG-UK) Consortium	Jacquelyn Wynn, Mairead Hyland, The Lighthouse Lab in Alderley Park and Alex Alderton, Roberto Amato, Sonia Goncalves, Ewan Harrison, David K. Jackson, Ian Johnston, Dominic Kwiatkowski, Cordelia Langford, John Sillitoe on behalf of the Wellcome Sanger Institute COVID-19 Surveillance Team
EPI_ISL_703176	Liverpool Clinical Laboratories	COVID-19 Genomics UK (COG-UK) Consortium	Sam Haldenby, Anita Lucaci, Steve Paterson, Julian Hiscox, Alistair Darby, M Almsaud, A Alrezaihi, Muhannad Alruwaili, Stuart D Armstrong, Jones Benjamin, Eleanor G Bentley, Anu Chawla, Jordan J Clark, Angela Cowell, Richard Eccles, Isabel García-Dorival, Matthew Gemmell, Alessandro Gerada, PKF Gilmore, Richard Gregory, Ximeng Han, Catherine Hartley, Margaret Hughes, Miren Iturriza-Gomara, James Johnson, L Luu, Jenifer Manson, Charlotte Nelson, Elaine O'Toole, Cassie Olateju, Rebekah Penrice-Randal , Lucille Rainbow, N.P Randle, Trevor Ian Robinson, Parul Sharma, Ghada T Shawli, James P Stewart, Neil Swainston, Ecaterina Vamos, Joanne Watts, Mark Whitehead
EPI_ISL_703177, EPI_ISL_703178, EPI_ISL_703180, EPI_ISL_703181	Lighthouse Lab in Alderley Park	Wellcome Sanger Institute for the COVID-19 Genomics UK (COG-UK) Consortium	Jacquelyn Wynn, Mairead Hyland, The Lighthouse Lab in Alderley Park and Alex Alderton, Roberto Amato, Sonia Goncalves, Ewan Harrison, David K. Jackson, Ian Johnston, Dominic Kwiatkowski, Cordelia Langford, John Sillitoe on behalf of the Wellcome Sanger Institute COVID-19 Surveillance Team
EPI_ISL_703182	Liverpool Clinical Laboratories	COVID-19 Genomics UK (COG-UK) Consortium	Sam Haldenby, Anita Lucaci, Steve Paterson, Julian Hiscox, Alistair Darby, M Almsaud, A Alrezaihi, Muhannad Alruwaili, Stuart D Armstrong, Jones Benjamin, Eleanor G Bentley, Anu Chawla, Jordan J Clark, Angela Cowell, Richard Eccles, Isabel García-Dorival, Matthew Gemmell, Alessandro Gerada, PKF Gilmore, Richard Gregory, Ximeng Han, Catherine Hartley, Margaret Hughes, Miren Iturriza-Gomara, James Johnson, L Luu, Jenifer Manson, Charlotte Nelson, Elaine O'Toole, Cassie Olateju, Rebekah Penrice-Randal , Lucille Rainbow, N.P Randle, Trevor Ian Robinson, Parul Sharma, Ghada T Shawli, James P Stewart, Neil Swainston, Ecaterina Vamos, Joanne Watts, Mark Whitehead
EPI_ISL_703185, EPI_ISL_703189, EPI_ISL_703191, EPI_ISL_703194, EPI_ISL_703196, EPI_ISL_703199,	Lighthouse Lab in Alderley Park	Wellcome Sanger Institute for the COVID-19 Genomics UK (COG-UK) Consortium	Jacquelyn Wynn, Mairead Hyland, The Lighthouse Lab in Alderley Park and Alex Alderton, Roberto Amato, Sonia Goncalves, Ewan Harrison, David K. Jackson, Ian Johnston, Dominic Kwiatkowski, Cordelia Langford, John Sillitoe on behalf of the Wellcome Sanger Institute COVID-19 Surveillance Team

EPI_ISL_703202, EPI_ISL_703205, EPI_ISL_703214, EPI_ISL_703218			
EPI_ISL_703219	Liverpool Clinical Laboratories	COVID-19 Genomics UK (COG-UK) Consortium	Sam Haldenby, Anita Lucaci, Steve Paterson, Julian Hiscox, Alistair Darby, M Almsaud, A Alrezaihi, Muhannad Alruwaili, Stuart D Armstrong, Jones Benjamin, Eleanor G Bentley, Anu Chawla, Jordan J Clark, Angela Cowell, Richard Eccles, Isabel García-Dorival, Matthew Gemmell, Alessandro Gerada, PKF Gilmore, Richard Gregory, Ximeng Han, Catherine Hartley, Margaret Hughes, Miren Iturriza-Gomara, James Johnson, L Luu, Jenifer Manson, Charlotte Nelson, Elaine O'Toole, Cassie Olateju, Rebekah Penrice-Randal , Lucille Rainbow, N.P Randle, Trevor Ian Robinson, Parul Sharma, Ghada T Shawli, James P Stewart, Neil Swainston, Ecaterina Vamos, Joanne Watts, Mark Whitehead
EPI_ISL_703223, EPI_ISL_703229, EPI_ISL_703234	Lighthouse Lab in Alderley Park	Wellcome Sanger Institute for the COVID-19 Genomics UK (COG-UK) Consortium	Jacquelyn Wynn, Mairead Hyland, The Lighthouse Lab in Alderley Park and Alex Alderton, Roberto Amato, Sonia Goncalves, Ewan Harrison, David K. Jackson, Ian Johnston, Dominic Kwiatkowski, Cordelia Langford, John Sillitoe on behalf of the Wellcome Sanger Institute COVID-19 Surveillance Team
EPI_ISL_703235	Liverpool Clinical Laboratories	COVID-19 Genomics UK (COG-UK) Consortium	Sam Haldenby, Anita Lucaci, Steve Paterson, Julian Hiscox, Alistair Darby, M Almsaud, A Alrezaihi, Muhannad Alruwaili, Stuart D Armstrong, Jones Benjamin, Eleanor G Bentley, Anu Chawla, Jordan J Clark, Angela Cowell, Richard Eccles, Isabel García-Dorival, Matthew Gemmell, Alessandro Gerada, PKF Gilmore, Richard Gregory, Ximeng Han, Catherine Hartley, Margaret Hughes, Miren Iturriza-Gomara, James Johnson, L Luu, Jenifer Manson, Charlotte Nelson, Elaine O'Toole, Cassie Olateju, Rebekah Penrice-Randal , Lucille Rainbow, N.P Randle, Trevor Ian Robinson, Parul Sharma, Ghada T Shawli, James P Stewart, Neil Swainston, Ecaterina Vamos, Joanne Watts, Mark Whitehead
EPI_ISL_703236, EPI_ISL_703239, EPI_ISL_703244	Lighthouse Lab in Alderley Park	Wellcome Sanger Institute for the COVID-19 Genomics UK (COG-UK) Consortium	Jacquelyn Wynn, Mairead Hyland, The Lighthouse Lab in Alderley Park and Alex Alderton, Roberto Amato, Sonia Goncalves, Ewan Harrison, David K. Jackson, Ian Johnston, Dominic Kwiatkowski, Cordelia Langford, John Sillitoe on behalf of the Wellcome Sanger Institute COVID-19 Surveillance Team
EPI_ISL_703246	West of Scotland Specialist Virology Centre, NHSGGC / MRC-University of Glasgow Centre for Virus Research	COVID-19 Genomics UK (COG-UK) Consortium	Ana da Silva Filipe, Natasha Johnson, Kathy Smollett, Daniel Mair, Stephen Carmichael, Alice Broos, Lily Tong, Jenna Nichols, Kyriaki Nomikou; Sarah McDonald; Richard Orton, Joseph Hughes, Sreenu Vattipally, David L Robertson; Alasdair MacLean, Rory Gunson; Sharif Shaaban, Matthew Holden; Rachel Blacow, Guy Mollett, Kathy Li, James Shepherd, Antonia Ho, Emma Thomson
EPI_ISL_703251	Wales Specialist Virology Centre Sequencing lab: Pathogen Genomics Unit	COVID-19 Genomics UK (COG-UK) Consortium	Catherine Moore, Johnathan Evans, Laura Gifford, Malorie Perry, Simon Cottrell, Angela Marchbank, Alec Birchley, Alexander Adams, Amy Gaskin, Bree Gatica-Wilcox, Jason Coombes, Joel Southgate, Lauren Gilbert, Lee Graham, Nicole Pacchiarini, Sara Kumziene-Summerhayes, Sarah Taylor, Sophie Jones, Sara Rey, Matthew Bull, Joanne Watkins, Sally Corden, Tom Connor
EPI_ISL_703269	West of Scotland Specialist Virology Centre, NHSGGC / MRC-University of Glasgow Centre for Virus Research	COVID-19 Genomics UK (COG-UK) Consortium	Ana da Silva Filipe, Natasha Johnson, Kathy Smollett, Daniel Mair, Stephen Carmichael, Alice Broos, Lily Tong, Jenna Nichols, Kyriaki Nomikou; Sarah McDonald; Richard Orton, Joseph Hughes, Sreenu Vattipally, David L Robertson; Alasdair MacLean, Rory Gunson; Sharif Shaaban, Matthew Holden; Rachel Blacow, Guy Mollett, Kathy Li, James Shepherd, Antonia Ho, Emma Thomson
EPI_ISL_703272	Liverpool Clinical Laboratories	COVID-19 Genomics UK (COG-UK) Consortium	Sam Haldenby, Anita Lucaci, Steve Paterson, Julian Hiscox, Alistair Darby, M Almsaud, A Alrezaihi, Muhannad Alruwaili, Stuart D Armstrong, Jones Benjamin, Eleanor G Bentley, Anu Chawla, Jordan J Clark, Angela Cowell, Richard Eccles, Isabel García-Dorival, Matthew Gemmell, Alessandro Gerada, PKF Gilmore, Richard Gregory, Ximeng Han, Catherine Hartley, Margaret Hughes, Miren Iturriza-Gomara, James Johnson, L Luu, Jenifer Manson, Charlotte Nelson, Elaine O'Toole, Cassie Olateju, Rebekah Penrice-Randal , Lucille Rainbow, N.P Randle, Trevor Ian Robinson, Parul Sharma, Ghada T Shawli, James P Stewart, Neil Swainston, Ecaterina Vamos, Joanne Watts, Mark Whitehead
EPI_ISL_703278	Wales Specialist Virology Centre Sequencing lab: Pathogen Genomics Unit	COVID-19 Genomics UK (COG-UK) Consortium	Catherine Moore, Johnathan Evans, Laura Gifford, Malorie Perry, Simon Cottrell, Angela Marchbank, Alec Birchley, Alexander Adams, Amy Gaskin, Bree Gatica-Wilcox, Jason Coombes, Joel Southgate, Lauren Gilbert, Lee Graham, Nicole Pacchiarini, Sara Kumziene-Summerhayes, Sarah Taylor, Sophie Jones, Sara Rey, Matthew Bull, Joanne Watkins, Sally Corden, Tom Connor
EPI_ISL_703319	University College London, Great Ormond Street Hospital for Children NHS Foundation Trust, Imperial College Healthcare NHS Trust	COVID-19 Genomics UK (COG-UK) Consortium	Sergi Castellano, Rachel Williams, Mark Kristiansen, Paola Resende Silva, Sunando Roy, Tony Brooks, Helena Tutill, Paola Niola, Patricia Dyal, Charlotte Williams, Leysa Forrest, Yasmin Panchbhaya, Jacqueline Findlay, Samuel Weeks, Julianne Brown, Kathryn Harris, Paul Randell, James Price, Alison Holmes, Judith Breuer
EPI_ISL_703321	Wales Specialist Virology Centre Sequencing lab: Pathogen Genomics Unit	COVID-19 Genomics UK (COG-UK) Consortium	Catherine Moore, Johnathan Evans, Laura Gifford, Malorie Perry, Simon Cottrell, Angela Marchbank, Alec Birchley, Alexander Adams, Amy Gaskin, Bree Gatica-Wilcox, Jason Coombes, Joel Southgate, Lauren Gilbert, Lee Graham, Nicole Pacchiarini, Sara Kumziene-Summerhayes, Sarah Taylor, Sophie Jones, Sara Rey, Matthew Bull, Joanne Watkins, Sally Corden, Tom Connor
EPI_ISL_703324	Liverpool Clinical Laboratories	COVID-19 Genomics UK (COG-UK) Consortium	Sam Haldenby, Anita Lucaci, Steve Paterson, Julian Hiscox, Alistair Darby, M Almsaud, A Alrezaihi, Muhannad Alruwaili, Stuart D Armstrong, Jones Benjamin, Eleanor G Bentley, Anu Chawla, Jordan J Clark, Angela Cowell, Richard Eccles, Isabel García-Dorival, Matthew Gemmell, Alessandro Gerada, PKF Gilmore, Richard Gregory, Ximeng Han, Catherine Hartley, Margaret Hughes, Miren Iturriza-Gomara, James Johnson, L Luu, Jenifer Manson, Charlotte Nelson, Elaine O'Toole, Cassie Olateju, Rebekah Penrice-Randal , Lucille Rainbow, N.P Randle, Trevor Ian Robinson, Parul Sharma, Ghada T Shawli, James P Stewart, Neil Swainston, Ecaterina Vamos, Joanne Watts, Mark Whitehead
EPI_ISL_703330	Wales Specialist Virology Centre Sequencing lab: Pathogen Genomics Unit	COVID-19 Genomics UK (COG-UK) Consortium	Catherine Moore, Johnathan Evans, Laura Gifford, Malorie Perry, Simon Cottrell, Angela Marchbank, Alec Birchley, Alexander Adams, Amy Gaskin, Bree Gatica-Wilcox, Jason Coombes, Joel Southgate, Lauren Gilbert, Lee Graham, Nicole Pacchiarini, Sara Kumziene-Summerhayes, Sarah Taylor, Sophie Jones, Sara Rey, Matthew Bull, Joanne Watkins, Sally Corden, Tom Connor
EPI_ISL_703335	University College London, Great Ormond Street Hospital for Children NHS Foundation Trust, Imperial College Healthcare NHS Trust	COVID-19 Genomics UK (COG-UK) Consortium	Sergi Castellano, Rachel Williams, Mark Kristiansen, Paola Resende Silva, Sunando Roy, Tony Brooks, Helena Tutill, Paola Niola, Patricia Dyal, Charlotte Williams, Leysa Forrest, Yasmin Panchbhaya, Jacqueline Findlay, Samuel Weeks, Julianne Brown, Kathryn Harris, Paul Randell, James Price, Alison Holmes, Judith Breuer
EPI_ISL_703343, EPI_ISL_703359	Wales Specialist Virology Centre Sequencing lab: Pathogen Genomics Unit	COVID-19 Genomics UK (COG-UK) Consortium	Catherine Moore, Johnathan Evans, Laura Gifford, Malorie Perry, Simon Cottrell, Angela Marchbank, Alec Birchley, Alexander Adams, Amy Gaskin, Bree Gatica-Wilcox, Jason Coombes, Joel Southgate, Lauren Gilbert, Lee Graham, Nicole Pacchiarini, Sara Kumziene-Summerhayes, Sarah Taylor, Sophie Jones, Sara Rey, Matthew Bull, Joanne Watkins, Sally Corden, Tom Connor
EPI_ISL_703364, EPI_ISL_703375	Liverpool Clinical Laboratories	COVID-19 Genomics UK (COG-UK) Consortium	Sam Haldenby, Anita Lucaci, Steve Paterson, Julian Hiscox, Alistair Darby, M Almsaud, A Alrezaihi, Muhannad Alruwaili, Stuart D Armstrong, Jones Benjamin, Eleanor G Bentley, Anu Chawla, Jordan J Clark, Angela Cowell, Richard Eccles, Isabel García-Dorival, Matthew Gemmell, Alessandro Gerada, PKF Gilmore, Richard Gregory, Ximeng Han, Catherine Hartley, Margaret Hughes, Miren Iturriza-Gomara, James Johnson, L Luu, Jenifer Manson, Charlotte Nelson, Elaine O'Toole, Cassie Olateju, Rebekah Penrice-Randal , Lucille Rainbow, N.P Randle, Trevor Ian Robinson, Parul Sharma, Ghada T Shawli, James P Stewart, Neil Swainston, Ecaterina Vamos, Joanne Watts, Mark Whitehead
EPI_ISL_703377	Northumbria University / South Tees Hospitals NHS Foundation Trust / North Cumbria Integrated Care NHS Foundation Trust / North Tees and Hartlepool NHS Foundation Trust / Newcastle Hospitals NHS Foundation Trust	COVID-19 Genomics UK (COG-UK) Consortium	Darren L Smith,Andrew Nelson,Matthew Bashton,Greg R Young,Joshua Loh,John Allan,Mohammad A Tariq,Giles S Holt,Gary Black,Wen C Yew,Lynn Dover,Paul Baker,Steve Liggett,Sarah Essex,Jane Greenaway,Debra Padgett,Clive Graham,Garren Scott,Edward Barton,Emma Swindells,Brendan Payne,Jennifer Collins,Yusri Taha,Gary Eltringham
EPI_ISL_703383, EPI_ISL_703385	University College London, Great Ormond Street Hospital for Children NHS Foundation Trust, Imperial College Healthcare NHS Trust	COVID-19 Genomics UK (COG-UK) Consortium	Sergi Castellano, Rachel Williams, Mark Kristiansen, Paola Resende Silva, Sunando Roy, Tony Brooks, Helena Tutill, Paola Niola, Patricia Dyal, Charlotte Williams, Leysa Forrest, Yasmin Panchbhaya, Jacqueline Findlay, Samuel Weeks, Julianne Brown, Kathryn Harris, Paul Randell, James Price, Alison Holmes, Judith Breuer
EPI_ISL_703387	Liverpool Clinical Laboratories	COVID-19 Genomics UK (COG-UK) Consortium	Sam Haldenby, Anita Lucaci, Steve Paterson, Julian Hiscox, Alistair Darby, M Almsaud, A Alrezaihi, Muhannad Alruwaili, Stuart D Armstrong, Jones Benjamin, Eleanor G Bentley, Anu Chawla, Jordan J Clark, Angela Cowell, Richard Eccles, Isabel García-Dorival, Matthew Gemmell, Alessandro Gerada, PKF Gilmore, Richard Gregory, Ximeng Han, Catherine Hartley, Margaret Hughes, Miren Iturriza-Gomara, James Johnson, L Luu, Jenifer Manson, Charlotte Nelson, Elaine O'Toole, Cassie Olateju, Rebekah Penrice-Randal , Lucille Rainbow, N.P Randle, Trevor Ian Robinson, Parul Sharma, Ghada T Shawli, James P Stewart, Neil Swainston, Ecaterina Vamos, Joanne Watts, Mark Whitehead
EPI_ISL_703390	Northumbria University / South Tees Hospitals NHS Foundation Trust / North Cumbria Integrated Care NHS Foundation Trust / North Tees and Hartlepool NHS Foundation Trust / Newcastle Hospitals NHS Foundation Trust	COVID-19 Genomics UK (COG-UK) Consortium	Darren L Smith,Andrew Nelson,Matthew Bashton,Greg R Young,Joshua Loh,John Allan,Mohammad A Tariq,Giles S Holt,Gary Black,Wen C Yew,Lynn Dover,Paul Baker,Steve Liggett,Sarah Essex,Jane Greenaway,Debra Padgett,Clive Graham,Garren Scott,Edward Barton,Emma Swindells,Brendan Payne,Jennifer Collins,Yusri Taha,Gary Eltringham
EPI_ISL_703395, EPI_ISL_703398	Wales Specialist Virology Centre Sequencing lab: Pathogen Genomics Unit	COVID-19 Genomics UK (COG-UK) Consortium	Catherine Moore, Johnathan Evans, Laura Gifford, Malorie Perry, Simon Cottrell, Angela Marchbank, Alec Birchley, Alexander Adams, Amy Gaskin, Bree Gatica-Wilcox, Jason Coombes, Joel Southgate, Lauren Gilbert, Lee Graham, Nicole Pacchiarini, Sara Kumziene-Summerhayes, Sarah Taylor, Sophie Jones, Sara Rey, Matthew Bull, Joanne Watkins, Sally Corden, Tom Connor

EPI_ISL_703484, EPI_ISL_703542, EPI_ISL_703545	Northumbria University / South Tees Hospitals NHS Foundation Trust / North Cumbria Integrated Care NHS Foundation Trust / North Tees and Hartlepool NHS Foundation Trust / Newcastle Hospitals NHS Foundation Trust	COVID-19 Genomics UK (COG-UK) Consortium	Darren L Smith,Andrew Nelson,Matthew Bashton,Greg R Young,Joshua Loh,John Allan,Mohammad A Tariq,Giles S Holt,Gary Black,Wen C Yew,Lynn Dover,Paul Baker,Steve Liggett,Sarah Essex,Jane Greenaway,Debra Padgett,Clive Graham,Garren Scott,Edward Barton,Emma Swindells,Brendan Payne,Jennifer Collins,Yusri Taha,Gary Eltringham
EPI_ISL_703577	Liverpool Clinical Laboratories	COVID-19 Genomics UK (COG-UK) Consortium	Sam Haldenby, Anita Lucaci, Steve Paterson, Julian Hiscox, Alistair Darby, M Almsaud, A Alrezaihi, Muhannad Alruwaili, Stuart D Armstrong, Jones Benjamin, Eleanor G Bentley, Anu Chawla, Jordan J Clark, Angela Cowell, Richard Eccles, Isabel García-Dorival, Matthew Gemmell, Alessandro Gerada, PKF Gilmore, Richard Gregory, Ximeng Han, Catherine Hartley, Margaret Hughes, Miren Iturriza-Gomara, James Johnson, L Luu, Jenifer Manson, Charlotte Nelson, Elaine O'Toole, Cassie Olateju, Rebekah Penrice-Randal , Lucille Rainbow, N.P Randle, Trevor Ian Robinson, Parul Sharma, Ghada T Shawli, James P Stewart, Neil Swainston, Ecaterina Vamos, Joanne Watts, Mark Whitehead
EPI_ISL_703614	Wales Specialist Virology Centre Sequencing lab: Pathogen Genomics Unit	COVID-19 Genomics UK (COG-UK) Consortium	Catherine Moore, Johnathan Evans, Laura Gifford, Malorie Perry, Simon Cottrell, Angela Marchbank, Alec Birchley, Alexander Adams, Amy Gaskin, Bree Gatica-Wilcox, Jason Coombes, Joel Southgate, Lauren Gilbert, Lee Graham, Nicole Pacchiarini, Sara Kumziene-Summerhayes, Sarah Taylor, Sophie Jones, Sara Rey, Matthew Bull, Joanne Watkins, Sally Corden, Tom Connor
EPI_ISL_703624, EPI_ISL_703698	Liverpool Clinical Laboratories	COVID-19 Genomics UK (COG-UK) Consortium	Sam Haldenby, Anita Lucaci, Steve Paterson, Julian Hiscox, Alistair Darby, M Almsaud, A Alrezaihi, Muhannad Alruwaili, Stuart D Armstrong, Jones Benjamin, Eleanor G Bentley, Anu Chawla, Jordan J Clark, Angela Cowell, Richard Eccles, Isabel García-Dorival, Matthew Gemmell, Alessandro Gerada, PKF Gilmore, Richard Gregory, Ximeng Han, Catherine Hartley, Margaret Hughes, Miren Iturriza-Gomara, James Johnson, L Luu, Jenifer Manson, Charlotte Nelson, Elaine O'Toole, Cassie Olateju, Rebekah Penrice-Randal , Lucille Rainbow, N.P Randle, Trevor Ian Robinson, Parul Sharma, Ghada T Shawli, James P Stewart, Neil Swainston, Ecaterina Vamos, Joanne Watts, Mark Whitehead
EPI_ISL_703704	Northumbria University / South Tees Hospitals NHS Foundation Trust / North Cumbria Integrated Care NHS Foundation Trust / North Tees and Hartlepool NHS Foundation Trust / Newcastle Hospitals NHS Foundation Trust	COVID-19 Genomics UK (COG-UK) Consortium	Darren L Smith,Andrew Nelson,Matthew Bashton,Greg R Young,Joshua Loh,John Allan,Mohammad A Tariq,Giles S Holt,Gary Black,Wen C Yew,Lynn Dover,Paul Baker,Steve Liggett,Sarah Essex,Jane Greenaway,Debra Padgett,Clive Graham,Garren Scott,Edward Barton,Emma Swindells,Brendan Payne,Jennifer Collins,Yusri Taha,Gary Eltringham
EPI_ISL_703706	University College London, Great Ormond Street Hospital for Children NHS Foundation Trust, Imperial College Healthcare NHS Trust	COVID-19 Genomics UK (COG-UK) Consortium	Sergi Castellano, Rachel Williams, Mark Kristiansen, Paola Resende Silva, Sunando Roy, Tony Brooks, Helena Tutill, Paola Niola, Patricia Dyal, Charlotte Williams, Leysa Forrest, Yasmin Panchbhaya, Jacqueline Findlay, Samuel Weeks, Julianne Brown, Kathryn Harris, Paul Randell, James Price, Alison Holmes, Judith Breuer
EPI_ISL_703717	West of Scotland Specialist Virology Centre, NHSGGC / MRC-University of Glasgow Centre for Virus Research	COVID-19 Genomics UK (COG-UK) Consortium	Ana da Silva Filipe, Natasha Johnson, Kathy Smollett, Daniel Mair, Stephen Carmichael, Alice Broos, Lily Tong, Jenna Nichols, Kyriaki Nomikou; Sarah McDonald; Richard Orton, Joseph Hughes, Sreenu Vattipally, David L Robertson; Alasdair MacLean, Rory Gunson; Sharif Shaaban, Matthew Holden; Rachel Blacow, Guy Mollett, Kathy Li, James Shepherd, Antonia Ho, Emma Thomson
EPI_ISL_703728	Northumbria University / South Tees Hospitals NHS Foundation Trust / North Cumbria Integrated Care NHS Foundation Trust / North Tees and Hartlepool NHS Foundation Trust / Newcastle Hospitals NHS Foundation Trust	COVID-19 Genomics UK (COG-UK) Consortium	Darren L Smith,Andrew Nelson,Matthew Bashton,Greg R Young,Joshua Loh,John Allan,Mohammad A Tariq,Giles S Holt,Gary Black,Wen C Yew,Lynn Dover,Paul Baker,Steve Liggett,Sarah Essex,Jane Greenaway,Debra Padgett,Clive Graham,Garren Scott,Edward Barton,Emma Swindells,Brendan Payne,Jennifer Collins,Yusri Taha,Gary Eltringham
EPI_ISL_703732	Liverpool Clinical Laboratories	COVID-19 Genomics UK (COG-UK) Consortium	Sam Haldenby, Anita Lucaci, Steve Paterson, Julian Hiscox, Alistair Darby, M Almsaud, A Alrezaihi, Muhannad Alruwaili, Stuart D Armstrong, Jones Benjamin, Eleanor G Bentley, Anu Chawla, Jordan J Clark, Angela Cowell, Richard Eccles, Isabel García-Dorival, Matthew Gemmell, Alessandro Gerada, PKF Gilmore, Richard Gregory, Ximeng Han, Catherine Hartley, Margaret Hughes, Miren Iturriza-Gomara, James Johnson, L Luu, Jenifer Manson, Charlotte Nelson, Elaine O'Toole, Cassie Olateju, Rebekah Penrice-Randal , Lucille Rainbow, N.P Randle, Trevor Ian Robinson, Parul Sharma, Ghada T Shawli, James P Stewart, Neil Swainston, Ecaterina Vamos, Joanne Watts, Mark Whitehead
EPI_ISL_703739	University College London, Great Ormond Street Hospital for Children NHS Foundation Trust, Imperial College Healthcare NHS Trust	COVID-19 Genomics UK (COG-UK) Consortium	Sergi Castellano, Rachel Williams, Mark Kristiansen, Paola Resende Silva, Sunando Roy, Tony Brooks, Helena Tutill, Paola Niola, Patricia Dyal, Charlotte Williams, Leysa Forrest, Yasmin Panchbhaya, Jacqueline Findlay, Samuel Weeks, Julianne Brown, Kathryn Harris, Paul Randell, James Price, Alison Holmes, Judith Breuer
EPI_ISL_703746	Wales Specialist Virology Centre Sequencing lab: Pathogen Genomics Unit	COVID-19 Genomics UK (COG-UK) Consortium	Catherine Moore, Johnathan Evans, Laura Gifford, Malorie Perry, Simon Cottrell, Angela Marchbank, Alec Birchley, Alexander Adams, Amy Gaskin, Bree Gatica-Wilcox, Jason Coombes, Joel Southgate, Lauren Gilbert, Lee Graham, Nicole Pacchiarini, Sara Kumziene-Summerhayes, Sarah Taylor, Sophie Jones, Sara Rey, Matthew Bull, Joanne Watkins, Sally Corden, Tom Connor
EPI_ISL_703748, EPI_ISL_703749, EPI_ISL_703756	West of Scotland Specialist Virology Centre, NHSGGC / MRC-University of Glasgow Centre for Virus Research	COVID-19 Genomics UK (COG-UK) Consortium	Ana da Silva Filipe, Natasha Johnson, Kathy Smollett, Daniel Mair, Stephen Carmichael, Alice Broos, Lily Tong, Jenna Nichols, Kyriaki Nomikou; Sarah McDonald; Richard Orton, Joseph Hughes, Sreenu Vattipally, David L Robertson; Alasdair MacLean, Rory Gunson; Sharif Shaaban, Matthew Holden; Rachel Blacow, Guy Mollett, Kathy Li, James Shepherd, Antonia Ho, Emma Thomson
EPI_ISL_703760, EPI_ISL_703761	Wales Specialist Virology Centre Sequencing lab: Pathogen Genomics Unit	COVID-19 Genomics UK (COG-UK) Consortium	Catherine Moore, Johnathan Evans, Laura Gifford, Malorie Perry, Simon Cottrell, Angela Marchbank, Alec Birchley, Alexander Adams, Amy Gaskin, Bree Gatica-Wilcox, Jason Coombes, Joel Southgate, Lauren Gilbert, Lee Graham, Nicole Pacchiarini, Sara Kumziene-Summerhayes, Sarah Taylor, Sophie Jones, Sara Rey, Matthew Bull, Joanne Watkins, Sally Corden, Tom Connor
EPI_ISL_703767	University College London, Great Ormond Street Hospital for Children NHS Foundation Trust, Imperial College Healthcare NHS Trust	COVID-19 Genomics UK (COG-UK) Consortium	Sergi Castellano, Rachel Williams, Mark Kristiansen, Paola Resende Silva, Sunando Roy, Tony Brooks, Helena Tutill, Paola Niola, Patricia Dyal, Charlotte Williams, Leysa Forrest, Yasmin Panchbhaya, Jacqueline Findlay, Samuel Weeks, Julianne Brown, Kathryn Harris, Paul Randell, James Price, Alison Holmes, Judith Breuer
EPI_ISL_703768	Wales Specialist Virology Centre Sequencing lab: Pathogen Genomics Unit	COVID-19 Genomics UK (COG-UK) Consortium	Catherine Moore, Johnathan Evans, Laura Gifford, Malorie Perry, Simon Cottrell, Angela Marchbank, Alec Birchley, Alexander Adams, Amy Gaskin, Bree Gatica-Wilcox, Jason Coombes, Joel Southgate, Lauren Gilbert, Lee Graham, Nicole Pacchiarini, Sara Kumziene-Summerhayes, Sarah Taylor, Sophie Jones, Sara Rey, Matthew Bull, Joanne Watkins, Sally Corden, Tom Connor
EPI_ISL_703770	Liverpool Clinical Laboratories	COVID-19 Genomics UK (COG-UK) Consortium	Sam Haldenby, Anita Lucaci, Steve Paterson, Julian Hiscox, Alistair Darby, M Almsaud, A Alrezaihi, Muhannad Alruwaili, Stuart D Armstrong, Jones Benjamin, Eleanor G Bentley, Anu Chawla, Jordan J Clark, Angela Cowell, Richard Eccles, Isabel García-Dorival, Matthew Gemmell, Alessandro Gerada, PKF Gilmore, Richard Gregory, Ximeng Han, Catherine Hartley, Margaret Hughes, Miren Iturriza-Gomara, James Johnson, L Luu, Jenifer Manson, Charlotte Nelson, Elaine O'Toole, Cassie Olateju, Rebekah Penrice-Randal , Lucille Rainbow, N.P Randle, Trevor Ian Robinson, Parul Sharma, Ghada T Shawli, James P Stewart, Neil Swainston, Ecaterina Vamos, Joanne Watts, Mark Whitehead
EPI_ISL_703810	Virology Department, Royal Infirmary of Edinburgh, NHS Lothian / School of Biological Sciences, University of Edinburgh / Institute of Genetics and Molecular Medicine, University of Edinburgh	COVID-19 Genomics UK (COG-UK) Consortium	McHugh M, Dewar R, Rooke S, Gallagher M, Balcaza C, O'Toole Á, Scher E, Hill V, McCrone JT, Colquhoun R, Yu X, Jackson B, Rambaut A, Williams TC, Templeton K
EPI_ISL_703858, EPI_ISL_703860	Wales Specialist Virology Centre Sequencing lab: Pathogen Genomics Unit	COVID-19 Genomics UK (COG-UK) Consortium	Catherine Moore, Johnathan Evans, Laura Gifford, Malorie Perry, Simon Cottrell, Angela Marchbank, Alec Birchley, Alexander Adams, Amy Gaskin, Bree Gatica-Wilcox, Jason Coombes, Joel Southgate, Lauren Gilbert, Lee Graham, Nicole Pacchiarini, Sara Kumziene-Summerhayes, Sarah Taylor, Sophie Jones, Sara Rey, Matthew Bull, Joanne Watkins, Sally Corden, Tom Connor
EPI_ISL_703865	University College London, Great Ormond Street Hospital for Children NHS Foundation Trust, Imperial College Healthcare NHS Trust	COVID-19 Genomics UK (COG-UK) Consortium	Sergi Castellano, Rachel Williams, Mark Kristiansen, Paola Resende Silva, Sunando Roy, Tony Brooks, Helena Tutill, Paola Niola, Patricia Dyal, Charlotte Williams, Leysa Forrest, Yasmin Panchbhaya, Jacqueline Findlay, Samuel Weeks, Julianne Brown, Kathryn Harris, Paul Randell, James Price, Alison Holmes, Judith Breuer
EPI_ISL_703870, EPI_ISL_703873	Wales Specialist Virology Centre Sequencing lab: Pathogen Genomics Unit	COVID-19 Genomics UK (COG-UK) Consortium	Catherine Moore, Johnathan Evans, Laura Gifford, Malorie Perry, Simon Cottrell, Angela Marchbank, Alec Birchley, Alexander Adams, Amy Gaskin, Bree Gatica-Wilcox, Jason Coombes, Joel Southgate, Lauren Gilbert, Lee Graham, Nicole Pacchiarini, Sara Kumziene-Summerhayes, Sarah Taylor, Sophie Jones, Sara Rey, Matthew Bull, Joanne Watkins, Sally Corden, Tom Connor
EPI_ISL_703879	University College London, Great Ormond Street Hospital for Children NHS Foundation Trust, Imperial College Healthcare NHS Trust	COVID-19 Genomics UK (COG-UK) Consortium	Sergi Castellano, Rachel Williams, Mark Kristiansen, Paola Resende Silva, Sunando Roy, Tony Brooks, Helena Tutill, Paola Niola, Patricia Dyal, Charlotte Williams, Leysa Forrest, Yasmin Panchbhaya, Jacqueline Findlay, Samuel Weeks, Julianne Brown, Kathryn Harris, Paul Randell, James Price, Alison Holmes, Judith Breuer
EPI_ISL_703884, EPI_ISL_703891, EPI_ISL_703897	Liverpool Clinical Laboratories	COVID-19 Genomics UK (COG-UK) Consortium	Sam Haldenby, Anita Lucaci, Steve Paterson, Julian Hiscox, Alistair Darby, M Almsaud, A Alrezaihi, Muhannad Alruwaili, Stuart D Armstrong, Jones Benjamin, Eleanor G Bentley, Anu Chawla, Jordan J Clark, Angela Cowell, Richard Eccles, Isabel García-Dorival, Matthew Gemmell, Alessandro Gerada,

			PKF Gilmore, Richard Gregory, Ximeng Han, Catherine Hartley, Margaret Hughes, Miren Iturriza-Gomara, James Johnson, L Luu, Jenifer Manson, Charlotte Nelson, Elaine O'Toole, Cassie Olateju, Rebekah Penrice-Randal , Lucille Rainbow, N.P Randle, Trevor Ian Robinson, Parul Sharma, Ghada T Shawli, James P Stewart, Neil Swainston, Ecaterina Vamos, Joanne Watts, Mark Whitehead
EPI_ISL_703911	West of Scotland Specialist Virology Centre, NHSGGC / MRC-University of Glasgow Centre for Virus Research	COVID-19 Genomics UK (COG-UK) Consortium	Ana da Silva Filipe, Natasha Johnson, Kathy Smollett, Daniel Mair, Stephen Carmichael, Alice Broos, Lily Tong, Jenna Nichols, Kyriaki Nomikou; Sarah McDonald; Richard Orton, Joseph Hughes, Sreenu Vattipally, David L Robertson; Alasdair MacLean, Rory Gunson; Sharif Shaaban, Matthew Holden; Rachel Blacow, Guy Mollett, Kathy Li, James Shepherd, Antonia Ho, Emma Thomson
EPI_ISL_703937	Liverpool Clinical Laboratories	COVID-19 Genomics UK (COG-UK) Consortium	Sam Haldenby, Anita Lucaci, Steve Paterson, Julian Hiscox, Alistair Darby, M Almsaud, A Alrezaihi, Muhannad Alruwaili, Stuart D Armstrong, Jones Benjamin, Eleanor G Bentley, Anu Chawla, Jordan J Clark, Angela Cowell, Richard Eccles, Isabel García-Dorival, Matthew Gemmell, Alessandro Gerada, PKF Gilmore, Richard Gregory, Ximeng Han, Catherine Hartley, Margaret Hughes, Miren Iturriza-Gomara, James Johnson, L Luu, Jenifer Manson, Charlotte Nelson, Elaine O'Toole, Cassie Olateju, Rebekah Penrice-Randal , Lucille Rainbow, N.P Randle, Trevor Ian Robinson, Parul Sharma, Ghada T Shawli, James P Stewart, Neil Swainston, Ecaterina Vamos, Joanne Watts, Mark Whitehead
EPI_ISL_703955	Wales Specialist Virology Centre Sequencing lab: Pathogen Genomics Unit	COVID-19 Genomics UK (COG-UK) Consortium	Catherine Moore, Johnathan Evans, Laura Gifford, Malorie Perry, Simon Cottrell, Angela Marchbank, Alec Birchley, Alexander Adams, Amy Gaskin, Bree Gatica-Wilcox, Jason Coombes, Joel Southgate, Lauren Gilbert, Lee Graham, Nicole Pacchiarini, Sara Kumziene-Summerhayes, Sarah Taylor, Sophie Jones, Sara Rey, Matthew Bull, Joanne Watkins, Sally Corden, Tom Connor
EPI_ISL_703977	Liverpool Clinical Laboratories	COVID-19 Genomics UK (COG-UK) Consortium	Sam Haldenby, Anita Lucaci, Steve Paterson, Julian Hiscox, Alistair Darby, M Almsaud, A Alrezaihi, Muhannad Alruwaili, Stuart D Armstrong, Jones Benjamin, Eleanor G Bentley, Anu Chawla, Jordan J Clark, Angela Cowell, Richard Eccles, Isabel García-Dorival, Matthew Gemmell, Alessandro Gerada, PKF Gilmore, Richard Gregory, Ximeng Han, Catherine Hartley, Margaret Hughes, Miren Iturriza-Gomara, James Johnson, L Luu, Jenifer Manson, Charlotte Nelson, Elaine O'Toole, Cassie Olateju, Rebekah Penrice-Randal , Lucille Rainbow, N.P Randle, Trevor Ian Robinson, Parul Sharma, Ghada T Shawli, James P Stewart, Neil Swainston, Ecaterina Vamos, Joanne Watts, Mark Whitehead
EPI_ISL_704001, EPI_ISL_704014	Wales Specialist Virology Centre Sequencing lab: Pathogen Genomics Unit	COVID-19 Genomics UK (COG-UK) Consortium	Catherine Moore, Johnathan Evans, Laura Gifford, Malorie Perry, Simon Cottrell, Angela Marchbank, Alec Birchley, Alexander Adams, Amy Gaskin, Bree Gatica-Wilcox, Jason Coombes, Joel Southgate, Lauren Gilbert, Lee Graham, Nicole Pacchiarini, Sara Kumziene-Summerhayes, Sarah Taylor, Sophie Jones, Sara Rey, Matthew Bull, Joanne Watkins, Sally Corden, Tom Connor
EPI_ISL_704025	University College London, Great Ormond Street Hospital for Children NHS Foundation Trust, Imperial College Healthcare NHS Trust	COVID-19 Genomics UK (COG-UK) Consortium	Sergi Castellano, Rachel Williams, Mark Kristiansen, Paola Resende Silva, Sunando Roy, Tony Brooks, Helena Tutill, Paola Niola, Patricia Dyal, Charlotte Williams, Leysa Forrest, Yasmin Panchbhaya, Jacqueline Findlay, Samuel Weeks, Julianne Brown, Kathryn Harris, Paul Randell, James Price, Alison Holmes, Judith Breuer
EPI_ISL_704030	Wales Specialist Virology Centre Sequencing lab: Pathogen Genomics Unit	COVID-19 Genomics UK (COG-UK) Consortium	Catherine Moore, Johnathan Evans, Laura Gifford, Malorie Perry, Simon Cottrell, Angela Marchbank, Alec Birchley, Alexander Adams, Amy Gaskin, Bree Gatica-Wilcox, Jason Coombes, Joel Southgate, Lauren Gilbert, Lee Graham, Nicole Pacchiarini, Sara Kumziene-Summerhayes, Sarah Taylor, Sophie Jones, Sara Rey, Matthew Bull, Joanne Watkins, Sally Corden, Tom Connor
EPI_ISL_704036	University College London, Great Ormond Street Hospital for Children NHS Foundation Trust, Imperial College Healthcare NHS Trust	COVID-19 Genomics UK (COG-UK) Consortium	Sergi Castellano, Rachel Williams, Mark Kristiansen, Paola Resende Silva, Sunando Roy, Tony Brooks, Helena Tutill, Paola Niola, Patricia Dyal, Charlotte Williams, Leysa Forrest, Yasmin Panchbhaya, Jacqueline Findlay, Samuel Weeks, Julianne Brown, Kathryn Harris, Paul Randell, James Price, Alison Holmes, Judith Breuer
EPI_ISL_704038, EPI_ISL_704044	Wales Specialist Virology Centre Sequencing lab: Pathogen Genomics Unit	COVID-19 Genomics UK (COG-UK) Consortium	Catherine Moore, Johnathan Evans, Laura Gifford, Malorie Perry, Simon Cottrell, Angela Marchbank, Alec Birchley, Alexander Adams, Amy Gaskin, Bree Gatica-Wilcox, Jason Coombes, Joel Southgate, Lauren Gilbert, Lee Graham, Nicole Pacchiarini, Sara Kumziene-Summerhayes, Sarah Taylor, Sophie Jones, Sara Rey, Matthew Bull, Joanne Watkins, Sally Corden, Tom Connor
EPI_ISL_704049	Liverpool Clinical Laboratories	COVID-19 Genomics UK (COG-UK) Consortium	Sam Haldenby, Anita Lucaci, Steve Paterson, Julian Hiscox, Alistair Darby, M Almsaud, A Alrezaihi, Muhannad Alruwaili, Stuart D Armstrong, Jones Benjamin, Eleanor G Bentley, Anu Chawla, Jordan J Clark, Angela Cowell, Richard Eccles, Isabel García-Dorival, Matthew Gemmell, Alessandro Gerada, PKF Gilmore, Richard Gregory, Ximeng Han, Catherine Hartley, Margaret Hughes, Miren Iturriza-Gomara, James Johnson, L Luu, Jenifer Manson, Charlotte Nelson, Elaine O'Toole, Cassie Olateju, Rebekah Penrice-Randal , Lucille Rainbow, N.P Randle, Trevor Ian Robinson, Parul Sharma, Ghada T Shawli, James P Stewart, Neil Swainston, Ecaterina Vamos, Joanne Watts, Mark Whitehead
EPI_ISL_704086	University College London, Great Ormond Street Hospital for Children NHS Foundation Trust, Imperial College Healthcare NHS Trust	COVID-19 Genomics UK (COG-UK) Consortium	Sergi Castellano, Rachel Williams, Mark Kristiansen, Paola Resende Silva, Sunando Roy, Tony Brooks, Helena Tutill, Paola Niola, Patricia Dyal, Charlotte Williams, Leysa Forrest, Yasmin Panchbhaya, Jacqueline Findlay, Samuel Weeks, Julianne Brown, Kathryn Harris, Paul Randell, James Price, Alison Holmes, Judith Breuer
EPI_ISL_704111, EPI_ISL_704161	Wales Specialist Virology Centre Sequencing lab: Pathogen Genomics Unit	COVID-19 Genomics UK (COG-UK) Consortium	Catherine Moore, Johnathan Evans, Laura Gifford, Malorie Perry, Simon Cottrell, Angela Marchbank, Alec Birchley, Alexander Adams, Amy Gaskin, Bree Gatica-Wilcox, Jason Coombes, Joel Southgate, Lauren Gilbert, Lee Graham, Nicole Pacchiarini, Sara Kumziene-Summerhayes, Sarah Taylor, Sophie Jones, Sara Rey, Matthew Bull, Joanne Watkins, Sally Corden, Tom Connor
EPI_ISL_704179	University College London, Great Ormond Street Hospital for Children NHS Foundation Trust, Imperial College Healthcare NHS Trust	COVID-19 Genomics UK (COG-UK) Consortium	Sergi Castellano, Rachel Williams, Mark Kristiansen, Paola Resende Silva, Sunando Roy, Tony Brooks, Helena Tutill, Paola Niola, Patricia Dyal, Charlotte Williams, Leysa Forrest, Yasmin Panchbhaya, Jacqueline Findlay, Samuel Weeks, Julianne Brown, Kathryn Harris, Paul Randell, James Price, Alison Holmes, Judith Breuer
EPI_ISL_704209	Liverpool Clinical Laboratories	COVID-19 Genomics UK (COG-UK) Consortium	Sam Haldenby, Anita Lucaci, Steve Paterson, Julian Hiscox, Alistair Darby, M Almsaud, A Alrezaihi, Muhannad Alruwaili, Stuart D Armstrong, Jones Benjamin, Eleanor G Bentley, Anu Chawla, Jordan J Clark, Angela Cowell, Richard Eccles, Isabel García-Dorival, Matthew Gemmell, Alessandro Gerada, PKF Gilmore, Richard Gregory, Ximeng Han, Catherine Hartley, Margaret Hughes, Miren Iturriza-Gomara, James Johnson, L Luu, Jenifer Manson, Charlotte Nelson, Elaine O'Toole, Cassie Olateju, Rebekah Penrice-Randal , Lucille Rainbow, N.P Randle, Trevor Ian Robinson, Parul Sharma, Ghada T Shawli, James P Stewart, Neil Swainston, Ecaterina Vamos, Joanne Watts, Mark Whitehead
EPI_ISL_704312, EPI_ISL_704372	University College London, Great Ormond Street Hospital for Children NHS Foundation Trust, Imperial College Healthcare NHS Trust	COVID-19 Genomics UK (COG-UK) Consortium	Sergi Castellano, Rachel Williams, Mark Kristiansen, Paola Resende Silva, Sunando Roy, Tony Brooks, Helena Tutill, Paola Niola, Patricia Dyal, Charlotte Williams, Leysa Forrest, Yasmin Panchbhaya, Jacqueline Findlay, Samuel Weeks, Julianne Brown, Kathryn Harris, Paul Randell, James Price, Alison Holmes, Judith Breuer
EPI_ISL_704382, EPI_ISL_704502, EPI_ISL_704505, EPI_ISL_704507	Department of Pathology, University of Cambridge	COVID-19 Genomics UK (COG-UK) Consortium	Aminu S. Jahun, Yasmin Chaudhry, Grant Hall, Iliana Georgana, Myra Hosmillo, Martin D. Curran, Malte Pinckert, Surendra Parmar, Ian Goodfellow
EPI_ISL_704572, EPI_ISL_704573, EPI_ISL_704574, EPI_ISL_704578	Northumbria University / South Tees Hospitals NHS Foundation Trust / North Cumbria Integrated Care NHS Foundation Trust / North Tees and Hartlepool NHS Foundation Trust / Newcastle Hospitals NHS Foundation Trust	COVID-19 Genomics UK (COG-UK) Consortium	Darren L Smith,Andrew Nelson,Matthew Bashton,Greg R Young,Joshua Loh,John Allan,Mohammad A Tariq,Giles S Holt,Gary Black,Wen C Yew,Lynn Dover,Paul Baker,Steve Liggett,Sarah Essex,Jane Greenaway,Debra Padgett,Clive Graham,Garren Scott,Edward Barton,Emma Swindells,Brendan Payne,Jennifer Collins,Yusri Taha,Gary Eltringham
EPI_ISL_704584	Wales Specialist Virology Centre Sequencing lab: Pathogen Genomics Unit	COVID-19 Genomics UK (COG-UK) Consortium	Catherine Moore, Johnathan Evans, Laura Gifford, Malorie Perry, Simon Cottrell, Angela Marchbank, Alec Birchley, Alexander Adams, Amy Gaskin, Bree Gatica-Wilcox, Jason Coombes, Joel Southgate, Lauren Gilbert, Lee Graham, Nicole Pacchiarini, Sara Kumziene-Summerhayes, Sarah Taylor, Sophie Jones, Sara Rey, Matthew Bull, Joanne Watkins, Sally Corden, Tom Connor
EPI_ISL_704595	West of Scotland Specialist Virology Centre, NHSGGC / MRC-University of Glasgow Centre for Virus Research	COVID-19 Genomics UK (COG-UK) Consortium	Ana da Silva Filipe, Natasha Johnson, Kathy Smollett, Daniel Mair, Stephen Carmichael, Alice Broos, Lily Tong, Jenna Nichols, Kyriaki Nomikou; Sarah McDonald; Richard Orton, Joseph Hughes, Sreenu Vattipally, David L Robertson; Alasdair MacLean, Rory Gunson; Sharif Shaaban, Matthew Holden; Rachel Blacow, Guy Mollett, Kathy Li, James Shepherd, Antonia Ho, Emma Thomson
EPI_ISL_704605	Virology Department, Sheffield Teaching Hospitals NHS Foundation Trust/Department of Infection, Immunity and Cardiovascular Disease, The Medical School, University of Sheffield	COVID-19 Genomics UK (COG-UK) Consortium	Thushan de Silva, Matthew Parker, Nikki Smith, Adri Angyal, Rebecca Brown, Luke Green, Rachel Tucker, Paul Parsons, Danielle Groves, Katie Johnson, Laura Carrilero, Alex Keeley, Dave Partridge, Matthew Wyles, Benjamin Lindsey, Mehmet Yavuz, Mohammad Raza, Cariad Evans
EPI_ISL_704628	Quadram Institute Bioscience	COVID-19 Genomics UK (COG-UK) Consortium	Dave J. Baker, Gemma L. Kay, Alp Aydin, Thanh Le-Viet, Steven Rudder, Ana P. Tedim, Anastasia Kolyva, Maria Diaz, Leonardo de Oliveira Martins, Nabil-Fareed Alikhan, Lizzie Meadows, Rachael Stanley, Ngozi Elumogo, Muhammed Yasir, Nicholas M. Thomson, Alexander J Trotter, Rachel Gilroy, Samuel Bloomfield, Claire Stuart, Andrew Bell, Reenesh Prakash, Samir Dervisevic, Alison E. Mather, John Wain, Mark Webber, Andrew J. Page, Justin O'Grady

EPI_ISL_704750, EPI_ISL_704867	Lighthouse Lab in Cambridge	Wellcome Sanger Institute for the COVID-19 Genomics UK (COG-UK) Consortium	Rob Howes, The Lighthouse Lab in Cambridge and Alex Alderton, Roberto Amato, Sonia Goncalves, Ewan Harrison, David K. Jackson, Ian Johnston, Dominic Kwiatkowski, Cordelia Langford, John Sillitoe on behalf of the Wellcome Sanger Institute COVID-19 Surveillance Team
EPI_ISL_704905	Liverpool Clinical Laboratories	COVID-19 Genomics UK (COG-UK) Consortium	Sam Haldenby, Anita Lucaci, Steve Paterson, Julian Hiscox, Alistair Darby, M Almsaud, A Alrezaihi, Muhannad Alruwaili, Stuart D Armstrong, Jones Benjamin, Eleanor G Bentley, Anu Chawla, Jordan J Clark, Angela Cowell, Richard Eccles, Isabel García-Dorival, Matthew Gemmell, Alessandro Gerada, PKF Gilmore, Richard Gregory, Ximeng Han, Catherine Hartley, Margaret Hughes, Miren Iturriza-Gomara, James Johnson, L Luu, Jenifer Manson, Charlotte Nelson, Elaine O'Toole, Cassie Olateju, Rebekah Penrice-Randal , Lucille Rainbow, N.P Randle, Trevor Ian Robinson, Parul Sharma, Ghada T Shawli, James P Stewart, Neil Swainston, Ecaterina Vamos, Joanne Watts, Mark Whitehead
EPI_ISL_704931	Wales Specialist Virology Centre Sequencing lab: Pathogen Genomics Unit	COVID-19 Genomics UK (COG-UK) Consortium	Catherine Moore, Johnathan Evans, Laura Gifford, Malorie Perry, Simon Cottrell, Angela Marchbank, Alec Birchley, Alexander Adams, Amy Gaskin, Bree Gatica-Wilcox, Jason Coombes, Joel Southgate, Lauren Gilbert, Lee Graham, Nicole Pacchiarini, Sara Kumziene-Summerhayes, Sarah Taylor, Sophie Jones, Sara Rey, Matthew Bull, Joanne Watkins, Sally Corden, Tom Connor
EPI_ISL_704932	Lighthouse Lab in Cambridge	Wellcome Sanger Institute for the COVID-19 Genomics UK (COG-UK) Consortium	Rob Howes, The Lighthouse Lab in Cambridge and Alex Alderton, Roberto Amato, Sonia Goncalves, Ewan Harrison, David K. Jackson, Ian Johnston, Dominic Kwiatkowski, Cordelia Langford, John Sillitoe on behalf of the Wellcome Sanger Institute COVID-19 Surveillance Team
EPI_ISL_704950	Liverpool Clinical Laboratories	COVID-19 Genomics UK (COG-UK) Consortium	Sam Haldenby, Anita Lucaci, Steve Paterson, Julian Hiscox, Alistair Darby, M Almsaud, A Alrezaihi, Muhannad Alruwaili, Stuart D Armstrong, Jones Benjamin, Eleanor G Bentley, Anu Chawla, Jordan J Clark, Angela Cowell, Richard Eccles, Isabel García-Dorival, Matthew Gemmell, Alessandro Gerada, PKF Gilmore, Richard Gregory, Ximeng Han, Catherine Hartley, Margaret Hughes, Miren Iturriza-Gomara, James Johnson, L Luu, Jenifer Manson, Charlotte Nelson, Elaine O'Toole, Cassie Olateju, Rebekah Penrice-Randal , Lucille Rainbow, N.P Randle, Trevor Ian Robinson, Parul Sharma, Ghada T Shawli, James P Stewart, Neil Swainston, Ecaterina Vamos, Joanne Watts, Mark Whitehead
EPI_ISL_704955, EPI_ISL_704965	University College London, Great Ormond Street Hospital for Children NHS Foundation Trust, Imperial College Healthcare NHS Trust	COVID-19 Genomics UK (COG-UK) Consortium	Sergi Castellano, Rachel Williams, Mark Kristiansen, Paola Resende Silva, Sunando Roy, Tony Brooks, Helena Tutill, Paola Niola, Patricia Dyal, Charlotte Williams, Leysa Forrest, Yasmin Panchbhaya, Jacqueline Findlay, Samuel Weeks, Julianne Brown, Kathryn Harris, Paul Randell, James Price, Alison Holmes, Judith Breuer
EPI_ISL_704968, EPI_ISL_704973	Liverpool Clinical Laboratories	COVID-19 Genomics UK (COG-UK) Consortium	Sam Haldenby, Anita Lucaci, Steve Paterson, Julian Hiscox, Alistair Darby, M Almsaud, A Alrezaihi, Muhannad Alruwaili, Stuart D Armstrong, Jones Benjamin, Eleanor G Bentley, Anu Chawla, Jordan J Clark, Angela Cowell, Richard Eccles, Isabel García-Dorival, Matthew Gemmell, Alessandro Gerada, PKF Gilmore, Richard Gregory, Ximeng Han, Catherine Hartley, Margaret Hughes, Miren Iturriza-Gomara, James Johnson, L Luu, Jenifer Manson, Charlotte Nelson, Elaine O'Toole, Cassie Olateju, Rebekah Penrice-Randal , Lucille Rainbow, N.P Randle, Trevor Ian Robinson, Parul Sharma, Ghada T Shawli, James P Stewart, Neil Swainston, Ecaterina Vamos, Joanne Watts, Mark Whitehead
EPI_ISL_705010	Northumbria University / South Tees Hospitals NHS Foundation Trust / North Cumbria Integrated Care NHS Foundation Trust / North Tees and Hartlepool NHS Foundation Trust / Newcastle Hospitals NHS Foundation Trust	COVID-19 Genomics UK (COG-UK) Consortium	Darren L Smith,Andrew Nelson,Matthew Bashton,Greg R Young,Joshua Loh,John Allan,Mohammad A Tariq,Giles S Holt,Gary Black,Wen C Yew,Lynn Dover,Paul Baker,Steve Liggett,Sarah Essex,Jane Greenaway,Debra Padgett,Clive Graham,Garren Scott,Edward Barton,Emma Swindells,Brendan Payne,Jennifer Collins,Yusri Taha,Gary Eltringham
EPI_ISL_705054	Liverpool Clinical Laboratories	COVID-19 Genomics UK (COG-UK) Consortium	Sam Haldenby, Anita Lucaci, Steve Paterson, Julian Hiscox, Alistair Darby, M Almsaud, A Alrezaihi, Muhannad Alruwaili, Stuart D Armstrong, Jones Benjamin, Eleanor G Bentley, Anu Chawla, Jordan J Clark, Angela Cowell, Richard Eccles, Isabel García-Dorival, Matthew Gemmell, Alessandro Gerada, PKF Gilmore, Richard Gregory, Ximeng Han, Catherine Hartley, Margaret Hughes, Miren Iturriza-Gomara, James Johnson, L Luu, Jenifer Manson, Charlotte Nelson, Elaine O'Toole, Cassie Olateju, Rebekah Penrice-Randal , Lucille Rainbow, N.P Randle, Trevor Ian Robinson, Parul Sharma, Ghada T Shawli, James P Stewart, Neil Swainston, Ecaterina Vamos, Joanne Watts, Mark Whitehead
EPI_ISL_705061	Lighthouse Lab in Cambridge	Wellcome Sanger Institute for the COVID-19 Genomics UK (COG-UK) Consortium	Rob Howes, The Lighthouse Lab in Cambridge and Alex Alderton, Roberto Amato, Sonia Goncalves, Ewan Harrison, David K. Jackson, Ian Johnston, Dominic Kwiatkowski, Cordelia Langford, John Sillitoe on behalf of the Wellcome Sanger Institute COVID-19 Surveillance Team
EPI_ISL_705086	Liverpool Clinical Laboratories	COVID-19 Genomics UK (COG-UK) Consortium	Sam Haldenby, Anita Lucaci, Steve Paterson, Julian Hiscox, Alistair Darby, M Almsaud, A Alrezaihi, Muhannad Alruwaili, Stuart D Armstrong, Jones Benjamin, Eleanor G Bentley, Anu Chawla, Jordan J Clark, Angela Cowell, Richard Eccles, Isabel García-Dorival, Matthew Gemmell, Alessandro Gerada, PKF Gilmore, Richard Gregory, Ximeng Han, Catherine Hartley, Margaret Hughes, Miren Iturriza-Gomara, James Johnson, L Luu, Jenifer Manson, Charlotte Nelson, Elaine O'Toole, Cassie Olateju, Rebekah Penrice-Randal , Lucille Rainbow, N.P Randle, Trevor Ian Robinson, Parul Sharma, Ghada T Shawli, James P Stewart, Neil Swainston, Ecaterina Vamos, Joanne Watts, Mark Whitehead
EPI_ISL_705104	Lighthouse Lab in Cambridge	Wellcome Sanger Institute for the COVID-19 Genomics UK (COG-UK) Consortium	Rob Howes, The Lighthouse Lab in Cambridge and Alex Alderton, Roberto Amato, Sonia Goncalves, Ewan Harrison, David K. Jackson, Ian Johnston, Dominic Kwiatkowski, Cordelia Langford, John Sillitoe on behalf of the Wellcome Sanger Institute COVID-19 Surveillance Team
EPI_ISL_705137	Northumbria University / South Tees Hospitals NHS Foundation Trust / North Cumbria Integrated Care NHS Foundation Trust / North Tees and Hartlepool NHS Foundation Trust / Newcastle Hospitals NHS Foundation Trust	COVID-19 Genomics UK (COG-UK) Consortium	Darren L Smith,Andrew Nelson,Matthew Bashton,Greg R Young,Joshua Loh,John Allan,Mohammad A Tariq,Giles S Holt,Gary Black,Wen C Yew,Lynn Dover,Paul Baker,Steve Liggett,Sarah Essex,Jane Greenaway,Debra Padgett,Clive Graham,Garren Scott,Edward Barton,Emma Swindells,Brendan Payne,Jennifer Collins,Yusri Taha,Gary Eltringham
EPI_ISL_705143	University College London, Great Ormond Street Hospital for Children NHS Foundation Trust, Imperial College Healthcare NHS Trust	COVID-19 Genomics UK (COG-UK) Consortium	Sergi Castellano, Rachel Williams, Mark Kristiansen, Paola Resende Silva, Sunando Roy, Tony Brooks, Helena Tutill, Paola Niola, Patricia Dyal, Charlotte Williams, Leysa Forrest, Yasmin Panchbhaya, Jacqueline Findlay, Samuel Weeks, Julianne Brown, Kathryn Harris, Paul Randell, James Price, Alison Holmes, Judith Breuer
EPI_ISL_705148	Northumbria University / South Tees Hospitals NHS Foundation Trust / North Cumbria Integrated Care NHS Foundation Trust / North Tees and Hartlepool NHS Foundation Trust / Newcastle Hospitals NHS Foundation Trust	COVID-19 Genomics UK (COG-UK) Consortium	Darren L Smith,Andrew Nelson,Matthew Bashton,Greg R Young,Joshua Loh,John Allan,Mohammad A Tariq,Giles S Holt,Gary Black,Wen C Yew,Lynn Dover,Paul Baker,Steve Liggett,Sarah Essex,Jane Greenaway,Debra Padgett,Clive Graham,Garren Scott,Edward Barton,Emma Swindells,Brendan Payne,Jennifer Collins,Yusri Taha,Gary Eltringham
EPI_ISL_705168, EPI_ISL_705176	Liverpool Clinical Laboratories	COVID-19 Genomics UK (COG-UK) Consortium	Sam Haldenby, Anita Lucaci, Steve Paterson, Julian Hiscox, Alistair Darby, M Almsaud, A Alrezaihi, Muhannad Alruwaili, Stuart D Armstrong, Jones Benjamin, Eleanor G Bentley, Anu Chawla, Jordan J Clark, Angela Cowell, Richard Eccles, Isabel García-Dorival, Matthew Gemmell, Alessandro Gerada, PKF Gilmore, Richard Gregory, Ximeng Han, Catherine Hartley, Margaret Hughes, Miren Iturriza-Gomara, James Johnson, L Luu, Jenifer Manson, Charlotte Nelson, Elaine O'Toole, Cassie Olateju, Rebekah Penrice-Randal , Lucille Rainbow, N.P Randle, Trevor Ian Robinson, Parul Sharma, Ghada T Shawli, James P Stewart, Neil Swainston, Ecaterina Vamos, Joanne Watts, Mark Whitehead
EPI_ISL_705191, EPI_ISL_705192	Lighthouse Lab in Milton Keynes	Wellcome Sanger Institute for the COVID-19 Genomics UK (COG-UK) Consortium	The Lighthouse Lab in Milton Keynes and Alex Alderton, Roberto Amato, Sonia Goncalves, Ewan Harrison, David K. Jackson, Ian Johnston, Dominic Kwiatkowski, Cordelia Langford, John Sillitoe on behalf of the Wellcome Sanger Institute COVID-19 Surveillance Team
EPI_ISL_705193	Liverpool Clinical Laboratories	COVID-19 Genomics UK (COG-UK) Consortium	Sam Haldenby, Anita Lucaci, Steve Paterson, Julian Hiscox, Alistair Darby, M Almsaud, A Alrezaihi, Muhannad Alruwaili, Stuart D Armstrong, Jones Benjamin, Eleanor G Bentley, Anu Chawla, Jordan J Clark, Angela Cowell, Richard Eccles, Isabel García-Dorival, Matthew Gemmell, Alessandro Gerada, PKF Gilmore, Richard Gregory, Ximeng Han, Catherine Hartley, Margaret Hughes, Miren Iturriza-Gomara, James Johnson, L Luu, Jenifer Manson, Charlotte Nelson, Elaine O'Toole, Cassie Olateju, Rebekah Penrice-Randal , Lucille Rainbow, N.P Randle, Trevor Ian Robinson, Parul Sharma, Ghada T Shawli, James P Stewart, Neil Swainston, Ecaterina Vamos, Joanne Watts, Mark Whitehead
EPI_ISL_705194, EPI_ISL_705196, EPI_ISL_705197, EPI_ISL_705199, EPI_ISL_705200, EPI_ISL_705202	Lighthouse Lab in Milton Keynes	Wellcome Sanger Institute for the COVID-19 Genomics UK (COG-UK) Consortium	The Lighthouse Lab in Milton Keynes and Alex Alderton, Roberto Amato, Sonia Goncalves, Ewan Harrison, David K. Jackson, Ian Johnston, Dominic Kwiatkowski, Cordelia Langford, John Sillitoe on behalf of the Wellcome Sanger Institute COVID-19 Surveillance Team
EPI_ISL_705203, EPI_ISL_705208	Liverpool Clinical Laboratories	COVID-19 Genomics UK (COG-UK) Consortium	Sam Haldenby, Anita Lucaci, Steve Paterson, Julian Hiscox, Alistair Darby, M Almsaud, A Alrezaihi, Muhannad Alruwaili, Stuart D Armstrong, Jones Benjamin, Eleanor G Bentley, Anu Chawla, Jordan J Clark, Angela Cowell, Richard Eccles, Isabel García-Dorival, Matthew Gemmell, Alessandro Gerada, PKF Gilmore, Richard Gregory, Ximeng Han, Catherine Hartley, Margaret Hughes, Miren Iturriza-Gomara, James Johnson, L Luu, Jenifer Manson, Charlotte Nelson, Elaine O'Toole, Cassie Olateju, Rebekah Penrice-Randal , Lucille Rainbow, N.P Randle, Trevor Ian Robinson, Parul Sharma, Ghada T Shawli, James P Stewart, Neil Swainston, Ecaterina Vamos, Joanne Watts, Mark Whitehead
EPI_ISL_705209	University College London, Great Ormond Street Hospital for Children NHS Foundation Trust, Imperial College Healthcare NHS Trust	COVID-19 Genomics UK (COG-UK) Consortium	Sergi Castellano, Rachel Williams, Mark Kristiansen, Paola Resende Silva, Sunando Roy, Tony Brooks, Helena Tutill, Paola Niola, Patricia Dyal, Charlotte Williams, Leysa Forrest, Yasmin Panchbhaya, Jacqueline Findlay, Samuel Weeks, Julianne Brown, Kathryn Harris, Paul Randell, James Price, Alison Holmes, Judith Breuer

EPI_ISL_705214	Northumbria University / South Tees Hospitals NHS Foundation Trust / North Cumbria Integrated Care NHS Foundation Trust / North Tees and Hartlepool NHS Foundation Trust / Newcastle Hospitals NHS Foundation Trust	COVID-19 Genomics UK (COG-UK) Consortium	Darren L Smith,Andrew Nelson,Matthew Bashton,Greg R Young,Joshua Loh,John Allan,Mohammad A Tariq,Giles S Holt,Gary Black,Wen C Yew,Lynn Dover,Paul Baker,Steve Liggett,Sarah Essex,Jane Greenaway,Debra Padgett,Clive Graham,Garren Scott,Edward Barton,Emma Swindells,Brendan Payne,Jennifer Collins,Yusri Taha,Gary Eltringham
EPI_ISL_705231	Liverpool Clinical Laboratories	COVID-19 Genomics UK (COG-UK) Consortium	Sam Haldenby, Anita Lucaci, Steve Paterson, Julian Hiscox, Alistair Darby, M Almsaud, A Alrezaihi, Muhannad Alruwaili, Stuart D Armstrong, Jones Benjamin, Eleanor G Bentley, Anu Chawla, Jordan J Clark, Angela Cowell, Richard Eccles, Isabel García-Dorival, Matthew Gemmell, Alessandro Gerada, PKF Gilmore, Richard Gregory, Ximeng Han, Catherine Hartley, Margaret Hughes, Miren Iturriza-Gomara, James Johnson, L Luu, Jenifer Manson, Charlotte Nelson, Elaine O'Toole, Cassie Olateju, Rebekah Penrice-Randal , Lucille Rainbow, N.P Randle, Trevor Ian Robinson, Parul Sharma, Ghada T Shawli, James P Stewart, Neil Swainston, Ecaterina Vamos, Joanne Watts, Mark Whitehead
EPI_ISL_705239, EPI_ISL_705244	Wales Specialist Virology Centre Sequencing lab: Pathogen Genomics Unit	COVID-19 Genomics UK (COG-UK) Consortium	Catherine Moore, Johnathan Evans, Laura Gifford, Malorie Perry, Simon Cottrell, Angela Marchbank, Alec Birchley, Alexander Adams, Amy Gaskin, Bree Gatica-Wilcox, Jason Coombes, Joel Southgate, Lauren Gilbert, Lee Graham, Nicole Pacchiarini, Sara Kumziene-Summerhayes, Sarah Taylor, Sophie Jones, Sara Rey, Matthew Bull, Joanne Watkins, Sally Corden, Tom Connor
EPI_ISL_705266, EPI_ISL_705282	Northumbria University / South Tees Hospitals NHS Foundation Trust / North Cumbria Integrated Care NHS Foundation Trust / North Tees and Hartlepool NHS Foundation Trust / Newcastle Hospitals NHS Foundation Trust	COVID-19 Genomics UK (COG-UK) Consortium	Darren L Smith,Andrew Nelson,Matthew Bashton,Greg R Young,Joshua Loh,John Allan,Mohammad A Tariq,Giles S Holt,Gary Black,Wen C Yew,Lynn Dover,Paul Baker,Steve Liggett,Sarah Essex,Jane Greenaway,Debra Padgett,Clive Graham,Garren Scott,Edward Barton,Emma Swindells,Brendan Payne,Jennifer Collins,Yusri Taha,Gary Eltringham
EPI_ISL_705287	Liverpool Clinical Laboratories	COVID-19 Genomics UK (COG-UK) Consortium	Sam Haldenby, Anita Lucaci, Steve Paterson, Julian Hiscox, Alistair Darby, M Almsaud, A Alrezaihi, Muhannad Alruwaili, Stuart D Armstrong, Jones Benjamin, Eleanor G Bentley, Anu Chawla, Jordan J Clark, Angela Cowell, Richard Eccles, Isabel García-Dorival, Matthew Gemmell, Alessandro Gerada, PKF Gilmore, Richard Gregory, Ximeng Han, Catherine Hartley, Margaret Hughes, Miren Iturriza-Gomara, James Johnson, L Luu, Jenifer Manson, Charlotte Nelson, Elaine O'Toole, Cassie Olateju, Rebekah Penrice-Randal , Lucille Rainbow, N.P Randle, Trevor Ian Robinson, Parul Sharma, Ghada T Shawli, James P Stewart, Neil Swainston, Ecaterina Vamos, Joanne Watts, Mark Whitehead
EPI_ISL_705295	Northumbria University / South Tees Hospitals NHS Foundation Trust / North Cumbria Integrated Care NHS Foundation Trust / North Tees and Hartlepool NHS Foundation Trust / Newcastle Hospitals NHS Foundation Trust	COVID-19 Genomics UK (COG-UK) Consortium	Darren L Smith,Andrew Nelson,Matthew Bashton,Greg R Young,Joshua Loh,John Allan,Mohammad A Tariq,Giles S Holt,Gary Black,Wen C Yew,Lynn Dover,Paul Baker,Steve Liggett,Sarah Essex,Jane Greenaway,Debra Padgett,Clive Graham,Garren Scott,Edward Barton,Emma Swindells,Brendan Payne,Jennifer Collins,Yusri Taha,Gary Eltringham
EPI_ISL_705310	University College London, Great Ormond Street Hospital for Children NHS Foundation Trust, Imperial College Healthcare NHS Trust	COVID-19 Genomics UK (COG-UK) Consortium	Sergi Castellano, Rachel Williams, Mark Kristiansen, Paola Resende Silva, Sunando Roy, Tony Brooks, Helena Tutill, Paola Niola, Patricia Dyal, Charlotte Williams, Leysa Forrest, Yasmin Panchbhaya, Jacqueline Findlay, Samuel Weeks, Julianne Brown, Kathryn Harris, Paul Randell, James Price, Alison Holmes, Judith Breuer
EPI_ISL_705329	Liverpool Clinical Laboratories	COVID-19 Genomics UK (COG-UK) Consortium	Sam Haldenby, Anita Lucaci, Steve Paterson, Julian Hiscox, Alistair Darby, M Almsaud, A Alrezaihi, Muhannad Alruwaili, Stuart D Armstrong, Jones Benjamin, Eleanor G Bentley, Anu Chawla, Jordan J Clark, Angela Cowell, Richard Eccles, Isabel García-Dorival, Matthew Gemmell, Alessandro Gerada, PKF Gilmore, Richard Gregory, Ximeng Han, Catherine Hartley, Margaret Hughes, Miren Iturriza-Gomara, James Johnson, L Luu, Jenifer Manson, Charlotte Nelson, Elaine O'Toole, Cassie Olateju, Rebekah Penrice-Randal , Lucille Rainbow, N.P Randle, Trevor Ian Robinson, Parul Sharma, Ghada T Shawli, James P Stewart, Neil Swainston, Ecaterina Vamos, Joanne Watts, Mark Whitehead
EPI_ISL_705342	University College London, Great Ormond Street Hospital for Children NHS Foundation Trust, Imperial College Healthcare NHS Trust	COVID-19 Genomics UK (COG-UK) Consortium	Sergi Castellano, Rachel Williams, Mark Kristiansen, Paola Resende Silva, Sunando Roy, Tony Brooks, Helena Tutill, Paola Niola, Patricia Dyal, Charlotte Williams, Leysa Forrest, Yasmin Panchbhaya, Jacqueline Findlay, Samuel Weeks, Julianne Brown, Kathryn Harris, Paul Randell, James Price, Alison Holmes, Judith Breuer
EPI_ISL_705343, EPI_ISL_705353	Northumbria University / South Tees Hospitals NHS Foundation Trust / North Cumbria Integrated Care NHS Foundation Trust / North Tees and Hartlepool NHS Foundation Trust / Newcastle Hospitals NHS Foundation Trust	COVID-19 Genomics UK (COG-UK) Consortium	Darren L Smith,Andrew Nelson,Matthew Bashton,Greg R Young,Joshua Loh,John Allan,Mohammad A Tariq,Giles S Holt,Gary Black,Wen C Yew,Lynn Dover,Paul Baker,Steve Liggett,Sarah Essex,Jane Greenaway,Debra Padgett,Clive Graham,Garren Scott,Edward Barton,Emma Swindells,Brendan Payne,Jennifer Collins,Yusri Taha,Gary Eltringham
EPI_ISL_705358	Liverpool Clinical Laboratories	COVID-19 Genomics UK (COG-UK) Consortium	Sam Haldenby, Anita Lucaci, Steve Paterson, Julian Hiscox, Alistair Darby, M Almsaud, A Alrezaihi, Muhannad Alruwaili, Stuart D Armstrong, Jones Benjamin, Eleanor G Bentley, Anu Chawla, Jordan J Clark, Angela Cowell, Richard Eccles, Isabel García-Dorival, Matthew Gemmell, Alessandro Gerada, PKF Gilmore, Richard Gregory, Ximeng Han, Catherine Hartley, Margaret Hughes, Miren Iturriza-Gomara, James Johnson, L Luu, Jenifer Manson, Charlotte Nelson, Elaine O'Toole, Cassie Olateju, Rebekah Penrice-Randal , Lucille Rainbow, N.P Randle, Trevor Ian Robinson, Parul Sharma, Ghada T Shawli, James P Stewart, Neil Swainston, Ecaterina Vamos, Joanne Watts, Mark Whitehead
EPI_ISL_705364, EPI_ISL_705367	Northumbria University / South Tees Hospitals NHS Foundation Trust / North Cumbria Integrated Care NHS Foundation Trust / North Tees and Hartlepool NHS Foundation Trust / Newcastle Hospitals NHS Foundation Trust	COVID-19 Genomics UK (COG-UK) Consortium	Darren L Smith,Andrew Nelson,Matthew Bashton,Greg R Young,Joshua Loh,John Allan,Mohammad A Tariq,Giles S Holt,Gary Black,Wen C Yew,Lynn Dover,Paul Baker,Steve Liggett,Sarah Essex,Jane Greenaway,Debra Padgett,Clive Graham,Garren Scott,Edward Barton,Emma Swindells,Brendan Payne,Jennifer Collins,Yusri Taha,Gary Eltringham
EPI_ISL_705384	Wales Specialist Virology Centre Sequencing lab: Pathogen Genomics Unit	COVID-19 Genomics UK (COG-UK) Consortium	Catherine Moore, Johnathan Evans, Laura Gifford, Malorie Perry, Simon Cottrell, Angela Marchbank, Alec Birchley, Alexander Adams, Amy Gaskin, Bree Gatica-Wilcox, Jason Coombes, Joel Southgate, Lauren Gilbert, Lee Graham, Nicole Pacchiarini, Sara Kumziene-Summerhayes, Sarah Taylor, Sophie Jones, Sara Rey, Matthew Bull, Joanne Watkins, Sally Corden, Tom Connor
EPI_ISL_705388	Liverpool Clinical Laboratories	COVID-19 Genomics UK (COG-UK) Consortium	Sam Haldenby, Anita Lucaci, Steve Paterson, Julian Hiscox, Alistair Darby, M Almsaud, A Alrezaihi, Muhannad Alruwaili, Stuart D Armstrong, Jones Benjamin, Eleanor G Bentley, Anu Chawla, Jordan J Clark, Angela Cowell, Richard Eccles, Isabel García-Dorival, Matthew Gemmell, Alessandro Gerada, PKF Gilmore, Richard Gregory, Ximeng Han, Catherine Hartley, Margaret Hughes, Miren Iturriza-Gomara, James Johnson, L Luu, Jenifer Manson, Charlotte Nelson, Elaine O'Toole, Cassie Olateju, Rebekah Penrice-Randal , Lucille Rainbow, N.P Randle, Trevor Ian Robinson, Parul Sharma, Ghada T Shawli, James P Stewart, Neil Swainston, Ecaterina Vamos, Joanne Watts, Mark Whitehead
EPI_ISL_705397	University College London, Great Ormond Street Hospital for Children NHS Foundation Trust, Imperial College Healthcare NHS Trust	COVID-19 Genomics UK (COG-UK) Consortium	Sergi Castellano, Rachel Williams, Mark Kristiansen, Paola Resende Silva, Sunando Roy, Tony Brooks, Helena Tutill, Paola Niola, Patricia Dyal, Charlotte Williams, Leysa Forrest, Yasmin Panchbhaya, Jacqueline Findlay, Samuel Weeks, Julianne Brown, Kathryn Harris, Paul Randell, James Price, Alison Holmes, Judith Breuer
EPI_ISL_705412	Wales Specialist Virology Centre Sequencing lab: Pathogen Genomics Unit	COVID-19 Genomics UK (COG-UK) Consortium	Catherine Moore, Johnathan Evans, Laura Gifford, Malorie Perry, Simon Cottrell, Angela Marchbank, Alec Birchley, Alexander Adams, Amy Gaskin, Bree Gatica-Wilcox, Jason Coombes, Joel Southgate, Lauren Gilbert, Lee Graham, Nicole Pacchiarini, Sara Kumziene-Summerhayes, Sarah Taylor, Sophie Jones, Sara Rey, Matthew Bull, Joanne Watkins, Sally Corden, Tom Connor
EPI_ISL_705422	Quadram Institute Bioscience	COVID-19 Genomics UK (COG-UK) Consortium	Dave J. Baker, Gemma L. Kay, Alp Aydin, Thanh Le-Viet, Steven Rudder, Ana P. Tedim, Anastasia Kolyva, Maria Diaz, Leonardo de Oliveira Martins, Nabil-Fareed Alikhan, Lizzie Meadows, Rachael Stanley, Ngozi Elumogo, Muhammed Yasir, Nicholas M. Thomson, Alexander J Trotter, Rachel Gilroy, Samuel Bloomfield, Claire Stuart, Andrew Bell, Reenesh Prakash, Samir Dervisevic, Alison E. Mather, John Wain, Mark Webber, Andrew J. Page, Justin O'Grady
EPI_ISL_705435, EPI_ISL_705440, EPI_ISL_705441, EPI_ISL_705442	Wales Specialist Virology Centre Sequencing lab: Pathogen Genomics Unit	COVID-19 Genomics UK (COG-UK) Consortium	Catherine Moore, Johnathan Evans, Laura Gifford, Malorie Perry, Simon Cottrell, Angela Marchbank, Alec Birchley, Alexander Adams, Amy Gaskin, Bree Gatica-Wilcox, Jason Coombes, Joel Southgate, Lauren Gilbert, Lee Graham, Nicole Pacchiarini, Sara Kumziene-Summerhayes, Sarah Taylor, Sophie Jones, Sara Rey, Matthew Bull, Joanne Watkins, Sally Corden, Tom Connor
EPI_ISL_705490	West of Scotland Specialist Virology Centre, NHSGGC / MRC-University of Glasgow Centre for Virus Research	COVID-19 Genomics UK (COG-UK) Consortium	Ana da Silva Filipe, Natasha Johnson, Kathy Smollett, Daniel Mair, Stephen Carmichael, Alice Broos, Lily Tong, Jenna Nichols, Kyriaki Nomikou; Sarah McDonald; Richard Orton, Joseph Hughes, Sreenu Vattipally, David L Robertson; Alasdair MacLean, Rory Gunson; Sharif Shaaban, Matthew Holden; Rachel Blacow, Guy Mollett, Kathy Li, James Shepherd, Antonia Ho, Emma Thomson
EPI_ISL_705496	Northumbria University / South Tees Hospitals NHS Foundation Trust / North Cumbria Integrated Care NHS Foundation Trust / North Tees and Hartlepool NHS Foundation Trust / Newcastle Hospitals NHS Foundation Trust	COVID-19 Genomics UK (COG-UK) Consortium	Darren L Smith,Andrew Nelson,Matthew Bashton,Greg R Young,Joshua Loh,John Allan,Mohammad A Tariq,Giles S Holt,Gary Black,Wen C Yew,Lynn Dover,Paul Baker,Steve Liggett,Sarah Essex,Jane Greenaway,Debra Padgett,Clive Graham,Garren Scott,Edward Barton,Emma Swindells,Brendan Payne,Jennifer Collins,Yusri Taha,Gary Eltringham

EPI_ISL_705560	Department of Pathology, University of Cambridge	COVID-19 Genomics UK (COG-UK) Consortium	Aminu S. Jahun, Yasmin Chaudhry, Grant Hall, Iliana Georgana, Myra Hosmillo, Martin D. Curran, Malte Pinckert, Surendra Parmar, Ian Goodfellow
EPI_ISL_705572, EPI_ISL_705593, EPI_ISL_705594	Quadram Institute Bioscience	COVID-19 Genomics UK (COG-UK) Consortium	Dave J. Baker, Gemma L. Kay, Alp Aydin, Thanh Le-Viet, Steven Rudder, Ana P. Tedim, Anastasia Kolyva, Maria Diaz, Leonardo de Oliveira Martins, Nabil-Fareed Alikhan, Lizzie Meadows, Rachael Stanley, Ngozi Elumogo, Muhammed Yasir, Nicholas M. Thomson, Alexander J Trotter, Rachel Gilroy, Samuel Bloomfield, Claire Stuart, Andrew Bell, Reenesh Prakash, Samir Dervisevic, Alison E. Mather, John Wain, Mark Webber, Andrew J. Page, Justin O'Grady
EPI_ISL_705626, EPI_ISL_705627, EPI_ISL_705628	Virology Department, Sheffield Teaching Hospitals NHS Foundation Trust/Department of Infection, Immunity and Cardiovascular Disease, The Medical School, University of Sheffield	COVID-19 Genomics UK (COG-UK) Consortium	Thushan de Silva, Matthew Parker, Nikki Smith, Adri Angyal, Rebecca Brown, Luke Green, Rachel Tucker, Paul Parsons, Danielle Groves, Katie Johnson, Laura Carrilero, Alex Keeley, Dave Partridge, Matthew Wyles, Benjamin Lindsey, Mehmet Yavuz, Mohammad Raza, Cariad Evans
EPI_ISL_705641, EPI_ISL_705642, EPI_ISL_705643, EPI_ISL_705644, EPI_ISL_705645, EPI_ISL_705646, EPI_ISL_705647, EPI_ISL_705648, EPI_ISL_705649, EPI_ISL_705650, EPI_ISL_705651, EPI_ISL_705652, EPI_ISL_705653, EPI_ISL_705654, EPI_ISL_705655, EPI_ISL_705656, EPI_ISL_705657, EPI_ISL_705658, EPI_ISL_705659, EPI_ISL_705660, EPI_ISL_705661, EPI_ISL_705662, EPI_ISL_705663, EPI_ISL_705664, EPI_ISL_705665, EPI_ISL_705666, EPI_ISL_705708, EPI_ISL_705710, EPI_ISL_705730			
see above	West of Scotland Specialist Virology Centre, NHSGGC / MRC-University of Glasgow Centre for Virus Research	COVID-19 Genomics UK (COG-UK) Consortium	Ana da Silva Filipe, Natasha Johnson, Kathy Smollett, Daniel Mair, Stephen Carmichael, Alice Broos, Lily Tong, Jenna Nichols, Kyriaki Nomikou; Sarah McDonald; Richard Orton, Joseph Hughes, Sreenu Vattipally, David L Robertson; Alasdair MacLean, Rory Gunson; Sharif Shaaban, Matthew Holden; Rachel Blacow, Guy Mollett, Kathy Li, James Shepherd, Antonia Ho, Emma Thomson
EPI_ISL_705804	Virology Department, Royal Infirmary of Edinburgh, NHS Lothian / School of Biological Sciences, University of Edinburgh / Institute of Genetics and Molecular Medicine, University of Edinburgh	COVID-19 Genomics UK (COG-UK) Consortium	McHugh M, Dewar R, Rooke S, Gallagher M, Balcaza C, O'Toole Á, Scher E, Hill V, McCrone JT, Colquhoun R, Yu X, Jackson B, Rambaut A, Williams TC, Templeton K
EPI_ISL_705810, EPI_ISL_705812, EPI_ISL_705818, EPI_ISL_705929, EPI_ISL_705930, EPI_ISL_705931, EPI_ISL_705932, EPI_ISL_705933, EPI_ISL_705934, EPI_ISL_705935, EPI_ISL_705936, EPI_ISL_705937, EPI_ISL_705938, EPI_ISL_705939, EPI_ISL_705940			
see above	Liverpool Clinical Laboratories	COVID-19 Genomics UK (COG-UK) Consortium	Sam Haldenby, Anita Lucaci, Steve Paterson, Julian Hiscox, Alistair Darby, M Almsaud, A Alrezaihi, Muhannad Alruwaili, Stuart D Armstrong, Jones Benjamin, Eleanor G Bentley, Anu Chawla, Jordan J Clark, Angela Cowell, Richard Eccles, Isabel García-Dorival, Matthew Gemmell, Alessandro Gerada, PKF Gilmore, Richard Gregory, Ximeng Han, Catherine Hartley, Margaret Hughes, Miren Iturriza-Gomara, James Johnson, L Luu, Jenifer Manson, Charlotte Nelson, Elaine O'Toole, Cassie Olateju, Rebekah Penrice-Randal , Lucille Rainbow, N.P Randle, Trevor Ian Robinson, Parul Sharma, Ghada T Shawli, James P Stewart, Neil Swainston, Ecaterina Vamos, Joanne Watts, Mark Whitehead
EPI_ISL_705949	University College London Hospital	COVID-19 Genomics UK (COG-UK) Consortium	Judith Heaney, Matthew Byott, Catherine Houlihan, Dan Frampton, Stuart Kirk, Moira Spyer and Eleni Nastouli
EPI_ISL_705961, EPI_ISL_705962, EPI_ISL_705963, EPI_ISL_705964, EPI_ISL_705965, EPI_ISL_705966, EPI_ISL_705967, EPI_ISL_705968, EPI_ISL_705969, EPI_ISL_705970, EPI_ISL_705971			
see above	University College London, Great Ormond Street Hospital for Children NHS Foundation Trust, Imperial College Healthcare NHS Trust	COVID-19 Genomics UK (COG-UK) Consortium	Sergi Castellano, Rachel Williams, Mark Kristiansen, Paola Resende Silva, Sunando Roy, Tony Brooks, Helena Tutill, Paola Niola, Patricia Dyal, Charlotte Williams, Leysa Forrest, Yasmin Panchbhaya, Jacqueline Findlay, Samuel Weeks, Julianne Brown, Kathryn Harris, Paul Randell, James Price, Alison Holmes, Judith Breuer
EPI_ISL_706312, EPI_ISL_706313, EPI_ISL_706314, EPI_ISL_706316, EPI_ISL_706317, EPI_ISL_706318, EPI_ISL_706319, EPI_ISL_706322, EPI_ISL_706334, EPI_ISL_706335, EPI_ISL_706336, EPI_ISL_706337, EPI_ISL_706338, EPI_ISL_706339, EPI_ISL_706341, EPI_ISL_706342, EPI_ISL_706343, EPI_ISL_706344, EPI_ISL_706345, EPI_ISL_706346, EPI_ISL_706347, EPI_ISL_706348, EPI_ISL_706349, EPI_ISL_706350, EPI_ISL_706351, EPI_ISL_706352			
see above	Northumbria University / South Tees Hospitals NHS Foundation Trust / North Cumbria Integrated Care NHS Foundation Trust / North Tees and Hartlepool NHS Foundation Trust / Newcastle Hospitals NHS Foundation Trust	COVID-19 Genomics UK (COG-UK) Consortium	Darren L Smith,Andrew Nelson,Matthew Bashton,Greg R Young,Joshua Loh,John Allan,Mohammad A Tariq,Giles S Holt,Gary Black,Wen C Yew,Lynn Dover,Paul Baker,Steve Liggett,Sarah Essex,Jane Greenaway,Debra Padgett,Clive Graham,Garren Scott,Edward Barton,Emma Swindells,Brendan Payne,Jennifer Collins,Yusri Taha,Gary Eltringham
EPI_ISL_706445, EPI_ISL_706449, EPI_ISL_706453, EPI_ISL_706454, EPI_ISL_706455, EPI_ISL_706458, EPI_ISL_706459, EPI_ISL_706460, EPI_ISL_706461, EPI_ISL_706462, EPI_ISL_706527, EPI_ISL_706529, EPI_ISL_706535, EPI_ISL_706538, EPI_ISL_706542, EPI_ISL_706554, EPI_ISL_706555, EPI_ISL_706556, EPI_ISL_706557, EPI_ISL_706558, EPI_ISL_706559, EPI_ISL_706560, EPI_ISL_706561, EPI_ISL_706562, EPI_ISL_706563, EPI_ISL_706564, EPI_ISL_706565, EPI_ISL_706566, EPI_ISL_706567, EPI_ISL_706568, EPI_ISL_706569, EPI_ISL_706570, EPI_ISL_706571, EPI_ISL_706572, EPI_ISL_706573, EPI_ISL_706574, EPI_ISL_706575, EPI_ISL_706576, EPI_ISL_706577, EPI_ISL_706578, EPI_ISL_706579, EPI_ISL_706580, EPI_ISL_706581, EPI_ISL_706582, EPI_ISL_706583, EPI_ISL_706584, EPI_ISL_706585, EPI_ISL_706586, EPI_ISL_706587, EPI_ISL_706588, EPI_ISL_706596, EPI_ISL_706597, EPI_ISL_706598, EPI_ISL_706599, EPI_ISL_706600, EPI_ISL_706601, EPI_ISL_706602, EPI_ISL_706608, EPI_ISL_706609, EPI_ISL_706610, EPI_ISL_706611, EPI_ISL_706612, EPI_ISL_706613, EPI_ISL_706620, EPI_ISL_706621, EPI_ISL_706622, EPI_ISL_706623, EPI_ISL_706624, EPI_ISL_706625, EPI_ISL_706626, EPI_ISL_706630, EPI_ISL_706631, EPI_ISL_706632, EPI_ISL_706633, EPI_ISL_706634, EPI_ISL_706635, EPI_ISL_706639, EPI_ISL_706640, EPI_ISL_706641, EPI_ISL_706642, EPI_ISL_706643, EPI_ISL_706644, EPI_ISL_706645, EPI_ISL_706646, EPI_ISL_706647, EPI_ISL_706648, EPI_ISL_706649, EPI_ISL_706650, EPI_ISL_706651, EPI_ISL_706652, EPI_ISL_706653, EPI_ISL_706654, EPI_ISL_706655, EPI_ISL_706656, EPI_ISL_706657, EPI_ISL_706662, EPI_ISL_706776, EPI_ISL_706783, EPI_ISL_706784, EPI_ISL_706785, EPI_ISL_706786, EPI_ISL_706787, EPI_ISL_706788, EPI_ISL_706789, EPI_ISL_706791, EPI_ISL_706792, EPI_ISL_706793, EPI_ISL_706794, EPI_ISL_706796, EPI_ISL_706797, EPI_ISL_706800, EPI_ISL_706801, EPI_ISL_706802, EPI_ISL_706803, EPI_ISL_706804, EPI_ISL_706805, EPI_ISL_706806, EPI_ISL_706807, EPI_ISL_706808, EPI_ISL_706809, EPI_ISL_706810, EPI_ISL_706811, EPI_ISL_706812, EPI_ISL_706813, EPI_ISL_706814, EPI_ISL_706815, EPI_ISL_706816, EPI_ISL_706817, EPI_ISL_706818, EPI_ISL_706819, EPI_ISL_706820, EPI_ISL_706821, EPI_ISL_706822, EPI_ISL_706823, EPI_ISL_706824, EPI_ISL_706825, EPI_ISL_706826, EPI_ISL_706827, EPI_ISL_706828, EPI_ISL_706829, EPI_ISL_706830, EPI_ISL_706831, EPI_ISL_706832, EPI_ISL_706833, EPI_ISL_706834, EPI_ISL_706835, EPI_ISL_706836, EPI_ISL_706837, EPI_ISL_706838, EPI_ISL_706839, EPI_ISL_706840, EPI_ISL_706841, EPI_ISL_706842, EPI_ISL_706843, EPI_ISL_706844, EPI_ISL_706845, EPI_ISL_706846, EPI_ISL_706847, EPI_ISL_706848, EPI_ISL_706849, EPI_ISL_706850, EPI_ISL_706851, EPI_ISL_706852, EPI_ISL_706853, EPI_ISL_706854, EPI_ISL_706855, EPI_ISL_706856, EPI_ISL_706857, EPI_ISL_706858, EPI_ISL_706859, EPI_ISL_706860, EPI_ISL_706861, EPI_ISL_706862, EPI_ISL_706863			
see above	Wales Specialist Virology Centre Sequencing lab: Pathogen Genomics Unit	COVID-19 Genomics UK (COG-UK) Consortium	Catherine Moore, Johnathan Evans, Laura Gifford, Malorie Perry, Simon Cottrell, Angela Marchbank, Alec Birchley, Alexander Adams, Amy Gaskin, Bree Gatica-Wilcox, Jason Coombes, Joel Southgate, Lauren Gilbert, Lee Graham, Nicole Pacchiarini, Sara Kumziene-Summerhayes, Sarah Taylor, Sophie Jones, Sara Rey, Matthew Bull, Joanne Watkins, Sally Corden, Tom Connor
EPI_ISL_706988	Virology Department, Sheffield Teaching Hospitals NHS Foundation Trust/Department of Infection, Immunity and Cardiovascular Disease, The Medical School, University of Sheffield	COVID-19 Genomics UK (COG-UK) Consortium	Thushan de Silva, Matthew Parker, Nikki Smith, Adri Angyal, Rebecca Brown, Luke Green, Rachel Tucker, Paul Parsons, Danielle Groves, Katie Johnson, Laura Carrilero, Alex Keeley, Dave Partridge, Matthew Wyles, Benjamin Lindsey, Mehmet Yavuz, Mohammad Raza, Cariad Evans
EPI_ISL_708136, EPI_ISL_708137, EPI_ISL_708138, EPI_ISL_708139	Medical Microbiology Unit, Department for Laboratory Medicine, Drammen Hospital, Vestre Viken Health Trust,	Norwegian Institute of Public Health, Department of Virology	Kathrine Stene-Johansen, Kamilla Heddeland Instefjord, Hilde Elshaug, Marie Paulsen Madsen, Rasmus Riis Kopperud, Hilde Vollan, Karoline Bragstad, Olav Hungnes
EPI_ISL_708140, EPI_ISL_708169, EPI_ISL_708170	Norwegian Institute of Public Health, Department of Virology	Norwegian Institute of Public Health, Department of Virology	Kathrine Stene-Johansen, Kamilla Heddeland Instefjord, Hilde Elshaug, Marie Paulsen Madsen, Rasmus Riis Kopperud, Hilde Vollan, Karoline Bragstad, Olav Hungnes
EPI_ISL_708172, EPI_ISL_708173, EPI_ISL_708174, EPI_ISL_708175	Oslo University Hospital, Department of Medical Microbiology	Norwegian Institute of Public Health, Department of Virology	Kathrine Stene-Johansen, Kamilla Heddeland Instefjord, Hilde Elshaug, Marie Paulsen Madsen, Rasmus Riis Kopperud, Hilde Vollan, Karoline Bragstad, Olav Hungnes
EPI_ISL_708180, EPI_ISL_708181	Norwegian Institute of Public Health, Department of Virology	Norwegian Institute of Public Health, Department of Virology	Kathrine Stene-Johansen, Kamilla Heddeland Instefjord, Hilde Elshaug, Marie Paulsen Madsen, Rasmus Riis Kopperud, Hilde Vollan, Karoline Bragstad, Olav Hungnes
EPI_ISL_708200, EPI_ISL_708201, EPI_ISL_708202, EPI_ISL_708203, EPI_ISL_708204, EPI_ISL_708205, EPI_ISL_708206, EPI_ISL_708207, EPI_ISL_708208, EPI_ISL_708209, EPI_ISL_708210, EPI_ISL_708211, EPI_ISL_708212, EPI_ISL_708213, EPI_ISL_708214, EPI_ISL_708215, EPI_ISL_708216, EPI_ISL_708217, EPI_ISL_708218, EPI_ISL_708219, EPI_ISL_708220, EPI_ISL_708221, EPI_ISL_708222, EPI_ISL_708223, EPI_ISL_708224, EPI_ISL_708225, EPI_ISL_708226, EPI_ISL_708227, EPI_ISL_708228, EPI_ISL_708229, EPI_ISL_708230, EPI_ISL_708231, EPI_ISL_708232, EPI_ISL_708233, EPI_ISL_708235, EPI_ISL_708236, EPI_ISL_708237, EPI_ISL_708238, EPI_ISL_708239, EPI_ISL_708240, EPI_ISL_708241, EPI_ISL_708242, EPI_ISL_708244, EPI_ISL_708247, EPI_ISL_708249, EPI_ISL_708251, EPI_ISL_708252, EPI_ISL_708253, EPI_ISL_708261, EPI_ISL_708262, EPI_ISL_708263, EPI_ISL_708264, EPI_ISL_708266, EPI_ISL_708267, EPI_ISL_708268, EPI_ISL_708269, EPI_ISL_708270, EPI_ISL_708272, EPI_ISL_708273, EPI_ISL_708318, EPI_ISL_708320, EPI_ISL_708332, EPI_ISL_708333, EPI_ISL_708345, EPI_ISL_708358, EPI_ISL_708359, EPI_ISL_708362, EPI_ISL_708366, EPI_ISL_708368, EPI_ISL_708369			
see above	Michigan Department of Health and Human Services, Bureau of Laboratories	Michigan Department of Health and Human Services, Bureau of Laboratories	Blankenship HM, Riner D, Soehnlen MK
EPI_ISL_708456, EPI_ISL_708457, EPI_ISL_708458, EPI_ISL_708459	Department of Clinical Microbiology	GIGA Medical Genomics	Keith Durkin, Maria Artesi, Justine Defêche, Gilles Darcis, Michel Moutschen, Sébastien Bontems, Raphaël Boreux, Bouchra Boujemla, Cécile Meex, Pierrette Melin, Marie-Pierre Hayette, Vincent Bours
EPI_ISL_709882	Lighthouse Lab in Milton Keynes	Wellcome Sanger Institute for the COVID-19 Genomics UK (COG-UK) Consortium	The Lighthouse Lab in Milton Keynes and Alex Alderton, Roberto Amato, Sonia Goncalves, Ewan Harrison, David K. Jackson, Ian Johnston, Dominic Kwiatkowski, Cordelia Langford, John Sillitoe on behalf of the Wellcome Sanger Institute COVID-19 Surveillance Team
EPI_ISL_710123	South Eastern Area Laboratory Services (SEALS)	CIDM-PH et al.	CIDM-PH et al.
EPI_ISL_710589, EPI_ISL_710590	Narhalsan Fjallbacka VC	The Public Health Agency of Sweden	Department of Microbiology, The Public Health Agency of Sweden
EPI_ISL_710607	Halsomedicinskt Center	The Public Health Agency of Sweden	Department of Microbiology, The Public Health Agency of Sweden
EPI_ISL_710830, EPI_ISL_710831,	Lighthouse Lab in Glasgow	Wellcome Sanger Institute for the COVID-19 Genomics UK	Harper VanSteenhouse, Yumi Kasai, David Gray, Carol Clugston, Anna Dominiczak and Alex Alderton, Roberto Amato, Sonia Goncalves, Ewan Harrison,

EPI_ISL_710832, EPI_ISL_710833, EPI_ISL_710834		(COG-UK) Consortium	David K. Jackson, Ian Johnston, Dominic Kwiatkowski, Cordelia Langford, John Sillitoe on behalf of the Wellcome Sanger Institute COVID-19 Surveillance Team
EPI_ISL_714098, EPI_ISL_714100, EPI_ISL_714101, EPI_ISL_714102, EPI_ISL_714103, EPI_ISL_714104, EPI_ISL_714105, EPI_ISL_714106, EPI_ISL_714108, EPI_ISL_714109, EPI_ISL_714110, EPI_ISL_714111, EPI_ISL_714112, EPI_ISL_714113, EPI_ISL_714114, EPI_ISL_714124, EPI_ISL_714125, EPI_ISL_714126, EPI_ISL_714127, EPI_ISL_714128, EPI_ISL_714129, EPI_ISL_714130, EPI_ISL_714131, EPI_ISL_714132, EPI_ISL_714133, EPI_ISL_714134, EPI_ISL_714135, EPI_ISL_714136, EPI_ISL_714137, EPI_ISL_714138, EPI_ISL_714139, EPI_ISL_714140, EPI_ISL_714141, EPI_ISL_714142, EPI_ISL_714143, EPI_ISL_714144, EPI_ISL_714145, EPI_ISL_714146, EPI_ISL_714147, EPI_ISL_714148, EPI_ISL_714149, EPI_ISL_714150, EPI_ISL_714151, EPI_ISL_714152, EPI_ISL_714153, EPI_ISL_714154, EPI_ISL_714155, EPI_ISL_714156, EPI_ISL_714157, EPI_ISL_714158, EPI_ISL_714159, EPI_ISL_714160, EPI_ISL_714161, EPI_ISL_714162, EPI_ISL_714163, EPI_ISL_714164, EPI_ISL_714165, EPI_ISL_714166, EPI_ISL_714167, EPI_ISL_714168, EPI_ISL_714169, EPI_ISL_714170, EPI_ISL_714171, EPI_ISL_714172, EPI_ISL_714173, EPI_ISL_714175, EPI_ISL_714176, EPI_ISL_714177, EPI_ISL_714178, EPI_ISL_714179, EPI_ISL_714180, EPI_ISL_714181, EPI_ISL_714183, EPI_ISL_714184, EPI_ISL_714185, EPI_ISL_714186, EPI_ISL_714187, EPI_ISL_714188, EPI_ISL_714189, EPI_ISL_714190, EPI_ISL_714192, EPI_ISL_714193, EPI_ISL_714194, EPI_ISL_714195, EPI_ISL_714199, EPI_ISL_714200, EPI_ISL_714201, EPI_ISL_714202, EPI_ISL_714203, EPI_ISL_714204, EPI_ISL_714205, EPI_ISL_714206, EPI_ISL_714207, EPI_ISL_714208, EPI_ISL_714209, EPI_ISL_714211, EPI_ISL_714212, EPI_ISL_714213, EPI_ISL_714214, EPI_ISL_714215, EPI_ISL_714216, EPI_ISL_714221, EPI_ISL_714223, EPI_ISL_714224, EPI_ISL_714225, EPI_ISL_714226, EPI_ISL_714228, EPI_ISL_714229, EPI_ISL_714230, EPI_ISL_714231, EPI_ISL_714232, EPI_ISL_714233, EPI_ISL_714234, EPI_ISL_714237, EPI_ISL_714238, EPI_ISL_714239, EPI_ISL_714242, EPI_ISL_714243, EPI_ISL_714244, EPI_ISL_714245, EPI_ISL_714246, EPI_ISL_714247, EPI_ISL_714248, EPI_ISL_714249, EPI_ISL_714250, EPI_ISL_714251, EPI_ISL_714255, EPI_ISL_714256, EPI_ISL_714257, EPI_ISL_714261, EPI_ISL_714262, EPI_ISL_714263, EPI_ISL_714265, EPI_ISL_714266, EPI_ISL_714267, EPI_ISL_714268, EPI_ISL_714270, EPI_ISL_714271, EPI_ISL_714273, EPI_ISL_714274, EPI_ISL_714277, EPI_ISL_714278, EPI_ISL_714280, EPI_ISL_714281, EPI_ISL_714283, EPI_ISL_714284, EPI_ISL_714285, EPI_ISL_714286, EPI_ISL_714287, EPI_ISL_714288, EPI_ISL_714290, EPI_ISL_714291, EPI_ISL_714292, EPI_ISL_714293, EPI_ISL_714294, EPI_ISL_714295, EPI_ISL_714296, EPI_ISL_714297, EPI_ISL_714298, EPI_ISL_714299, EPI_ISL_714320, EPI_ISL_714321, EPI_ISL_714322, EPI_ISL_714323, EPI_ISL_714324, EPI_ISL_714325, EPI_ISL_714326, EPI_ISL_714327, EPI_ISL_714328, EPI_ISL_714329, EPI_ISL_714330, EPI_ISL_714331, EPI_ISL_714332, EPI_ISL_714333, EPI_ISL_714334, EPI_ISL_714335, EPI_ISL_714336, EPI_ISL_714337, EPI_ISL_714338, EPI_ISL_714339, EPI_ISL_714340, EPI_ISL_714341, EPI_ISL_714342, EPI_ISL_714343, EPI_ISL_714344, EPI_ISL_714345, EPI_ISL_714346, EPI_ISL_714347, EPI_ISL_714348, EPI_ISL_714349, EPI_ISL_714350, EPI_ISL_714351, EPI_ISL_714352, EPI_ISL_714353, EPI_ISL_714354, EPI_ISL_714355, EPI_ISL_714356, EPI_ISL_714357, EPI_ISL_714358, EPI_ISL_714359, EPI_ISL_714360, EPI_ISL_714361, EPI_ISL_714362, EPI_ISL_714365, EPI_ISL_714367, EPI_ISL_714368, EPI_ISL_714370, EPI_ISL_714371, EPI_ISL_714372, EPI_ISL_714373, EPI_ISL_714374, EPI_ISL_714375, EPI_ISL_714376, EPI_ISL_714378, EPI_ISL_714379, EPI_ISL_714380, EPI_ISL_714381, EPI_ISL_714382, EPI_ISL_714383, EPI_ISL_714384, EPI_ISL_714392, EPI_ISL_714393, EPI_ISL_714394, EPI_ISL_714395, EPI_ISL_714396, EPI_ISL_714397, EPI_ISL_714398, EPI_ISL_714399, EPI_ISL_714400, EPI_ISL_714401, EPI_ISL_714402, EPI_ISL_714403, EPI_ISL_714404, EPI_ISL_714405, EPI_ISL_714406, EPI_ISL_714407, EPI_ISL_714408, EPI_ISL_714409, EPI_ISL_714410, EPI_ISL_714413, EPI_ISL_714414, EPI_ISL_714415, EPI_ISL_714417, EPI_ISL_714418, EPI_ISL_714444, EPI_ISL_714445, EPI_ISL_714446, EPI_ISL_714447, EPI_ISL_714448, EPI_ISL_714449, EPI_ISL_714451, EPI_ISL_714452, EPI_ISL_714453, EPI_ISL_714454, EPI_ISL_714455, EPI_ISL_714456, EPI_ISL_714457, EPI_ISL_714458, EPI_ISL_714459, EPI_ISL_714460, EPI_ISL_714461, EPI_ISL_714465, EPI_ISL_714466, EPI_ISL_714467, EPI_ISL_714468, EPI_ISL_714469, EPI_ISL_714470, EPI_ISL_714471, EPI_ISL_714472, EPI_ISL_714473, EPI_ISL_714474, EPI_ISL_714477, EPI_ISL_714566, EPI_ISL_714567, EPI_ISL_714568, EPI_ISL_714569, EPI_ISL_714570, EPI_ISL_714571, EPI_ISL_714572, EPI_ISL_714573, EPI_ISL_714574, EPI_ISL_714575, EPI_ISL_714576, EPI_ISL_714577, EPI_ISL_714578, EPI_ISL_714579, EPI_ISL_714580, EPI_ISL_714581, EPI_ISL_714582, EPI_ISL_714583, EPI_ISL_714584, EPI_ISL_714585, EPI_ISL_714586, EPI_ISL_714587, EPI_ISL_714588, EPI_ISL_714589, EPI_ISL_714590, EPI_ISL_714591, EPI_ISL_714592, EPI_ISL_714593, EPI_ISL_714594, EPI_ISL_714595, EPI_ISL_714596, EPI_ISL_714597, EPI_ISL_714598, EPI_ISL_714599, EPI_ISL_714600, EPI_ISL_714601, EPI_ISL_714602, EPI_ISL_714603, EPI_ISL_714604, EPI_ISL_714605, EPI_ISL_714606, EPI_ISL_714607, EPI_ISL_714608, EPI_ISL_714609, EPI_ISL_714610, EPI_ISL_714611, EPI_ISL_714612, EPI_ISL_714613, EPI_ISL_714614, EPI_ISL_714615, EPI_ISL_714616, EPI_ISL_714617, EPI_ISL_714618, EPI_ISL_714619, EPI_ISL_714620, EPI_ISL_714621, EPI_ISL_714622, EPI_ISL_714623, EPI_ISL_714624, EPI_ISL_714625, EPI_ISL_714626, EPI_ISL_714627, EPI_ISL_714628, EPI_ISL_714629, EPI_ISL_714630, EPI_ISL_714631, EPI_ISL_714632, EPI_ISL_714633, EPI_ISL_714634, EPI_ISL_714635, EPI_ISL_714636, EPI_ISL_714637, EPI_ISL_714638, EPI_ISL_714639, EPI_ISL_714640, EPI_ISL_714641, EPI_ISL_714642, EPI_ISL_714643, EPI_ISL_714644, EPI_ISL_714645, EPI_ISL_714646, EPI_ISL_714647, EPI_ISL_714648, EPI_ISL_714649, EPI_ISL_714650, EPI_ISL_714651, EPI_ISL_714652, EPI_ISL_714653, EPI_ISL_714654, EPI_ISL_714655, EPI_ISL_714656, EPI_ISL_714657, EPI_ISL_714658, EPI_ISL_714659, EPI_ISL_714660, EPI_ISL_714661, EPI_ISL_714662, EPI_ISL_714663, EPI_ISL_714664, EPI_ISL_714665, EPI_ISL_714666, EPI_ISL_714667, EPI_ISL_714668, EPI_ISL_714669, EPI_ISL_714670, EPI_ISL_714671, EPI_ISL_714672, EPI_ISL_714673, EPI_ISL_714674, EPI_ISL_714675, EPI_ISL_714676, EPI_ISL_714677, EPI_ISL_714678, EPI_ISL_714679, EPI_ISL_714680, EPI_ISL_714681, EPI_ISL_714682, EPI_ISL_714683, EPI_ISL_714684, EPI_ISL_714685, EPI_ISL_714686, EPI_ISL_714687, EPI_ISL_714688, EPI_ISL_714689, EPI_ISL_714690, EPI_ISL_714691, EPI_ISL_714692, EPI_ISL_714693, EPI_ISL_714694, EPI_ISL_714695, EPI_ISL_714696, EPI_ISL_714697, EPI_ISL_714698, EPI_ISL_714699, EPI_ISL_714700, EPI_ISL_714701, EPI_ISL_714702, EPI_ISL_714703, EPI_ISL_714704, EPI_ISL_714705, EPI_ISL_714706, EPI_ISL_714707, EPI_ISL_714708, EPI_ISL_714709, EPI_ISL_714710, EPI_ISL_714711, EPI_ISL_714712, EPI_ISL_714713, EPI_ISL_714714, EPI_ISL_714715, EPI_ISL_714716, EPI_ISL_714717, EPI_ISL_714718, EPI_ISL_714719, EPI_ISL_714720, EPI_ISL_714721, EPI_ISL_714722, EPI_ISL_714723, EPI_ISL_714724, EPI_ISL_714725, EPI_ISL_714726, EPI_ISL_714727, EPI_ISL_714728, EPI_ISL_714729, EPI_ISL_714730, EPI_ISL_714731, EPI_ISL_714732, EPI_ISL_714733, EPI_ISL_714734, EPI_ISL_714735, EPI_ISL_714736, EPI_ISL_714737, EPI_ISL_714738, EPI_ISL_714739, EPI_ISL_714740, EPI_ISL_714741, EPI_ISL_714742, EPI_ISL_714743, EPI_ISL_714744, EPI_ISL_714745, EPI_ISL_714746, EPI_ISL_714747, EPI_ISL_714748, EPI_ISL_714749, EPI_ISL_714750, EPI_ISL_714751, EPI_ISL_714752, EPI_ISL_714753, EPI_ISL_714754, EPI_ISL_714755, EPI_ISL_714756, EPI_ISL_714757, EPI_ISL_714758, EPI_ISL_714759, EPI_ISL_714760, EPI_ISL_714761, EPI_ISL_714762, EPI_ISL_714763, EPI_ISL_714764, EPI_ISL_714765, EPI_ISL_714766, EPI_ISL_714767, EPI_ISL_714768, EPI_ISL_714769, EPI_ISL_714770, EPI_ISL_714771, EPI_ISL_714772, EPI_ISL_714773, EPI_ISL_714774, EPI_ISL_714775, EPI_ISL_714776, EPI_ISL_714777, EPI_ISL_714778, EPI_ISL_714779, EPI_ISL_714780, EPI_ISL_714781, EPI_ISL_714782, EPI_ISL_714783, EPI_ISL_714784, EPI_ISL_714785, EPI_ISL_714786, EPI_ISL_714787, EPI_ISL_714788, EPI_ISL_714789, EPI_ISL_714790, EPI_ISL_714791, EPI_ISL_714792, EPI_ISL_714793, EPI_ISL_714794, EPI_ISL_714795, EPI_ISL_714796, EPI_ISL_714797, EPI_ISL_714798, EPI_ISL_714799, EPI_ISL_714800, EPI_ISL_714801, EPI_ISL_714802, EPI_ISL_714803, EPI_ISL_714804, EPI_ISL_714805, EPI_ISL_714806, EPI_ISL_714807, EPI_ISL_714808, EPI_ISL_714809, EPI_ISL_714810, EPI_ISL_714811, EPI_ISL_714812, EPI_ISL_714813, EPI_ISL_714814, EPI_ISL_714815, EPI_ISL_714816, EPI_ISL_714817, EPI_ISL_714818, EPI_ISL_714819, EPI_ISL_714820, EPI_ISL_714821, EPI_ISL_714822, EPI_ISL_714823, EPI_ISL_714824, EPI_ISL_714825, EPI_ISL_714826, EPI_ISL_714827, EPI_ISL_714828, EPI_ISL_714829, EPI_ISL_714830, EPI_ISL_714831, EPI_ISL_714832, EPI_ISL_714833, EPI_ISL_714834, EPI_ISL_714835, EPI_ISL_714836, EPI_ISL_714837, EPI_ISL_714838, EPI_ISL_714839, EPI_ISL_714840, EPI_ISL_714841, EPI_ISL_714842, EPI_ISL_714843, EPI_ISL_714844, EPI_ISL_714845, EPI_ISL_714846, EPI_ISL_714847, EPI_ISL_714848, EPI_ISL_714849, EPI_ISL_714850, EPI_ISL_714851, EPI_ISL_714852, EPI_ISL_714853, EPI_ISL_714854, EPI_ISL_714855, EPI_ISL_714856, EPI_ISL_714857, EPI_ISL_714858, EPI_ISL_714859, EPI_ISL_714860, EPI_ISL_714861, EPI_ISL_714862, EPI_ISL_714863, EPI_ISL_714864, EPI_ISL_714865, EPI_ISL_714866, EPI_ISL_714867, EPI_ISL_714868, EPI_ISL_714869, EPI_ISL_714870, EPI_ISL_714871, EPI_ISL_714872, EPI_ISL_714873, EPI_ISL_714874, EPI_ISL_714875, EPI_ISL_714876, EPI_ISL_714877, EPI_ISL_714878, EPI_ISL_714879, EPI_ISL_714880, EPI_ISL_714881, EPI_ISL_714882, EPI_ISL_714883, EPI_ISL_714884, EPI_ISL_714885, EPI_ISL_714886, EPI_ISL_714887, EPI_ISL_714888, EPI_ISL_714889, EPI_ISL_714890, EPI_ISL_714891, EPI_ISL_714892, EPI_ISL_714893, EPI_ISL_714894, EPI_ISL_714895, EPI_ISL_714896, EPI_ISL_714897, EPI_ISL_714898, EPI_ISL_714899, EPI_ISL_714900, EPI_ISL_714901, EPI_ISL_714902, EPI_ISL_714903			
see above	Department of Virus and Microbiological Special Diagnostics, Statens Serum Institut, Copenhagen, Denmark	Albertsen Lab, Department of Chemistry and Bioscience, Aalborg University, Denmark	Danish Covid-19 Genome Consortium
EPI_ISL_717620, EPI_ISL_717621	Lab voor klinische biologie	Onderzoeksgroep Virologie	Nick Vereecke, Laurens Lambrechts, Marthe Pauwels, Bruno Verhasselt, Linos Vandekerckhove, Hans Nauwynck, Sebastiaan Theuns
EPI_ISL_717985	Lab voor klinische biologie	Onderzoeksgroep Virologie	Laurens Lambrechts, Nick Vereecke, Marthe Pauwels, Bruno Verhasselt, Linos Vandekerckhove, Hans Nauwynck, Sebastiaan Theuns
EPI_ISL_717987	Lab voor klinische biologie	Onderzoeksgroep Virologie	Nick Vereecke, Laurens Lambrechts, Marthe Pauwels, Bruno Verhasselt, Linos Vandekerckhove, Hans Nauwynck, Sebastiaan Theuns
EPI_ISL_717989	Lab voor klinische biologie	Onderzoeksgroep Virologie	Laurens Lambrechts, Nick Vereecke, Marthe Pauwels, Bruno Verhasselt, Linos Vandekerckhove, Hans Nauwynck, Sebastiaan Theuns
EPI_ISL_718008	Lab voor klinische biologie	Onderzoeksgroep Virologie	Nick Vereecke, Laurens Lambrechts, Marthe Pauwels, Bruno Verhasselt, Linos Vandekerckhove, Hans Nauwynck, Sebastiaan Theuns
EPI_ISL_718010, EPI_ISL_718012	Lab voor klinische biologie	Onderzoeksgroep Virologie	Laurens Lambrechts, Nick Vereecke, Marthe Pauwels, Bruno Verhasselt, Linos Vandekerckhove, Hans Nauwynck, Sebastiaan Theuns
EPI_ISL_718016, EPI_ISL_718017, EPI_ISL_718018	Lab voor klinische biologie	Onderzoeksgroep Virologie	Nick Vereecke, Laurens Lambrechts, Marthe Pauwels, Bruno Verhasselt, Linos Vandekerckhove, Hans Nauwynck, Sebastiaan Theuns
EPI_ISL_718172, EPI_ISL_718200	Ministry of Health Hospitals	Institute of Health and Community Medicine	David Perera, Ooi Mong How, Chua Hock Hin, Tonnii Sia Loong Loong, Wong Jyn Shan, Wong Kiing Aik, Chan Chia Jui
EPI_ISL_718257	Institute of Virology, Biomedical Research Center of the Slovak Academy of Sciences, Bratislava	Faculty of Natural Sciences, Comenius University, Bratislava	Broa Brejová, Viktória Hodorová, Kristína Boršová, Viktória abanová, Sabina Fumaová Havlíková, Juraj Kopáek, Martina Liková, ubomíra Lukáiková, Martina Neboháová, Monika Sláviková, Tomáš Vina, Jozef Nosek, Boris Klempa
EPI_ISL_718258	Institute of Virology, Biomedical Research Center of the Slovak Academy of Sciences, Bratislava	Faculty of Natural Sciences, Comenius University, Bratislava	Viktória Hodorová, Kristína Boršová, Broa Brejová, Viktória abanová, Sabina Fumaová Havlíková, Juraj Kopáek, Martina Liková, ubomíra Lukáiková, Martina Neboháová, Monika Sláviková, Tomáš Vina, Jozef Nosek, Boris Klempa
EPI_ISL_719605, EPI_ISL_719606, EPI_ISL_719607, EPI_ISL_719608, EPI_ISL_719609, EPI_ISL_719610, EPI_ISL_719611, EPI_ISL_719612, EPI_ISL_719613, EPI_ISL_719614, EPI_ISL_719615, EPI_ISL_719616, EPI_ISL_719617, EPI_ISL_719618, EPI_ISL_719619, EPI_ISL_719620, EPI_ISL_719621, EPI_ISL_719622, EPI_ISL_719623, EPI_ISL_719624, EPI_ISL_719625, EPI_ISL_719626, EPI_ISL_719627, EPI_ISL_719628, EPI_ISL_719629, EPI_ISL_719630, EPI_ISL_719631, EPI_ISL_719632, EPI_ISL_719633, EPI_ISL_719634, EPI_ISL_719635, EPI_ISL_719636, EPI_ISL_719637, EPI_ISL_719638, EPI_ISL_719639, EPI_ISL_719640, EPI_ISL_719641, EPI_ISL_719642, EPI_ISL_719643, EPI_ISL_719644, EPI_ISL_719645, EPI_ISL_719646, EPI_ISL_719647, EPI_ISL_719648, EPI_ISL_719649, EPI_ISL_719650, EPI_ISL_719651, EPI_ISL_719652, EPI_ISL_719653, EPI_ISL_719654, EPI_ISL_719655, EPI_ISL_719656, EPI_ISL_719657, EPI_ISL_719658, EPI_ISL_719659, EPI_ISL_719660, EPI_ISL_719661, EPI_ISL_719662, EPI_ISL_719663, EPI_ISL_719664, EPI_ISL_719665, EPI_ISL_719666, EPI_ISL_719667, EPI_ISL_719668, EPI_ISL_719669, EPI_ISL_719670, EPI_ISL_719671, EPI_ISL_719672, EPI_ISL_719673, EPI_ISL_719674, EPI_ISL_719675, EPI_ISL_719676, EPI_ISL_719677, EPI_ISL_719678, EPI_ISL_719679, EPI_ISL_719680, EPI_ISL_719681, EPI_ISL_719682, EPI_ISL_719683, EPI_ISL_719684, EPI_ISL_719685, EPI_ISL_719686, EPI_ISL_719687, EPI_ISL_719688, EPI_ISL_719689, EPI_ISL_719690, EPI_ISL_719691, EPI_ISL_719692, EPI_ISL_719693, EPI_ISL_719694, EPI_ISL_719695, EPI_ISL_719696, EPI_ISL_719697, EPI_ISL_719698, EPI_ISL_719699, EPI_ISL_719700, EPI_ISL_719701, EPI_ISL_719702, EPI_ISL_719703, EPI_ISL_719704, EPI_ISL_719705, EPI_ISL_719706, EPI_ISL_719707, EPI_ISL_719708, EPI_ISL_719709, EPI_ISL_719710, EPI_ISL_719711, EPI_ISL_719712, EPI_ISL_719713, EPI_ISL_719714, EPI_ISL_719715, EPI_ISL_719716, EPI_ISL_719717, EPI_ISL_719718, EPI_ISL_719719, EPI_ISL_719720, EPI_ISL_719721, EPI_ISL_719722, EPI_ISL_719723, EPI_ISL_719724, EPI_ISL_719725, EPI_ISL_719726, EPI_ISL_719727, EPI_ISL_719728, EPI_ISL_719729, EPI_ISL_719730, EPI_ISL_719731, EPI_ISL_719732, EPI_ISL_719733, EPI_ISL_719734, EPI_ISL_719735, EPI_ISL_719736, EPI_ISL_719737, EPI_ISL_719738, EPI_ISL_719739, EPI_ISL_719740, EPI_ISL_719741, EPI_ISL_719742, EPI_ISL_719743, EPI_ISL_719744, EPI_ISL_719745, EPI_ISL_719746, EPI_ISL_719747, EPI_ISL_719748, EPI_ISL_719749, EPI_ISL_719750, EPI_ISL_719751, EPI_ISL_719752, EPI_ISL_719753, EPI_ISL_719754, EPI_ISL_719755, EPI_ISL_719756, EPI_ISL_719757, EPI_ISL_719758, EPI_ISL_719759, EPI_ISL_719760, EPI_ISL_719761, EPI_ISL_719762, EPI_ISL_719763, EPI_ISL_719764, EPI_ISL_719765, EPI_ISL_719766, EPI_ISL_719767, EPI_ISL_719768, EPI_ISL_719769, EPI_ISL_719770, EPI_ISL_719771, EPI_ISL_719772, EPI_ISL_719773, EPI_ISL_719774, EPI_ISL_719775, EPI_ISL_719776, EPI_ISL_719777, EPI_ISL_719778, EPI_ISL_719779, EPI_ISL_719780, EPI_ISL_719781, EPI_ISL_719782, EPI_ISL_719783, EPI_ISL_719784, EPI_ISL_719785, EPI_ISL_719786, EPI_ISL_719787, EPI_ISL_719788, EPI_ISL_719789, EPI_ISL_719790, EPI_ISL_719791, EPI_ISL_719792, EPI_ISL_719793, EPI_ISL_719794, EPI_ISL_719795, EPI_ISL_719796, EPI_ISL_719797, EPI_ISL_719798, EPI_ISL_719799, EPI_ISL_719800, EPI_ISL_719801, EPI_ISL_719802, EPI_ISL_719803, EPI_ISL_719804, EPI_ISL_719805, EPI_ISL_719806, EPI_ISL_719807, EPI_ISL_719808, EPI_ISL_719809, EPI_ISL_719810, EPI_ISL_719811, EPI_ISL_719812, EPI_ISL_719813, EPI_ISL_719814, EPI_ISL_719815, EPI_ISL_719816, EPI_ISL_719817, EPI_ISL_719818, EPI_ISL_719819, EPI_ISL_719820, EPI_ISL_719821, EPI_ISL_719822, EPI_ISL_719823, EPI_ISL_719824, EPI_ISL_719825, EPI_ISL_719826, EPI_ISL_719827, EPI_ISL_719828, EPI_ISL_719829, EPI_ISL_719830, EPI_ISL_719831, EPI_ISL_719832, EPI_ISL_719833, EPI_ISL_719834, EPI_ISL_719835, EPI_ISL_719836, EPI_ISL_719837, EPI_ISL_719838, EPI_ISL_719839, EPI_ISL_719840, EPI_ISL_719841, EPI_ISL_719842, EPI_ISL_719843, EPI_ISL_719844, EPI_ISL_719845, EPI_ISL_719846, EPI_ISL_719847, EPI_ISL_719848, EPI_ISL_719849, EPI_ISL_719850, EPI_ISL_719851, EPI_ISL_719852, EPI_ISL_719853, EPI_ISL_719854, EPI_ISL_719855, EPI_ISL_719856, EPI_ISL_719857, EPI_ISL_719858, EPI_ISL_719859, EPI_ISL_719860, EPI_ISL_719861, EPI_ISL_719862, EPI_ISL_719863, EPI_ISL_719864, EPI_ISL_719865, EPI_ISL_719866, EPI_ISL_719867, EPI_ISL_719868, EPI_ISL_719869, EPI_ISL_719870, EPI_ISL_719871, EPI_ISL_719872, EPI_ISL_719873, EPI_ISL_719874, EPI_ISL_719875, EPI_ISL_719876, EPI_ISL_719877, EPI_ISL_719878, EPI_ISL_719879, EPI_ISL_719880, EPI_ISL_719881, EPI_ISL_719882, EPI_ISL_719883, EPI_ISL_719884, EPI_ISL_719885, EPI_ISL_719886, EPI_ISL_719887, EPI_ISL_719888, EPI_ISL_719889, EPI_ISL_719890, EPI_ISL_719891, EPI_ISL_719892, EPI_ISL_719893, EPI_ISL_719894, EPI_ISL_719895, EPI_ISL_719896, EPI_ISL_719897, EPI_ISL_719898, EPI_ISL_719899, EPI_ISL_719900, EPI_ISL_719901, EPI_ISL_719902, EPI_ISL_719903, EPI_ISL_719904, EPI_ISL_719905, EPI_ISL_719906, EPI_ISL_719907, EPI_ISL_719908, EPI_ISL_719909, EPI_ISL_719910, EPI_ISL_719911, EPI_ISL_719912, EPI_ISL_719913, EPI_ISL_719914, EPI_ISL_719915, EPI_ISL_719916, EPI_ISL_719917, EPI_ISL_719918, EPI_ISL_719919, EPI_ISL_719920, EPI_ISL_719921, EPI_ISL_719922, EPI_ISL_719923, EPI_ISL_719924, EPI_ISL_719925, EPI_ISL_719926, EPI_ISL_719927, EPI_ISL_719928, EPI_ISL_719929, EPI_ISL_719930, EPI_ISL_719931, EPI_ISL_719932, EPI_ISL_719933, EPI_ISL_719934, EPI_ISL_719935, EPI_ISL_719936, EPI_ISL_719937, EPI_ISL_719938, EPI_ISL_719939, EPI_ISL_719940, EPI_ISL_719941, EPI_ISL_719942, EPI_ISL_719943, EPI_ISL_719944, EPI_ISL_719945, EPI_ISL_719946, EPI_ISL_719947, EPI_ISL_719948, EPI_ISL_719949, EPI_ISL_719950, EPI_ISL_719951, EPI_ISL_719952, EPI_ISL_719953, EPI_ISL_719954, EPI_ISL_719955, EPI_ISL_719956, EPI_ISL_719957, EPI_ISL_719958, EPI_ISL_719959, EPI_ISL_719960, EPI_ISL_719961, EPI_ISL_719962, EPI_ISL_719963, EPI_ISL_719964, EPI_ISL_719965, EPI_ISL_719966, EPI_ISL_719967, EPI_ISL_719968, EPI_ISL_719969, EPI_ISL_719970, EPI_ISL_719971, EPI_ISL_719972, EPI_ISL_719973, EPI_ISL_719974, EPI_ISL_719975, EPI_ISL_719976, EPI_ISL_719977, EPI_ISL_719978, EPI_ISL_719979, EPI_ISL_719980, EPI_ISL_719981, EPI_ISL_719982, EPI_ISL_719983, EPI_ISL_719984, EPI_ISL_719985, EPI_ISL_719986			
see above	Lighthouse Lab in Glasgow	Wellcome Sanger Institute for the COVID-19 Genomics UK (COG-UK) Consortium	Harper VanSteenhouse, Yumi Kasai, David Gray, Carol Clugston, Anna Dominiczak and Alex Alderton, Roberto Amato, Sonia Goncalves, Ewan Harrison, David K. Jackson, Ian Johnston, Dominic Kwiatkowski, Cordelia Langford, John Sillitoe on behalf of the Wellcome Sanger Institute COVID-19 Surveillance Team
EPI_ISL_719987, EPI_ISL_719988, EPI_ISL_719989, EPI_ISL_719990, EPI_ISL_719991, EPI_ISL_719992, EPI_ISL_719993, EPI_ISL_719994, EPI_ISL_719995, EPI_ISL_719996, EPI_ISL_719997, EPI_ISL_719998, EPI_ISL_719999, EPI_ISL_720000, EPI_ISL_720001, EPI_ISL_720002, EPI_ISL_720003, EPI_ISL_720004, EPI_ISL_720005, EPI_ISL_720006, EPI_ISL_720007, EPI_ISL_720008, EPI_ISL_720009, EPI_ISL_720010, EPI_ISL_720011, EPI_ISL_720012, EPI_ISL_720013, EPI_ISL_720014, EPI_ISL_720015, EPI_ISL_720016, EPI_ISL_720017, EPI_ISL_720018, EPI_ISL_720019, EPI_ISL_720020, EPI_ISL_720021, EPI_ISL_720022, EPI_ISL_720023, EPI_ISL_720024, EPI_ISL_720025, EPI_ISL_720026, EPI_ISL_720027, EPI_ISL_720028, EPI_ISL_720029, EPI_ISL_720030, EPI_ISL_720031, EPI_ISL_720032, EPI_ISL_720033, EPI_ISL_720034, EPI_ISL_720035, EPI_ISL_720036, EPI_ISL_720037, EPI_ISL_720038, EPI_ISL_720039, EPI_ISL_720040, EPI_ISL_720041, EPI_ISL_720042, EPI_ISL_720043, EPI_ISL_720044, EPI_ISL_720045, EPI_ISL_720046, EPI_ISL_720047, EPI_ISL_720048, EPI_ISL_720049, EPI_ISL_720050, EPI_ISL_720051, EPI_ISL_720052, EPI_ISL_720053, EPI_ISL_720054, EPI_ISL_720055, EPI_ISL_720056, EPI_ISL_720057, EPI_ISL_720058,			

EPI_ISL_720059, EPI_ISL_720060, EPI_ISL_720061, EPI_ISL_720062, EPI_ISL_720063, EPI_ISL_720064, EPI_ISL_720065, EPI_ISL_720066, EPI_ISL_720067, EPI_ISL_720068, EPI_ISL_720069, EPI_ISL_720070, EPI_ISL_720071, EPI_ISL_720072, EPI_ISL_720073, EPI_ISL_720074, EPI_ISL_720075, EPI_ISL_720076, EPI_ISL_720077, EPI_ISL_720078, EPI_ISL_720079, EPI_ISL_720080, EPI_ISL_720081, EPI_ISL_720082, EPI_ISL_720083, EPI_ISL_720084, EPI_ISL_720085, EPI_ISL_720086, EPI_ISL_720087, EPI_ISL_720088, EPI_ISL_720089, EPI_ISL_720090, EPI_ISL_720091, EPI_ISL_720092, EPI_ISL_720093, EPI_ISL_720094, EPI_ISL_720095, EPI_ISL_720096, EPI_ISL_720097, EPI_ISL_720098, EPI_ISL_720099, EPI_ISL_720100, EPI_ISL_720101, EPI_ISL_720102, EPI_ISL_720103, EPI_ISL_720104, EPI_ISL_720105, EPI_ISL_720106, EPI_ISL_720107, EPI_ISL_720108, EPI_ISL_720109, EPI_ISL_720110, EPI_ISL_720111, EPI_ISL_720112, EPI_ISL_720113, EPI_ISL_720114, EPI_ISL_720115, EPI_ISL_720116, EPI_ISL_720117, EPI_ISL_720118, EPI_ISL_720119, EPI_ISL_720120, EPI_ISL_720121, EPI_ISL_720122, EPI_ISL_720123, EPI_ISL_720124, EPI_ISL_720125, EPI_ISL_720126, EPI_ISL_720127, EPI_ISL_720128, EPI_ISL_720129, EPI_ISL_720130, EPI_ISL_720131, EPI_ISL_720132, EPI_ISL_720133, EPI_ISL_720134, EPI_ISL_720135, EPI_ISL_720136, EPI_ISL_720137, EPI_ISL_720138, EPI_ISL_720139, EPI_ISL_720140, EPI_ISL_720141, EPI_ISL_720142, EPI_ISL_720143, EPI_ISL_720144, EPI_ISL_720145, EPI_ISL_720147, EPI_ISL_720148, EPI_ISL_720149, EPI_ISL_720150, EPI_ISL_720151, EPI_ISL_720152, EPI_ISL_720153, EPI_ISL_720154, EPI_ISL_720155, EPI_ISL_720156, EPI_ISL_720157, EPI_ISL_720158, EPI_ISL_720159, EPI_ISL_720160, EPI_ISL_720161, EPI_ISL_720162, EPI_ISL_720163, EPI_ISL_720164, EPI_ISL_720165, EPI_ISL_720166, EPI_ISL_720167, EPI_ISL_720168, EPI_ISL_720169, EPI_ISL_720170, EPI_ISL_720171, EPI_ISL_720172, EPI_ISL_720173, EPI_ISL_720174, EPI_ISL_720175, EPI_ISL_720176, EPI_ISL_720177, EPI_ISL_720178, EPI_ISL_720179, EPI_ISL_720180, EPI_ISL_720181, EPI_ISL_720182, EPI_ISL_720183, EPI_ISL_720184, EPI_ISL_720185, EPI_ISL_720186, EPI_ISL_720187, EPI_ISL_720188, EPI_ISL_720189, EPI_ISL_720190, EPI_ISL_720191, EPI_ISL_720192, EPI_ISL_720193, EPI_ISL_720194, EPI_ISL_720195, EPI_ISL_720196, EPI_ISL_720197, EPI_ISL_720198, EPI_ISL_720199, EPI_ISL_720200, EPI_ISL_720201, EPI_ISL_720202, EPI_ISL_720203, EPI_ISL_720204, EPI_ISL_720205, EPI_ISL_720206, EPI_ISL_720207, EPI_ISL_720208, EPI_ISL_720209, EPI_ISL_720210, EPI_ISL_720211, EPI_ISL_720212, EPI_ISL_720213, EPI_ISL_720214, EPI_ISL_720215, EPI_ISL_720216, EPI_ISL_720217, EPI_ISL_720218, EPI_ISL_720219, EPI_ISL_720220, EPI_ISL_720221, EPI_ISL_720222, EPI_ISL_720223, EPI_ISL_720224, EPI_ISL_720225, EPI_ISL_720226, EPI_ISL_720227, EPI_ISL_720228, EPI_ISL_720229, EPI_ISL_720230, EPI_ISL_720231, EPI_ISL_720232, EPI_ISL_720233, EPI_ISL_720234, EPI_ISL_720235, EPI_ISL_720236, EPI_ISL_720237, EPI_ISL_720238, EPI_ISL_720239, EPI_ISL_720240, EPI_ISL_720241, EPI_ISL_720242, EPI_ISL_720243, EPI_ISL_720244, EPI_ISL_720245, EPI_ISL_720246, EPI_ISL_720247, EPI_ISL_720248, EPI_ISL_720249, EPI_ISL_720250, EPI_ISL_720251, EPI_ISL_720252, EPI_ISL_720253, EPI_ISL_720254, EPI_ISL_720255, EPI_ISL_720256, EPI_ISL_720257, EPI_ISL_720258, EPI_ISL_720259, EPI_ISL_720260, EPI_ISL_720261, EPI_ISL_720262, EPI_ISL_720263, EPI_ISL_720264, EPI_ISL_720265, EPI_ISL_720266, EPI_ISL_720267, EPI_ISL_720268, EPI_ISL_720269, EPI_ISL_720270, EPI_ISL_720271, EPI_ISL_720272, EPI_ISL_720273, EPI_ISL_720274, EPI_ISL_720275, EPI_ISL_720276, EPI_ISL_720277, EPI_ISL_720278, EPI_ISL_720279, EPI_ISL_720280, EPI_ISL_720281, EPI_ISL_720282, EPI_ISL_720283, EPI_ISL_720284, EPI_ISL_720285, EPI_ISL_720286, EPI_ISL_720287, EPI_ISL_720288, EPI_ISL_720289, EPI_ISL_720290, EPI_ISL_720291, EPI_ISL_720292, EPI_ISL_720293, EPI_ISL_720294, EPI_ISL_720295, EPI_ISL_720296, EPI_ISL_720297, EPI_ISL_720298, EPI_ISL_720299, EPI_ISL_720300, EPI_ISL_720301, EPI_ISL_720302, EPI_ISL_720303, EPI_ISL_720304, EPI_ISL_720305, EPI_ISL_720306, EPI_ISL_720307, EPI_ISL_720308, EPI_ISL_720309, EPI_ISL_720310, EPI_ISL_720311, EPI_ISL_720312, EPI_ISL_720313, EPI_ISL_720314, EPI_ISL_720315, EPI_ISL_720316, EPI_ISL_720317, EPI_ISL_720318, EPI_ISL_720319, EPI_ISL_720320, EPI_ISL_720321, EPI_ISL_720322, EPI_ISL_720323, EPI_ISL_720324, EPI_ISL_720325, EPI_ISL_720326, EPI_ISL_720327, EPI_ISL_720328, EPI_ISL_720329, EPI_ISL_720330, EPI_ISL_720331, EPI_ISL_720332, EPI_ISL_720333, EPI_ISL_720334, EPI_ISL_720335, EPI_ISL_720336, EPI_ISL_720337, EPI_ISL_720338, EPI_ISL_720339, EPI_ISL_720340, EPI_ISL_720341, EPI_ISL_720342, EPI_ISL_720343, EPI_ISL_720344, EPI_ISL_720345, EPI_ISL_720346, EPI_ISL_720347, EPI_ISL_720348, EPI_ISL_720349, EPI_ISL_720350, EPI_ISL_720351, EPI_ISL_720352, EPI_ISL_720353, EPI_ISL_720354, EPI_ISL_720355, EPI_ISL_720356, EPI_ISL_720357, EPI_ISL_720358, EPI_ISL_720359, EPI_ISL_720360, EPI_ISL_720361, EPI_ISL_720362, EPI_ISL_720363, EPI_ISL_720364, EPI_ISL_720365, EPI_ISL_720366, EPI_ISL_720367, EPI_ISL_720368, EPI_ISL_720369, EPI_ISL_720370, EPI_ISL_720371, EPI_ISL_720372, EPI_ISL_720373, EPI_ISL_720374, EPI_ISL_720375, EPI_ISL_720376, EPI_ISL_720377, EPI_ISL_720378, EPI_ISL_720379, EPI_ISL_720380, EPI_ISL_720381, EPI_ISL_720382, EPI_ISL_720383, EPI_ISL_720384, EPI_ISL_720385, EPI_ISL_720386, EPI_ISL_720387, EPI_ISL_720388, EPI_ISL_720389, EPI_ISL_720390, EPI_ISL_720391, EPI_ISL_720392, EPI_ISL_720393, EPI_ISL_720394, EPI_ISL_720395, EPI_ISL_720396, EPI_ISL_720397, EPI_ISL_720398, EPI_ISL_720399, EPI_ISL_720400, EPI_ISL_720401, EPI_ISL_720402, EPI_ISL_720403, EPI_ISL_720404, EPI_ISL_720405, EPI_ISL_720406, EPI_ISL_720407, EPI_ISL_720408, EPI_ISL_720409, EPI_ISL_720410, EPI_ISL_720411, EPI_ISL_720412, EPI_ISL_720413, EPI_ISL_720414, EPI_ISL_720415, EPI_ISL_720416, EPI_ISL_720417, EPI_ISL_720418, EPI_ISL_720419, EPI_ISL_720420, EPI_ISL_720421, EPI_ISL_720422, EPI_ISL_720423, EPI_ISL_720424, EPI_ISL_720425, EPI_ISL_720426, EPI_ISL_720427, EPI_ISL_720428, EPI_ISL_720429, EPI_ISL_720430, EPI_ISL_720431, EPI_ISL_720432, EPI_ISL_720433, EPI_ISL_720434, EPI_ISL_720435, EPI_ISL_720436, EPI_ISL_720437, EPI_ISL_720438, EPI_ISL_720439, EPI_ISL_720440, EPI_ISL_720441, EPI_ISL_720442, EPI_ISL_720443, EPI_ISL_720444, EPI_ISL_720445, EPI_ISL_720446, EPI_ISL_720447, EPI_ISL_720448, EPI_ISL_720449, EPI_ISL_720450, EPI_ISL_720451, EPI_ISL_720452, EPI_ISL_720453, EPI_ISL_720454, EPI_ISL_720455, EPI_ISL_720456, EPI_ISL_720457, EPI_ISL_720458, EPI_ISL_720459, EPI_ISL_720460, EPI_ISL_720461, EPI_ISL_720462, EPI_ISL_720463, EPI_ISL_720464, EPI_ISL_720465, EPI_ISL_720466, EPI_ISL_720467, EPI_ISL_720468, EPI_ISL_720469, EPI_ISL_720470, EPI_ISL_720471, EPI_ISL_720472, EPI_ISL_720473, EPI_ISL_720474, EPI_ISL_720475, EPI_ISL_720476, EPI_ISL_720477, EPI_ISL_720478, EPI_ISL_720479, EPI_ISL_720480, EPI_ISL_720481, EPI_ISL_720482, EPI_ISL_720483, EPI_ISL_720484, EPI_ISL_720485, EPI_ISL_720486, EPI_ISL_720487, EPI_ISL_720488, EPI_ISL_720489, EPI_ISL_720490, EPI_ISL_720491, EPI_ISL_720492, EPI_ISL_720493, EPI_ISL_720494, EPI_ISL_720495, EPI_ISL_720496, EPI_ISL_720497, EPI_ISL_720498, EPI_ISL_720499, EPI_ISL_720500, EPI_ISL_720501, EPI_ISL_720502, EPI_ISL_720503, EPI_ISL_720504, EPI_ISL_720505, EPI_ISL_720506, EPI_ISL_720507, EPI_ISL_720508, EPI_ISL_720509, EPI_ISL_720510, EPI_ISL_720511, EPI_ISL_720512, EPI_ISL_720513, EPI_ISL_720514, EPI_ISL_720515, EPI_ISL_720516, EPI_ISL_720517, EPI_ISL_720518, EPI_ISL_720519, EPI_ISL_720520, EPI_ISL_720521, EPI_ISL_720522, EPI_ISL_720523, EPI_ISL_720524, EPI_ISL_720525, EPI_ISL_720526, EPI_ISL_720527, EPI_ISL_720528, EPI_ISL_720529, EPI_ISL_720530, EPI_ISL_720531, EPI_ISL_720532, EPI_ISL_720533, EPI_ISL_720534, EPI_ISL_720535, EPI_ISL_720536, EPI_ISL_720537, EPI_ISL_720538, EPI_ISL_720539, EPI_ISL_720540, EPI_ISL_720541, EPI_ISL_720542, EPI_ISL_720543, EPI_ISL_720544, EPI_ISL_720545, EPI_ISL_720546, EPI_ISL_720547, EPI_ISL_720548, EPI_ISL_720549, EPI_ISL_720550, EPI_ISL_720551, EPI_ISL_720552, EPI_ISL_720553, EPI_ISL_720554, EPI_ISL_720555, EPI_ISL_720556, EPI_ISL_720557, EPI_ISL_720558, EPI_ISL_720559, EPI_ISL_720560, EPI_ISL_720561, EPI_ISL_720562, EPI_ISL_720563, EPI_ISL_720564, EPI_ISL_720565, EPI_ISL_720566, EPI_ISL_720567, EPI_ISL_720568, EPI_ISL_720569, EPI_ISL_720570, EPI_ISL_720571, EPI_ISL_720572, EPI_ISL_720573, EPI_ISL_720574, EPI_ISL_720575, EPI_ISL_720576, EPI_ISL_720577, EPI_ISL_720578, EPI_ISL_720579, EPI_ISL_720580, EPI_ISL_720581, EPI_ISL_720582, EPI_ISL_720583, EPI_ISL_720584, EPI_ISL_720585, EPI_ISL_720586, EPI_ISL_720587, EPI_ISL_720588, EPI_ISL_720589, EPI_ISL_720590, EPI_ISL_720591, EPI_ISL_720592, EPI_ISL_720593, EPI_ISL_720594, EPI_ISL_720595, EPI_ISL_720596, EPI_ISL_720597, EPI_ISL_720598, EPI_ISL_720599, EPI_ISL_720600, EPI_ISL_720601, EPI_ISL_720602, EPI_ISL_720603, EPI_ISL_720604, EPI_ISL_720605, EPI_ISL_720606, EPI_ISL_720607, EPI_ISL_720608, EPI_ISL_720609, EPI_ISL_720610, EPI_ISL_720611, EPI_ISL_720612, EPI_ISL_720613, EPI_ISL_720614, EPI_ISL_720615, EPI_ISL_720616, EPI_ISL_720617, EPI_ISL_720618, EPI_ISL_720619, EPI_ISL_720620, EPI_ISL_720621, EPI_ISL_720622, EPI_ISL_720623, EPI_ISL_720624, EPI_ISL_720625, EPI_ISL_720626, EPI_ISL_720627, EPI_ISL_720628, EPI_ISL_720629, EPI_ISL_720630, EPI_ISL_720631, EPI_ISL_720632, EPI_ISL_720633, EPI_ISL_720634, EPI_ISL_720635, EPI_ISL_720636, EPI_ISL_720637, EPI_ISL_720638, EPI_ISL_720639, EPI_ISL_720640, EPI_ISL_720641, EPI_ISL_720642, EPI_ISL_720643, EPI_ISL_720644, EPI_ISL_720645, EPI_ISL_720646, EPI_ISL_720647, EPI_ISL_720648, EPI_ISL_720649, EPI_ISL_720650, EPI_ISL_720651, EPI_ISL_720652, EPI_ISL_720653, EPI_ISL_720654, EPI_ISL_720655, EPI_ISL_720656, EPI_ISL_720657, EPI_ISL_720658, EPI_ISL_720659, EPI_ISL_720660, EPI_ISL_720661, EPI_ISL_720662, EPI_ISL_720663, EPI_ISL_720664, EPI_ISL_720665, EPI_ISL_720666, EPI_ISL_720667			
see above	Lighthouse Lab in Milton Keynes	Wellcome Sanger Institute for the COVID-19 Genomics UK (COG-UK) Consortium	The Lighthouse Lab in Milton Keynes and Alex Alderton, Roberto Amato, Sonia Goncalves, Ewan Harrison, David K. Jackson, Ian Johnston, Dominic Kwiatkowski, Cordelia Langford, John Sillitoe on behalf of the Wellcome Sanger Institute COVID-19 Surveillance Team
EPI_ISL_720909, EPI_ISL_720910, EPI_ISL_720911, EPI_ISL_720912, EPI_ISL_720913, EPI_ISL_720914, EPI_ISL_720915, EPI_ISL_720916, EPI_ISL_720917, EPI_ISL_720918, EPI_ISL_720919, EPI_ISL_720920, EPI_ISL_720921, EPI_ISL_720922, EPI_ISL_720923, EPI_ISL_720924, EPI_ISL_720925, EPI_ISL_720926, EPI_ISL_720927, EPI_ISL_720928, EPI_ISL_720929, EPI_ISL_720930, EPI_ISL_720931, EPI_ISL_720932, EPI_ISL_720933, EPI_ISL_720934, EPI_ISL_720935, EPI_ISL_720936, EPI_ISL_720937, EPI_ISL_720938, EPI_ISL_720939, EPI_ISL_720940, EPI_ISL_720941, EPI_ISL_720942, EPI_ISL_720943, EPI_ISL_720944, EPI_ISL_720945, EPI_ISL_720946, EPI_ISL_720947, EPI_ISL_720948, EPI_ISL_720949, EPI_ISL_720950, EPI_ISL_720951, EPI_ISL_720952, EPI_ISL_720953, EPI_ISL_720954, EPI_ISL_720955, EPI_ISL_720956, EPI_ISL_720957, EPI_ISL_720958, EPI_ISL_720959, EPI_ISL_720960, EPI_ISL_720961, EPI_ISL_720962, EPI_ISL_720963, EPI_ISL_720964, EPI_ISL_720965, EPI_ISL_720966, EPI_ISL_720967, EPI_ISL_720968, EPI_ISL_720969, EPI_ISL_720970, EPI_ISL_720971, EPI_ISL_720972, EPI_ISL_720973, EPI_ISL_720974, EPI_ISL_720975, EPI_ISL_720976, EPI_ISL_720977, EPI_ISL_720978, EPI_ISL_720979, EPI_ISL_720980, EPI_ISL_720981, EPI_ISL_720982, EPI_ISL_720983, EPI_ISL_720984, EPI_ISL_720985, EPI_ISL_720986, EPI_ISL_720987, EPI_ISL_720988, EPI_ISL_720989, EPI_ISL_720990, EPI_ISL_720991, EPI_ISL_720992, EPI_ISL_720993, EPI_ISL_720994, EPI_ISL_720995, EPI_ISL_720996, EPI_ISL_720997, EPI_ISL_720998, EPI_ISL_720999, EPI_ISL_721000, EPI_ISL_721001, EPI_ISL_721002, EPI_ISL_721003, EPI_ISL_721004, EPI_ISL_721005, EPI_ISL_721006, EPI_ISL_721007, EPI_ISL_721008, EPI_ISL_721009, EPI_ISL_721010, EPI_ISL_721011, EPI_ISL_721012, EPI_ISL_721013, EPI_ISL_721014, EPI_ISL_721015, EPI_ISL_721016, EPI_ISL_721017, EPI_ISL_721018, EPI_ISL_721019, EPI_ISL_721020, EPI_ISL_721021, EPI_ISL_721022, EPI_ISL_721023, EPI_ISL_721024, EPI_ISL_721025, EPI_ISL_721026, EPI_ISL_721027, EPI_ISL_721028, EPI_ISL_721029, EPI_ISL_721030, EPI_ISL_721031, EPI_ISL_721032, EPI_ISL_721033, EPI_ISL_721034, EPI_ISL_721035, EPI_ISL_721036, EPI_ISL_721037, EPI_ISL_721038, EPI_ISL_721039, EPI_ISL_721040, EPI_ISL_721041, EPI_ISL_721042, EPI_ISL_721043, EPI_ISL_721044, EPI_ISL_721045, EPI_ISL_721046, EPI_ISL_721047, EPI_ISL_721048, EPI_ISL_721049, EPI_ISL_721050, EPI_ISL_721051, EPI_ISL_721052, EPI_ISL_721053, EPI_ISL_721054, EPI_ISL_721055, EPI_ISL_721056, EPI_ISL_721057, EPI_ISL_721058, EPI_ISL_721059, EPI_ISL_721060, EPI_ISL_721061, EPI_ISL_721062, EPI_ISL_721063, EPI_ISL_721064, EPI_ISL_721065, EPI_ISL_721066, EPI_ISL_721067, EPI_ISL_721068, EPI_ISL_721069, EPI_ISL_721070, EPI_ISL_721071, EPI_ISL_721072, EPI_ISL_721073, EPI_ISL_721074, EPI_ISL_721075, EPI_ISL_721076, EPI_ISL_721077, EPI_ISL_721078, EPI_ISL_721079, EPI_ISL_721080, EPI_ISL_721081, EPI_ISL_721082, EPI_ISL_721083, EPI_ISL_721084, EPI_ISL_721085, EPI_ISL_721086, EPI_ISL_721087, EPI_ISL_721088, EPI_ISL_721089, EPI_ISL_721090, EPI_ISL_721091, EPI_ISL_721092, EPI_ISL_721093, EPI_ISL_721094, EPI_ISL_721095, EPI_ISL_721096, EPI_ISL_721097, EPI_ISL_721098, EPI_ISL_721099, EPI_ISL_721100, EPI_ISL_721101, EPI_ISL_721102, EPI_ISL_721103, EPI_ISL_721104, EPI_ISL_721105, EPI_ISL_721106, EPI_ISL_721107, EPI_ISL_721108, EPI_ISL_721109, EPI_ISL_721110, EPI_ISL_721111, EPI_ISL_721112, EPI_ISL_721113, EPI_ISL_721114, EPI_ISL_721115, EPI_ISL_721116, EPI_ISL_721117, EPI_ISL_721118, EPI_ISL_721119, EPI_ISL_721120, EPI_ISL_721121, EPI_ISL_721122, EPI_ISL_721123, EPI_ISL_721124, EPI_ISL_721125, EPI_ISL_721126, EPI_ISL_721127, EPI_ISL_721128, EPI_ISL_721129, EPI_ISL_721130, EPI_ISL_721131, EPI_ISL_721132, EPI_ISL_721133, EPI_ISL_721134, EPI_ISL_721135, EPI_ISL_721136, EPI_ISL_721137, EPI_ISL_721138, EPI_ISL_721139, EPI_ISL_721140, EPI_ISL_721141, EPI_ISL_721142, EPI_ISL_721143, EPI_ISL_721144, EPI_ISL_721145, EPI_ISL_721146, EPI_ISL_721147, EPI_ISL_721148, EPI_ISL_721149, EPI_ISL_721150, EPI_ISL_721151, EPI_ISL_721152, EPI_ISL_721153, EPI_ISL_721154, EPI_ISL_721155, EPI_ISL_721156, EPI_ISL_721157, EPI_ISL_721158, EPI_ISL_721159, EPI_ISL_721160, EPI_ISL_721161, EPI_ISL_721162, EPI_ISL_721163, EPI_ISL_721164, EPI_ISL_721165, EPI_ISL_721166, EPI_ISL_721167, EPI_ISL_721168, EPI_ISL_721169, EPI_ISL_721170, EPI_ISL_721171, EPI_ISL_721172, EPI_ISL_721173, EPI_ISL_721174, EPI_ISL_721175, EPI_ISL_721176, EPI_ISL_721177, EPI_ISL_721178, EPI_ISL_721179, EPI_ISL_721180, EPI_ISL_721181, EPI_ISL_721182, EPI_ISL_721183, EPI_ISL_721184			
see above	Lighthouse Lab in Glasgow	Wellcome Sanger Institute for the COVID-19 Genomics UK (COG-UK) Consortium	Harper VanSteenhouse, Yumi Kasai, David Gray, Carol Clugston, Anna Dominiczak and Alex Alderton, Roberto Amato, Sonia Goncalves, Ewan Harrison, David K. Jackson, Ian Johnston, Dominic Kwiatkowski, Cordelia Langford, John Sillitoe on behalf of the Wellcome Sanger Institute COVID-19 Surveillance Team
EPI_ISL_721306, EPI_ISL_721370, EPI_ISL_721456, EPI_ISL_721460	Lighthouse Lab in Alderley Park	Wellcome Sanger Institute for the COVID-19 Genomics UK (COG-UK) Consortium	Jacquelyn Wynn, Mairead Hyland, The Lighthouse Lab in Alderley Park and Alex Alderton, Roberto Amato, Sonia Goncalves, Ewan Harrison, David K. Jackson, Ian Johnston, Dominic Kwiatkowski, Cordelia Langford, John Sillitoe on behalf of the Wellcome Sanger Institute COVID-19 Surveillance Team
EPI_ISL_721624	National Institute for Infectious Diseases, INMI, "L. Spallanzani" IRCCS	National Institute for Infectious Diseases, INMI, "L. Spallanzani" IRCCS	C.E.M Gruber, B Bartolini, M Rueca, F Messina, E Giombini, A Di Caro, MR Capobianchi
EPI_ISL_721625	National Institute for Infectious Diseases, INMI, "L. Spallanzani" IRCCS	National Institute for Infectious Diseases, INMI, "L. Spallanzani" IRCCS	B Bartolini, C.E.M Gruber, M Rueca, F Messina, E Giombini, MR Capobianchi, A Di Caro
EPI_ISL_721628	National Institute for Infectious Diseases, INMI, "L. Spallanzani" IRCCS	National Institute for Infectious Diseases, INMI, "L. Spallanzani" IRCCS	E Giombini, M Rueca, B Bartolini, C.E.M Gruber, F Messina, A Di Caro, MR Capobianchi
EPI_ISL_722276, EPI_ISL_722277, EPI_ISL_722279, EPI_ISL_722284, EPI_ISL_722288, EPI_ISL_722302, EPI_ISL_722306, EPI_ISL_722321, EPI_ISL_722326, EPI_ISL_722327, EPI_ISL_722331, EPI_ISL_722340, EPI_ISL_722345, EPI_ISL_722346, EPI_ISL_722350, EPI_ISL_722364, EPI_ISL_722376, EPI_ISL_722391, EPI_ISL_722393, EPI_ISL_722395, EPI_ISL_722408, EPI_ISL_722419, EPI_ISL_722432, EPI_ISL_722648, EPI_ISL_722649, EPI_ISL_722650, EPI_ISL_722651, EPI_ISL_722652, EPI_ISL_722653, EPI_ISL_722654, EPI_ISL_722655, EPI_ISL_722656, EPI_ISL_722657, EPI_ISL_722658, EPI_ISL_722659, EPI_ISL_722660, EPI_ISL_722661, EPI_ISL_722662, EPI_ISL_722663, EPI_ISL_722664, EPI_ISL_722665, EPI_ISL_722666, EPI_ISL_722667, EPI_ISL_722668, EPI_ISL_722669, EPI_ISL_722670, EPI_ISL_722671, EPI_ISL_722672, EPI_ISL_722673, EPI_ISL_722674, EPI_ISL_722675, EPI_ISL_722676, EPI_ISL_722677, EPI_ISL_722678			
see above	Dutch COVID-19 response team	Erasmus Medical Center	Bas Oude Munnink, Reina Sikkema, David Nieuwenhuijse, Irina Chestakova, Anne van der Linden, Marjan Boter, Emmanuelle Munger, Corine GeurtsvanKessel, Annemiek van der Eijk, Richard Molenkamp, Marion Koopmans, on behalf of the Dutch national COVID-19 response team.
EPI_ISL_723123	Minnesota Department of Health, Public Health Laboratory	Minnesota Department of Health, Public Health Laboratory	Alexandra Lorentz, Jacob Garfin, Matt Plumb, and Xiong Wang
EPI_ISL_723145	Mayo Clinic & Mayo Clinic Laboratories	Minnesota Department of Health, Public Health Laboratory	Alexandra Lorentz, Jacob Garfin, Matt Plumb, and Xiong Wang
EPI_ISL_723157, EPI_ISL_723179, EPI_ISL_723225, EPI_ISL_723251, EPI_ISL_723272, EPI_ISL_723273, EPI_ISL_723274, EPI_ISL_723292, EPI_ISL_723293, EPI_ISL_723294, EPI_ISL_723295, EPI_ISL_723296, EPI_ISL_723297, EPI_ISL_723324, EPI_ISL_723325, EPI_ISL_723406, EPI_ISL_723407, EPI_ISL_723408, EPI_ISL_723409, EPI_ISL_723410, EPI_ISL_723411, EPI_ISL_723424, EPI_ISL_723428, EPI_ISL_723430, EPI_ISL_723431, EPI_ISL_723432, EPI_ISL_723433, EPI_ISL_723434, EPI_ISL_723435, EPI_ISL_723436, EPI_ISL_723440, EPI_ISL_723444, EPI_ISL_723459			
see above	Dutch COVID-19 response team	National Institute for Public Health and the Environment (RIVM)	Adam Meijer, Harry Vennema, Jeroen Cremer, Sharon van den Brink, Bas van der Veer, AnneMarie van den Brandt, Florian Zwagemaker, Dennis Schmitz, Chantal Reusken, on behalf of the national COVID-19 response team
EPI_ISL_723493, EPI_ISL_723512, EPI_ISL_723513, EPI_ISL_723514	Virginia Division of Consolidated Laboratory Services (DCLS)	Virginia Division of Consolidated Laboratory Services (DCLS)	Virginia DCLS
EPI_ISL_724063	Northumbria University / South Tees Hospitals NHS Foundation Trust / North Cumbria Integrated Care NHS Foundation Trust / North Tees and Hartlepool NHS Foundation Trust / Newcastle Hospitals NHS Foundation Trust	COVID-19 Genomics UK (COG-UK) Consortium	Darren L Smith,Andrew Nelson,Matthew Bashton,Greg R Young,Joshua Loh,John Allan,Mohammad A Tariq,Giles S Holt,Gary Black,Wen C Yew,Lynn Dover,Paul Baker,Steve Liggett,Sarah Essex,Jane Greenaway,Debra Padgett,Clive Graham,Garren Scott,Edward Barton,Emma Swindells,Brendan Payne,Jennifer Collins,Yusri Taha,Gary Eltringham

EPI_ISL_724125, EPI_ISL_724126, EPI_ISL_724127, EPI_ISL_724128, EPI_ISL_724129, EPI_ISL_724148, EPI_ISL_724149, EPI_ISL_724150, EPI_ISL_724188, EPI_ISL_724200, EPI_ISL_724201, EPI_ISL_724202, EPI_ISL_724203, EPI_ISL_724204, EPI_ISL_724205, EPI_ISL_724206, EPI_ISL_724207, EPI_ISL_724208, EPI_ISL_724209			
see above	Quadram Institute Bioscience	COVID-19 Genomics UK (COG-UK) Consortium	Dave J. Baker, Gemma L. Kay, Alp Aydin, Thanh Le-Viet, Steven Rudder, Ana P. Tedim, Anastasia Kolyva, Maria Diaz, Leonardo de Oliveira Martins, Nabil-Fareed Alikhan, Lizzie Meadows, Rachael Stanley, Ngozi Elumogo, Muhammed Yasir, Nicholas M. Thomson, Alexander J Trotter, Rachel Gilroy, Samuel Bloomfield, Claire Stuart, Andrew Bell, Reenesh Prakash, Samir Dervisevic, Alison E. Mather, John Wain, Mark Webber, Andrew J. Page, Justin O'Grady
EPI_ISL_724212	West of Scotland Specialist Virology Centre, NHSGGC / MRC-University of Glasgow Centre for Virus Research	COVID-19 Genomics UK (COG-UK) Consortium	Ana da Silva Filipe, Natasha Johnson, Kathy Smollett, Daniel Mair, Stephen Carmichael, Alice Broos, Lily Tong, Jenna Nichols, Kyriaki Nomikou; Sarah McDonald; Richard Orton, Joseph Hughes, Sreenu Vattipally, David L Robertson; Alasdair MacLean, Rory Gunson; Sharif Shaaban, Matthew Holden; Rachel Blacow, Guy Mollett, Kathy Li, James Shepherd, Antonia Ho, Emma Thomson
EPI_ISL_724541, EPI_ISL_724549, EPI_ISL_724612, EPI_ISL_724616, EPI_ISL_724619, EPI_ISL_724641, EPI_ISL_724653, EPI_ISL_724654, EPI_ISL_724663, EPI_ISL_724668, EPI_ISL_724669, EPI_ISL_724670, EPI_ISL_724688, EPI_ISL_724711, EPI_ISL_724714, EPI_ISL_724721, EPI_ISL_724768, EPI_ISL_724769, EPI_ISL_724770, EPI_ISL_724772, EPI_ISL_724774, EPI_ISL_724775			
see above	University College London, Great Ormond Street Hospital for Children NHS Foundation Trust, Imperial College Healthcare NHS Trust	COVID-19 Genomics UK (COG-UK) Consortium	Sergi Castellano, Rachel Williams, Mark Kristiansen, Paola Resende Silva, Sunando Roy, Tony Brooks, Helena Tutill, Paola Niola, Patricia Dyal, Charlotte Williams, Leysa Forrest, Yasmin Panchbhaya, Jacqueline Findlay, Samuel Weeks, Julianne Brown, Kathryn Harris, Paul Randell, James Price, Alison Holmes, Judith Breuer
EPI_ISL_724924, EPI_ISL_724925, EPI_ISL_724926, EPI_ISL_724928, EPI_ISL_724929, EPI_ISL_724931, EPI_ISL_725007, EPI_ISL_725008, EPI_ISL_725009, EPI_ISL_725010, EPI_ISL_725011			
see above	Northumbria University / South Tees Hospitals NHS Foundation Trust / North Cumbria Integrated Care NHS Foundation Trust / North Tees and Hartlepool NHS Foundation Trust / Newcastle Hospitals NHS Foundation Trust	COVID-19 Genomics UK (COG-UK) Consortium	Darren L Smith,Andrew Nelson,Matthew Bashton,Greg R Young,Joshua Loh,John Allan,Mohammad A Tariq,Giles S Holt,Gary Black,Wen C Yew,Lynn Dover,Paul Baker,Steve Liggett,Sarah Essex,Jane Greenaway,Debra Padgett,Clive Graham,Garren Scott,Edward Barton,Emma Swindells,Brendan Payne,Jennifer Collins,Yusri Taha,Gary Eltringham
EPI_ISL_725160, EPI_ISL_725174, EPI_ISL_725177, EPI_ISL_725178, EPI_ISL_725179, EPI_ISL_725183, EPI_ISL_725184, EPI_ISL_725186, EPI_ISL_725188, EPI_ISL_725189, EPI_ISL_725194, EPI_ISL_725195, EPI_ISL_725196, EPI_ISL_725197, EPI_ISL_725199, EPI_ISL_725200, EPI_ISL_725202, EPI_ISL_725203, EPI_ISL_725207, EPI_ISL_725208, EPI_ISL_725209, EPI_ISL_725211, EPI_ISL_725213, EPI_ISL_725214, EPI_ISL_725215, EPI_ISL_725216, EPI_ISL_725217, EPI_ISL_725218, EPI_ISL_725219, EPI_ISL_725221, EPI_ISL_725222, EPI_ISL_725228, EPI_ISL_725233, EPI_ISL_725237, EPI_ISL_725238, EPI_ISL_725239, EPI_ISL_725240, EPI_ISL_725241, EPI_ISL_725242, EPI_ISL_725248, EPI_ISL_725249, EPI_ISL_725251, EPI_ISL_725252, EPI_ISL_725254, EPI_ISL_725258, EPI_ISL_725260, EPI_ISL_725262, EPI_ISL_725281, EPI_ISL_725283			
see above	Quadram Institute Bioscience	COVID-19 Genomics UK (COG-UK) Consortium	Dave J. Baker, Gemma L. Kay, Alp Aydin, Thanh Le-Viet, Steven Rudder, Ana P. Tedim, Anastasia Kolyva, Maria Diaz, Leonardo de Oliveira Martins, Nabil-Fareed Alikhan, Lizzie Meadows, Rachael Stanley, Ngozi Elumogo, Muhammed Yasir, Nicholas M. Thomson, Alexander J Trotter, Rachel Gilroy, Samuel Bloomfield, Claire Stuart, Andrew Bell, Reenesh Prakash, Samir Dervisevic, Alison E. Mather, John Wain, Mark Webber, Andrew J. Page, Justin O'Grady
EPI_ISL_725679, EPI_ISL_725718, EPI_ISL_725720, EPI_ISL_725721, EPI_ISL_725722, EPI_ISL_725723, EPI_ISL_725724, EPI_ISL_725982, EPI_ISL_725984, EPI_ISL_725994, EPI_ISL_725995, EPI_ISL_725996, EPI_ISL_725997, EPI_ISL_725998, EPI_ISL_726002, EPI_ISL_726003, EPI_ISL_726004, EPI_ISL_726005, EPI_ISL_726006, EPI_ISL_726010, EPI_ISL_726011, EPI_ISL_726012, EPI_ISL_726013, EPI_ISL_726014, EPI_ISL_726015, EPI_ISL_726016, EPI_ISL_726025, EPI_ISL_726032, EPI_ISL_726053, EPI_ISL_726063, EPI_ISL_726064, EPI_ISL_726065, EPI_ISL_726066, EPI_ISL_726067, EPI_ISL_726068, EPI_ISL_726069, EPI_ISL_726070, EPI_ISL_726071, EPI_ISL_726072, EPI_ISL_726073, EPI_ISL_726074, EPI_ISL_726075, EPI_ISL_726076, EPI_ISL_726077, EPI_ISL_726078, EPI_ISL_726079, EPI_ISL_726080, EPI_ISL_726081, EPI_ISL_726082, EPI_ISL_726083, EPI_ISL_726084, EPI_ISL_726085, EPI_ISL_726086, EPI_ISL_726087, EPI_ISL_726088, EPI_ISL_726089, EPI_ISL_726090, EPI_ISL_726091, EPI_ISL_726092, EPI_ISL_726093, EPI_ISL_726094, EPI_ISL_726095, EPI_ISL_726096, EPI_ISL_726097, EPI_ISL_726098, EPI_ISL_726099, EPI_ISL_726100, EPI_ISL_726101, EPI_ISL_726102, EPI_ISL_726103, EPI_ISL_726104, EPI_ISL_726105, EPI_ISL_726106, EPI_ISL_726108, EPI_ISL_726109, EPI_ISL_726110, EPI_ISL_726112, EPI_ISL_726113, EPI_ISL_726114, EPI_ISL_726115, EPI_ISL_726116, EPI_ISL_726117, EPI_ISL_726118, EPI_ISL_726119, EPI_ISL_726121, EPI_ISL_726122, EPI_ISL_726124, EPI_ISL_726125, EPI_ISL_726126, EPI_ISL_726127, EPI_ISL_726128, EPI_ISL_726130, EPI_ISL_726208, EPI_ISL_726213, EPI_ISL_726253, EPI_ISL_726254, EPI_ISL_726255, EPI_ISL_726256, EPI_ISL_726280, EPI_ISL_726306, EPI_ISL_726308, EPI_ISL_726309, EPI_ISL_726310, EPI_ISL_726522, EPI_ISL_726537, EPI_ISL_726570, EPI_ISL_726571, EPI_ISL_726605, EPI_ISL_726606, EPI_ISL_726607, EPI_ISL_726609, EPI_ISL_726610, EPI_ISL_726611, EPI_ISL_726613, EPI_ISL_726615, EPI_ISL_726616, EPI_ISL_726618, EPI_ISL_726619			
see above	Wales Specialist Virology Centre Sequencing lab: Pathogen Genomics Unit	COVID-19 Genomics UK (COG-UK) Consortium	Catherine Moore, Johnathan Evans, Laura Gifford, Malorie Perry, Simon Cottrell, Angela Marchbank, Alec Birchley, Alexander Adams, Amy Gaskin, Bree Gatica-Wilcox, Jason Coombes, Joel Southgate, Lauren Gilbert, Lee Graham, Nicole Pacchiarini, Sara Kumziene-Summerhayes, Sarah Taylor, Sophie Jones, Sara Rey, Matthew Bull, Joanne Watkins, Sally Corden, Tom Connor
EPI_ISL_727734	Centre for Enzyme Innovation, University of Portsmouth / Translational Research Laboratory, Portsmouth Hospitals NHS Trust	COVID-19 Genomics UK (COG-UK) Consortium	Angela Beckett,Yann Bourgeois,Garry Scarlett,Sharon Glaysher,Scott Elliott,Kelly Bicknell,Robert Impey,Allyson Lloyd,Sarah Wyllie,Ethan Butcher,Anoop Chauhan,Samuel Robson
EPI_ISL_727991	Virology Department, Sheffield Teaching Hospitals NHS Foundation Trust/Department of Infection, Immunity and Cardiovascular Disease, The Medical School, University of Sheffield	COVID-19 Genomics UK (COG-UK) Consortium	Thushan de Silva, Matthew Parker, Nikki Smith, Adri Angyal, Rebecca Brown, Luke Green, Rachel Tucker, Paul Parsons, Danielle Groves, Katie Johnson, Laura Carrilero, Alex Keeley, Dave Partridge, Matthew Wyles, Benjamin Lindsey, Mehmet Yavuz, Mohammad Raza, Cariad Evans
EPI_ISL_728006, EPI_ISL_728008, EPI_ISL_728038, EPI_ISL_728039, EPI_ISL_728040, EPI_ISL_728047, EPI_ISL_728048, EPI_ISL_728049, EPI_ISL_728094, EPI_ISL_728098, EPI_ISL_728099, EPI_ISL_728100, EPI_ISL_728101, EPI_ISL_728102, EPI_ISL_728108, EPI_ISL_728109, EPI_ISL_728112, EPI_ISL_728113, EPI_ISL_728116, EPI_ISL_728117, EPI_ISL_728118, EPI_ISL_728120, EPI_ISL_728121, EPI_ISL_728122, EPI_ISL_728124, EPI_ISL_728125, EPI_ISL_728126, EPI_ISL_728127, EPI_ISL_728129, EPI_ISL_728133			
see above	University of Wisconsin-Madison AIDS Vaccine Research Laboratories	University of Wisconsin-Madison AIDS Vaccine Research Laboratories	Gage Moreno, Katarina Braun, et al. AIDS Vaccine Research Laboratories
EPI_ISL_728248, EPI_ISL_728249, EPI_ISL_728250, EPI_ISL_728251, EPI_ISL_728252	Institute for Medical Research, Infectious Disease Research Centre, National Institutes of Health, Ministry of Health Malaysia	Institute for Medical Research, Infectious Disease Research Centre, National Institutes of Health, Ministry of Health Malaysia	Suppiah J, Kamel K, Mohd-Zawawi Z, Thayan R
EPI_ISL_728300	Lighthouse Lab in Glasgow	Wellcome Sanger Institute for the COVID-19 Genomics UK (COG-UK) Consortium	Harper VanSteenhouse, Yumi Kasai, David Gray, Carol Clugston, Anna Dominiczak and Alex Alderton, Roberto Amato, Sonia Goncalves, Ewan Harrison, David K. Jackson, Ian Johnston, Dominic Kwiatkowski, Cordelia Langford, John Sillitoe on behalf of the Wellcome Sanger Institute COVID-19 Surveillance Team
EPI_ISL_728311, EPI_ISL_728312, EPI_ISL_728313, EPI_ISL_728314, EPI_ISL_728315, EPI_ISL_728316, EPI_ISL_728317, EPI_ISL_728318, EPI_ISL_728319, EPI_ISL_728320, EPI_ISL_728516, EPI_ISL_728517, EPI_ISL_728518, EPI_ISL_728519, EPI_ISL_728520, EPI_ISL_728521, EPI_ISL_728522, EPI_ISL_728523, EPI_ISL_728524, EPI_ISL_728525, EPI_ISL_728526, EPI_ISL_728527, EPI_ISL_728528, EPI_ISL_728529, EPI_ISL_728530, EPI_ISL_728531, EPI_ISL_728532, EPI_ISL_728533			
see above	CNR Virus des Infections Respiratoires - France SUD	CNR Virus des Infections Respiratoires - France SUD	Antonin Bal, Gregory Destras, Claudia Gonzalez, Gwendolyne Burfin, Quentin Semanas, Martine Valette, Bruno Lina, Laurence Josset
EPI_ISL_728553	University of Wisconsin-Madison AIDS Vaccine Research Laboratories	University of Wisconsin-Madison AIDS Vaccine Research Laboratories	Gage Moreno, Katarina Braun, et al. AIDS Vaccine Research Laboratories
EPI_ISL_728676, EPI_ISL_728677, EPI_ISL_728678, EPI_ISL_728679, EPI_ISL_728680, EPI_ISL_728681, EPI_ISL_728684, EPI_ISL_728713, EPI_ISL_728714, EPI_ISL_728715, EPI_ISL_728716, EPI_ISL_728717, EPI_ISL_728718, EPI_ISL_728719, EPI_ISL_728720, EPI_ISL_728723, EPI_ISL_728724, EPI_ISL_728753, EPI_ISL_728754, EPI_ISL_728755			
see above	Dutch COVID-19 response team	National Institute for Public Health and the Environment (RIVM)	Adam Meijer, Harry Vennema, Jeroen Cremer, Sharon van den Brink, Bas van der Veer, AnneMarie van den Brandt, Florian Zwagemaker, Dennis Schmitz, Chantal Reusken, on behalf of the national COVID-19 response team
EPI_ISL_728968, EPI_ISL_729008	Viollier AG	Department of Biosystems Science and Engineering, ETH Zürich	Chaoran Chen, Sarah Nadeau, Catharine Aquino, Ivan Topolsky, Pedro Ferreira, Philipp Jablonski, Susana Posada-Céspedes, Andreia Cabral de Gouvea, Maria Domenica Moccia, Simon Grüter, Timothy Sykes, Lennart Opitz, Ralph Schlapbach, Christiane Beckmann, Maurice Redondo, Olivier Kobel, Christoph Noppen, Sophie Seidel, Noemie Santamaria de Souza, Niko Beerenwinkel, Tanja Stadler
EPI_ISL_729338, EPI_ISL_729371, EPI_ISL_729443, EPI_ISL_729445, EPI_ISL_729446, EPI_ISL_729467, EPI_ISL_729645, EPI_ISL_729646, EPI_ISL_729647, EPI_ISL_729648, EPI_ISL_729649, EPI_ISL_729650, EPI_ISL_729651, EPI_ISL_729652, EPI_ISL_729671, EPI_ISL_729672, EPI_ISL_729673, EPI_ISL_729674, EPI_ISL_729675, EPI_ISL_729676, EPI_ISL_729677, EPI_ISL_729678, EPI_ISL_729679, EPI_ISL_729680, EPI_ISL_729681, EPI_ISL_729682			
see above	A. Krumbholz, Labor Dr. Krause und Kollegen MVZ GmbH, Kiel	Charité Universitätsmedizin Berlin, Institut für Virologie	Victor M Corman, Barbara Mühlemann, Jörn Beheim-Schwarzbach, Talitha Veith, Julia Schneider, Terry Jones, Christian Drosten
EPI_ISL_731251, EPI_ISL_731252, EPI_ISL_731253	Lighthouse Lab in Milton Keynes	Wellcome Sanger Institute for the COVID-19 Genomics UK (COG-UK) Consortium	The Lighthouse Lab in Milton Keynes and Alex Alderton, Roberto Amato, Sonia Goncalves, Ewan Harrison, David K. Jackson, Ian Johnston, Dominic Kwiatkowski, Cordelia Langford, John Sillitoe on behalf of the Wellcome Sanger Institute COVID-19 Surveillance Team
EPI_ISL_731894	Lighthouse Lab in Glasgow	Wellcome Sanger Institute for the COVID-19 Genomics UK (COG-UK) Consortium	Harper VanSteenhouse, Yumi Kasai, David Gray, Carol Clugston, Anna Dominiczak and Alex Alderton, Roberto Amato, Sonia Goncalves, Ewan Harrison, David K. Jackson, Ian Johnston, Dominic Kwiatkowski, Cordelia Langford, John Sillitoe on behalf of the Wellcome Sanger Institute COVID-19 Surveillance Team

EPI_ISL_731897	Instituto Nacional de Saude (INSA) and Instituto Gulbenkian de Ciencia (IGC)	Instituto Nacional de Saude (INSA) and Instituto Gulbenkian de Ciencia (IGC)	Borges et al
EPI_ISL_731920, EPI_ISL_731921	Instituto Nacional de Saude (INSA)	Instituto Nacional de Saude (INSA)	Borges et al
EPI_ISL_731925, EPI_ISL_731927, EPI_ISL_731928	Instituto Nacional de Saude (INSA) and Instituto Gulbenkian de Ciencia (IGC)	Instituto Nacional de Saude (INSA) and Instituto Gulbenkian de Ciencia (IGC)	Borges et al
EPI_ISL_731951, EPI_ISL_731998, EPI_ISL_731999, EPI_ISL_732008, EPI_ISL_732009, EPI_ISL_732010, EPI_ISL_732011	Instituto Nacional de Saude (INSA)	Instituto Nacional de Saude (INSA)	Borges et al
EPI_ISL_732218, EPI_ISL_732219, EPI_ISL_732220, EPI_ISL_732232, EPI_ISL_732233, EPI_ISL_732234, EPI_ISL_732235, EPI_ISL_732236, EPI_ISL_732237, EPI_ISL_732238, EPI_ISL_732239, EPI_ISL_732240			
see above	Instituto Nacional de Saude (INSA) and Instituto Gulbenkian de Ciencia (IGC)	Instituto Nacional de Saude (INSA) and Instituto Gulbenkian de Ciencia (IGC)	Borges et al
EPI_ISL_732382, EPI_ISL_732383, EPI_ISL_732384, EPI_ISL_732394, EPI_ISL_732403, EPI_ISL_732404, EPI_ISL_732408, EPI_ISL_732409, EPI_ISL_732423, EPI_ISL_732427, EPI_ISL_732445, EPI_ISL_732448, EPI_ISL_732449, EPI_ISL_732451, EPI_ISL_732452, EPI_ISL_732453, EPI_ISL_732493, EPI_ISL_732503, EPI_ISL_732504, EPI_ISL_732505, EPI_ISL_732506, EPI_ISL_732507, EPI_ISL_732508, EPI_ISL_732509			
see above	National Virus Reference Laboratory	National Virus Reference Laboratory	Michael Carr, Gabriel Gonzalez, Jonathan Dean, Daniel Hare, Cillian F De Gascun
EPI_ISL_732686, EPI_ISL_732687, EPI_ISL_732688	CNR Virus des Infections Respiratoires - France SUD	CNR Virus des Infections Respiratoires - France SUD	Antonin Bal, Gregory Destras, Claudia Gonzalez, Gwendolyne Burfin, Quentin Semanas, Martine Valette, Bruno Lina, Laurence Josset
EPI_ISL_732948, EPI_ISL_732949, EPI_ISL_732950, EPI_ISL_732951, EPI_ISL_732952, EPI_ISL_732953, EPI_ISL_732954, EPI_ISL_732955, EPI_ISL_732956, EPI_ISL_732957, EPI_ISL_732958, EPI_ISL_732959			
see above	Department of Tropical Parasitology	Laboratory of Recombinant Vaccines	Lukasz Rabalski, Maciej Kosinski, Teemu Smura, Kirsi Aaltonen, Ravi Kant, Tarja Sironen, Boguslaw Szewczyk, Maciej Grzybek
EPI_ISL_732974, EPI_ISL_732975, EPI_ISL_732976, EPI_ISL_732977, EPI_ISL_732978, EPI_ISL_732979, EPI_ISL_732980, EPI_ISL_732981, EPI_ISL_732982, EPI_ISL_732983, EPI_ISL_732984, EPI_ISL_732985, EPI_ISL_732986, EPI_ISL_732987, EPI_ISL_732988, EPI_ISL_733459, EPI_ISL_733460, EPI_ISL_733461, EPI_ISL_733464, EPI_ISL_733476, EPI_ISL_733477			
see above	WHO National Influenza Centre Russian Federation	WHO National Influenza Centre Russian Federation	Andrey Komissarov, Artem Fadeev, Anna Ivanova, Kseniya Komissarova, Dmitry Bazhenov, Daria Danilenko, Ksenia Safina, Elena Nabieva, Georgii Bazykin, Dmitry Lioznov
EPI_ISL_733492, EPI_ISL_733493	Omsk Research Institute of Natural Focal Infections	WHO National Influenza Centre Russian Federation	Andrey Komissarov, Artem Fadeev, Anna Ivanova, Kseniya Komissarova, Dmitry Bazhenov, Daria Danilenko, Ekaterina Gradoboeva, Elena Poleshchuk, Aleksei Vasilenko, Valery Yakimenko, Ksenia Safina, Elena Nabieva, Georgii Bazykin, Dmitry Lioznov
EPI_ISL_733494, EPI_ISL_733495	WHO National Influenza Centre Russian Federation	WHO National Influenza Centre Russian Federation	Andrey Komissarov, Artem Fadeev, Anna Ivanova, Kseniya Komissarova, Dmitry Bazhenov, Daria Danilenko, Ksenia Safina, Elena Nabieva, Georgii Bazykin, Dmitry Lioznov
EPI_ISL_734170, EPI_ISL_734171, EPI_ISL_734172, EPI_ISL_734173, EPI_ISL_734174, EPI_ISL_734175, EPI_ISL_734176, EPI_ISL_734180, EPI_ISL_734181, EPI_ISL_734182, EPI_ISL_734183, EPI_ISL_734184, EPI_ISL_734185, EPI_ISL_734186, EPI_ISL_734187, EPI_ISL_734188, EPI_ISL_734189, EPI_ISL_734190, EPI_ISL_734191, EPI_ISL_734192, EPI_ISL_734199, EPI_ISL_734200, EPI_ISL_734201, EPI_ISL_734202, EPI_ISL_734203, EPI_ISL_734204, EPI_ISL_734205, EPI_ISL_734206, EPI_ISL_734224, EPI_ISL_734225, EPI_ISL_734254, EPI_ISL_734255, EPI_ISL_734256, EPI_ISL_734269, EPI_ISL_734280, EPI_ISL_734281, EPI_ISL_734282, EPI_ISL_734283, EPI_ISL_734284, EPI_ISL_734285, EPI_ISL_734286			
see above	Virginia Division of Consolidated Laboratory Services (DCLS)	Virginia Division of Consolidated Laboratory Services (DCLS)	Virginia DCLS
EPI_ISL_734481	Masonic Medical Research Institute	Wadsworth Center, New York State Department.of Health	Kirsten St. George, Nathan Tucker, Ryan D. Pfeiffer, Daryl M. Lamson, Alexis Russel, Jonathan Plitnick, Navjot Singh, John Kelly, Sara Griesemer, Erasmus Schneider, Erica Lasek-Nesselquist
EPI_ISL_736936, EPI_ISL_736937	Netcare	KRISP, KZN Research Innovation and Sequencing Platform	Giandhari J, Pillay S, Lessells R, ChimukangaraB, Mdlalose K, York D, Khan S, Tegally H, Wilkinson E, de Oliveira T
EPI_ISL_736960	NHLS-IALCH	KRISP, KZN Research Innovation and Sequencing Platform	Giandhari J, Pillay S, Lessells R, ChimukangaraB, Mdlalose K, York D, Khan S, Tegally H, Wilkinson E, de Oliveira T
EPI_ISL_736961, EPI_ISL_736962, EPI_ISL_736963, EPI_ISL_736964, EPI_ISL_736965	Netcare	KRISP, KZN Research Innovation and Sequencing Platform	Giandhari J, Pillay S, Lessells R, ChimukangaraB, Mdlalose K, York D, Khan S, Tegally H, Wilkinson E, de Oliveira T
EPI_ISL_737154, EPI_ISL_737160, EPI_ISL_737163, EPI_ISL_737164, EPI_ISL_737169, EPI_ISL_737178, EPI_ISL_737179, EPI_ISL_737180, EPI_ISL_737182, EPI_ISL_737193, EPI_ISL_737264, EPI_ISL_737265, EPI_ISL_737268, EPI_ISL_737269, EPI_ISL_737281, EPI_ISL_737284, EPI_ISL_737287, EPI_ISL_737290, EPI_ISL_737299, EPI_ISL_737306, EPI_ISL_737307			
see above	Michigan Department of Health and Human Services, Bureau of Laboratories	Michigan Department of Health and Human Services, Bureau of Laboratories	Blankenship HM, Riner D, Soehnlen MK
EPI_ISL_738503	Orange County Public Health Lab	Chan-Zuckerberg Biohub	CZB Cliahub Consortium
EPI_ISL_738504	Humboldt County Public Health Laboratory	Chan-Zuckerberg Biohub	CZB Cliahub Consortium
EPI_ISL_738516, EPI_ISL_738532	Santa Clara County Public Health Laboratory	Chan-Zuckerberg Biohub	CZB Cliahub Consortium
EPI_ISL_738539	Humboldt County Public Health Laboratory	Chan-Zuckerberg Biohub	CZB Cliahub Consortium
EPI_ISL_738556	Santa Clara County Public Health Laboratory	Chan-Zuckerberg Biohub	CZB Cliahub Consortium
EPI_ISL_738559	Orange County Public Health Lab	Chan-Zuckerberg Biohub	CZB Cliahub Consortium
EPI_ISL_738563	Humboldt County Public Health Laboratory	Chan-Zuckerberg Biohub	CZB Cliahub Consortium
EPI_ISL_738568, EPI_ISL_738581, EPI_ISL_738586, EPI_ISL_738590	Santa Clara County Public Health Laboratory	Chan-Zuckerberg Biohub	CZB Cliahub Consortium
EPI_ISL_738594, EPI_ISL_738599	Orange County Public Health Lab	Chan-Zuckerberg Biohub	CZB Cliahub Consortium
EPI_ISL_738601	Santa Clara County Public Health Laboratory	Chan-Zuckerberg Biohub	CZB Cliahub Consortium
EPI_ISL_738603	Orange County Public Health Lab	Chan-Zuckerberg Biohub	CZB Cliahub Consortium
EPI_ISL_738612, EPI_ISL_738618	Santa Clara County Public Health Laboratory	Chan-Zuckerberg Biohub	CZB Cliahub Consortium
EPI_ISL_738621, EPI_ISL_738632	Orange County Public Health Lab	Chan-Zuckerberg Biohub	CZB Cliahub Consortium
EPI_ISL_738645, EPI_ISL_738670, EPI_ISL_738681, EPI_ISL_738682, EPI_ISL_738685, EPI_ISL_738691	Santa Clara County Public Health Laboratory	Chan-Zuckerberg Biohub	CZB Cliahub Consortium
EPI_ISL_738693, EPI_ISL_738694	Orange County Public Health Lab	Chan-Zuckerberg Biohub	CZB Cliahub Consortium
EPI_ISL_738698, EPI_ISL_738701	Santa Clara County Public Health Laboratory	Chan-Zuckerberg Biohub	CZB Cliahub Consortium
EPI_ISL_738709	Orange County Public Health Lab	Chan-Zuckerberg Biohub	CZB Cliahub Consortium
EPI_ISL_738710	Santa Clara County Public Health Laboratory	Chan-Zuckerberg Biohub	CZB Cliahub Consortium
EPI_ISL_738722, EPI_ISL_738729, EPI_ISL_738755, EPI_ISL_738761	Orange County Public Health Lab	Chan-Zuckerberg Biohub	CZB Cliahub Consortium
EPI_ISL_738769, EPI_ISL_738771, EPI_ISL_738780, EPI_ISL_738798	Santa Clara County Public Health Laboratory	Chan-Zuckerberg Biohub	CZB Cliahub Consortium

EPI_ISL_738799	Orange County Public Health Lab	Chan-Zuckerberg Biohub	CZB Cliahub Consortium
EPI_ISL_738801, EPI_ISL_738806, EPI_ISL_738807	Humboldt County Public Health Laboratory	Chan-Zuckerberg Biohub	CZB Cliahub Consortium
EPI_ISL_738811, EPI_ISL_738818, EPI_ISL_738824, EPI_ISL_738830	Orange County Public Health Lab	Chan-Zuckerberg Biohub	CZB Cliahub Consortium
EPI_ISL_738842	Santa Clara County Public Health Laboratory	Chan-Zuckerberg Biohub	CZB Cliahub Consortium
EPI_ISL_738845	Orange County Public Health Lab	Chan-Zuckerberg Biohub	CZB Cliahub Consortium
EPI_ISL_738849	Humboldt County Public Health Laboratory	Chan-Zuckerberg Biohub	CZB Cliahub Consortium
EPI_ISL_738860, EPI_ISL_738862	Orange County Public Health Lab	Chan-Zuckerberg Biohub	CZB Cliahub Consortium
EPI_ISL_738864	Santa Clara County Public Health Laboratory	Chan-Zuckerberg Biohub	CZB Cliahub Consortium
EPI_ISL_738865	Humboldt County Public Health Laboratory	Chan-Zuckerberg Biohub	CZB Cliahub Consortium
EPI_ISL_738867	Santa Clara County Public Health Laboratory	Chan-Zuckerberg Biohub	CZB Cliahub Consortium
EPI_ISL_738869, EPI_ISL_738873	Orange County Public Health Lab	Chan-Zuckerberg Biohub	CZB Cliahub Consortium
EPI_ISL_738875	Humboldt County Public Health Laboratory	Chan-Zuckerberg Biohub	CZB Cliahub Consortium
EPI_ISL_738884	Orange County Public Health Lab	Chan-Zuckerberg Biohub	CZB Cliahub Consortium
EPI_ISL_738887	Santa Clara County Public Health Laboratory	Chan-Zuckerberg Biohub	CZB Cliahub Consortium
EPI_ISL_738902	Humboldt County Public Health Laboratory	Chan-Zuckerberg Biohub	CZB Cliahub Consortium
EPI_ISL_738903	Santa Clara County Public Health Laboratory	Chan-Zuckerberg Biohub	CZB Cliahub Consortium
EPI_ISL_738905	Orange County Public Health Lab	Chan-Zuckerberg Biohub	CZB Cliahub Consortium
EPI_ISL_738917	Humboldt County Public Health Laboratory	Chan-Zuckerberg Biohub	CZB Cliahub Consortium
EPI_ISL_738921	Orange County Public Health Lab	Chan-Zuckerberg Biohub	CZB Cliahub Consortium
EPI_ISL_738924	Santa Clara County Public Health Laboratory	Chan-Zuckerberg Biohub	CZB Cliahub Consortium
EPI_ISL_738935	Orange County Public Health Lab	Chan-Zuckerberg Biohub	CZB Cliahub Consortium
EPI_ISL_738936	Santa Clara County Public Health Laboratory	Chan-Zuckerberg Biohub	CZB Cliahub Consortium
EPI_ISL_738938	Humboldt County Public Health Laboratory	Chan-Zuckerberg Biohub	CZB Cliahub Consortium
EPI_ISL_738944	Orange County Public Health Lab	Chan-Zuckerberg Biohub	CZB Cliahub Consortium
EPI_ISL_738960, EPI_ISL_738965	Santa Clara County Public Health Laboratory	Chan-Zuckerberg Biohub	CZB Cliahub Consortium
EPI_ISL_738966, EPI_ISL_738968	Orange County Public Health Lab	Chan-Zuckerberg Biohub	CZB Cliahub Consortium
EPI_ISL_738969	Humboldt County Public Health Laboratory	Chan-Zuckerberg Biohub	CZB Cliahub Consortium
EPI_ISL_738970, EPI_ISL_738973, EPI_ISL_738975	Orange County Public Health Lab	Chan-Zuckerberg Biohub	CZB Cliahub Consortium
EPI_ISL_738980, EPI_ISL_739000, EPI_ISL_739001, EPI_ISL_739007	Humboldt County Public Health Laboratory	Chan-Zuckerberg Biohub	CZB Cliahub Consortium
EPI_ISL_739015, EPI_ISL_739026	Santa Clara County Public Health Laboratory	Chan-Zuckerberg Biohub	CZB Cliahub Consortium
EPI_ISL_739028	Orange County Public Health Lab	Chan-Zuckerberg Biohub	CZB Cliahub Consortium
EPI_ISL_739031	Santa Clara County Public Health Laboratory	Chan-Zuckerberg Biohub	CZB Cliahub Consortium
EPI_ISL_739043	Humboldt County Public Health Laboratory	Chan-Zuckerberg Biohub	CZB Cliahub Consortium
EPI_ISL_739048, EPI_ISL_739055	Orange County Public Health Lab	Chan-Zuckerberg Biohub	CZB Cliahub Consortium
EPI_ISL_739060	Santa Clara County Public Health Laboratory	Chan-Zuckerberg Biohub	CZB Cliahub Consortium
EPI_ISL_739063, EPI_ISL_739071	Orange County Public Health Lab	Chan-Zuckerberg Biohub	CZB Cliahub Consortium
EPI_ISL_739074	Santa Clara County Public Health Laboratory	Chan-Zuckerberg Biohub	CZB Cliahub Consortium
EPI_ISL_739078	Orange County Public Health Lab	Chan-Zuckerberg Biohub	CZB Cliahub Consortium
EPI_ISL_739091	Santa Clara County Public Health Laboratory	Chan-Zuckerberg Biohub	CZB Cliahub Consortium
EPI_ISL_739092, EPI_ISL_739099	Orange County Public Health Lab	Chan-Zuckerberg Biohub	CZB Cliahub Consortium
EPI_ISL_739103	Santa Clara County Public Health Laboratory	Chan-Zuckerberg Biohub	CZB Cliahub Consortium
EPI_ISL_739112, EPI_ISL_739114, EPI_ISL_739123	Humboldt County Public Health Laboratory	Chan-Zuckerberg Biohub	CZB Cliahub Consortium
EPI_ISL_739147	Santa Clara County Public Health Laboratory	Chan-Zuckerberg Biohub	CZB Cliahub Consortium
EPI_ISL_739162	Orange County Public Health Lab	Chan-Zuckerberg Biohub	CZB Cliahub Consortium
EPI_ISL_739170	Humboldt County Public Health Laboratory	Chan-Zuckerberg Biohub	CZB Cliahub Consortium
EPI_ISL_739195, EPI_ISL_739204	Santa Clara County Public Health Laboratory	Chan-Zuckerberg Biohub	CZB Cliahub Consortium
EPI_ISL_739205	Orange County Public Health Lab	Chan-Zuckerberg Biohub	CZB Cliahub Consortium
EPI_ISL_739210	Humboldt County Public Health Laboratory	Chan-Zuckerberg Biohub	CZB Cliahub Consortium
EPI_ISL_739227	Orange County Public Health Lab	Chan-Zuckerberg Biohub	CZB Cliahub Consortium
EPI_ISL_739228	Humboldt County Public Health Laboratory	Chan-Zuckerberg Biohub	CZB Cliahub Consortium
EPI_ISL_739230	Orange County Public Health Lab	Chan-Zuckerberg Biohub	CZB Cliahub Consortium
EPI_ISL_739232	Santa Clara County Public Health Laboratory	Chan-Zuckerberg Biohub	CZB Cliahub Consortium
EPI_ISL_739238	Orange County Public Health Lab	Chan-Zuckerberg Biohub	CZB Cliahub Consortium
EPI_ISL_739240, EPI_ISL_739242	Humboldt County Public Health Laboratory	Chan-Zuckerberg Biohub	CZB Cliahub Consortium
EPI_ISL_739246, EPI_ISL_739253	Santa Clara County Public Health Laboratory	Chan-Zuckerberg Biohub	CZB Cliahub Consortium

EPI_ISL_739255, EPI_ISL_739271	Orange County Public Health Lab	Chan-Zuckerberg Biohub	CZB Cliahub Consortium
EPI_ISL_739274	Humboldt County Public Health Laboratory	Chan-Zuckerberg Biohub	CZB Cliahub Consortium
EPI_ISL_739275	Santa Clara County Public Health Laboratory	Chan-Zuckerberg Biohub	CZB Cliahub Consortium
EPI_ISL_739276	Orange County Public Health Lab	Chan-Zuckerberg Biohub	CZB Cliahub Consortium
EPI_ISL_739284	Santa Clara County Public Health Laboratory	Chan-Zuckerberg Biohub	CZB Cliahub Consortium
EPI_ISL_739287	Humboldt County Public Health Laboratory	Chan-Zuckerberg Biohub	CZB Cliahub Consortium
EPI_ISL_739289	Orange County Public Health Lab	Chan-Zuckerberg Biohub	CZB Cliahub Consortium
EPI_ISL_739293	Santa Clara County Public Health Laboratory	Chan-Zuckerberg Biohub	CZB Cliahub Consortium
EPI_ISL_739296	Orange County Public Health Lab	Chan-Zuckerberg Biohub	CZB Cliahub Consortium
EPI_ISL_739305	Santa Clara County Public Health Laboratory	Chan-Zuckerberg Biohub	CZB Cliahub Consortium
EPI_ISL_739313	Humboldt County Public Health Laboratory	Chan-Zuckerberg Biohub	CZB Cliahub Consortium
EPI_ISL_739327	Santa Clara County Public Health Laboratory	Chan-Zuckerberg Biohub	CZB Cliahub Consortium
EPI_ISL_739331	Orange County Public Health Lab	Chan-Zuckerberg Biohub	CZB Cliahub Consortium
EPI_ISL_739332	Santa Clara County Public Health Laboratory	Chan-Zuckerberg Biohub	CZB Cliahub Consortium
EPI_ISL_739334	Orange County Public Health Lab	Chan-Zuckerberg Biohub	CZB Cliahub Consortium
EPI_ISL_739335, EPI_ISL_739343	Santa Clara County Public Health Laboratory	Chan-Zuckerberg Biohub	CZB Cliahub Consortium
EPI_ISL_739345, EPI_ISL_739350	Orange County Public Health Lab	Chan-Zuckerberg Biohub	CZB Cliahub Consortium
EPI_ISL_739352	Santa Clara County Public Health Laboratory	Chan-Zuckerberg Biohub	CZB Cliahub Consortium
EPI_ISL_739353, EPI_ISL_739362	Orange County Public Health Lab	Chan-Zuckerberg Biohub	CZB Cliahub Consortium
EPI_ISL_739365	Santa Clara County Public Health Laboratory	Chan-Zuckerberg Biohub	CZB Cliahub Consortium
EPI_ISL_739373	Orange County Public Health Lab	Chan-Zuckerberg Biohub	CZB Cliahub Consortium
EPI_ISL_739376	Santa Clara County Public Health Laboratory	Chan-Zuckerberg Biohub	CZB Cliahub Consortium
EPI_ISL_739379	Orange County Public Health Lab	Chan-Zuckerberg Biohub	CZB Cliahub Consortium
EPI_ISL_739380	Santa Clara County Public Health Laboratory	Chan-Zuckerberg Biohub	CZB Cliahub Consortium
EPI_ISL_739389	Humboldt County Public Health Laboratory	Chan-Zuckerberg Biohub	CZB Cliahub Consortium
EPI_ISL_739397	Orange County Public Health Lab	Chan-Zuckerberg Biohub	CZB Cliahub Consortium
EPI_ISL_739403	Humboldt County Public Health Laboratory	Chan-Zuckerberg Biohub	CZB Cliahub Consortium
EPI_ISL_739404	Santa Clara County Public Health Laboratory	Chan-Zuckerberg Biohub	CZB Cliahub Consortium
EPI_ISL_739405	Humboldt County Public Health Laboratory	Chan-Zuckerberg Biohub	CZB Cliahub Consortium
EPI_ISL_739409	Santa Clara County Public Health Laboratory	Chan-Zuckerberg Biohub	CZB Cliahub Consortium
EPI_ISL_739410, EPI_ISL_739412	Orange County Public Health Lab	Chan-Zuckerberg Biohub	CZB Cliahub Consortium
EPI_ISL_739417, EPI_ISL_739425	Humboldt County Public Health Laboratory	Chan-Zuckerberg Biohub	CZB Cliahub Consortium
EPI_ISL_739428	Orange County Public Health Lab	Chan-Zuckerberg Biohub	CZB Cliahub Consortium
EPI_ISL_739436	Humboldt County Public Health Laboratory	Chan-Zuckerberg Biohub	CZB Cliahub Consortium
EPI_ISL_739438	Orange County Public Health Lab	Chan-Zuckerberg Biohub	CZB Cliahub Consortium
EPI_ISL_739453	Santa Clara County Public Health Laboratory	Chan-Zuckerberg Biohub	CZB Cliahub Consortium
EPI_ISL_739461	Humboldt County Public Health Laboratory	Chan-Zuckerberg Biohub	CZB Cliahub Consortium
EPI_ISL_739465	Orange County Public Health Lab	Chan-Zuckerberg Biohub	CZB Cliahub Consortium
EPI_ISL_739475	Humboldt County Public Health Laboratory	Chan-Zuckerberg Biohub	CZB Cliahub Consortium
EPI_ISL_739476, EPI_ISL_739479, EPI_ISL_739481	Santa Clara County Public Health Laboratory	Chan-Zuckerberg Biohub	CZB Cliahub Consortium
EPI_ISL_739482, EPI_ISL_739493	Orange County Public Health Lab	Chan-Zuckerberg Biohub	CZB Cliahub Consortium
EPI_ISL_739502, EPI_ISL_739510, EPI_ISL_739521	Santa Clara County Public Health Laboratory	Chan-Zuckerberg Biohub	CZB Cliahub Consortium
EPI_ISL_739531	Humboldt County Public Health Laboratory	Chan-Zuckerberg Biohub	CZB Cliahub Consortium
EPI_ISL_739532, EPI_ISL_739534	Orange County Public Health Lab	Chan-Zuckerberg Biohub	CZB Cliahub Consortium
EPI_ISL_739542	Humboldt County Public Health Laboratory	Chan-Zuckerberg Biohub	CZB Cliahub Consortium
EPI_ISL_739543	Santa Clara County Public Health Laboratory	Chan-Zuckerberg Biohub	CZB Cliahub Consortium
EPI_ISL_739547	Orange County Public Health Lab	Chan-Zuckerberg Biohub	CZB Cliahub Consortium
EPI_ISL_739560	Santa Clara County Public Health Laboratory	Chan-Zuckerberg Biohub	CZB Cliahub Consortium
EPI_ISL_739568, EPI_ISL_739570, EPI_ISL_739573	Orange County Public Health Lab	Chan-Zuckerberg Biohub	CZB Cliahub Consortium
EPI_ISL_739574	Santa Clara County Public Health Laboratory	Chan-Zuckerberg Biohub	CZB Cliahub Consortium
EPI_ISL_739582, EPI_ISL_739591	Orange County Public Health Lab	Chan-Zuckerberg Biohub	CZB Cliahub Consortium
EPI_ISL_739596	Santa Clara County Public Health Laboratory	Chan-Zuckerberg Biohub	CZB Cliahub Consortium
EPI_ISL_739611	Humboldt County Public Health Laboratory	Chan-Zuckerberg Biohub	CZB Cliahub Consortium
EPI_ISL_739612	Santa Clara County Public Health Laboratory	Chan-Zuckerberg Biohub	CZB Cliahub Consortium
EPI_ISL_739614	Orange County Public Health Lab	Chan-Zuckerberg Biohub	CZB Cliahub Consortium
EPI_ISL_739624	Santa Clara County Public Health Laboratory	Chan-Zuckerberg Biohub	CZB Cliahub Consortium
EPI_ISL_739625, EPI_ISL_739627,	Orange County Public Health Lab	Chan-Zuckerberg Biohub	CZB Cliahub Consortium

EPI_ISL_739636			
EPI_ISL_739651	Humboldt County Public Health Laboratory	Chan-Zuckerberg Biohub	CZB Cliahub Consortium
EPI_ISL_739706, EPI_ISL_739707, EPI_ISL_739708, EPI_ISL_739710, EPI_ISL_739719, EPI_ISL_739741, EPI_ISL_739752, EPI_ISL_739772, EPI_ISL_739775, EPI_ISL_739805, EPI_ISL_739811, EPI_ISL_739842, EPI_ISL_739876, EPI_ISL_739879, EPI_ISL_739887, EPI_ISL_739888, EPI_ISL_739893, EPI_ISL_739899, EPI_ISL_739932, EPI_ISL_739938, EPI_ISL_739942, EPI_ISL_739959, EPI_ISL_739979, EPI_ISL_739989, EPI_ISL_740009, EPI_ISL_740035, EPI_ISL_740065, EPI_ISL_740075, EPI_ISL_740088, EPI_ISL_740089, EPI_ISL_740102, EPI_ISL_740112, EPI_ISL_740128, EPI_ISL_740143, EPI_ISL_740151, EPI_ISL_740152, EPI_ISL_740157, EPI_ISL_740164, EPI_ISL_740183, EPI_ISL_740216, EPI_ISL_740235, EPI_ISL_740253, EPI_ISL_740255, EPI_ISL_740285, EPI_ISL_740297, EPI_ISL_740303, EPI_ISL_740308, EPI_ISL_740311, EPI_ISL_740312, EPI_ISL_740315, EPI_ISL_740350, EPI_ISL_740367, EPI_ISL_740369, EPI_ISL_740379, EPI_ISL_740422, EPI_ISL_740426, EPI_ISL_740459, EPI_ISL_740465, EPI_ISL_740476, EPI_ISL_740483, EPI_ISL_740484, EPI_ISL_740517, EPI_ISL_740524			
see above	Laboratoire national de santé, Microbiology, Virology	Laboratoire national de santé, Microbiology, Microbial Genomics Platform	Anke Wienecke-Baldacchino, Catherine Ragimbeau,Jessica Tapp, Fatu Djabi, Lise Pignon, Raoul Salmon, Tamir Abdelrahman
EPI_ISL_741516	Quadram Institute Bioscience	COVID-19 Genomics UK (COG-UK) Consortium	Dave J. Baker, Gemma L. Kay, Alp Aydin, Thanh Le-Viet, Steven Rudder, Ana P. Tedim, Anastasia Kolyva, Maria Diaz, Leonardo de Oliveira Martins, Nabil-Fareed Alikhan, Lizzie Meadows, Rachael Stanley, Ngozi Elumogo, Muhammed Yasir, Nicholas M. Thomson, Alexander J Trotter, Rachel Gilroy, Samuel Bloomfield, Claire Stuart, Andrew Bell, Reenesh Prakash, Samir Dervisevic, Alison E. Mather, John Wain, Mark Webber, Andrew J. Page, Justin O'Grady
EPI_ISL_741716, EPI_ISL_741717, EPI_ISL_741718, EPI_ISL_741719, EPI_ISL_741740, EPI_ISL_741741	Centre for Enzyme Innovation, University of Portsmouth / Translational Research Laboratory, Portsmouth Hospitals NHS Trust	COVID-19 Genomics UK (COG-UK) Consortium	Angela Beckett,Yann Bourgeois,Garry Scarlett,Sharon Glaysher,Scott Elliott,Kelly Bicknell,Robert Impey,Allyson Lloyd,Sarah Wyllie,Ethan Butcher,Anoop Chauhan,Samuel Robson
EPI_ISL_742541, EPI_ISL_742542, EPI_ISL_742543, EPI_ISL_742544, EPI_ISL_742547, EPI_ISL_742548, EPI_ISL_742549, EPI_ISL_742559, EPI_ISL_742561, EPI_ISL_742563, EPI_ISL_742564, EPI_ISL_742585, EPI_ISL_742586, EPI_ISL_742588, EPI_ISL_742589, EPI_ISL_742590, EPI_ISL_742591, EPI_ISL_742592, EPI_ISL_742593, EPI_ISL_742594, EPI_ISL_742595, EPI_ISL_742596, EPI_ISL_742597, EPI_ISL_742598, EPI_ISL_742599, EPI_ISL_742600, EPI_ISL_742601, EPI_ISL_742602, EPI_ISL_742603, EPI_ISL_742604, EPI_ISL_742605, EPI_ISL_742606, EPI_ISL_742607, EPI_ISL_742608, EPI_ISL_742609, EPI_ISL_742610, EPI_ISL_742611, EPI_ISL_742612, EPI_ISL_742613, EPI_ISL_742614, EPI_ISL_742615, EPI_ISL_742616, EPI_ISL_742617, EPI_ISL_742618, EPI_ISL_742619, EPI_ISL_742620, EPI_ISL_742621, EPI_ISL_742622, EPI_ISL_742623, EPI_ISL_742624, EPI_ISL_742625, EPI_ISL_742626, EPI_ISL_742627, EPI_ISL_742628, EPI_ISL_742629, EPI_ISL_742630, EPI_ISL_742631, EPI_ISL_742632, EPI_ISL_742633, EPI_ISL_742634, EPI_ISL_742635, EPI_ISL_742636			
see above	Wales Specialist Virology Centre Sequencing lab: Pathogen Genomics Unit	COVID-19 Genomics UK (COG-UK) Consortium	Catherine Moore, Johnathan Evans, Laura Gifford, Malorie Perry, Simon Cottrell, Angela Marchbank, Alec Birchley, Alexander Adams, Amy Gaskin, Bree Gatica-Wilcox, Jason Coombes, Joel Southgate, Lauren Gilbert, Lee Graham, Nicole Pacchiarini, Sara Kumziene-Summerhayes, Sarah Taylor, Sophie Jones, Sara Rey, Matthew Bull, Joanne Watkins, Sally Corden, Tom Connor
EPI_ISL_744137, EPI_ISL_744148, EPI_ISL_744177, EPI_ISL_744179, EPI_ISL_744186, EPI_ISL_744244, EPI_ISL_744248, EPI_ISL_744250, EPI_ISL_744251, EPI_ISL_744254, EPI_ISL_744256, EPI_ISL_744263, EPI_ISL_744268, EPI_ISL_744271, EPI_ISL_744277, EPI_ISL_744310, EPI_ISL_744329, EPI_ISL_744336, EPI_ISL_744349, EPI_ISL_744353, EPI_ISL_744407, EPI_ISL_744415, EPI_ISL_744422, EPI_ISL_744424, EPI_ISL_744426, EPI_ISL_744453, EPI_ISL_744462, EPI_ISL_744476, EPI_ISL_744494, EPI_ISL_744499, EPI_ISL_744526, EPI_ISL_744545, EPI_ISL_744562, EPI_ISL_744603, EPI_ISL_744614, EPI_ISL_744629, EPI_ISL_744648, EPI_ISL_744656, EPI_ISL_744657, EPI_ISL_744667, EPI_ISL_744692, EPI_ISL_744704, EPI_ISL_744708, EPI_ISL_744740, EPI_ISL_744776, EPI_ISL_744781, EPI_ISL_744795, EPI_ISL_744797, EPI_ISL_744802, EPI_ISL_744834, EPI_ISL_744844, EPI_ISL_744853, EPI_ISL_744880, EPI_ISL_744923, EPI_ISL_744933, EPI_ISL_744953, EPI_ISL_744957, EPI_ISL_744982, EPI_ISL_744983, EPI_ISL_745000, EPI_ISL_745003, EPI_ISL_745021, EPI_ISL_745026			
see above	Laboratoire national de santé, Microbiology, Virology	Laboratoire national de santé, Microbiology, Microbial Genomics Platform	Anke Wienecke-Baldacchino, Catherine Ragimbeau,Jessica Tapp, Fatu Djabi, Lise Pignon, Raoul Salmon, Tamir Abdelrahman
EPI_ISL_745399	DOHMH Morrisania	New York City Public Health Laboratory	Jade Wang, et al.
EPI_ISL_745404	DOHMH Central Harlem	New York City Public Health Laboratory	Jade Wang, et al.
EPI_ISL_746878, EPI_ISL_746965, EPI_ISL_746969, EPI_ISL_746982, EPI_ISL_746996	Utah Public Health Laboratory	Utah Public Health Laboratory	Erin Young, Kelly Oakeson, Tara Gallagher
EPI_ISL_747155, EPI_ISL_747156, EPI_ISL_747157, EPI_ISL_747158, EPI_ISL_747159, EPI_ISL_747160, EPI_ISL_747161, EPI_ISL_747162, EPI_ISL_747163, EPI_ISL_747164, EPI_ISL_747165, EPI_ISL_747166, EPI_ISL_747167, EPI_ISL_747169, EPI_ISL_747179, EPI_ISL_747180, EPI_ISL_747191, EPI_ISL_747193			
see above	Respiratory Viruses Branch, Centers for Disease Control and Prevention	Respiratory Viruses Branch, Centers for Disease Control and Prevention	Queen,K., Li,Y., Tao,Y., Uehara,A., Montmayeur,A., Paden,C.R., Cook,P.W., Marine,R., Sheth,M., Wang,H., Lee,J., Tong,S.
EPI_ISL_747384, EPI_ISL_747386, EPI_ISL_747392, EPI_ISL_747393, EPI_ISL_747397, EPI_ISL_747400, EPI_ISL_747401, EPI_ISL_747402, EPI_ISL_747439, EPI_ISL_747446, EPI_ISL_747447, EPI_ISL_747448, EPI_ISL_747451, EPI_ISL_747452			
see above	Division of Emerging Infectious Diseases, Bureau of Infectious Diseases Diagnosis Control, Korea Disease Control and Prevention Agency	Division of Emerging Infectious Diseases, Bureau of Infectious Diseases Diagnosis Control, Korea Disease Control and Prevention Agency	Ae Kyung Park, Il-Hwan Kim, Heui Man Kim, Jeong-Min Kim, Namjoo Lee, Chaeyoung Lee, Sang Hee Woo, Eun-Jin Kim
EPI_ISL_747588, EPI_ISL_747604, EPI_ISL_747605, EPI_ISL_747649, EPI_ISL_747653, EPI_ISL_747720, EPI_ISL_747744, EPI_ISL_747751, EPI_ISL_748113, EPI_ISL_748114, EPI_ISL_748115, EPI_ISL_748116, EPI_ISL_748117, EPI_ISL_748118			
see above	Department of Virus and Microbiological Special Diagnostics, Statens Serum Institut, Copenhagen, Denmark	Albertsen Lab, Department of Chemistry and Bioscience, Aalborg University, Denmark	Danish Covid-19 Genome Consortium
EPI_ISL_751454, EPI_ISL_751455, EPI_ISL_751456, EPI_ISL_751457, EPI_ISL_751463, EPI_ISL_751465, EPI_ISL_751474, EPI_ISL_751475, EPI_ISL_751476	CHU Purpan - Laboratoire de Virologie - Institut Fédératif de Biologie	CHU Purpan - Laboratoire de Virologie - Institut Fédératif de Biologie	Latour J., Ranger N., Dubois M., Carcenac R., Harter A., Boyer P., Tremeaux P., Izopet J.
EPI_ISL_751546	VA-Division of Consolidated Laboratory Services	Genomics and Discovery, Respiratory Viruses Branch, Division of Viral Diseases, Centers for Disease Control and Prevention	Krista Queen, Yan Li, Ying Tao, Jing Zhang, Anna Uehara, Anna Montmayeur, Clinton R. Paden, Peter W. Cook,Rachel Marine, Mili Sheth, Haibin Wang, Justin Lee, Suxiang Tong
EPI_ISL_751558	NH Dept. of Health and Human Services Public Health Labs	Genomics and Discovery, Respiratory Viruses Branch, Division of Viral Diseases, Centers for Disease Control and Prevention	Krista Queen, Yan Li, Ying Tao, Jing Zhang, Anna Uehara, Anna Montmayeur, Clinton R. Paden, Peter W. Cook,Rachel Marine, Mili Sheth, Haibin Wang, Justin Lee, Suxiang Tong
EPI_ISL_751560	NYSDOH Wadsworth Center, Virology Lab	Genomics and Discovery, Respiratory Viruses Branch, Division of Viral Diseases, Centers for Disease Control and Prevention	Krista Queen, Yan Li, Ying Tao, Jing Zhang, Anna Uehara, Anna Montmayeur, Clinton R. Paden, Peter W. Cook,Rachel Marine, Mili Sheth, Haibin Wang, Justin Lee, Suxiang Tong
EPI_ISL_751564	VA-Division of Consolidated Laboratory Services	Genomics and Discovery, Respiratory Viruses Branch, Division of Viral Diseases, Centers for Disease Control and Prevention	Krista Queen, Yan Li, Ying Tao, Jing Zhang, Anna Uehara, Anna Montmayeur, Clinton R. Paden, Peter W. Cook,Rachel Marine, Mili Sheth, Haibin Wang, Justin Lee, Suxiang Tong
EPI_ISL_751576	MN PHL Division, Minnesota Department of Health	Genomics and Discovery, Respiratory Viruses Branch, Division of Viral Diseases, Centers for Disease Control and Prevention	Krista Queen, Yan Li, Ying Tao, Jing Zhang, Anna Uehara, Anna Montmayeur, Clinton R. Paden, Peter W. Cook,Rachel Marine, Mili Sheth, Haibin Wang, Justin Lee, Suxiang Tong
EPI_ISL_751588	VA-Division of Consolidated Laboratory Services	Genomics and Discovery, Respiratory Viruses Branch, Division of Viral Diseases, Centers for Disease Control and Prevention	Krista Queen, Yan Li, Ying Tao, Jing Zhang, Anna Uehara, Anna Montmayeur, Clinton R. Paden, Peter W. Cook,Rachel Marine, Mili Sheth, Haibin Wang, Justin Lee, Suxiang Tong
EPI_ISL_751590	ID Bureau of Laboratories	Genomics and Discovery, Respiratory Viruses Branch, Division of Viral Diseases, Centers for Disease Control and Prevention	Krista Queen, Yan Li, Ying Tao, Jing Zhang, Anna Uehara, Anna Montmayeur, Clinton R. Paden, Peter W. Cook,Rachel Marine, Mili Sheth, Haibin Wang, Justin Lee, Suxiang Tong
EPI_ISL_751592, EPI_ISL_751598	AK State Public Health Lab, State Health Department	Genomics and Discovery, Respiratory Viruses Branch, Division of Viral Diseases, Centers for Disease Control and Prevention	Krista Queen, Yan Li, Ying Tao, Jing Zhang, Anna Uehara, Anna Montmayeur, Clinton R. Paden, Peter W. Cook,Rachel Marine, Mili Sheth, Haibin Wang, Justin Lee, Suxiang Tong
EPI_ISL_751602	DE Public Health Laboratory	Genomics and Discovery, Respiratory Viruses Branch,	Krista Queen, Yan Li, Ying Tao, Jing Zhang, Anna Uehara, Anna Montmayeur, Clinton R. Paden, Peter W. Cook,Rachel Marine, Mili Sheth, Haibin Wang,

		Division of Viral Diseases, Centers for Disease Control and Prevention	Justin Lee, Suxiang Tong
EPI_ISL_751610	OH Department of Health Laboratory	Genomics and Discovery, Respiratory Viruses Branch, Division of Viral Diseases, Centers for Disease Control and Prevention	Krista Queen, Yan Li, Ying Tao, Jing Zhang, Anna Uehara, Anna Montmayeur, Clinton R. Paden, Peter W. Cook,Rachel Marine, Mili Sheth, Haibin Wang, Justin Lee, Suxiang Tong
EPI_ISL_751612	ID Bureau of Laboratories	Genomics and Discovery, Respiratory Viruses Branch, Division of Viral Diseases, Centers for Disease Control and Prevention	Krista Queen, Yan Li, Ying Tao, Jing Zhang, Anna Uehara, Anna Montmayeur, Clinton R. Paden, Peter W. Cook,Rachel Marine, Mili Sheth, Haibin Wang, Justin Lee, Suxiang Tong
EPI_ISL_751613	OH Department of Health Laboratory	Genomics and Discovery, Respiratory Viruses Branch, Division of Viral Diseases, Centers for Disease Control and Prevention	Krista Queen, Yan Li, Ying Tao, Jing Zhang, Anna Uehara, Anna Montmayeur, Clinton R. Paden, Peter W. Cook,Rachel Marine, Mili Sheth, Haibin Wang, Justin Lee, Suxiang Tong
EPI_ISL_751614	ID Bureau of Laboratories	Genomics and Discovery, Respiratory Viruses Branch, Division of Viral Diseases, Centers for Disease Control and Prevention	Krista Queen, Yan Li, Ying Tao, Jing Zhang, Anna Uehara, Anna Montmayeur, Clinton R. Paden, Peter W. Cook,Rachel Marine, Mili Sheth, Haibin Wang, Justin Lee, Suxiang Tong
EPI_ISL_751616	OH Department of Health Laboratory	Genomics and Discovery, Respiratory Viruses Branch, Division of Viral Diseases, Centers for Disease Control and Prevention	Krista Queen, Yan Li, Ying Tao, Jing Zhang, Anna Uehara, Anna Montmayeur, Clinton R. Paden, Peter W. Cook,Rachel Marine, Mili Sheth, Haibin Wang, Justin Lee, Suxiang Tong
EPI_ISL_751618	ID Bureau of Laboratories	Genomics and Discovery, Respiratory Viruses Branch, Division of Viral Diseases, Centers for Disease Control and Prevention	Krista Queen, Yan Li, Ying Tao, Jing Zhang, Anna Uehara, Anna Montmayeur, Clinton R. Paden, Peter W. Cook,Rachel Marine, Mili Sheth, Haibin Wang, Justin Lee, Suxiang Tong
EPI_ISL_751641	MN PHL Division, Minnesota Department of Health	Genomics and Discovery, Respiratory Viruses Branch, Division of Viral Diseases, Centers for Disease Control and Prevention	Krista Queen, Yan Li, Ying Tao, Jing Zhang, Anna Uehara, Anna Montmayeur, Clinton R. Paden, Peter W. Cook,Rachel Marine, Mili Sheth, Haibin Wang, Justin Lee, Suxiang Tong
EPI_ISL_751656	MO State Public Health Laboratory	Genomics and Discovery, Respiratory Viruses Branch, Division of Viral Diseases, Centers for Disease Control and Prevention	Krista Queen, Yan Li, Ying Tao, Jing Zhang, Anna Uehara, Anna Montmayeur, Clinton R. Paden, Peter W. Cook,Rachel Marine, Mili Sheth, Haibin Wang, Justin Lee, Suxiang Tong
EPI_ISL_751659, EPI_ISL_751662	NJ Public Health and Environmental Laboratories	Genomics and Discovery, Respiratory Viruses Branch, Division of Viral Diseases, Centers for Disease Control and Prevention	Krista Queen, Yan Li, Ying Tao, Jing Zhang, Anna Uehara, Anna Montmayeur, Clinton R. Paden, Peter W. Cook,Rachel Marine, Mili Sheth, Haibin Wang, Justin Lee, Suxiang Tong
EPI_ISL_751694	AK State Public Health Lab, State Health Department	Genomics and Discovery, Respiratory Viruses Branch, Division of Viral Diseases, Centers for Disease Control and Prevention	Krista Queen, Yan Li, Ying Tao, Jing Zhang, Anna Uehara, Anna Montmayeur, Clinton R. Paden, Peter W. Cook,Rachel Marine, Mili Sheth, Haibin Wang, Justin Lee, Suxiang Tong
EPI_ISL_751716	MN PHL Division, Minnesota Department of Health	Genomics and Discovery, Respiratory Viruses Branch, Division of Viral Diseases, Centers for Disease Control and Prevention	Krista Queen, Yan Li, Ying Tao, Jing Zhang, Anna Uehara, Anna Montmayeur, Clinton R. Paden, Peter W. Cook,Rachel Marine, Mili Sheth, Haibin Wang, Justin Lee, Suxiang Tong
EPI_ISL_751720	TX DSHS, Lab Services Section MC 1947	Genomics and Discovery, Respiratory Viruses Branch, Division of Viral Diseases, Centers for Disease Control and Prevention	Krista Queen, Yan Li, Ying Tao, Jing Zhang, Anna Uehara, Anna Montmayeur, Clinton R. Paden, Peter W. Cook,Rachel Marine, Mili Sheth, Haibin Wang, Justin Lee, Suxiang Tong
EPI_ISL_751734	NE Public Health Laboratory	Genomics and Discovery, Respiratory Viruses Branch, Division of Viral Diseases, Centers for Disease Control and Prevention	Krista Queen, Yan Li, Ying Tao, Jing Zhang, Anna Uehara, Anna Montmayeur, Clinton R. Paden, Peter W. Cook,Rachel Marine, Mili Sheth, Haibin Wang, Justin Lee, Suxiang Tong
EPI_ISL_751741	OH Department of Health Laboratory	Genomics and Discovery, Respiratory Viruses Branch, Division of Viral Diseases, Centers for Disease Control and Prevention	Krista Queen, Yan Li, Ying Tao, Jing Zhang, Anna Uehara, Anna Montmayeur, Clinton R. Paden, Peter W. Cook,Rachel Marine, Mili Sheth, Haibin Wang, Justin Lee, Suxiang Tong
EPI_ISL_751748, EPI_ISL_751782	ID Bureau of Laboratories	Genomics and Discovery, Respiratory Viruses Branch, Division of Viral Diseases, Centers for Disease Control and Prevention	Krista Queen, Yan Li, Ying Tao, Jing Zhang, Anna Uehara, Anna Montmayeur, Clinton R. Paden, Peter W. Cook,Rachel Marine, Mili Sheth, Haibin Wang, Justin Lee, Suxiang Tong
EPI_ISL_751784	VA-Division of Consolidated Laboratory Services	Genomics and Discovery, Respiratory Viruses Branch, Division of Viral Diseases, Centers for Disease Control and Prevention	Krista Queen, Yan Li, Ying Tao, Jing Zhang, Anna Uehara, Anna Montmayeur, Clinton R. Paden, Peter W. Cook,Rachel Marine, Mili Sheth, Haibin Wang, Justin Lee, Suxiang Tong
EPI_ISL_751789	ID Bureau of Laboratories	Genomics and Discovery, Respiratory Viruses Branch, Division of Viral Diseases, Centers for Disease Control and Prevention	Krista Queen, Yan Li, Ying Tao, Jing Zhang, Anna Uehara, Anna Montmayeur, Clinton R. Paden, Peter W. Cook,Rachel Marine, Mili Sheth, Haibin Wang, Justin Lee, Suxiang Tong
EPI_ISL_751793	AK State Public Health Lab, State Health Department	Genomics and Discovery, Respiratory Viruses Branch, Division of Viral Diseases, Centers for Disease Control and Prevention	Krista Queen, Yan Li, Ying Tao, Jing Zhang, Anna Uehara, Anna Montmayeur, Clinton R. Paden, Peter W. Cook,Rachel Marine, Mili Sheth, Haibin Wang, Justin Lee, Suxiang Tong
EPI_ISL_753114	State Laboratories Division, Hawaii State Department of Health	State Laboratories Division, Hawaii State Department of Health	Pamela O'Brien, Sabrina Diemert, Drew Kuwazaki, Razvan Sultana, Edward Desmond
EPI_ISL_753345, EPI_ISL_753346, EPI_ISL_753347, EPI_ISL_753348, EPI_ISL_753349, EPI_ISL_753350, EPI_ISL_753351, EPI_ISL_753352, EPI_ISL_753353, EPI_ISL_753354, EPI_ISL_753355, EPI_ISL_753356, EPI_ISL_753357, EPI_ISL_753358, EPI_ISL_753359, EPI_ISL_753360, EPI_ISL_753361, EPI_ISL_753362, EPI_ISL_753363, EPI_ISL_753364, EPI_ISL_753365, EPI_ISL_753366, EPI_ISL_753367, EPI_ISL_753368, EPI_ISL_753369, EPI_ISL_753370, EPI_ISL_753371, EPI_ISL_753372, EPI_ISL_753373, EPI_ISL_753374, EPI_ISL_753375, EPI_ISL_753376, EPI_ISL_753377, EPI_ISL_753378, EPI_ISL_753379, EPI_ISL_753380, EPI_ISL_753381, EPI_ISL_753382, EPI_ISL_753383, EPI_ISL_753384, EPI_ISL_753385, EPI_ISL_753386, EPI_ISL_753387, EPI_ISL_753389, EPI_ISL_753390, EPI_ISL_753391, EPI_ISL_753392, EPI_ISL_753403, EPI_ISL_753404, EPI_ISL_753405, EPI_ISL_753406, EPI_ISL_753407, EPI_ISL_753408			
see above	Clinical virology Laboratory, Children's Hospital Los Angeles	Center for Personalized Medicine, Children's Hospital Los Angeles	Gai et al
EPI_ISL_753714, EPI_ISL_753769, EPI_ISL_753827, EPI_ISL_753831, EPI_ISL_753833	Charité Universitätsmedizin Berlin, Institut für Virologie/Labor Berlin	Charité Universitätsmedizin Berlin, Institut für Virologie	Victor M Corman, Jörn Beheim-Schwarzbach, Barbara Mühlemann, Julia Schneider, Talitha Veith, Terry Jones, Christian Drosten
EPI_ISL_754141, EPI_ISL_754161	Laboratoire de Microbiologie	National Reference Center for Viruses of Respiratory Infections, Institut Pasteur, Paris	Marion Barbet, Sylvie Behillil, Méline Bizard, Angela Brisebarre, Camille Capel, Etienne Simon-Lorière, Vincent Enouf, Maud Vanpeene, Sylvie van der Werf, Marie-Sarah Fangous
EPI_ISL_754546, EPI_ISL_754579, EPI_ISL_754580, EPI_ISL_754581, EPI_ISL_754582, EPI_ISL_754583, EPI_ISL_754584, EPI_ISL_754585, EPI_ISL_754586	Wadsworth Center, New York State Department.of Health	Wadsworth Center, New York State Department.of Health	Kirsten St. George, Daryl M. Lamson, Alexis Russel, Matthew Shudt, Melissa A Leisner, Jonathan Plitnick, Navjot Singh, John Kelly, Sara Griesemer, Erasmus Schneider, Erica Lasek-Nesselquist
EPI_ISL_754771, EPI_ISL_754773, EPI_ISL_754774	Emory Molecular Diagnostics Laboratory, Emory Healthcare	Piantadosi Lab, Emory Department of Pathology	Ahmed Babiker, Anne Piantadosi

EPI_ISL_755117, EPI_ISL_755119, EPI_ISL_755130, EPI_ISL_755134, EPI_ISL_755136, EPI_ISL_755270, EPI_ISL_755271, EPI_ISL_755274, EPI_ISL_755275, EPI_ISL_755280, EPI_ISL_755290, EPI_ISL_755295			
see above	San Diego County Public Health Laboratory	Andersen lab at Scripps Research	SEARCH Alliance San Diego with Tracy Basler, Jovan Shephard, Brett Austin
EPI_ISL_755805, EPI_ISL_755806, EPI_ISL_755807	Toronto Invasive Bacterial Diseases Network	McMaster University	Allison McGeer, Patryk Aftanas, Hooman Derakhshani, Angel Li, Kuganya Nirmalarajah, Emily Panousis, Ahmed Draia, Jalees Nasir, Michael Surette, Samira Mubareka, Andrew G. McArthur
EPI_ISL_756311	The Caribbean Public Health Agency	Carrington Lab, Department of PreClinical Sciences, Faculty of Medical Sciences, The University of the West Indies	Nikita S. D. Sahadeo, Arianne Brown-Jordan, Sarah Hill, Vernie Ramkissoon, Roshan Parasram, Naresh Nandram, Avery Hinds, Jerome Foster, Stanley Giddings, Karla Georges, Marsha Ivey, Rahul Naidu, Risha Singh, SueMin Nathaniel, Rajini Haraksingh, Jaya Jayaraman, Chinna Chinnadurai, Adesh Ramsubhag, Nuno Faria, Oliver Pybus, Christopher Oura, Gabriel Escobar, Christine V. F. Carrington
EPI_ISL_756360	Caribbean Regional Public Health Authority	Carrington Lab, Department of PreClinical Sciences, Faculty of Medical Sciences, The University of the West Indies	Nikita S. D. Sahadeo, Gabriel Escobar, Sarah Hill, Vernie Ramkissoon, Risha Singh, SueMin Nathaniel, Simone Keizer-Beache, Arianne Brown-Jordan, Naresh Nandram, Avery Hinds, Jerome Foster, Stanley Giddings, Karla Georges, Marsha Ivey, Rahul Naidu, , Rajini Haraksingh, Jaya Jayaraman, Chinna Chinnadurai, Adesh Ramsubhag, Nuno Faria, Oliver Pybus, Christopher Oura, Christine V. F. Carrington
EPI_ISL_757187, EPI_ISL_757188, EPI_ISL_757189, EPI_ISL_757190, EPI_ISL_757191, EPI_ISL_757192, EPI_ISL_757193, EPI_ISL_757194, EPI_ISL_757195, EPI_ISL_757196, EPI_ISL_757197, EPI_ISL_757198, EPI_ISL_757199, EPI_ISL_757200, EPI_ISL_757201, EPI_ISL_757202, EPI_ISL_757203, EPI_ISL_757204, EPI_ISL_757205, EPI_ISL_757206, EPI_ISL_757207, EPI_ISL_757208, EPI_ISL_757209, EPI_ISL_757210, EPI_ISL_757211, EPI_ISL_757212, EPI_ISL_757213, EPI_ISL_757214, EPI_ISL_757215, EPI_ISL_757216, EPI_ISL_757217, EPI_ISL_757218, EPI_ISL_757219, EPI_ISL_757220, EPI_ISL_757221, EPI_ISL_757222, EPI_ISL_757223, EPI_ISL_757224, EPI_ISL_757273, EPI_ISL_757274, EPI_ISL_757275, EPI_ISL_757276, EPI_ISL_757277, EPI_ISL_757278, EPI_ISL_757279, EPI_ISL_757280, EPI_ISL_757281, EPI_ISL_757282, EPI_ISL_757283, EPI_ISL_757284			
see above	Lighthouse Lab in Glasgow	Wellcome Sanger Institute for the COVID-19 Genomics UK (COG-UK) Consortium	Harper VanSteenhouse, Yumi Kasai, David Gray, Carol Clugston, Anna Dominiczak and Alex Alderton, Roberto Amato, Sonia Goncalves, Ewan Harrison, David K. Jackson, Ian Johnston, Dominic Kwiatkowski, Cordelia Langford, John Sillitoe on behalf of the Wellcome Sanger Institute COVID-19 Surveillance Team
EPI_ISL_757296, EPI_ISL_757297, EPI_ISL_757298, EPI_ISL_757299, EPI_ISL_757300, EPI_ISL_757301, EPI_ISL_757302, EPI_ISL_757303, EPI_ISL_757304, EPI_ISL_757305, EPI_ISL_757306, EPI_ISL_757307, EPI_ISL_757308, EPI_ISL_757309, EPI_ISL_757310, EPI_ISL_757311, EPI_ISL_757312, EPI_ISL_757313, EPI_ISL_757314, EPI_ISL_757315, EPI_ISL_757316, EPI_ISL_757317, EPI_ISL_757318, EPI_ISL_757319, EPI_ISL_757320, EPI_ISL_757321, EPI_ISL_757322, EPI_ISL_757323, EPI_ISL_757324, EPI_ISL_757325, EPI_ISL_757326, EPI_ISL_757327, EPI_ISL_757328, EPI_ISL_757329, EPI_ISL_757330, EPI_ISL_757331, EPI_ISL_757332, EPI_ISL_757333, EPI_ISL_757334, EPI_ISL_757335, EPI_ISL_757336, EPI_ISL_757337, EPI_ISL_757338, EPI_ISL_757339, EPI_ISL_757340, EPI_ISL_757341			
see above	Department of Virology and Immunology, University of Helsinki and Helsinki University Hospital, Huslab Finland	Department of Virology, Faculty of Medicine, University of Helsinki, Helsinki, Finland	Teemu Smura, Ravi Kant, Phuoc Truong, Hussein Alburkat, Hannimari Kallio-Kokko, Jenni Virtanen, Maija Suvanto, Sari Hannula, Harri Kangas, Pekka Ellonen, Olli Vapalahti
EPI_ISL_759722	Instituto Nacional de Saude (INSA)	Instituto Nacional de Saude (INSA)	Borges et al
EPI_ISL_759973, EPI_ISL_759974	Furst Medical Laboratory	Norwegian Institute of Public Health, Department of Virology	Kathrine Stene-Johansen, Kamilla Heddeland Instefjord, Hilde Elshaug, Marie Paulsen Madsen, Rasmus Riis Kopperud, Hilde Vollan, Karoline Bragstad, Olav Hungnes
EPI_ISL_759975	Haukeland University Hospital, Dept. of Microbiology	Norwegian Institute of Public Health, Department of Virology	Kathrine Stene-Johansen, Kamilla Heddeland Instefjord, Hilde Elshaug, Marie Paulsen Madsen, Rasmus Riis Kopperud, Hilde Vollan, Karoline Bragstad, Olav Hungnes
EPI_ISL_759976	Hospital of Southern Norway - Kristiansand, Department of Medical Microbiology	Norwegian Institute of Public Health, Department of Virology	Kathrine Stene-Johansen, Kamilla Heddeland Instefjord, Hilde Elshaug, Marie Paulsen Madsen, Rasmus Riis Kopperud, Hilde Vollan, Karoline Bragstad, Olav Hungnes
EPI_ISL_759977	Medical Microbiology Unit, Department for Laboratory Medicine, Drammen Hospital, Vestre Viken Health Trust,	Norwegian Institute of Public Health, Department of Virology	Kathrine Stene-Johansen, Kamilla Heddeland Instefjord, Hilde Elshaug, Marie Paulsen Madsen, Rasmus Riis Kopperud, Hilde Vollan, Karoline Bragstad, Olav Hungnes
EPI_ISL_760230, EPI_ISL_760231, EPI_ISL_760232, EPI_ISL_760233, EPI_ISL_760234, EPI_ISL_760235, EPI_ISL_760236, EPI_ISL_760237, EPI_ISL_760238, EPI_ISL_760239, EPI_ISL_760240, EPI_ISL_760241, EPI_ISL_760242, EPI_ISL_760243, EPI_ISL_760244, EPI_ISL_760245			
see above	Division of Emerging Infectious Diseases, Bureau of Infectious Diseases Diagnosis Control, Korea Disease Control and Prevention Agency	Division of Emerging Infectious Diseases, Bureau of Infectious Diseases Diagnosis Control, Korea Disease Control and Prevention Agency	Ae Kyung Park, Il-Hwan Kim, Heui Man Kim, Jeong-Min Kim, Namjoo Lee, Chaeyoung Lee, Sang Hee Woo, Eun-Jin Kim
EPI_ISL_763111, EPI_ISL_763163, EPI_ISL_763172, EPI_ISL_763235, EPI_ISL_763305	Dutch COVID-19 response team	Erasmus Medical Center	Bas Oude Munnink, Reina Sikkema, David Nieuwenhuijse, Irina Chestakova, Anne van der Linden, Marjan Boter, Emmanuelle Munger, Corine GeurtsvanKessel, Annemiek van der Eijk, Richard Molenkamp, Marion Koopmans, on behalf of the Dutch national COVID-19 response team.
EPI_ISL_763331, EPI_ISL_763337, EPI_ISL_763338, EPI_ISL_763339, EPI_ISL_763340, EPI_ISL_763341, EPI_ISL_763342	Florida Bureau of Public Health Laboratories	Florida Bureau of Public Health Laboratories	Sarah Schmedes, Jason Blanton
EPI_ISL_765748, EPI_ISL_765749, EPI_ISL_765844, EPI_ISL_765845, EPI_ISL_765846, EPI_ISL_765847, EPI_ISL_765848, EPI_ISL_765849, EPI_ISL_765850, EPI_ISL_765851, EPI_ISL_765852, EPI_ISL_765853			
see above	Massachusetts General Hospital	Infectious Disease Program, Broad Institute of Harvard and MIT	Lemieux,J.E., Siddle,K.J., Shaw,B., Adams,G., Pierce,V., Turbett,S., Anahtar,M., Branda,J., Slater,D., Harris,J., Lin,A.E., Gladden-Young,A., Lagerborg,K., Rudy,M., DeRuff,K., Carter,A., Normandin,E., Bauer,M., Reilly,S., Tomkins-Tinch,C., Loreth,C., Chaluvadi,S., Neumann,A., Cusick,C., Chapman,S.B., Gnirke,A., Flowers,K., Cerrato,F., Birren,B.W., Gallagher,G., Smole,S., Park,D.J., MacInnis,B.L., Ryan,E., LaRocque,R., Rosenberg,E. and Sabeti,P.C.
EPI_ISL_766071, EPI_ISL_766077, EPI_ISL_766094, EPI_ISL_766098, EPI_ISL_766271, EPI_ISL_766273, EPI_ISL_766274, EPI_ISL_766275	Respiratory Virus Unit, National Infection Service, Public Health England	COVID-19 Genomics UK (COG-UK) Consortium	PHE Covid Sequencing Team
EPI_ISL_766621	Klinisk mikrobiologi	The Public Health Agency of Sweden	Department of Microbiology, The Public Health Agency of Sweden
EPI_ISL_766742	Texas Department of State Health Services	Texas Department of State Health Services	Rashmi Tuladhar, Bonnie Oh, Jenny Zhang, Maliha Rahman, Anita Pokharel, Myong Koag, Chung Wang, Rachel Lee, Grace Kubin, Mayela Pedrueza, James Daniel Bonser
EPI_ISL_767001, EPI_ISL_767004	Delaware Public Health Laboratory	Delaware Public Health Laboratory	Gregory Hovan
EPI_ISL_767239	Lighthouse Lab in Milton Keynes	Wellcome Sanger Institute for the COVID-19 Genomics UK (COG-UK) Consortium	The Lighthouse Lab in Milton Keynes and Alex Alderton, Roberto Amato, Sonia Goncalves, Ewan Harrison, David K. Jackson, Ian Johnston, Dominic Kwiatkowski, Cordelia Langford, John Sillitoe on behalf of the Wellcome Sanger Institute COVID-19 Surveillance Team
EPI_ISL_767351, EPI_ISL_767353, EPI_ISL_767356	Michigan Department of Health and Human Services, Bureau of Laboratories	Michigan Department of Health and Human Services, Bureau of Laboratories	Blankenship HM, Riner D, Soehnlen MK
EPI_ISL_767861, EPI_ISL_767862	Sydney South West Pathology Service (SSWPS) - Royal Prince Alfred Hospital - NSW Health Pathology	NSW Health Pathology - Institute of Clinical Pathology and Medical Research; Westmead Hospital; University of Sydney	CIDM-PH et al.
EPI_ISL_768522	Synphaet Hospital	National Institute of Health, Department of Medical Sciences, Ministry of Public Health, Thailand	Pilailuk Okada; Siripaporn Phuygun; Sittiporn Parnmen; Ratana Tacharoenmuang; Pakorn Piromtong; Natchaya Khiadsang; Thanutsapa Thanadachakul; Warawan Wongboot; sirikanda wimol; Sunthareeya Waicharoen;
EPI_ISL_768727	Jena University Hospital, Institute for Infectious Diseases and Infection Control	Institute of infectious medicine & hospital hygiene, CaSe-Group	Spott, Riccardo; Marquet, Mike; Pletz, Matthias W.; Brandt, Christian
EPI_ISL_769865	Respiratory Virus Unit, National Infection Service, Public Health England	COVID-19 Genomics UK (COG-UK) Consortium	PHE Covid Sequencing Team
EPI_ISL_769986	Area De Salud Catedral Noreste	Inciensa, Instituto Costarricense de Investigación y Enseñanza en Nutrición y Salud	Francisco Duarte, Hebleen Porras, Claudio Soto-Garita, Estela Cordero, Adriana Godínez, Melany Calderón & Mariel López
EPI_ISL_769988	Area De Salud San Francisco-San Antonio (Coopesana)	Inciensa, Instituto Costarricense de Investigación y Enseñanza en Nutrición y Salud	Francisco Duarte, Hebleen Porras, Claudio Soto-Garita, Estela Cordero, Adriana Godínez, Melany Calderón & Mariel López
EPI_ISL_770021	Area De Salud Catedral Noreste	Inciensa, Instituto Costarricense de Investigación y Enseñanza en Nutrición y Salud	Francisco Duarte, Hebleen Porras, Claudio Soto-Garita, Estela Cordero, Adriana Godínez, Melany Calderón & Mariel López

EPI_ISL_770024	Hle - Asociacion Hogar De Ancianos Santiago Crespo Calvo	Inciensa, Instituto Costarricense de Investigación y Enseñanza en Nutrición y Salud	Francisco Duarte, Hebleen Porras, Claudio Soto-Garita, Estela Cordero, Adriana Godínez, Melany Calderón & Mariel López
EPI_ISL_770026	Area De Salud Catedral Noreste	Inciensa, Instituto Costarricense de Investigación y Enseñanza en Nutrición y Salud	Francisco Duarte, Hebleen Porras, Claudio Soto-Garita, Estela Cordero, Adriana Godínez, Melany Calderón & Mariel López
EPI_ISL_770635	WHO National Influenza Centre Russian Federation	WHO National Influenza Centre Russian Federation	Andrey Komissarov, Artem Fadeev, Anna Ivanova, Kseniya Komissarova, Dmitry Bazhenov, Daria Danilenko, Ksenia Safina, Elena Nabieva, Georgii Bazykin, Dmitry Lioznov
EPI_ISL_770780, EPI_ISL_770781, EPI_ISL_770782	Minnesota Department of Health, Public Health Laboratory	Minnesota Department of Health, Public Health Laboratory	Alexandra Lorentz, Jacob Garfin, Matt Plumb, and Xiong Wang
EPI_ISL_770808, EPI_ISL_770809, EPI_ISL_770812	Essentia Health-St. Mary's Medical Center	Minnesota Department of Health, Public Health Laboratory	Alexandra Lorentz, Jacob Garfin, Matt Plumb, and Xiong Wang
EPI_ISL_771152, EPI_ISL_771154, EPI_ISL_771155, EPI_ISL_771158, EPI_ISL_771159, EPI_ISL_771161, EPI_ISL_771163, EPI_ISL_771164, EPI_ISL_771171, EPI_ISL_771172, EPI_ISL_771181, EPI_ISL_771184, EPI_ISL_771189, EPI_ISL_771200, EPI_ISL_771203, EPI_ISL_771206, EPI_ISL_771207, EPI_ISL_771209, EPI_ISL_771210, EPI_ISL_771211, EPI_ISL_771213, EPI_ISL_771216, EPI_ISL_771220, EPI_ISL_771247, EPI_ISL_771248, EPI_ISL_771249, EPI_ISL_771250, EPI_ISL_771251, EPI_ISL_771252, EPI_ISL_771253, EPI_ISL_771254, EPI_ISL_771255, EPI_ISL_771256, EPI_ISL_771257, EPI_ISL_771258, EPI_ISL_771259, EPI_ISL_771260, EPI_ISL_771261, EPI_ISL_771262, EPI_ISL_771263, EPI_ISL_771264, EPI_ISL_771265, EPI_ISL_771266, EPI_ISL_771267, EPI_ISL_771268, EPI_ISL_771269, EPI_ISL_771270, EPI_ISL_771271, EPI_ISL_771272, EPI_ISL_771273, EPI_ISL_771274, EPI_ISL_771275, EPI_ISL_771276, EPI_ISL_771277, EPI_ISL_771278, EPI_ISL_771279, EPI_ISL_771280, EPI_ISL_771281, EPI_ISL_771282, EPI_ISL_771283, EPI_ISL_771284, EPI_ISL_771285, EPI_ISL_771286, EPI_ISL_771287, EPI_ISL_771288, EPI_ISL_771289, EPI_ISL_771290, EPI_ISL_771292, EPI_ISL_771293, EPI_ISL_771294, EPI_ISL_771295, EPI_ISL_771296, EPI_ISL_771297, EPI_ISL_771298, EPI_ISL_771299, EPI_ISL_771302, EPI_ISL_771307, EPI_ISL_771331, EPI_ISL_771332, EPI_ISL_771350			
see above	Colorado Department of Public Health and Environment	Colorado Department of Puplic Health and Environment	Laura Bankers, Molly C. Hetherington-Rauth, Diana Ir, Shannon Ely, Shannon R. Matzinger, Sarah Elizabeth Totten, Emily A. Travanty
EPI_ISL_775271, EPI_ISL_775294	Furst Medical Laboratory	Norwegian Institute of Public Health, Department of Virology	Kathrine Stene-Johansen, Kamilla Heddeland Instefjord, Hilde Elshaug, Atiya R Ali,Marie Paulsen Madsen, Rasmus Riis Kopperud, Hilde Vollan, Karoline Bragstad, Olav Hungnes
EPI_ISL_775297	Nordland Hospital - Bodo, Laboratory Department, Molecular Biology Unit	Norwegian Institute of Public Health, Department of Virology	Kathrine Stene-Johansen, Kamilla Heddeland Instefjord, Hilde Elshaug, Atiya R Ali,Marie Paulsen Madsen, Rasmus Riis Kopperud, Hilde Vollan, Karoline Bragstad, Olav Hungnes
EPI_ISL_775335, EPI_ISL_775336	Haukeland University Hospital, Dept. of Microbiology	Norwegian Institute of Public Health, Department of Virology	Kathrine Stene-Johansen, Kamilla Heddeland Instefjord, Hilde Elshaug, Atiya R Ali,Marie Paulsen Madsen, Rasmus Riis Kopperud, Hilde Vollan, Karoline Bragstad, Olav Hungnes
EPI_ISL_775349, EPI_ISL_775350, EPI_ISL_775351, EPI_ISL_775352	Unilabs Laboratory Medicine	Norwegian Institute of Public Health, Department of Virology	Kathrine Stene-Johansen, Kamilla Heddeland Instefjord, Hilde Elshaug, Atiya R Ali,Marie Paulsen Madsen, Rasmus Riis Kopperud, Hilde Vollan, Karoline Bragstad, Olav Hungnes
EPI_ISL_775354	Dept. of Medical Microbiology, Stavanger University Hospital, Helse Stavanger HF	Norwegian Institute of Public Health, Department of Virology	Kathrine Stene-Johansen, Kamilla Heddeland Instefjord, Hilde Elshaug, Atiya R Ali,Marie Paulsen Madsen, Rasmus Riis Kopperud, Hilde Vollan, Karoline Bragstad, Olav Hungnes
EPI_ISL_775363, EPI_ISL_775364, EPI_ISL_775365, EPI_ISL_775366, EPI_ISL_775367	Foerde Hospital, Department of Microbiology	Norwegian Institute of Public Health, Department of Virology	Kathrine Stene-Johansen, Kamilla Heddeland Instefjord, Hilde Elshaug, Atiya R Ali,Marie Paulsen Madsen, Rasmus Riis Kopperud, Hilde Vollan, Karoline Bragstad, Olav Hungnes
EPI_ISL_775388	Akershus University Hospital, Department for Microbiology and Infectious Disease Control	Norwegian Institute of Public Health, Department of Virology	Kathrine Stene-Johansen, Kamilla Heddeland Instefjord, Hilde Elshaug, Atiya R Ali,Marie Paulsen Madsen, Rasmus Riis Kopperud, Hilde Vollan, Karoline Bragstad, Olav Hungnes
EPI_ISL_775457	Oslo University Hospital, Department of Medical Microbiology	Norwegian Institute of Public Health, Department of Virology	Kathrine Stene-Johansen, Kamilla Heddeland Instefjord, Hilde Elshaug, Atiya R Ali,Marie Paulsen Madsen, Rasmus Riis Kopperud, Hilde Vollan, Karoline Bragstad, Olav Hungnes
EPI_ISL_775484, EPI_ISL_775489, EPI_ISL_775490	Furst Medical Laboratory	Norwegian Institute of Public Health, Department of Virology	Kathrine Stene-Johansen, Kamilla Heddeland Instefjord, Hilde Elshaug, Atiya R Ali,Marie Paulsen Madsen, Rasmus Riis Kopperud, Hilde Vollan, Karoline Bragstad, Olav Hungnes
EPI_ISL_775498, EPI_ISL_775499	Vestfold Hospital, Toensberg Department of Microbiology	Norwegian Institute of Public Health, Department of Virology	Kathrine Stene-Johansen, Kamilla Heddeland Instefjord, Hilde Elshaug, Atiya R Ali,Marie Paulsen Madsen, Rasmus Riis Kopperud, Hilde Vollan, Karoline Bragstad, Olav Hungnes
EPI_ISL_775514	Norwegian Institute of Public Health, Department of Virology	Norwegian Institute of Public Health, Department of Virology	Kathrine Stene-Johansen, Kamilla Heddeland Instefjord, Hilde Elshaug, Atiya R Ali,Marie Paulsen Madsen, Rasmus Riis Kopperud, Hilde Vollan, Karoline Bragstad, Olav Hungnes
EPI_ISL_778834	AIID	Irish Coronavirus Sequencing Consortium-Teagasc Grange	Matthew McCabe, Aljandro Abner Garcia Leon, Fiona Crispie, Calum Walsh, Michael Carr, John Kenny, Paul Cotter, Patrick Mallon, Gabriel Gonzalez
EPI_ISL_779185, EPI_ISL_779186, EPI_ISL_779188, EPI_ISL_779192, EPI_ISL_779196	Laboratorio Estatal de Salud Pública de Nuevo León	Laboratorio de Infectología Molecular, Departamento de Bioquímica y Medicina Molecular,Facultad de Medicina - Universidad Autónoma de Nuevo León	Kame A. Galán-Huerta, María F. Herrera-Saldivar, Natalia Martínez-Acuña, Sonia A. Lozano-Sepúlveda, Daniel Arellanos-Soto, Ana M. Rivas-Estilla, Samuel Buentello-Wong, Else del Carmen García-García, Gloria A. Jasso-de-la-Peña, Roberto Montes-de-Oca, Consuelo Treviño-Garza, Manuel E. de-la-O-Cavazos
EPI_ISL_779781, EPI_ISL_779782, EPI_ISL_779821, EPI_ISL_779822, EPI_ISL_779823, EPI_ISL_779824, EPI_ISL_779825, EPI_ISL_779826, EPI_ISL_779827, EPI_ISL_779828, EPI_ISL_779829, EPI_ISL_779830, EPI_ISL_779831, EPI_ISL_779832, EPI_ISL_779833, EPI_ISL_779834, EPI_ISL_779835			
see above	Laboratoire de virologie, CHU de Grenoble	CNR Virus des Infections Respiratoires - France SUD	Antonin Bal, Gregory Destras, Gwendolyne Burfin, Hadrien Règue, Quentin Semanas, Martine Valette, Bruno Lina, Sylvie Larrat, Laurence Josset
EPI_ISL_779955, EPI_ISL_779959, EPI_ISL_779963, EPI_ISL_779969, EPI_ISL_779971, EPI_ISL_779972, EPI_ISL_779973, EPI_ISL_779975, EPI_ISL_779976, EPI_ISL_780012, EPI_ISL_780013, EPI_ISL_780014, EPI_ISL_780015, EPI_ISL_780016, EPI_ISL_780017, EPI_ISL_780019, EPI_ISL_780020, EPI_ISL_780029, EPI_ISL_780032, EPI_ISL_780034, EPI_ISL_780039, EPI_ISL_780042, EPI_ISL_780045, EPI_ISL_780050, EPI_ISL_780055, EPI_ISL_780056, EPI_ISL_780060, EPI_ISL_780063, EPI_ISL_780065, EPI_ISL_780067, EPI_ISL_780075, EPI_ISL_780076, EPI_ISL_780078, EPI_ISL_780079, EPI_ISL_780080, EPI_ISL_780081, EPI_ISL_780082, EPI_ISL_780083, EPI_ISL_780084, EPI_ISL_780085, EPI_ISL_780086, EPI_ISL_780087, EPI_ISL_780088, EPI_ISL_780089			
see above	Hospital General Universitario Gregorio Marañón	SeqCOVID-SPAIN consortium/IBV(CSIC)	Darío García de Viedma, Laura Pérez-Lago, Marta Herranz, Jon Sicilia, Julia Suárez, Pilar Catalán, Patricia Muñoz and SeqCOVID-SPAIN consortium
EPI_ISL_780397, EPI_ISL_780398, EPI_ISL_780399, EPI_ISL_780400, EPI_ISL_780401	Bermuda Government Molecular Diagnostics Laboratory (MDL)	Respiratory Virus Unit, National Infection Service, Public Health England	PHE Covid Sequencing Team, Dr Carika Weldon (Bermuda), Dr Ayoola Oyinloye (Bermuda)
EPI_ISL_783069, EPI_ISL_783070, EPI_ISL_783071, EPI_ISL_783072, EPI_ISL_783073, EPI_ISL_783074, EPI_ISL_783075, EPI_ISL_783076, EPI_ISL_783077, EPI_ISL_783078, EPI_ISL_783079, EPI_ISL_783080, EPI_ISL_783081, EPI_ISL_783082, EPI_ISL_783083, EPI_ISL_783084, EPI_ISL_783085, EPI_ISL_783086, EPI_ISL_783087, EPI_ISL_783088, EPI_ISL_783089, EPI_ISL_783090, EPI_ISL_783091, EPI_ISL_783092, EPI_ISL_783093, EPI_ISL_783094, EPI_ISL_783095, EPI_ISL_783096, EPI_ISL_783097, EPI_ISL_783098, EPI_ISL_783099, EPI_ISL_783100, EPI_ISL_783101, EPI_ISL_783102, EPI_ISL_783103, EPI_ISL_783104, EPI_ISL_783105, EPI_ISL_783106, EPI_ISL_783107, EPI_ISL_783108, EPI_ISL_783109, EPI_ISL_783110, EPI_ISL_783111, EPI_ISL_783112, EPI_ISL_783113, EPI_ISL_783114, EPI_ISL_783115, EPI_ISL_783116, EPI_ISL_783117, EPI_ISL_783118, EPI_ISL_783119, EPI_ISL_783120, EPI_ISL_783121, EPI_ISL_783122, EPI_ISL_783123, EPI_ISL_783124, EPI_ISL_783125, EPI_ISL_783126, EPI_ISL_783127, EPI_ISL_783128, EPI_ISL_783129, EPI_ISL_783130, EPI_ISL_783131, EPI_ISL_783132, EPI_ISL_783133, EPI_ISL_783134, EPI_ISL_783135, EPI_ISL_783136, EPI_ISL_783137, EPI_ISL_783138, EPI_ISL_783139, EPI_ISL_783140, EPI_ISL_783141, EPI_ISL_783142, EPI_ISL_783143, EPI_ISL_783144, EPI_ISL_783145, EPI_ISL_783146, EPI_ISL_783147, EPI_ISL_783148, EPI_ISL_783149, EPI_ISL_783150, EPI_ISL_783151, EPI_ISL_783152, EPI_ISL_783153, EPI_ISL_783154, EPI_ISL_783155, EPI_ISL_783156, EPI_ISL_783157, EPI_ISL_783158, EPI_ISL_783159, EPI_ISL_783160, EPI_ISL_783161, EPI_ISL_783162, EPI_ISL_783163, EPI_ISL_783164, EPI_ISL_783165, EPI_ISL_783166, EPI_ISL_783167, EPI_ISL_783168, EPI_ISL_783169, EPI_ISL_783170, EPI_ISL_783171, EPI_ISL_783172, EPI_ISL_783173, EPI_ISL_783174, EPI_ISL_783175, EPI_ISL_783176, EPI_ISL_783177, EPI_ISL_783178, EPI_ISL_783179, EPI_ISL_783180, EPI_ISL_783181, EPI_ISL_783182, EPI_ISL_783183, EPI_ISL_783184, EPI_ISL_783185, EPI_ISL_783186, EPI_ISL_783187, EPI_ISL_783188, EPI_ISL_783189, EPI_ISL_783190, EPI_ISL_783191, EPI_ISL_783192, EPI_ISL_783193, EPI_ISL_783194, EPI_ISL_783195, EPI_ISL_783196, EPI_ISL_783197, EPI_ISL_783198, EPI_ISL_783199, EPI_ISL_783200, EPI_ISL_783201, EPI_ISL_783202, EPI_ISL_783203, EPI_ISL_783204, EPI_ISL_783205, EPI_ISL_783206, EPI_ISL_783207, EPI_ISL_783208, EPI_ISL_783209, EPI_ISL_783210, EPI_ISL_783211, EPI_ISL_783212, EPI_ISL_783213, EPI_ISL_783214, EPI_ISL_783215, EPI_ISL_783216, EPI_ISL_783217, EPI_ISL_783218, EPI_ISL_783219, EPI_ISL_783220, EPI_ISL_783221, EPI_ISL_783222, EPI_ISL_783223, EPI_ISL_783224, EPI_ISL_783225, EPI_ISL_783226, EPI_ISL_783227, EPI_ISL_783228, EPI_ISL_783229, EPI_ISL_783230, EPI_ISL_783231, EPI_ISL_783232, EPI_ISL_783233, EPI_ISL_783234, EPI_ISL_783235, EPI_ISL_783236, EPI_ISL_783237, EPI_ISL_783238, EPI_ISL_783239, EPI_ISL_783240, EPI_ISL_783241, EPI_ISL_783242, EPI_ISL_783243, EPI_ISL_783244, EPI_ISL_783245, EPI_ISL_783246, EPI_ISL_783247, EPI_ISL_783248, EPI_ISL_783249, EPI_ISL_783250, EPI_ISL_783251, EPI_ISL_783252, EPI_ISL_783253, EPI_ISL_783254, EPI_ISL_783255, EPI_ISL_783256, EPI_ISL_783257, EPI_ISL_783258, EPI_ISL_783259, EPI_ISL_783260, EPI_ISL_783261, EPI_ISL_783262, EPI_ISL_783263, EPI_ISL_783264, EPI_ISL_783265, EPI_ISL_783266, EPI_ISL_783267, EPI_ISL_783268, EPI_ISL_783269, EPI_ISL_783270, EPI_ISL_783271, EPI_ISL_783272, EPI_ISL_783273, EPI_ISL_783274, EPI_ISL_783275, EPI_ISL_783276, EPI_ISL_783277, EPI_ISL_783278, EPI_ISL_783279, EPI_ISL_783280, EPI_ISL_783281, EPI_ISL_783282, EPI_ISL_783283, EPI_ISL_783284, EPI_ISL_783285, EPI_ISL_783286, EPI_ISL_783287, EPI_ISL_783288, EPI_ISL_783289, EPI_ISL_783290, EPI_ISL_783291, EPI_ISL_783292, EPI_ISL_783293, EPI_ISL_783294, EPI_ISL_783295, EPI_ISL_783296, EPI_ISL_783297, EPI_ISL_783298, EPI_ISL_783299, EPI_ISL_783300, EPI_ISL_783301, EPI_ISL_783302, EPI_ISL_783303, EPI_ISL_783304, EPI_ISL_783305, EPI_ISL_783306, EPI_ISL_783307, EPI_ISL_783308, EPI_ISL_783309, EPI_ISL_783310, EPI_ISL_783311, EPI_ISL_783312, EPI_ISL_783313, EPI_ISL_783314, EPI_ISL_783315, EPI_ISL_783316, EPI_ISL_783317, EPI_ISL_783318, EPI_ISL_783319, EPI_ISL_783320, EPI_ISL_783321, EPI_ISL_783322, EPI_ISL_783323, EPI_ISL_783324, EPI_ISL_783325, EPI_ISL_783326, EPI_ISL_783327, EPI_ISL_783328, EPI_ISL_783329, EPI_ISL_783330, EPI_ISL_783331, EPI_ISL_783332, EPI_ISL_783333, EPI_ISL_783334, EPI_ISL_783335, EPI_ISL_783336, EPI_ISL_783337, EPI_ISL_783338, EPI_ISL_783339, EPI_ISL_783340, EPI_ISL_783341, EPI_ISL_783342, EPI_ISL_783343, EPI_ISL_783344, EPI_ISL_783345, EPI_ISL_783346, EPI_ISL_783347, EPI_ISL_783348, EPI_ISL_783349, EPI_ISL_783350, EPI_ISL_783351, EPI_ISL_783352, EPI_ISL_783353, EPI_ISL_783354, EPI_ISL_783355, EPI_ISL_783356, EPI_ISL_783357, EPI_ISL_783358, EPI_ISL_783359, EPI_ISL_783360, EPI_ISL_783361, EPI_ISL_783362, EPI_ISL_783363, EPI_ISL_783364, EPI_ISL_783365, EPI_ISL_783366			
see above	Lighthouse Lab in Milton Keynes	Wellcome Sanger Institute for the COVID-19 Genomics UK (COG-UK) Consortium	The Lighthouse Lab in Milton Keynes and Alex Alderton, Roberto Amato, Sonia Goncalves, Ewan Harrison, David K. Jackson, Ian Johnston, Dominic Kwiatkowski, Cordelia Langford, John Sillitoe on behalf of the Wellcome Sanger Institute COVID-19 Surveillance Team
EPI_ISL_783537, EPI_ISL_783540, EPI_ISL_783545, EPI_ISL_783547, EPI_ISL_783549, EPI_ISL_783551,	Houston Methodist Hospital	Houston Methodist Hospital	S. Wesley Long, Randall J. Olsen, Paul A. Christensen, David W. Bernard, James J. Davis, Maulik Shukla, Marcus Nguyen, Matthew Ojeda Saavedra, Prasanti Yerramilli, Layne Pruitt, Sishir Subedi, Heather Hendrickson, and James M. Musser

EPI_ISL_783553, EPI_ISL_783569			
EPI_ISL_783850	Bermuda Government Molecular Diagnostics Laboratory (MDL)	Respiratory Virus Unit, National Infection Service, Public Health England	PHE Covid Sequencing Team, Dr Carika Weldon (Bermuda), Dr Ayoola Oyinloye (Bermuda)
EPI_ISL_783869, EPI_ISL_783870, EPI_ISL_783871, EPI_ISL_783872, EPI_ISL_783873, EPI_ISL_783874, EPI_ISL_783879, EPI_ISL_783880, EPI_ISL_783881, EPI_ISL_783882, EPI_ISL_783883, EPI_ISL_783889, EPI_ISL_783890, EPI_ISL_783891, EPI_ISL_783892, EPI_ISL_783893, EPI_ISL_783898, EPI_ISL_783899, EPI_ISL_783900, EPI_ISL_783901, EPI_ISL_783902, EPI_ISL_783903, EPI_ISL_783908, EPI_ISL_783909, EPI_ISL_783910, EPI_ISL_783911, EPI_ISL_783912, EPI_ISL_783913, EPI_ISL_783914, EPI_ISL_783919, EPI_ISL_783920, EPI_ISL_783921, EPI_ISL_783922, EPI_ISL_783923, EPI_ISL_783924, EPI_ISL_783928, EPI_ISL_783929, EPI_ISL_783930, EPI_ISL_783931, EPI_ISL_783932, EPI_ISL_783933, EPI_ISL_783940, EPI_ISL_783941, EPI_ISL_783942, EPI_ISL_783943, EPI_ISL_783944, EPI_ISL_783945, EPI_ISL_783946, EPI_ISL_783950, EPI_ISL_783953, EPI_ISL_783957, EPI_ISL_783963, EPI_ISL_783964, EPI_ISL_783967, EPI_ISL_783971, EPI_ISL_783977, EPI_ISL_783979, EPI_ISL_783981, EPI_ISL_783982, EPI_ISL_783983, EPI_ISL_783985, EPI_ISL_783991, EPI_ISL_783992, EPI_ISL_784001, EPI_ISL_784002, EPI_ISL_784004, EPI_ISL_784008, EPI_ISL_784013, EPI_ISL_784019, EPI_ISL_784022, EPI_ISL_784024, EPI_ISL_784025, EPI_ISL_784026, EPI_ISL_784027, EPI_ISL_784035, EPI_ISL_784040, EPI_ISL_784045, EPI_ISL_784052, EPI_ISL_784059, EPI_ISL_784060, EPI_ISL_784066, EPI_ISL_784069, EPI_ISL_784073, EPI_ISL_784076, EPI_ISL_784077, EPI_ISL_784078, EPI_ISL_784081, EPI_ISL_784085, EPI_ISL_784087, EPI_ISL_784092, EPI_ISL_784099, EPI_ISL_784106, EPI_ISL_784112, EPI_ISL_784113, EPI_ISL_784123, EPI_ISL_784134, EPI_ISL_784146, EPI_ISL_784150, EPI_ISL_784154, EPI_ISL_784162, EPI_ISL_784170, EPI_ISL_784171, EPI_ISL_784399, EPI_ISL_784400, EPI_ISL_784401, EPI_ISL_784402, EPI_ISL_784403, EPI_ISL_784410, EPI_ISL_784411, EPI_ISL_784412, EPI_ISL_784413, EPI_ISL_784414, EPI_ISL_784415, EPI_ISL_784423, EPI_ISL_784424, EPI_ISL_784425, EPI_ISL_784426, EPI_ISL_784431, EPI_ISL_784435, EPI_ISL_784436, EPI_ISL_784437, EPI_ISL_784438, EPI_ISL_784446, EPI_ISL_784447, EPI_ISL_784448, EPI_ISL_784449, EPI_ISL_784450, EPI_ISL_784454, EPI_ISL_784456, EPI_ISL_784458, EPI_ISL_784459, EPI_ISL_784460, EPI_ISL_784461, EPI_ISL_784462, EPI_ISL_784469, EPI_ISL_784470, EPI_ISL_784471, EPI_ISL_784472, EPI_ISL_784473, EPI_ISL_784474, EPI_ISL_784481, EPI_ISL_784482, EPI_ISL_784492, EPI_ISL_784509, EPI_ISL_784510, EPI_ISL_784514, EPI_ISL_784524, EPI_ISL_784568, EPI_ISL_784570, EPI_ISL_784572, EPI_ISL_784575, EPI_ISL_784577, EPI_ISL_784578, EPI_ISL_784580, EPI_ISL_784582, EPI_ISL_784583, EPI_ISL_784584, EPI_ISL_784586, EPI_ISL_784587, EPI_ISL_784588, EPI_ISL_784589, EPI_ISL_784590, EPI_ISL_784593, EPI_ISL_784594, EPI_ISL_784595, EPI_ISL_784596, EPI_ISL_784597, EPI_ISL_784600, EPI_ISL_784601, EPI_ISL_784602, EPI_ISL_784603, EPI_ISL_784605, EPI_ISL_784606, EPI_ISL_784607, EPI_ISL_784608, EPI_ISL_784609, EPI_ISL_784610, EPI_ISL_784611, EPI_ISL_784612, EPI_ISL_784613, EPI_ISL_784614, EPI_ISL_784615, EPI_ISL_784618, EPI_ISL_784619, EPI_ISL_784620, EPI_ISL_784621, EPI_ISL_784622, EPI_ISL_784623, EPI_ISL_784624, EPI_ISL_784625, EPI_ISL_784626, EPI_ISL_784627, EPI_ISL_784628, EPI_ISL_784629, EPI_ISL_784630, EPI_ISL_784631, EPI_ISL_784632, EPI_ISL_784633, EPI_ISL_784634, EPI_ISL_784635, EPI_ISL_784636, EPI_ISL_784637, EPI_ISL_784638, EPI_ISL_784641, EPI_ISL_784642, EPI_ISL_784643, EPI_ISL_784644, EPI_ISL_784645, EPI_ISL_784646, EPI_ISL_784647, EPI_ISL_784648, EPI_ISL_784649, EPI_ISL_784650, EPI_ISL_784653, EPI_ISL_784654, EPI_ISL_784655, EPI_ISL_784656, EPI_ISL_784657, EPI_ISL_784658, EPI_ISL_784659, EPI_ISL_784660, EPI_ISL_784661, EPI_ISL_784665, EPI_ISL_784666, EPI_ISL_784667, EPI_ISL_784668, EPI_ISL_784669, EPI_ISL_784670, EPI_ISL_784671, EPI_ISL_784672, EPI_ISL_784673, EPI_ISL_784674, EPI_ISL_784675, EPI_ISL_784676, EPI_ISL_784677, EPI_ISL_784678, EPI_ISL_784679, EPI_ISL_784680, EPI_ISL_784681, EPI_ISL_784682, EPI_ISL_784683, EPI_ISL_784684, EPI_ISL_784685, EPI_ISL_784686, EPI_ISL_784687, EPI_ISL_784688, EPI_ISL_784689, EPI_ISL_784690, EPI_ISL_784691, EPI_ISL_784692, EPI_ISL_784693, EPI_ISL_784694, EPI_ISL_784695, EPI_ISL_784696, EPI_ISL_784697, EPI_ISL_784698, EPI_ISL_784699, EPI_ISL_784700, EPI_ISL_784701, EPI_ISL_784702, EPI_ISL_784703, EPI_ISL_784704, EPI_ISL_784705, EPI_ISL_784706, EPI_ISL_784708, EPI_ISL_784709, EPI_ISL_784710, EPI_ISL_784711, EPI_ISL_784712, EPI_ISL_784713, EPI_ISL_784714, EPI_ISL_784715, EPI_ISL_784716, EPI_ISL_784717, EPI_ISL_784718, EPI_ISL_784719, EPI_ISL_784720, EPI_ISL_784721, EPI_ISL_784722, EPI_ISL_784723, EPI_ISL_784724, EPI_ISL_784725, EPI_ISL_784726, EPI_ISL_784727, EPI_ISL_784728, EPI_ISL_784729, EPI_ISL_784730, EPI_ISL_784731, EPI_ISL_784732, EPI_ISL_784733, EPI_ISL_784734, EPI_ISL_784735, EPI_ISL_784736, EPI_ISL_784737, EPI_ISL_784738, EPI_ISL_784739, EPI_ISL_784740, EPI_ISL_784741, EPI_ISL_784742, EPI_ISL_784743, EPI_ISL_784744, EPI_ISL_784745, EPI_ISL_784746, EPI_ISL_784747, EPI_ISL_784748, EPI_ISL_784749, EPI_ISL_784750, EPI_ISL_784751, EPI_ISL_784752, EPI_ISL_784753, EPI_ISL_784754, EPI_ISL_784755, EPI_ISL_784756, EPI_ISL_784757, EPI_ISL_784758, EPI_ISL_784759, EPI_ISL_784760, EPI_ISL_784761, EPI_ISL_784762, EPI_ISL_784763, EPI_ISL_784764, EPI_ISL_784765, EPI_ISL_784766, EPI_ISL_784767, EPI_ISL_784768, EPI_ISL_784769, EPI_ISL_784770, EPI_ISL_784771, EPI_ISL_784772, EPI_ISL_784774, EPI_ISL_784776, EPI_ISL_784778, EPI_ISL_784780, EPI_ISL_784783, EPI_ISL_784784, EPI_ISL_784785, EPI_ISL_784787, EPI_ISL_784788, EPI_ISL_784789, EPI_ISL_784791, EPI_ISL_784792, EPI_ISL_784793, EPI_ISL_784795, EPI_ISL_784796, EPI_ISL_784797, EPI_ISL_784798, EPI_ISL_784799, EPI_ISL_784800, EPI_ISL_784801, EPI_ISL_784803, EPI_ISL_784804, EPI_ISL_784805, EPI_ISL_784806, EPI_ISL_784807, EPI_ISL_784808, EPI_ISL_784809, EPI_ISL_784810, EPI_ISL_784811, EPI_ISL_784812, EPI_ISL_784816, EPI_ISL_784819, EPI_ISL_784820, EPI_ISL_784821, EPI_ISL_784822, EPI_ISL_784823, EPI_ISL_784825, EPI_ISL_784827, EPI_ISL_784829, EPI_ISL_784831, EPI_ISL_784833, EPI_ISL_784834, EPI_ISL_784835, EPI_ISL_784836, EPI_ISL_784837, EPI_ISL_784838, EPI_ISL_784840, EPI_ISL_784843, EPI_ISL_784844, EPI_ISL_784845, EPI_ISL_784846, EPI_ISL_784847, EPI_ISL_784848, EPI_ISL_784850, EPI_ISL_784851, EPI_ISL_784853, EPI_ISL_784855, EPI_ISL_784856, EPI_ISL_784858, EPI_ISL_784859, EPI_ISL_784863, EPI_ISL_784864, EPI_ISL_784867, EPI_ISL_784868, EPI_ISL_784869, EPI_ISL_784870, EPI_ISL_784872, EPI_ISL_784873, EPI_ISL_784874, EPI_ISL_784875, EPI_ISL_784878, EPI_ISL_784879, EPI_ISL_784881, EPI_ISL_784883, EPI_ISL_784885, EPI_ISL_784887, EPI_ISL_784892, EPI_ISL_784895, EPI_ISL_784896, EPI_ISL_784897, EPI_ISL_784899, EPI_ISL_784901, EPI_ISL_784902, EPI_ISL_784903, EPI_ISL_784904, EPI_ISL_784905, EPI_ISL_784908, EPI_ISL_784911, EPI_ISL_784912, EPI_ISL_784913, EPI_ISL_784915, EPI_ISL_784916, EPI_ISL_784919, EPI_ISL_784921, EPI_ISL_784923, EPI_ISL_784925, EPI_ISL_784926, EPI_ISL_784927, EPI_ISL_784928, EPI_ISL_784933, EPI_ISL_784936, EPI_ISL_784937, EPI_ISL_784938, EPI_ISL_784940, EPI_ISL_784941, EPI_ISL_784942, EPI_ISL_784943, EPI_ISL_784944, EPI_ISL_784946, EPI_ISL_784947, EPI_ISL_784948, EPI_ISL_784949, EPI_ISL_784950, EPI_ISL_784953, EPI_ISL_784956, EPI_ISL_784958, EPI_ISL_784960, EPI_ISL_784962, EPI_ISL_784964, EPI_ISL_784967, EPI_ISL_784975, EPI_ISL_784977, EPI_ISL_784987, EPI_ISL_784992, EPI_ISL_784998, EPI_ISL_785000, EPI_ISL_785007, EPI_ISL_785012, EPI_ISL_785013, EPI_ISL_785016, EPI_ISL_785023, EPI_ISL_785025, EPI_ISL_785026, EPI_ISL_785027, EPI_ISL_785028, EPI_ISL_785034, EPI_ISL_785038, EPI_ISL_785048, EPI_ISL_785049, EPI_ISL_785050, EPI_ISL_785051, EPI_ISL_785052, EPI_ISL_785074, EPI_ISL_785106, EPI_ISL_785122, EPI_ISL_786091, EPI_ISL_786092, EPI_ISL_786093, EPI_ISL_786094, EPI_ISL_786095, EPI_ISL_786096, EPI_ISL_786097, EPI_ISL_786098, EPI_ISL_786099, EPI_ISL_786100, EPI_ISL_786101, EPI_ISL_786102, EPI_ISL_786103, EPI_ISL_786104, EPI_ISL_786105, EPI_ISL_786106, EPI_ISL_786107, EPI_ISL_786108, EPI_ISL_786109, EPI_ISL_786110, EPI_ISL_786111, EPI_ISL_786112, EPI_ISL_786113, EPI_ISL_786114, EPI_ISL_786115, EPI_ISL_786116, EPI_ISL_786117, EPI_ISL_786118, EPI_ISL_786119, EPI_ISL_786120, EPI_ISL_786121, EPI_ISL_786122, EPI_ISL_786123, EPI_ISL_786124, EPI_ISL_786125, EPI_ISL_786126, EPI_ISL_786127, EPI_ISL_786128, EPI_ISL_786129, EPI_ISL_786130, EPI_ISL_786131, EPI_ISL_786132, EPI_ISL_786133, EPI_ISL_786134, EPI_ISL_786135, EPI_ISL_786136, EPI_ISL_786137, EPI_ISL_786138, EPI_ISL_786139, EPI_ISL_786140, EPI_ISL_786141, EPI_ISL_786142, EPI_ISL_786143, EPI_ISL_786144, EPI_ISL_786145, EPI_ISL_786146, EPI_ISL_786147, EPI_ISL_786148, EPI_ISL_786149, EPI_ISL_786150, EPI_ISL_786151, EPI_ISL_786152, EPI_ISL_786153, EPI_ISL_786154, EPI_ISL_786155, EPI_ISL_786156, EPI_ISL_786157, EPI_ISL_786158, EPI_ISL_786159, EPI_ISL_786160, EPI_ISL_786161, EPI_ISL_786162, EPI_ISL_786163, EPI_ISL_786164, EPI_ISL_786165, EPI_ISL_786166, EPI_ISL_786167, EPI_ISL_786168, EPI_ISL_786169, EPI_ISL_786170, EPI_ISL_786171, EPI_ISL_786172, EPI_ISL_786173, EPI_ISL_786174, EPI_ISL_786175, EPI_ISL_786176, EPI_ISL_786177, EPI_ISL_786178, EPI_ISL_786179, EPI_ISL_786180, EPI_ISL_786181, EPI_ISL_786182, EPI_ISL_786183, EPI_ISL_786184, EPI_ISL_786185, EPI_ISL_786186, EPI_ISL_786187, EPI_ISL_786188, EPI_ISL_786189, EPI_ISL_786190, EPI_ISL_786191			
see above	Houston Methodist Hospital	Houston Methodist Hospital	S. Wesley Long, Randall J. Olsen, Paul A. Christensen, David W. Bernard, James J. Davis, Maulik Shukla, Marcus Nguyen, Matthew Ojeda Saavedra, Prasanti Yerramilli, Layne Pruitt, Sishir Subedi, Heather Hendrickson, and James M. Musser
EPI_ISL_790631, EPI_ISL_790674	Dutch COVID-19 response team	National Institute for Public Health and the Environment (RIVM)	Adam Meijer, Harry Vennema, Jeroen Cremer, Sharon van den Brink, Bas van der Veer, AnneMarie van den Brandt, Florian Zwagemaker, Dennis Schmitz, Chantal Reusken, on behalf of the national COVID-19 response team
EPI_ISL_791298	National Virus Reference Laboratory	Irish Coronavirus Sequencing Consortium - Teagasc Moorepark	Alejandro Abner Garcia Leon, Paul Cotter, Fiona Crispie, John Kenny, Paddy Mallon, Calum Walsh
EPI_ISL_791366, EPI_ISL_791369, EPI_ISL_791380, EPI_ISL_791407, EPI_ISL_791409, EPI_ISL_791410	Johns Hopkins Hospital Department of Pathology	Johns Hopkins Hospital Department of Pathology	C. Paul Morris, Chun Huai Luo, Heba H. Mostafa
EPI_ISL_792558	Grubaugh Lab - Yale School of Public Health	Grubaugh Lab - Yale School of Public Health	Joseph Fauver, Tara Alpert, Anderson Brito, Nathan Grubaugh
EPI_ISL_792649	LACEN-PR	Laboratory of Respiratory Viruses and Measles, Oswaldo Cruz Institute, FIOCRUZ	Paola Resende, Luciana Appolinario, Fernando Motta, Anna Carolina Paixao, Ana Carolina Mendonca, Maria do Carmo Debur, Irina Nastassja Riediger, Marilda Siqueira
EPI_ISL_792686, EPI_ISL_792690, EPI_ISL_792693, EPI_ISL_792694, EPI_ISL_792705, EPI_ISL_792706	The National Institute of Public Health	State Veterinary Institute Prague	Nagy,A;Jirincova,H;Trnka,D;Vecerova,J
EPI_ISL_792828, EPI_ISL_793012	Department of Virus and Microbiological Special Diagnostics, Statens Serum Institut, Copenhagen, Denmark	Albertsen Lab, Department of Chemistry and Bioscience, Aalborg University, Denmark	Danish Covid-19 Genome Consortium
EPI_ISL_796777	Instituto Nacional de Saude (INSA)	Instituto Nacional de Saude (INSA)	Borges et al
EPI_ISL_800897, EPI_ISL_800920, EPI_ISL_800924, EPI_ISL_800967, EPI_ISL_800971, EPI_ISL_800995, EPI_ISL_801002, EPI_ISL_801055, EPI_ISL_801063, EPI_ISL_801110, EPI_ISL_801125, EPI_ISL_801148			
see above	Lighthouse Lab in Cambridge	Wellcome Sanger Institute for the COVID-19 Genomics UK (COG-UK) Consortium	Rob Howes, The Lighthouse Lab in Cambridge and Alex Alderton, Roberto Amato, Sonia Goncalves, Ewan Harrison, David K. Jackson, Ian Johnston, Dominic Kwiatkowski, Cordelia Langford, John Sillitoe on behalf of the Wellcome Sanger Institute COVID-19 Surveillance Team
EPI_ISL_801182, EPI_ISL_801183, EPI_ISL_801184, EPI_ISL_801185, EPI_ISL_801186, EPI_ISL_801187, EPI_ISL_801188, EPI_ISL_801189, EPI_ISL_801190, EPI_ISL_801191, EPI_ISL_801192, EPI_ISL_801193, EPI_ISL_801194, EPI_ISL_801195, EPI_ISL_801196, EPI_ISL_801197, EPI_ISL_801198, EPI_ISL_801199, EPI_ISL_801200, EPI_ISL_801201, EPI_ISL_801202, EPI_ISL_801203, EPI_ISL_801204, EPI_ISL_801205, EPI_ISL_801206, EPI_ISL_801207, EPI_ISL_801208, EPI_ISL_801209, EPI_ISL_801210, EPI_ISL_801211, EPI_ISL_801212, EPI_ISL_801213, EPI_ISL_801214, EPI_ISL_801215, EPI_ISL_801216, EPI_ISL_801217, EPI_ISL_801218, EPI_ISL_801219, EPI_ISL_801220, EPI_ISL_801221, EPI_ISL_801222, EPI_ISL_801223, EPI_ISL_801224, EPI_ISL_801225, EPI_ISL_801226, EPI_ISL_801227, EPI_ISL_801228, EPI_ISL_801229, EPI_ISL_801230			
see above	Lighthouse Lab in Milton Keynes	Wellcome Sanger Institute for the COVID-19 Genomics UK (COG-UK) Consortium	The Lighthouse Lab in Milton Keynes and Alex Alderton, Roberto Amato, Sonia Goncalves, Ewan Harrison, David K. Jackson, Ian Johnston, Dominic Kwiatkowski, Cordelia Langford, John Sillitoe on behalf of the Wellcome Sanger Institute COVID-19 Surveillance Team
EPI_ISL_802855	Vilnius University Hospital Santaros Klinikos, Vilnius University	Institute of Biotechnology, Life Sciences Center, Vilnius University	Emilija Vasiliunaite, Milda Norkiene, Albertas Timinskas, Alma Gedvilaite, Aurelija Zvirbliene, Daniel Naumovas, Laimonas Griskevicius
EPI_ISL_803101, EPI_ISL_803104	Respiratory Viruses Branch, Centers for Disease Control and Prevention	Respiratory Viruses Branch, Centers for Disease Control and Prevention	Queen,K., Li,Y., Tao,Y., Uehara,A., Montmayeur,A., Paden,C.R., Cook,P.W., Marine,R., Sheth,M., Wang,H., Lee,J., Tong,S.
EPI_ISL_804582	MEPHI, Aix Marseille University	MEPHI, Aix Marseille University	Anthony LEVASSEUR
EPI_ISL_804602, EPI_ISL_804608, EPI_ISL_804614, EPI_ISL_804615, EPI_ISL_804624, EPI_ISL_804631, EPI_ISL_804632, EPI_ISL_804634, EPI_ISL_804635, EPI_ISL_804641, EPI_ISL_804644, EPI_ISL_804645, EPI_ISL_804650, EPI_ISL_804651, EPI_ISL_804655, EPI_ISL_804657, EPI_ISL_804658, EPI_ISL_804661, EPI_ISL_804662, EPI_ISL_804666, EPI_ISL_804671, EPI_ISL_804673, EPI_ISL_804674, EPI_ISL_804675, EPI_ISL_804678, EPI_ISL_804682, EPI_ISL_804684, EPI_ISL_804688, EPI_ISL_804690, EPI_ISL_804692, EPI_ISL_804693, EPI_ISL_804698, EPI_ISL_804699, EPI_ISL_804702, EPI_ISL_804704, EPI_ISL_804710, EPI_ISL_804743, EPI_ISL_804754, EPI_ISL_804756, EPI_ISL_804757, EPI_ISL_804758, EPI_ISL_804759, EPI_ISL_804779, EPI_ISL_804781, EPI_ISL_804786, EPI_ISL_804790, EPI_ISL_804792, EPI_ISL_804806, EPI_ISL_804808			
see above	Michigan Department of Health and Human Services, Bureau of Laboratories	Michigan Department of Health and Human Services, Bureau of Laboratories	Blankenship HM, Riner D, Soehnlen MK
EPI_ISL_806819, EPI_ISL_806820, EPI_ISL_806821	Alaska State Virology Laboratory	Alaska State Virology Laboratory	Stephanie DeRonde, Lisa Smith, Ph.D., Devin M. Drown, Ph.D., Jack Chen, Ph.D.
EPI_ISL_807015, EPI_ISL_807021, EPI_ISL_807055, EPI_ISL_807056, EPI_ISL_807057, EPI_ISL_807058, EPI_ISL_807059, EPI_ISL_807060, EPI_ISL_807061, EPI_ISL_807062, EPI_ISL_807063, EPI_ISL_807064, EPI_ISL_807065, EPI_ISL_807066, EPI_ISL_807067, EPI_ISL_807068, EPI_ISL_807069, EPI_ISL_807070, EPI_ISL_807071, EPI_ISL_807072, EPI_ISL_807073, EPI_ISL_807074, EPI_ISL_807075, EPI_ISL_807076, EPI_ISL_807077, EPI_ISL_807078, EPI_ISL_807079, EPI_ISL_807080, EPI_ISL_807081, EPI_ISL_807082, EPI_ISL_807083, EPI_ISL_807084, EPI_ISL_807085, EPI_ISL_807086, EPI_ISL_807087, EPI_ISL_807089, EPI_ISL_807090, EPI_ISL_807091, EPI_ISL_807092, EPI_ISL_807093, EPI_ISL_807095, EPI_ISL_807096, EPI_ISL_807097, EPI_ISL_807098, EPI_ISL_807099, EPI_ISL_807100, EPI_ISL_807101, EPI_ISL_807102, EPI_ISL_807103, EPI_ISL_807104, EPI_ISL_807105, EPI_ISL_807106, EPI_ISL_807107, EPI_ISL_807109,			

EPI_ISL_807110, EPI_ISL_807111, EPI_ISL_807112, EPI_ISL_807115			
see above	Washington State Department of Health	Seattle Flu Study	Deborah A. Nickerson, Chris D. Frazar, Jover Lee, Benjamin Pelle, Matthew Richardson, Amanda Adler, Elisabeth Brandstetter, Peter D. Han, Kairsten Fay, Misja Ilcisin, Kirsten Lacombe, Thomas R. Sibley, Melissa Truong, Caitlin R. Wolf, Romesh Gautom, Geoff Melly, Brian Hiatt, Philip Dykema, Scott Lindquist, Michael Boeckh, Janet A. Englund, Michael Famulare, Barry R. Lutz, Mark J. Rieder, Lea M. Starita, Matthew Thompson, Helen Y. Chu, Jay Shendure, Trevor Bedford
EPI_ISL_812132, EPI_ISL_812139	TN Division of Laboratory Services	Pathogen Discovery, Respiratory Viruses Branch, Division of Viral Diseases, Centers for Disease Control and Prevention	Yan Li, Ying Tao, Anna Montmayeur, Jing Zhang, Brian Lynch, Krista Queen, Anna Uehara, Rachel Marine, Peter Cook, Clinton R. Paden, Haibin Wang, Suxiang Tong
EPI_ISL_812151, EPI_ISL_812152, EPI_ISL_812153, EPI_ISL_812154, EPI_ISL_812155, EPI_ISL_812197	GA Department of Public Health Laboratory	Pathogen Discovery, Respiratory Viruses Branch, Division of Viral Diseases, Centers for Disease Control and Prevention	Yan Li, Ying Tao, Anna Montmayeur, Jing Zhang, Brian Lynch, Krista Queen, Anna Uehara, Rachel Marine, Peter Cook, Clinton R. Paden, Haibin Wang, Suxiang Tong
EPI_ISL_812204, EPI_ISL_812205, EPI_ISL_812206, EPI_ISL_812207, EPI_ISL_812208, EPI_ISL_812209, EPI_ISL_812210, EPI_ISL_812211, EPI_ISL_812212, EPI_ISL_812213, EPI_ISL_812214, EPI_ISL_812215, EPI_ISL_812216, EPI_ISL_812217, EPI_ISL_812218, EPI_ISL_812220, EPI_ISL_812221, EPI_ISL_812222, EPI_ISL_812223, EPI_ISL_812224, EPI_ISL_812225, EPI_ISL_812226, EPI_ISL_812227, EPI_ISL_812228, EPI_ISL_812229, EPI_ISL_812230, EPI_ISL_812231			
see above	TN Division of Laboratory Services	Pathogen Discovery, Respiratory Viruses Branch, Division of Viral Diseases, Centers for Disease Control and Prevention	Yan Li, Ying Tao, Anna Montmayeur, Jing Zhang, Brian Lynch, Krista Queen, Anna Uehara, Rachel Marine, Peter Cook, Clinton R. Paden, Haibin Wang, Suxiang Tong
EPI_ISL_813751	Liverpool Clinical Laboratories	COVID-19 Genomics UK (COG-UK) Consortium	Sam Haldenby, Anita Lucaci, Steve Paterson, Julian Hiscox, Alistair Darby, M Almsaud, A Alrezaihi, Muhannad Alruwaili, Stuart D Armstrong, Jones Benjamin, Eleanor G Bentley, Anu Chawla, Jordan J Clark, Angela Cowell, Richard Eccles, Isabel García-Dorival, Matthew Gemmell, Alessandro Gerada, PKF Gilmore, Richard Gregory, Ximeng Han, Catherine Hartley, Margaret Hughes, Miren Iturriza-Gomara, James Johnson, L Luu, Jenifer Manson, Charlotte Nelson, Elaine O'Toole, Cassie Olateju, Rebekah Penrice-Randal , Lucille Rainbow, N.P Randle, Trevor Ian Robinson, Parul Sharma, Ghada T Shawli, James P Stewart, Neil Swainston, Ecaterina Vamos, Joanne Watts, Mark Whitehead
EPI_ISL_814534, EPI_ISL_814571	Virology Department, Royal Infirmary of Edinburgh, NHS Lothian / School of Biological Sciences, University of Edinburgh / Institute of Genetics and Molecular Medicine, University of Edinburgh	COVID-19 Genomics UK (COG-UK) Consortium	McHugh M, Dewar R, Rooke S, Gallagher M, Balcaza C, O'Toole Á, Scher E, Hill V, McCrone JT, Colquhoun R, Yu X, Jackson B, Rambaut A, Williams TC, Templeton K
EPI_ISL_814796, EPI_ISL_814797	Wales Specialist Virology Centre Sequencing lab: Pathogen Genomics Unit	COVID-19 Genomics UK (COG-UK) Consortium	Catherine Moore, Johnathan Evans, Laura Gifford, Malorie Perry, Simon Cottrell, Angela Marchbank, Alec Birchley, Alexander Adams, Amy Gaskin, Bree Gatica-Wilcox, Jason Coombes, Joel Southgate, Lauren Gilbert, Lee Graham, Nicole Pacchiarini, Sara Kumziene-Summerhayes, Sarah Taylor, Sophie Jones, Sara Rey, Matthew Bull, Joanne Watkins, Sally Corden, Tom Connor
EPI_ISL_815463, EPI_ISL_815466, EPI_ISL_815484, EPI_ISL_815558, EPI_ISL_815600, EPI_ISL_816095, EPI_ISL_816096, EPI_ISL_816097, EPI_ISL_816098, EPI_ISL_816099, EPI_ISL_816100, EPI_ISL_816101, EPI_ISL_816102, EPI_ISL_816103, EPI_ISL_816104, EPI_ISL_816105, EPI_ISL_816106, EPI_ISL_816107, EPI_ISL_816108, EPI_ISL_816109, EPI_ISL_816110			
see above	Department of Virus and Microbiological Special Diagnostics, Statens Serum Institut, Copenhagen, Denmark	Albertsen Lab, Department of Chemistry and Bioscience, Aalborg University, Denmark	Danish Covid-19 Genome Consortium
EPI_ISL_816324, EPI_ISL_816453	Virology Department, Sheffield Teaching Hospitals NHS Foundation Trust/Department of Infection, Immunity and Cardiovascular Disease, The Medical School, University of Sheffield	COVID-19 Genomics UK (COG-UK) Consortium	Thushan de Silva, Matthew Parker, Nikki Smith, Adri Angyal, Rebecca Brown, Luke Green, Rachel Tucker, Paul Parsons, Danielle Groves, Katie Johnson, Laura Carrilero, Alex Keeley, Dave Partridge, Matthew Wyles, Benjamin Lindsey, Mehmet Yavuz, Mohammad Raza, Cariad Evans
EPI_ISL_823970	Department of Homeless Services	New York City Public Health Laboratory	Jade Wang, et al.
EPI_ISL_823971	OCME Office Of Chief Medical Examiner	New York City Public Health Laboratory	Jade Wang, et al.
EPI_ISL_825035	B.J. Medical College and Civil hospital, Ahmedabad	Gujarat Biotechnology Research Centre	Nilima Shah, Sanjay Kapadia, Nitin Savaliya, Dinesh Kumar, Zuber Saiyed, Labdhi Pandya, Afzal Ansari, Nikha Trivedi, Apurvasinh Puvar, Ramesh Pandit, Janvi Raval, Zarna Patel, Kamlesh J Upadhyay, Pranay Shah, Dipa Kinariwala, Chaitanya Joshi, Madhvi Joshi
EPI_ISL_825092, EPI_ISL_825096, EPI_ISL_825097, EPI_ISL_825098, EPI_ISL_825099, EPI_ISL_825101, EPI_ISL_825104, EPI_ISL_825106, EPI_ISL_825107, EPI_ISL_825108	NHLS-IALCH	KRISP, KZN Research Innovation and Sequencing Platform	Giandhari J, Pillay S, Lessells R, Mdlalose K, York D, Khan S, Tegally H, Wilkinson E, de Oliveira T
EPI_ISL_825330, EPI_ISL_825331, EPI_ISL_825358, EPI_ISL_825359, EPI_ISL_825360	Hospital Universitari Vall d'Hebron - Vall d'Hebron Institut de Rercerca	Hospital Universitari Vall d'Hebron	Cristina Andrés, Maria Piñana, Josep F Abril, Damir Garcia-Cehic, Ariadna Rando, Juliana Esperalba, Maria Gema Codina, Carla Castillo, Maria Carmen Martin, Tomàs Pumarola, Josep Quer, Andrés Antón
EPI_ISL_825597, EPI_ISL_825598, EPI_ISL_825599, EPI_ISL_825600, EPI_ISL_825601, EPI_ISL_825602, EPI_ISL_825603, EPI_ISL_825604	Respiratory Virus Unit, National Infection Service, Public Health England	COVID-19 Genomics UK (COG-UK) Consortium	PHE Covid Sequencing Team
EPI_ISL_826569	Texas Department of State Health Services	Texas Department of State Health Services	Rashmi Tuladhar, Bonnie Oh, Jenny Zhang, Maliha Rahman, Anita Pokharel, Myong Koag, Chung Wang, Rachel Lee, Grace Kubin, Mayela Pedrueza, James Daniel Bonser
EPI_ISL_826723, EPI_ISL_826995, EPI_ISL_826998, EPI_ISL_827267	deCODE genetics	deCODE genetics	Daniel F Gudbjartsson; Agnar Helgason; Hakon Jonsson; Olafur T Magnusson; Pall Melsted; Gudmundur L Norddahl; Jona Saemundsdottir; Asgeir Sigurdsson; Patrick Sulem; Arna B Agustsdottir; Hannes Eggertsson; Berglind Eiriksdottir; Run Fridriksdottir; Elisabet E Gardarsdottir; Gudmundur Georgsson; Olafia S Gretarsdottir; Kjartan R Gudmundsson; Thora R Gunnarsdottir; Arnaldur Gylfason; Hilma Holm; Brynjar O Jensson; Aslaug Jonasdottir; Kamilla S Josefsdottir; Thordur Kristjansson; Droplaug N Magnusdottir; Solvi Rognvaldsson; Louise le Roux; Gudrun Sigmundsdottir; Gardar Sveinbjornsson; Kristin E Sveinsdottir; Maney Sveinsdottir; Emil A Thorarensen; Bjarni Thorbjornsson; Gisli Masson; Ingileif Jonsdottir; Alma Moller; Thorolfur Gudnason; Karl G Kristinsson; Unnur Thorsteinsdottir; Kari Stefansson
EPI_ISL_827395	The National University Hospital of Iceland	deCODE genetics	Daniel F Gudbjartsson; Agnar Helgason; Hakon Jonsson; Olafur T Magnusson; Pall Melsted; Gudmundur L Norddahl; Jona Saemundsdottir; Asgeir Sigurdsson; Patrick Sulem; Arna B Agustsdottir; Hannes Eggertsson; Berglind Eiriksdottir; Run Fridriksdottir; Elisabet E Gardarsdottir; Gudmundur Georgsson; Olafia S Gretarsdottir; Kjartan R Gudmundsson; Thora R Gunnarsdottir; Arnaldur Gylfason; Hilma Holm; Brynjar O Jensson; Aslaug Jonasdottir; Kamilla S Josefsdottir; Thordur Kristjansson; Droplaug N Magnusdottir; Solvi Rognvaldsson; Louise le Roux; Gudrun Sigmundsdottir; Gardar Sveinbjornsson; Kristin E Sveinsdottir; Maney Sveinsdottir; Emil A Thorarensen; Bjarni Thorbjornsson; Gisli Masson; Ingileif Jonsdottir; Alma Moller; Thorolfur Gudnason; Karl G Kristinsson; Unnur Thorsteinsdottir; Kari Stefansson
EPI_ISL_827397, EPI_ISL_827398, EPI_ISL_827399, EPI_ISL_827400, EPI_ISL_827402, EPI_ISL_827442, EPI_ISL_827458, EPI_ISL_827830, EPI_ISL_827852, EPI_ISL_827861, EPI_ISL_828110			
see above	deCODE genetics	deCODE genetics	Daniel F Gudbjartsson; Agnar Helgason; Hakon Jonsson; Olafur T Magnusson; Pall Melsted; Gudmundur L Norddahl; Jona Saemundsdottir; Asgeir Sigurdsson; Patrick Sulem; Arna B Agustsdottir; Hannes Eggertsson; Berglind Eiriksdottir; Run Fridriksdottir; Elisabet E Gardarsdottir; Gudmundur Georgsson; Olafia S Gretarsdottir; Kjartan R Gudmundsson; Thora R Gunnarsdottir; Arnaldur Gylfason; Hilma Holm; Brynjar O Jensson; Aslaug Jonasdottir; Kamilla S Josefsdottir; Thordur Kristjansson; Droplaug N Magnusdottir; Solvi Rognvaldsson; Louise le Roux; Gudrun Sigmundsdottir; Gardar Sveinbjornsson; Kristin E Sveinsdottir; Maney Sveinsdottir; Emil A Thorarensen; Bjarni Thorbjornsson; Gisli Masson; Ingileif Jonsdottir; Alma Moller; Thorolfur Gudnason; Karl G Kristinsson; Unnur Thorsteinsdottir; Kari Stefansson
EPI_ISL_828802, EPI_ISL_828804	The National University Hospital of Iceland	deCODE genetics	Daniel F Gudbjartsson; Agnar Helgason; Hakon Jonsson; Olafur T Magnusson; Pall Melsted; Gudmundur L Norddahl; Jona Saemundsdottir; Asgeir Sigurdsson; Patrick Sulem; Arna B Agustsdottir; Hannes Eggertsson; Berglind Eiriksdottir; Run Fridriksdottir; Elisabet E Gardarsdottir; Gudmundur Georgsson; Olafia S Gretarsdottir; Kjartan R Gudmundsson; Thora R Gunnarsdottir; Arnaldur Gylfason; Hilma Holm; Brynjar O Jensson; Aslaug

			Jonasdottir; Kamilla S Josefsdottir; Thordur Kristjansson; Droplaug N Magnusdottir; Solvi Rognvaldsson; Louise le Roux; Gudrun Sigmundsdottir; Gardar Sveinbjornsson; Kristin E Sveinsdottir; Maney Sveinsdottir; Emil A Thorarensen; Bjarni Thorbjornsson; Gisli Masson; Ingileif Jonsdottir; Alma Moller; Thorolfur Gudnason; Karl G Kristinsson; Unnur Thorsteinsdottir; Kari Stefansson
EPI_ISL_828964, EPI_ISL_829041	deCODE genetics	deCODE genetics	Daniel F Gudbjartsson; Agnar Helgason; Hakon Jonsson; Olafur T Magnusson; Pall Melsted; Gudmundur L Norddahl; Jona Saemundsdottir; Asgeir Sigurdsson; Patrick Sulem; Arna B Agustsdottir; Hannes Eggertsson; Berglind Eiriksdottir; Run Fridriksdottir; Elisabet E Gardarsdottir; Gudmundur Georgsson; Olafia S Gretarsdottir; Kjartan R Gudmundsson; Thora R Gunnarsdottir; Arnaldur Gylfason; Hilma Holm; Brynjar O Jensson; Aslaug Jonasdottir; Kamilla S Josefsdottir; Thordur Kristjansson; Droplaug N Magnusdottir; Solvi Rognvaldsson; Louise le Roux; Gudrun Sigmundsdottir; Gardar Sveinbjornsson; Kristin E Sveinsdottir; Maney Sveinsdottir; Emil A Thorarensen; Bjarni Thorbjornsson; Gisli Masson; Ingileif Jonsdottir; Alma Moller; Thorolfur Gudnason; Karl G Kristinsson; Unnur Thorsteinsdottir; Kari Stefansson
EPI_ISL_829105	The National University Hospital of Iceland	deCODE genetics	Daniel F Gudbjartsson; Agnar Helgason; Hakon Jonsson; Olafur T Magnusson; Pall Melsted; Gudmundur L Norddahl; Jona Saemundsdottir; Asgeir Sigurdsson; Patrick Sulem; Arna B Agustsdottir; Hannes Eggertsson; Berglind Eiriksdottir; Run Fridriksdottir; Elisabet E Gardarsdottir; Gudmundur Georgsson; Olafia S Gretarsdottir; Kjartan R Gudmundsson; Thora R Gunnarsdottir; Arnaldur Gylfason; Hilma Holm; Brynjar O Jensson; Aslaug Jonasdottir; Kamilla S Josefsdottir; Thordur Kristjansson; Droplaug N Magnusdottir; Solvi Rognvaldsson; Louise le Roux; Gudrun Sigmundsdottir; Gardar Sveinbjornsson; Kristin E Sveinsdottir; Maney Sveinsdottir; Emil A Thorarensen; Bjarni Thorbjornsson; Gisli Masson; Ingileif Jonsdottir; Alma Moller; Thorolfur Gudnason; Karl G Kristinsson; Unnur Thorsteinsdottir; Kari Stefansson
EPI_ISL_829198, EPI_ISL_829235	deCODE genetics	deCODE genetics	Daniel F Gudbjartsson; Agnar Helgason; Hakon Jonsson; Olafur T Magnusson; Pall Melsted; Gudmundur L Norddahl; Jona Saemundsdottir; Asgeir Sigurdsson; Patrick Sulem; Arna B Agustsdottir; Hannes Eggertsson; Berglind Eiriksdottir; Run Fridriksdottir; Elisabet E Gardarsdottir; Gudmundur Georgsson; Olafia S Gretarsdottir; Kjartan R Gudmundsson; Thora R Gunnarsdottir; Arnaldur Gylfason; Hilma Holm; Brynjar O Jensson; Aslaug Jonasdottir; Kamilla S Josefsdottir; Thordur Kristjansson; Droplaug N Magnusdottir; Solvi Rognvaldsson; Louise le Roux; Gudrun Sigmundsdottir; Gardar Sveinbjornsson; Kristin E Sveinsdottir; Maney Sveinsdottir; Emil A Thorarensen; Bjarni Thorbjornsson; Gisli Masson; Ingileif Jonsdottir; Alma Moller; Thorolfur Gudnason; Karl G Kristinsson; Unnur Thorsteinsdottir; Kari Stefansson
EPI_ISL_829617	The National University Hospital of Iceland	deCODE genetics	Daniel F Gudbjartsson; Agnar Helgason; Hakon Jonsson; Olafur T Magnusson; Pall Melsted; Gudmundur L Norddahl; Jona Saemundsdottir; Asgeir Sigurdsson; Patrick Sulem; Arna B Agustsdottir; Hannes Eggertsson; Berglind Eiriksdottir; Run Fridriksdottir; Elisabet E Gardarsdottir; Gudmundur Georgsson; Olafia S Gretarsdottir; Kjartan R Gudmundsson; Thora R Gunnarsdottir; Arnaldur Gylfason; Hilma Holm; Brynjar O Jensson; Aslaug Jonasdottir; Kamilla S Josefsdottir; Thordur Kristjansson; Droplaug N Magnusdottir; Solvi Rognvaldsson; Louise le Roux; Gudrun Sigmundsdottir; Gardar Sveinbjornsson; Kristin E Sveinsdottir; Maney Sveinsdottir; Emil A Thorarensen; Bjarni Thorbjornsson; Gisli Masson; Ingileif Jonsdottir; Alma Moller; Thorolfur Gudnason; Karl G Kristinsson; Unnur Thorsteinsdottir; Kari Stefansson
EPI_ISL_829726, EPI_ISL_829727, EPI_ISL_829728, EPI_ISL_829729, EPI_ISL_829730, EPI_ISL_829731, EPI_ISL_829976, EPI_ISL_829980, EPI_ISL_829983, EPI_ISL_829985, EPI_ISL_830157, EPI_ISL_830158, EPI_ISL_830159, EPI_ISL_830160, EPI_ISL_830161			
see above	deCODE genetics	deCODE genetics	Daniel F Gudbjartsson; Agnar Helgason; Hakon Jonsson; Olafur T Magnusson; Pall Melsted; Gudmundur L Norddahl; Jona Saemundsdottir; Asgeir Sigurdsson; Patrick Sulem; Arna B Agustsdottir; Hannes Eggertsson; Berglind Eiriksdottir; Run Fridriksdottir; Elisabet E Gardarsdottir; Gudmundur Georgsson; Olafia S Gretarsdottir; Kjartan R Gudmundsson; Thora R Gunnarsdottir; Arnaldur Gylfason; Hilma Holm; Brynjar O Jensson; Aslaug Jonasdottir; Kamilla S Josefsdottir; Thordur Kristjansson; Droplaug N Magnusdottir; Solvi Rognvaldsson; Louise le Roux; Gudrun Sigmundsdottir; Gardar Sveinbjornsson; Kristin E Sveinsdottir; Maney Sveinsdottir; Emil A Thorarensen; Bjarni Thorbjornsson; Gisli Masson; Ingileif Jonsdottir; Alma Moller; Thorolfur Gudnason; Karl G Kristinsson; Unnur Thorsteinsdottir; Kari Stefansson
EPI_ISL_830235, EPI_ISL_830237, EPI_ISL_830240, EPI_ISL_830242, EPI_ISL_830245	The National University Hospital of Iceland	deCODE genetics	Daniel F Gudbjartsson; Agnar Helgason; Hakon Jonsson; Olafur T Magnusson; Pall Melsted; Gudmundur L Norddahl; Jona Saemundsdottir; Asgeir Sigurdsson; Patrick Sulem; Arna B Agustsdottir; Hannes Eggertsson; Berglind Eiriksdottir; Run Fridriksdottir; Elisabet E Gardarsdottir; Gudmundur Georgsson; Olafia S Gretarsdottir; Kjartan R Gudmundsson; Thora R Gunnarsdottir; Arnaldur Gylfason; Hilma Holm; Brynjar O Jensson; Aslaug Jonasdottir; Kamilla S Josefsdottir; Thordur Kristjansson; Droplaug N Magnusdottir; Solvi Rognvaldsson; Louise le Roux; Gudrun Sigmundsdottir; Gardar Sveinbjornsson; Kristin E Sveinsdottir; Maney Sveinsdottir; Emil A Thorarensen; Bjarni Thorbjornsson; Gisli Masson; Ingileif Jonsdottir; Alma Moller; Thorolfur Gudnason; Karl G Kristinsson; Unnur Thorsteinsdottir; Kari Stefansson
EPI_ISL_831032, EPI_ISL_831202, EPI_ISL_831205, EPI_ISL_831206	Hospital Universitario La Paz (Madrid)	SeqCOVID-SPAIN consortium/IBV(CSIC)	María Rodríguez-Tejedor, Elias Dahdouh, Fernando Lázaro-Perona, Jesús Mingorance and SeqCOVID-SPAIN consortium
EPI_ISL_831707, EPI_ISL_831717, EPI_ISL_831720, EPI_ISL_831721, EPI_ISL_831722, EPI_ISL_831724, EPI_ISL_831725, EPI_ISL_831726, EPI_ISL_831727, EPI_ISL_831728, EPI_ISL_831729, EPI_ISL_831744, EPI_ISL_831753, EPI_ISL_831755, EPI_ISL_831756, EPI_ISL_831803, EPI_ISL_831804, EPI_ISL_831805, EPI_ISL_831806, EPI_ISL_831807, EPI_ISL_831808, EPI_ISL_831809, EPI_ISL_831810, EPI_ISL_831811, EPI_ISL_831812, EPI_ISL_831822, EPI_ISL_831823, EPI_ISL_831824			
see above	United States Air Force School of Aerospace Medicine	United States Air Force School of Aerospace Medicine	Anthony Fries, Jennifer Meyer, William Gruner, Amanda Javorina, Sarah Purves, Clarise Starr, Elizabeth Macias
EPI_ISL_832758, EPI_ISL_832759, EPI_ISL_832760, EPI_ISL_832776, EPI_ISL_832777, EPI_ISL_832778, EPI_ISL_832779, EPI_ISL_832780, EPI_ISL_832783, EPI_ISL_832784, EPI_ISL_832785, EPI_ISL_832786, EPI_ISL_832787, EPI_ISL_832788, EPI_ISL_832789, EPI_ISL_832790, EPI_ISL_832791, EPI_ISL_832792, EPI_ISL_832793			
see above	OHSU Lab Services Molecular Microbiology Lab	Oregon SARS-CoV-2 Genome Sequencing Center	Brendan L. O'Connell, Ruth V. Nichols, Sally Grindstaff, Alex J. Hirsch, Donna Hansel, Guang Fan, Daniel N. Streblow, William B. Messer, Andrew C. Adey, Benjamin N. Bimber, Brian J. O'Roak
EPI_ISL_833157	Instituto Adolfo Lutz - Regional de Santo Andre	Instituto Adolfo Lutz, Interdiciplinary Procedures Center, Strategic Laboratory	Claudio Tavares Sacchi, Claudia Regina Gonçalves, Erica Valessa Ramos Gomes, Karoline Rodrigues Campos
EPI_ISL_833163	Instituto Adolfo Lutz - Regional de Marilia	Instituto Adolfo Lutz, Interdiciplinary Procedures Center, Strategic Laboratory	Claudio Tavares Sacchi, Claudia Regina Gonçalves, Erica Valessa Ramos Gomes, Karoline Rodrigues Campos
EPI_ISL_833463, EPI_ISL_833471	CHU Purpan - Laboratoire de Virologie - Institut Fédératif de Biologie	CHU Purpan - Laboratoire de Virologie - Institut Fédératif de Biologie	Latour J., Ranger N., Dubois M., Carcenac R., Harter A., Boyer P., Tremeaux P., Izopet J.
EPI_ISL_838349	Queens Medical Centre, Clinical Microbiology Department / DeepSeq Nottingham	COVID-19 Genomics UK (COG-UK) Consortium	Gemma Clark, Wendy Smith, Manjinder Khakh, Vicki M Fleming, Michelle M Lister, Hannah Howson-Wells, Jonathan Ball, Patrick McClure, Joseph Chappell, Theocharis Tsoleridis, Nadine Holmes, Matthew Carlisle, Christopher Moore, Fei Sang, Johnny Debebe, Victoria Wright, Matthew Loose
EPI_ISL_840186, EPI_ISL_840187, EPI_ISL_840190, EPI_ISL_840191, EPI_ISL_840192, EPI_ISL_840193, EPI_ISL_840194, EPI_ISL_840195, EPI_ISL_840196, EPI_ISL_840197, EPI_ISL_840198, EPI_ISL_840199, EPI_ISL_840207			
see above	Oxford Viromics, NDM, University of Oxford; Oxford University Hospitals; Basingstoke and North Hampshire Hospital	COVID-19 Genomics UK (COG-UK) Consortium	Tanya Golubchik, David Bonsall, George Macintyre, Amy Trebes, Mariateresa de Cesare, Catrin Moore, Alex Mobbs, Anita Justice, Robert Shaw, Monique Andersson, Timothy Peto, Emma Wise, Nathan Moore, Jessica Lynch, Nick Cortes, Matilde Mori, Stephen Kidd, David Buck, John Todd, Christophe Fraser
EPI_ISL_842204	Virology Department, Sheffield Teaching Hospitals NHS Foundation Trust/Department of Infection, Immunity and Cardiovascular Disease, The Medical School, University of Sheffield	COVID-19 Genomics UK (COG-UK) Consortium	Thushan de Silva, Matthew Parker, Nikki Smith, Adri Angyal, Rebecca Brown, Luke Green, Rachel Tucker, Paul Parsons, Danielle Groves, Katie Johnson, Laura Carrilero, Alex Keeley, Dave Partridge, Matthew Wyles, Benjamin Lindsey, Mehmet Yavuz, Mohammad Raza, Cariad Evans
EPI_ISL_842910, EPI_ISL_842911, EPI_ISL_842912, EPI_ISL_842913, EPI_ISL_842914, EPI_ISL_842915	Barts Health NHS Trust	COVID-19 Genomics UK (COG-UK) Consortium	CUTINO-MOGUEL, Maria-Teresa; HARRINGTON, David; OWOYEMI, Dola; SHYLINI, Raghavendran; BROAD, Claire; KELE, Beatrix
EPI_ISL_844234, EPI_ISL_844299, EPI_ISL_844300, EPI_ISL_844539, EPI_ISL_844540	Department of Virus and Microbiological Special Diagnostics, Statens Serum Institut, Copenhagen, Denmark	Albertsen Lab, Department of Chemistry and Bioscience, Aalborg University, Denmark	Danish Covid-19 Genome Consortium
EPI_ISL_845557, EPI_ISL_845558, EPI_ISL_845559, EPI_ISL_845560, EPI_ISL_845561, EPI_ISL_845562	National Public Health Laboratory, Cameroon	African Centre of Excellence for Genomics of Infectious Diseases (ACEGID), Redeemer's University	Oluniyi P.E. et al
EPI_ISL_846656, EPI_ISL_846659, EPI_ISL_846664, EPI_ISL_846665, EPI_ISL_846666, EPI_ISL_846667, EPI_ISL_846669, EPI_ISL_846670, EPI_ISL_846671, EPI_ISL_846676, EPI_ISL_846677, EPI_ISL_846682, EPI_ISL_846684, EPI_ISL_846685, EPI_ISL_846686, EPI_ISL_846690, EPI_ISL_846691, EPI_ISL_846693, EPI_ISL_846695, EPI_ISL_846696, EPI_ISL_846698, EPI_ISL_846702, EPI_ISL_846703, EPI_ISL_846736, EPI_ISL_846737, EPI_ISL_846738, EPI_ISL_846739, EPI_ISL_846740, EPI_ISL_846741, EPI_ISL_846742, EPI_ISL_846743, EPI_ISL_846744, EPI_ISL_846745, EPI_ISL_846747, EPI_ISL_846767, EPI_ISL_846768, EPI_ISL_846769, EPI_ISL_846770, EPI_ISL_846771, EPI_ISL_846772, EPI_ISL_846773			

see above	University of Michigan Clinical Microbiology Laboratory	Lauring Lab, University of Michigan, Department of Microbiology and Immunology	Valesano
EPI_ISL_847524, EPI_ISL_847539, EPI_ISL_847576, EPI_ISL_847582, EPI_ISL_847586, EPI_ISL_847695, EPI_ISL_847696, EPI_ISL_847697, EPI_ISL_847698, EPI_ISL_847699, EPI_ISL_847700, EPI_ISL_847701, EPI_ISL_847702, EPI_ISL_847727, EPI_ISL_847732, EPI_ISL_847754, EPI_ISL_847816, EPI_ISL_847817, EPI_ISL_847818			
see above	California Department of Public Health	Chiu Laboratory, University of California, San Francisco	Charles Chiu, Xianding (Wayne) Deng, Candace Wang, Brian Bushnell, Scot Federman, Jill Hacker, Debra Wadford
EPI_ISL_848211, EPI_ISL_848213, EPI_ISL_848214, EPI_ISL_848217, EPI_ISL_848221, EPI_ISL_848225, EPI_ISL_848233, EPI_ISL_848240, EPI_ISL_848241, EPI_ISL_848242, EPI_ISL_848250, EPI_ISL_848256, EPI_ISL_848257, EPI_ISL_848258, EPI_ISL_848264, EPI_ISL_848273, EPI_ISL_848385, EPI_ISL_848386, EPI_ISL_848387, EPI_ISL_848388, EPI_ISL_848389, EPI_ISL_848390, EPI_ISL_848391, EPI_ISL_848392, EPI_ISL_848393, EPI_ISL_848394, EPI_ISL_848395, EPI_ISL_848396, EPI_ISL_848442, EPI_ISL_848444, EPI_ISL_848454, EPI_ISL_848465, EPI_ISL_848524, EPI_ISL_848525, EPI_ISL_848526			
see above	Illinois Department of Public Health	Gagnon Lab, Southern Illinois University	Keith Gagnon
EPI_ISL_848758, EPI_ISL_848759, EPI_ISL_848760, EPI_ISL_848761, EPI_ISL_848762, EPI_ISL_848763, EPI_ISL_848764, EPI_ISL_848765, EPI_ISL_848766, EPI_ISL_848767, EPI_ISL_848768, EPI_ISL_848769, EPI_ISL_848770, EPI_ISL_848771, EPI_ISL_848772, EPI_ISL_848773, EPI_ISL_848774, EPI_ISL_848775, EPI_ISL_848776, EPI_ISL_848777, EPI_ISL_848778, EPI_ISL_848779, EPI_ISL_848780, EPI_ISL_848781, EPI_ISL_848782, EPI_ISL_848783, EPI_ISL_848784, EPI_ISL_848785, EPI_ISL_848786, EPI_ISL_848787, EPI_ISL_848788, EPI_ISL_848789, EPI_ISL_848790, EPI_ISL_848791, EPI_ISL_848792, EPI_ISL_848793, EPI_ISL_848794, EPI_ISL_848795, EPI_ISL_848796, EPI_ISL_848797, EPI_ISL_848798, EPI_ISL_848799, EPI_ISL_848800, EPI_ISL_848801, EPI_ISL_848802, EPI_ISL_848803, EPI_ISL_848804, EPI_ISL_848805, EPI_ISL_848806, EPI_ISL_848807, EPI_ISL_848808, EPI_ISL_848809			
see above	Florida Bureau of Public Health Laboratories	Florida Bureau of Public Health Laboratories	Sarah Schmedes, Jason Blanton
EPI_ISL_849516, EPI_ISL_849517	Seattle Flu Study	Seattle Flu Study	Deborah A. Nickerson, Chris D. Frazar, Jover Lee, Benjamin Pelle, Matthew Richardson, Amanda Adler, Elisabeth Brandstetter, Peter D. Han, Kairsten Fay, Misja Ilcisin, Kirsten Lacombe, Thomas R. Sibley, Melissa Truong, Caitlin R. Wolf, Michael Boeckh, Janet A. Englund, Michael Famulare, Barry R. Lutz, Mark J. Rieder, Lea M. Starita, Matthew Thompson, Jay Shendure, Trevor Bedford, Helen Y. Chu
EPI_ISL_849686	unknown	PHV-FSS	Son Nguyen et al.
EPI_ISL_849948, EPI_ISL_849951	UC Davis- Department of Pathology and Laboratory Medicine	Chan-Zuckerberg Biohub	CZB Cliahub Consortium
EPI_ISL_853329, EPI_ISL_853332	UPMC Clinical Microbiology Laboratory	Microbial Genome Sequencing Center; Microbial Genomic Epidemiology Laboratory	Mustapha M. Mustapha, Jane W. Marsh, Dan Snyder, Marissa P. Griffith, Stephanie L. Mitchell, Vatsala R. Srinivasa, Kady D. Waggle, Chinelo Ezeonwuku, Vaughn S. Cooper, Lee H. Harrison
EPI_ISL_853767, EPI_ISL_853792, EPI_ISL_853933, EPI_ISL_853942, EPI_ISL_853945	Department of Microbiology, University Innsbruck	Bergthaler laboratory, CeMM Research Center for Molecular Medicine of the Austrian Academy of Sciences	Lukas Endler, Alexandra Popa, Benedikt Agerer, Jakob-Wendelin Genger, Alexander Lercher, Anna Schedl, Thomas Penz, Michael Schuster, Jan Laine, Martin Senekowitsch, Christoph Bock, Andreas Bergthaler
EPI_ISL_855389, EPI_ISL_855390, EPI_ISL_855391	California Department of Public Health	Chiu Laboratory, University of California, San Francisco	Charles Chiu, Xianding (Wayne) Deng, Candace Wang, Brian Bushnell, Scot Federman, Jill Hacker, Debra Wadford
EPI_ISL_855499, EPI_ISL_855516, EPI_ISL_855521, EPI_ISL_855528, EPI_ISL_855529	KEMRI-Wellcome Trust Research Programme/KEMRI-CGMR-C Kilifi	KEMRI-Wellcome Trust Research Programme/KEMRI-CGMR-C Kilifi	Githinji et al
EPI_ISL_856760	Servicio Virosis Respiratorias-Departamento Virología-INEI	Instituto Nacional Enfermedades Infecciosas C.G.Malbran	Baumeister E., Avaro M., Benedetti E., Russo M., Dattero ME, Pontoriero A., Cisterna D., Molina V., Perandones C., Tuduri E., Lorenzo F., Poklepovich T., Campos J.
EPI_ISL_857049	Colorado Department of Public Health and Environment	Colorado Department of Puplic Health and Environment	Laura Bankers, Molly C. Hetherington-Rauth, Diana Ir, Shannon Ely, Shannon R. Matzinger, Sarah Elizabeth Totten, Emily A. Travanty
EPI_ISL_857496, EPI_ISL_857497, EPI_ISL_857513	Swiss National Reference Centre for Influenza	Swiss National Reference Centre for Influenza	Ana Rita Goncalves,Samuel Cordey, Laurent Kaiser, Lorenzo Cerutti, Henri Pegeot, Melyssa Elies, Keith Harshman, Ioannis Xenarios, Emmanouil Dermitzakis
EPI_ISL_857520, EPI_ISL_857540	Swiss National Reference Centre for Influenza	Swiss National Reference Centre for Influenza	Tim Roloff, Ana Rita Gonçalves, Madlen Stange, Helena MB Seth-Smith, Alfredo Mari, Karoline Leuzinger, Julia Bielicki, Manuel Battegay, Hans Hirsch, Laurent Kaiser, Adrian Egli
EPI_ISL_859555, EPI_ISL_859560	Lighthouse Lab in Cambridge	Wellcome Sanger Institute for the COVID-19 Genomics UK (COG-UK) Consortium	Rob Howes, The Lighthouse Lab in Cambridge and Alex Alderton, Roberto Amato, Sonia Goncalves, Ewan Harrison, David K. Jackson, Ian Johnston, Dominic Kwiatkowski, Cordelia Langford, John Sillitoe on behalf of the Wellcome Sanger Institute COVID-19 Surveillance Team
EPI_ISL_859846, EPI_ISL_859847, EPI_ISL_859848, EPI_ISL_859849, EPI_ISL_859850, EPI_ISL_859851, EPI_ISL_859852, EPI_ISL_859853, EPI_ISL_859865, EPI_ISL_859866, EPI_ISL_859867, EPI_ISL_859868, EPI_ISL_859869, EPI_ISL_859870, EPI_ISL_859871, EPI_ISL_859872, EPI_ISL_859873, EPI_ISL_859874, EPI_ISL_859875, EPI_ISL_859887, EPI_ISL_859902, EPI_ISL_859903, EPI_ISL_859924, EPI_ISL_859925, EPI_ISL_859926, EPI_ISL_859927, EPI_ISL_859928			
see above	BTC, Khalifa University	BTC, Khalifa University	Al Safar et al
EPI_ISL_860123, EPI_ISL_860134, EPI_ISL_860135, EPI_ISL_860149, EPI_ISL_860152, EPI_ISL_860153, EPI_ISL_860156, EPI_ISL_860158, EPI_ISL_860159	Keio University School of Medicine	Keio University School of Medicine	Kenjiro Kosaki, Yuka Iwasaki, Hirotsugu Ishizu, Haruhiko Siomi, Kodai Abe
EPI_ISL_861887, EPI_ISL_861888	LATE - Laboratório de Técnicas Especiais - Hospital Israelita Albert Einstein	LATE - Laboratório de Técnicas Especiais - Hospital Israelita Albert Einstein	Deyvid Amgarten, Fernanda de Mello Malta, Raquel Riyuzo, Ana Paula Moreira Salles, Pedro Henrique Sebe Rodrigues, João Renato Rebello Pinho
EPI_ISL_864573	Institute of Medical Microbiology and Hospital Hygiene	Institute of Medical Microbiology and Hospital Hygiene	Prof. Dr. Achim Kaasch, Aljoscha Tersteegen
EPI_ISL_865173	Liverpool Clinical Laboratories	COVID-19 Genomics UK (COG-UK) Consortium	Sam Haldenby, Anita Lucaci, Steve Paterson, Julian Hiscox, Alistair Darby, M Almsaud, A Alrezaihi, Muhannad Alruwaili, Stuart D Armstrong, Jones Benjamin, Eleanor G Bentley, Anu Chawla, Jordan J Clark, Angela Cowell, Richard Eccles, Isabel García-Dorival, Matthew Gemmell, Alessandro Gerada, PKF Gilmore, Richard Gregory, Ximeng Han, Catherine Hartley, Margaret Hughes, Miren Iturriza-Gomara, James Johnson, L Luu, Jenifer Manson, Charlotte Nelson, Elaine O'Toole, Cassie Olateju, Rebekah Penrice-Randal , Lucille Rainbow, N.P Randle, Trevor Ian Robinson, Parul Sharma, Ghada T Shawli, James P Stewart, Neil Swainston, Ecaterina Vamos, Joanne Watts, Mark Whitehead
EPI_ISL_865683, EPI_ISL_865697, EPI_ISL_865698, EPI_ISL_865699, EPI_ISL_865700, EPI_ISL_865701	University College London, Great Ormond Street Hospital for Children NHS Foundation Trust, Imperial College Healthcare NHS Trust	COVID-19 Genomics UK (COG-UK) Consortium	Sergi Castellano, Rachel Williams, Mark Kristiansen, Paola Resende Silva, Sunando Roy, Tony Brooks, Helena Tutill, Paola Niola, Patricia Dyal, Charlotte Williams, Leysa Forrest, Yasmin Panchbhaya, Jacqueline Findlay, Samuel Weeks, Julianne Brown, Kathryn Harris, Paul Randell, James Price, Alison Holmes, Judith Breuer
EPI_ISL_866054	University College London Hospital	COVID-19 Genomics UK (COG-UK) Consortium	Judith Heaney, Matthew Byott, Catherine Houlihan, Dan Frampton, Stuart Kirk, Moira Spyer and Eleni Nastouli
EPI_ISL_872607	Nigeria Centre for Disease Control (NCDC)	African Centre of Excellence for Genomics of Infectious Diseases (ACEGID), Redeemer's University	Oluniyi P.E. et al
EPI_ISL_872692, EPI_ISL_872693, EPI_ISL_872694, EPI_ISL_872695, EPI_ISL_872696	Rhode Island Department of Health	Infectious Disease Program, Broad Institute of Harvard and MIT	Lemieux,J.E., Siddle,K.J., Huard,R., King,E., Azevedo,K., Miller,A., Adams,G., Gladden-Young,A., Lagerborg,K., Rudy,M., DeRuff,K., Carter,A., Normandin,E., Bauer,M., Reilly,S., Tomkins-Tinch,C., Loreth,C., Chaluvadi,S., Birren,B.W., Gallagher,G., Smole,S., Park,D.J., MacInnis,B.L., and Sabeti,P.C.
EPI_ISL_873132, EPI_ISL_873135, EPI_ISL_873138, EPI_ISL_873140, EPI_ISL_873143, EPI_ISL_873144, EPI_ISL_873146, EPI_ISL_873154	University of Michigan Clinical Microbiology Laboratory	Lauring Lab, University of Michigan, Department of Microbiology and Immunology	Valesano
EPI_ISL_876189, EPI_ISL_876325, EPI_ISL_876326, EPI_ISL_876327	Massachusetts State Public Health Laboratory	Massachusetts State Public Health Laboratory	Andrew Lang, Timelia Fink, Glen Gallagher, Sandra Smole
EPI_ISL_876567	Florida Bureau of Public Health Laboratories	Florida Bureau of Public Health Laboratories	Sarah Schmedes, Jason Blanton
EPI_ISL_877331, EPI_ISL_877332, EPI_ISL_877333, EPI_ISL_877334, EPI_ISL_877335, EPI_ISL_877336, EPI_ISL_877337, EPI_ISL_877338, EPI_ISL_877339, EPI_ISL_877340, EPI_ISL_877341, EPI_ISL_877342, EPI_ISL_877343, EPI_ISL_877344, EPI_ISL_877345, EPI_ISL_877346, EPI_ISL_877347, EPI_ISL_877348, EPI_ISL_877349, EPI_ISL_877350, EPI_ISL_877351, EPI_ISL_877352, EPI_ISL_877353, EPI_ISL_877354, EPI_ISL_877355, EPI_ISL_877356, EPI_ISL_877357, EPI_ISL_877358, EPI_ISL_877359, EPI_ISL_877360, EPI_ISL_877361, EPI_ISL_877362, EPI_ISL_877363, EPI_ISL_877364, EPI_ISL_877365, EPI_ISL_877366, EPI_ISL_877367, EPI_ISL_877368, EPI_ISL_877369, EPI_ISL_877370, EPI_ISL_877371, EPI_ISL_877372, EPI_ISL_877373, EPI_ISL_877374, EPI_ISL_877375, EPI_ISL_877376, EPI_ISL_877377, EPI_ISL_877378, EPI_ISL_877379, EPI_ISL_877380, EPI_ISL_877381, EPI_ISL_877382, EPI_ISL_877383, EPI_ISL_877384,			

EPI_ISL_877385, EPI_ISL_877386, EPI_ISL_877387, EPI_ISL_877388, EPI_ISL_877389, EPI_ISL_877390, EPI_ISL_877391, EPI_ISL_877392, EPI_ISL_877393, EPI_ISL_877394, EPI_ISL_877395, EPI_ISL_877396, EPI_ISL_877397, EPI_ISL_877398, EPI_ISL_877399, EPI_ISL_877400, EPI_ISL_877401, EPI_ISL_877402, EPI_ISL_877403, EPI_ISL_877404, EPI_ISL_877405, EPI_ISL_877406, EPI_ISL_877407, EPI_ISL_877408, EPI_ISL_877409, EPI_ISL_877410			
see above	Eurofins Diatherix	Hudsonalpha Genome Sequencing Center	Jane Grimwood, Melissa Williams, Lori H. Handley, Joshua Stough, Leslie Malone, Stefan Brzezinski, Ada Stewart, Teresa Jones, Jenell Webber, John Lovell, Jennifer Cart, and Jeremy Schmutz
EPI_ISL_878399, EPI_ISL_878403, EPI_ISL_878405, EPI_ISL_878407, EPI_ISL_878431	San Diego County Public Health Laboratory	Andersen lab at Scripps Research	SEARCH Alliance San Diego with Tracy Basler, Jovan Shephard, Brett Austin
EPI_ISL_878547	Robert Garry lab	Andersen lab at Scripps Research	Allison Smither, Gilberto Sabino-Santos, Patricia Snarski, Lilia Melnik, Antoinette Bell, Kaylynn Genemaras, Arnaud Drouin, Dahlene Fusco, Robert Garry with SEARCH Alliance San Diego
EPI_ISL_878682, EPI_ISL_878684, EPI_ISL_878726	Rady's Childrens Hospital	Andersen lab at Scripps Research	SEARCH Alliance San Diego with Nanda Radamchar, David Dimmock, Linda Luo, Christina Clarke, Kathryn Bouic, Teresa Mueller, Denise Malicki
EPI_ISL_882688, EPI_ISL_882689, EPI_ISL_882697, EPI_ISL_882698, EPI_ISL_882699, EPI_ISL_882700, EPI_ISL_882701, EPI_ISL_882704, EPI_ISL_882705, EPI_ISL_882706, EPI_ISL_882707, EPI_ISL_882708			
see above	1.AO Universitaria 'S. Giovanni di Dio e Ruggi D'Aragona, Scuola Medica Salernitana' Hospital / 2.UOC di Virologia e Microbiologia, Università della Campania 'L. Vanvitelli' / 3.AO Universitaria 'Federico II' Napoli Hospital / 4.AORN 'San Giuseppe Moscati' Avellino Hospital / 5.AO 'San Pio - presidio G. Rummo' Benevento Hospital / 6.AO 'Sant'Anna e San Sebastiano' Caserta Hospital / 7.PO 'Maria Santissima Addolorata' Eboli Hospital / 8.Biogem Istituto di Ricerche Genetiche	1. Genome Research Center for Health (CRGS) / 2. Laboratory of Molecular Medicine and Genomics(LMMGe) / 3. Center for Research in Pure and Applied Mathematics (CRMPA)	Giorgio Giurato, Francesca Rizzo, Alessandro Weisz, Gianluigi Franci, Giovanni Nassa, Pasquale Pagliano, Roberta Tarallo, Elena Alexandrova, Ylenia D'Agostino, Carlo Ferravante, Jessica Lamberti, Viola Melone, Domenico Memoli, Valeria Mirici Cappa, Domenico Palumbo, Giovanni Pecoraro, Assunta Sellitto, Oriana Strianese, Ilaria Terenzi, Giuseppe Fenza, Aniello Gentile, Antonello Saccomanno, Sonia Amabile, Teresa Rocco, Annamaria Salvati, Emilia Vaccaro, Massimiliano Galdiero, Michele Cennamo, Giuseppe Portella, Maria Grazia Foti, Mariarosaria Ingino, Maria Landi, Maurizio Fumi, Vincenzo Rocco, Rita Greco, Vittoria Letizia, Arnolfo Petruzziello, Maddalena Schioppa, Gregorio Goffredi, Francesca Marciano, Michele Caraglia, Alessia Cossu, Marianna Scrima
EPI_ISL_882957	Specjalistyczny Szpital im. Dra A. Sokoowskiego	National Institute of Public Health - National Institute of Hygiene	Wokowicz Tomasz, Zacharczuk Katarzyna, Gawor Jan
EPI_ISL_885130	California Institute of Technology	Chan-Zuckerberg Biohub	CZB Cliahub Consortium
EPI_ISL_888836	Michigan Department of Health and Human Services, Bureau of Laboratories	Michigan Department of Health and Human Services, Bureau of Laboratories	Blankenship HM, Riner D, Soehnlen MK
EPI_ISL_888988	RS Qadr	Eijkman Institute for Molecular Biology, Ministry of Research and Technology/National Agency for Research and Innovation	Frilasita A Yudhaputri, Hidayat Trimarsanto, Iskandar Adnan, Lydia V. Panggalo, Sukma Oktavianthi, Willy Agustine, Edison Johar, Safarina G Malik, Khin Saw Myint, Amin Soebandrio
EPI_ISL_888989	RS Pondok Indah Bintaro Jaya	Eijkman Institute for Molecular Biology, Ministry of Research and Technology/National Agency for Research and Innovation	Frilasita A Yudhaputri, Hidayat Trimarsanto, Iskandar Adnan, Lydia V. Panggalo, Sukma Oktavianthi, Willy Agustine, Edison Johar, Safarina G Malik, Khin Saw Myint, Amin Soebandrio
EPI_ISL_889010	RSU Medika Dramaga	Eijkman Institute for Molecular Biology, Ministry of Research and Technology/National Agency for Research and Innovation	Frilasita A Yudhaputri, Hidayat Trimarsanto, Iskandar Adnan, Lydia V. Panggalo, Sukma Oktavianthi, Willy Agustine, Edison Johar, Safarina G Malik, Khin Saw Myint, Amin Soebandrio
EPI_ISL_891195	DPH, Massachusetts State Public Health Lab	DPH, Massachusetts State Public Health Lab	Lang,A.S., Fink,T., Gallagher,G.R., Smole,S.C.
EPI_ISL_902996, EPI_ISL_902997, EPI_ISL_902998, EPI_ISL_902999, EPI_ISL_903000, EPI_ISL_903001, EPI_ISL_903003	Seattle Flu Study	Seattle Flu Study	Deborah A. Nickerson, Chris D. Frazar, Jover Lee, Benjamin Pelle, Erica Ryke, Matthew Richardson, Amanda Adler, Elisabeth Brandstetter, Peter D. Han, Kairsten Fay, Misja Ilcisin, Kirsten Lacombe, Thomas R. Sibley, Melissa Truong, Caitlin R. Wolf, Michael Boeckh, Janet A. Englund, Michael Famulare, Barry R. Lutz, Mark J. Rieder, Lea M. Starita, Matthew Thompson, Jay Shendure, Trevor Bedford, Helen Y. Chu
EPI_ISL_903633	WA State Department of Health	Genomics and Discovery, Respiratory Viruses Branch, Division of Viral Diseases, Centers for Disease Control and Prevention	Krista Queen, Yan Li, Ying Tao, Jing Zhang, Anna Uehara, Anna Montmayeur, Clinton R. Paden, Peter W. Cook, Rachel Marine, Mili Sheth, Jasmine Padilla, Sarah Nobles, Mark Burroughs, Lori Rowe, Haibin Wang, Ben L. Rambo-Martin, Dhwani Batra, Justin Lee, Suxiang Tong
EPI_ISL_903748, EPI_ISL_903785, EPI_ISL_903820, EPI_ISL_903854	PA Department of Health, Bureau of Laboratories	Genomics and Discovery, Respiratory Viruses Branch, Division of Viral Diseases, Centers for Disease Control and Prevention	Krista Queen, Yan Li, Ying Tao, Jing Zhang, Anna Uehara, Anna Montmayeur, Clinton R. Paden, Peter W. Cook, Rachel Marine, Mili Sheth, Jasmine Padilla, Sarah Nobles, Mark Burroughs, Lori Rowe, Haibin Wang, Ben L. Rambo-Martin, Dhwani Batra, Justin Lee, Suxiang Tong
EPI_ISL_903949, EPI_ISL_903952	WA State Department of Health	Genomics and Discovery, Respiratory Viruses Branch, Division of Viral Diseases, Centers for Disease Control and Prevention	Krista Queen, Yan Li, Ying Tao, Jing Zhang, Anna Uehara, Anna Montmayeur, Clinton R. Paden, Peter W. Cook, Rachel Marine, Mili Sheth, Jasmine Padilla, Sarah Nobles, Mark Burroughs, Lori Rowe, Haibin Wang, Ben L. Rambo-Martin, Dhwani Batra, Justin Lee, Suxiang Tong
EPI_ISL_904735	Dutch COVID-19 response team	National Institute for Public Health and the Environment (RIVM)	Adam Meijer, Harry Vennema, Dirk Eggink, Jeroen Cremer, Sharon van den Brink, Bas van der Veer, AnneMarie van den Brandt, Florian Zwagemaker, Dennis Schmitz, Chantal Reusken, on behalf of the national COVID-19 response team
EPI_ISL_904947, EPI_ISL_904948	Vilnius University Hospital Santaros Klinikos, Vilnius University	Institute of Biotechnology, Life Sciences Center, Vilnius University	Emilija Vasiliunaite, Milda Norkiene, Albertas Timinskas, Alma Gedvilaite, Aurelija Zvirbliene, Daniel Naumovas, Laimonas Griskevicius
EPI_ISL_906105	Child Health Research Foundation	Child Health Research Foundation	Senjuti Saha, Sharmistha Goswami, Afroza Akter Tanni, Syed Muktadir Al Sium, Arif Mohammad Tanmoy, Roly Malaker, Md Hafizur Rahman, Samir K Saha
EPI_ISL_906130	Institute of Microbiology and Immunology, Faculty of Medicine, University of Ljubljana	Institute of Microbiology and Immunology, Faculty of Medicine, University of Ljubljana	Samo Zakotnik, Tomaž Mark Zorec, Matic Brvar, Miša Korva, Mario Poljak, Tatjana Avši - Županc
EPI_ISL_906263	University of Wisconsin-Madison AIDS Vaccine Research Laboratories	University of Wisconsin-Madison AIDS Vaccine Research Laboratories	Gage Moreno, Katarina Braun, et al. AIDS Vaccine Research Laboratories
EPI_ISL_906852	Respiratory Viruses Branch, Centers for Disease Control and Prevention	Respiratory Viruses Branch, Centers for Disease Control and Prevention	Tao,Y., Li,Y., Zhang,J., Queen,K., Uehara,A., Cook,P., Paden,C.R., Wang,H., Tong,S.
EPI_ISL_907077, EPI_ISL_907078, EPI_ISL_907079, EPI_ISL_907080, EPI_ISL_907081, EPI_ISL_907082, EPI_ISL_907083, EPI_ISL_907084, EPI_ISL_907085	Biology, MCL	Biology, MCL	Seadawy,M.G., Shamel,M.D., Gad,A.F., Elhoseiny,M.F., Zekri,A.N.
EPI_ISL_911239, EPI_ISL_911240	Laboratoire national de sante, Microbiology, Virology	Laboratoire national de sante, Microbiology, Microbial Genomics Platform	Anke Wienecke-Baldacchino, Catherine Ragimbeau,Jessica Tapp, Fatu Djabi, Lise Pignon, Raoul Salmon, Tamir Abdelrahman
EPI_ISL_911687	Alaska State Virology Laboratory	Alaska State Virology Laboratory	Stephanie DeRonde, Lisa Smith, Ph.D., Jack Chen, Ph.D.
EPI_ISL_912466, EPI_ISL_912472, EPI_ISL_912475, EPI_ISL_912479, EPI_ISL_912481, EPI_ISL_912487, EPI_ISL_912536	NHLS Universitas Academic	UFS Virology	PA Bester, MM Nyaga, P Nthiga, MT Mogotsi, D Goedhals, T de Oliveira
EPI_ISL_912925	Hôpital Pitié-Salpêtrière	Department of Virology, Henri Mondor University Hospital, Assistance Publique Hôpitaux de Paris, Université Paris-Est Créteil, INSERM U955	Christophe Rodriguez, Slim Fourati, Vanessa Demontant, Guillaume Gricourt, Melissa N'Debi, Alexandre Soulier, Elisabeth Trawinski, Jean-Michel Pawlotsky

EPI_ISL_913088	Center for Virology	Center for Virology	Jeremy V. Camp, Irene Goerzer, Monika Redlberger-Fritz, Stephan W. Aberle
EPI_ISL_913117, EPI_ISL_913122, EPI_ISL_913123, EPI_ISL_913124, EPI_ISL_913132, EPI_ISL_913140, EPI_ISL_913145, EPI_ISL_913147, EPI_ISL_913148, EPI_ISL_913151, EPI_ISL_913152, EPI_ISL_913153, EPI_ISL_913154, EPI_ISL_913157, EPI_ISL_913160, EPI_ISL_913201, EPI_ISL_913202, EPI_ISL_913203, EPI_ISL_913204, EPI_ISL_913205, EPI_ISL_913206, EPI_ISL_913207, EPI_ISL_913208, EPI_ISL_913209, EPI_ISL_913210, EPI_ISL_913211, EPI_ISL_913212, EPI_ISL_913213, EPI_ISL_913214, EPI_ISL_913215, EPI_ISL_913216, EPI_ISL_913217, EPI_ISL_913218, EPI_ISL_913219, EPI_ISL_913220, EPI_ISL_913221, EPI_ISL_913222, EPI_ISL_913223, EPI_ISL_913224, EPI_ISL_913225, EPI_ISL_913226, EPI_ISL_913227, EPI_ISL_913228, EPI_ISL_913229, EPI_ISL_913230, EPI_ISL_913231, EPI_ISL_913232, EPI_ISL_913233, EPI_ISL_913234, EPI_ISL_913235, EPI_ISL_913236, EPI_ISL_913237, EPI_ISL_913238, EPI_ISL_913239, EPI_ISL_913240, EPI_ISL_913241, EPI_ISL_913242, EPI_ISL_913243, EPI_ISL_913244, EPI_ISL_913245, EPI_ISL_913246, EPI_ISL_913247, EPI_ISL_913248, EPI_ISL_913249, EPI_ISL_913250, EPI_ISL_913251, EPI_ISL_913252, EPI_ISL_913253, EPI_ISL_913254, EPI_ISL_913255			
see above	University of Michigan Clinical Microbiology Laboratory	Lauring Lab, University of Michigan, Department of Microbiology and Immunology	Valesano
EPI_ISL_913480	Klinisk mikrobiologi	The Public Health Agency of Sweden	Anna-Malin Linde, Maria Lind Karlberg, Carlo Berg, Oskar Karlsson Lindsjo, Sofia Stamouli, Reza Advani, Mattias Haukland, Petra Holmstrom, Noura Walai, Petra Edquist, Mia Brytting, Anna Risberg, Karin Tegmark-Wisell
EPI_ISL_913517, EPI_ISL_913518	University of Michigan Clinical Microbiology Laboratory	Lauring Lab, University of Michigan, Department of Microbiology and Immunology	Valesano
EPI_ISL_913933, EPI_ISL_913958	Instituto de Diagnostico y Referencia Epidemiologicos INDRE_RNLSP	Instituto de Diagnostico y Referencia Epidemiologicos (INDRE)	Claudia Wong-Arambula, Abril Rodriguez-Maldonado, Fabiola Garces-Ayala, Adnan Araiza-Rodriguez, David Fragoso-Fonseca, Sergio Rangel-Guerrero, Mayra Jimenez-Morales, Nancy Munoz-Hernandez, Natividad Cruz-Ortiz, Tatiana Nunez-Garcia, Gisela Barrera-Badillo, Lucia Hernandez-Rivas, Irma Lopez-Martinez, Ernesto Ramirez-Gonzalez.
EPI_ISL_915365, EPI_ISL_915366, EPI_ISL_915371, EPI_ISL_915378, EPI_ISL_915412	Keio University School of Medicine	Keio University School of Medicine	Kenjiro Kosaki, Yuka Iwasaki, Hirotsugu Ishizu, Haruhiko Siomi, Kodai Abe
EPI_ISL_915656, EPI_ISL_916068, EPI_ISL_916071, EPI_ISL_916075, EPI_ISL_916076, EPI_ISL_916079, EPI_ISL_916080, EPI_ISL_916082, EPI_ISL_916086, EPI_ISL_916087, EPI_ISL_916088, EPI_ISL_916091, EPI_ISL_916092, EPI_ISL_916094, EPI_ISL_916098, EPI_ISL_916099, EPI_ISL_916100, EPI_ISL_916105, EPI_ISL_916106, EPI_ISL_916107, EPI_ISL_916108, EPI_ISL_916109, EPI_ISL_916110, EPI_ISL_916111, EPI_ISL_916113, EPI_ISL_916121, EPI_ISL_916124, EPI_ISL_916127, EPI_ISL_916129, EPI_ISL_916131, EPI_ISL_916132, EPI_ISL_916134, EPI_ISL_916137, EPI_ISL_916144, EPI_ISL_916145, EPI_ISL_916147, EPI_ISL_916151, EPI_ISL_916152, EPI_ISL_916154, EPI_ISL_916159, EPI_ISL_916161, EPI_ISL_916162, EPI_ISL_916164, EPI_ISL_916165, EPI_ISL_916166, EPI_ISL_916167, EPI_ISL_916169, EPI_ISL_916171, EPI_ISL_916174			
see above	Lighthouse Lab in Alderley Park	Wellcome Sanger Institute for the COVID-19 Genomics UK (COG-UK) Consortium	Jacquelyn Wynn, Mairead Hyland, The Lighthouse Lab in Alderley Park and Alex Alderton, Roberto Amato, Sonia Goncalves, Ewan Harrison, David K. Jackson, Ian Johnston, Dominic Kwiatkowski, Cordelia Langford, John Sillitoe on behalf of the Wellcome Sanger Institute COVID-19 Surveillance Team
EPI_ISL_918184, EPI_ISL_918202, EPI_ISL_918205, EPI_ISL_918206, EPI_ISL_918207, EPI_ISL_918209, EPI_ISL_918211, EPI_ISL_918214, EPI_ISL_918216	Innovative Genomics Institute, UC Berkeley	Innovative Genomics Institute, UC Berkeley	Stacia Wyman, Haridha Shivram, Phil Frankino, Liana Lareau, Shana McDevitt, Justin Choi
EPI_ISL_918515	LACEN - Laboratório Central de Saúde Pública do Para	Evandro Chagas Institute	Santos, M.C.; Silva, A.M.; Junior, W.D.C.; Barbagelata, L.S.; Ferreira, J.A.; Sousa, E.M.A.; da Silva, P.S.; Pinheiro, K.C.; L.C.; Sousa Junior, E.C.
EPI_ISL_918564, EPI_ISL_918565, EPI_ISL_918566, EPI_ISL_918567, EPI_ISL_918568, EPI_ISL_918585, EPI_ISL_918586, EPI_ISL_918587, EPI_ISL_918588, EPI_ISL_918590, EPI_ISL_918665			
see above	University of Birmingham	COVID-19 Genomics UK (COG-UK) Consortium	Institute of Microbiology, University of Birmingham: Claire McMurray, Joanne Stockton, Samuel Nicholls, Radoslaw Poplawski, Will Rowe, Josh Quick, Nicholas Loman. University of Birmingham Testing Laboratory: Celina M Whalley, Andrew Bosworth, Charlotte Poxon, Kasun Wanigasooriya, Oliver Pickles, Mike Kidd, Alex Richter, Andrew D Beggs PHE Heartlands Lab: Husam Osman, Andrew Bosworth. Queen Elizabeth Hospital: Anna Casey
EPI_ISL_920268, EPI_ISL_920278, EPI_ISL_920355, EPI_ISL_920364, EPI_ISL_920376, EPI_ISL_920517	University College London Hospital	COVID-19 Genomics UK (COG-UK) Consortium	Judith Heaney, Matthew Byott, Catherine Houlihan, Dan Frampton, Stuart Kirk, Moira Spyer and Eleni Nastouli
EPI_ISL_925181, EPI_ISL_925182, EPI_ISL_925183, EPI_ISL_925184, EPI_ISL_925185, EPI_ISL_925186, EPI_ISL_925187, EPI_ISL_925188, EPI_ISL_925189, EPI_ISL_925190, EPI_ISL_925191, EPI_ISL_925192, EPI_ISL_925193, EPI_ISL_925194, EPI_ISL_925195, EPI_ISL_925196, EPI_ISL_925197, EPI_ISL_925201, EPI_ISL_925202, EPI_ISL_925203			
see above	Virginia DCLS	Virginia DCLS	Virginia DCLS
EPI_ISL_925376, EPI_ISL_925377, EPI_ISL_925378, EPI_ISL_925466, EPI_ISL_925495	Department of Clinical Microbiology	GIGA Medical Genomics	Keith Durkin, Maria Artesi, Sébastien Bontems, Raphaël Boreux, Bouchra Boujemla, Cécile Meex, Pierrette Melin, Marie-Pierre Hayette, Vincent Bours
EPI_ISL_925992, EPI_ISL_926388, EPI_ISL_926432, EPI_ISL_926494, EPI_ISL_926504, EPI_ISL_926768, EPI_ISL_926787, EPI_ISL_926811, EPI_ISL_927079, EPI_ISL_927118, EPI_ISL_927211, EPI_ISL_927252, EPI_ISL_927295, EPI_ISL_927345, EPI_ISL_927431, EPI_ISL_927603, EPI_ISL_928118, EPI_ISL_928179, EPI_ISL_928282, EPI_ISL_928285, EPI_ISL_928297, EPI_ISL_928380, EPI_ISL_928422, EPI_ISL_928513, EPI_ISL_928606, EPI_ISL_928628, EPI_ISL_928630, EPI_ISL_928655, EPI_ISL_928670, EPI_ISL_928722, EPI_ISL_928884, EPI_ISL_928954, EPI_ISL_929022, EPI_ISL_929213, EPI_ISL_929286, EPI_ISL_929299, EPI_ISL_929391, EPI_ISL_929450, EPI_ISL_929476, EPI_ISL_929657, EPI_ISL_929678, EPI_ISL_929797, EPI_ISL_929900, EPI_ISL_929916, EPI_ISL_930088, EPI_ISL_930112, EPI_ISL_930187, EPI_ISL_930202, EPI_ISL_930220, EPI_ISL_930376, EPI_ISL_930403, EPI_ISL_930562			
see above	Department of Virus and Microbiological Special Diagnostics, Statens Serum Institut, Copenhagen, Denmark	Aalborg University	Danish Covid-19 Genome Consortium
EPI_ISL_931343, EPI_ISL_931344, EPI_ISL_931345, EPI_ISL_931346, EPI_ISL_931347, EPI_ISL_931348, EPI_ISL_931349, EPI_ISL_931350, EPI_ISL_931351, EPI_ISL_931352, EPI_ISL_931353, EPI_ISL_931354, EPI_ISL_931355, EPI_ISL_931356, EPI_ISL_931357, EPI_ISL_931358, EPI_ISL_931359, EPI_ISL_931360, EPI_ISL_931361, EPI_ISL_931362, EPI_ISL_931370, EPI_ISL_931371, EPI_ISL_931372, EPI_ISL_931373, EPI_ISL_931374, EPI_ISL_931375			
see above	University Hospital Basel, Clinical Virology	University Hospital Basel, Clinical Bacteriology	Tim Roloff, Madlen Stange, Helena MB Seth-Smith, Alfredo Mari, Karoline Leuzinger, Julia Bielicki, Manuel Battegay, Hans Hirsch, Adrian Egli
EPI_ISL_933495	Lighthouse Lab in Milton Keynes	Wellcome Sanger Institute for the COVID-19 Genomics UK (COG-UK) Consortium	The Lighthouse Lab in Milton Keynes and Alex Alderton, Roberto Amato, Sonia Goncalves, Ewan Harrison, David K. Jackson, Ian Johnston, Dominic Kwiatkowski, Cordelia Langford, John Sillitoe on behalf of the Wellcome Sanger Institute COVID-19 Surveillance Team
EPI_ISL_933540, EPI_ISL_933542, EPI_ISL_933543, EPI_ISL_933544, EPI_ISL_933545, EPI_ISL_933546	Arizona State Public Health Laboratory	Arizona State Public Health Laboratory	Trung Huynh, Jessica Escobar, Katherine Fullerton, Nobuko Fukushima, Stacy White, Linda Getsinger, Victor Waddell
EPI_ISL_933707	Instituto de Diagnostico y Referencia Epidemiologicos INDRE_RNLSP	Instituto de Diagnostico y Referencia Epidemiologicos (INDRE)	Claudia Wong-Arambula, Abril Rodriguez-Maldonado, Fabiola Garces-Ayala, Adnan Araiza-Rodriguez, David Fragoso-Fonseca, Sergio Rangel-Guerrero, Mayra Jimenez-Morales, Nancy Munoz-Hernandez, Natividad Cruz-Ortiz, Tatiana Nunez-Garcia, Gisela Barrera-Badillo, Lucia Hernandez-Rivas, Irma Lopez-Martinez, Ernesto Ramirez-Gonzalez.
EPI_ISL_934542, EPI_ISL_934544	Institut für Virologie am Department für Hygiene, Mikrobiologie und Public Health	Bergthaler laboratory, CeMM Research Center for Molecular Medicine of the Austrian Academy of Sciences	Lukas Endler, Anna Schedl, Thomas Penz, Benedikt Agerer, Maelle Le Moing, Michael Schuster, Bekir Erguner, Jan Laine, Martin Senekowitsch, Christoph Bock, Andreas Bergthaler
EPI_ISL_935107, EPI_ISL_935108, EPI_ISL_935119, EPI_ISL_935120, EPI_ISL_935121, EPI_ISL_935125, EPI_ISL_935128	1.AO Universitaria 'S. Giovanni di Dio e Ruggi D'Aragona, Scuola Medica Salernitana' Hospital / 2.UOC di Virologia e Microbiologia, Università della Campania 'L. Vanvitelli' / 3.AO Universitaria 'Federico II' Napoli Hospital / 4.AORN 'San Giuseppe Moscati' Avellino Hospital / 5.AO 'San Pio - presidio G. Rummo' Benevento Hospital / 6.AO 'Sant'Anna e San Sebastiano' Caserta Hospital / 7.PO 'Maria Santissima Addolorata' Eboli Hospital / 8.Biogem Istituto di Ricerche Genetiche	1. Genome Research Center for Health (CRGS) / 2. Laboratory of Molecular Medicine and Genomics(LMMGe) / 3. Center for Research in Pure and Applied Mathematics (CRMPA)	Giorgio Giurato (Corresponding Author), Francesca Rizzo (Corresponding Author), Alessandro Weisz (Corresponding Author), Gianluigi Franci, Giovanni Nassa, Pasquale Pagliano, Roberta Tarallo, Elena Alexandrova, Ylenia D'Agostino, Carlo Ferravante, Jessica Lamberti, Viola Melone, Domenico Memoli, Valeria Mirici Cappa, Domenico Palumbo, Giovanni Pecoraro, Assunta Sellitto, Oriana Strianese, Ilaria Terenzi, Giuseppe Fenza, Aniello Gentile, Antonello Saccomanno, Sonia Amabile, Teresa Rocco, Annamaria Salvati, Emilia Vaccaro, Massimiliano Galdiero, Michele Cennamo, Giuseppe Portella, Maria Grazia Foti, Mariarosaria Ingino, Maria Landi, Maurizio Fumi, Vincenzo Rocco, Rita Greco, Vittoria Letizia, Arnolfo Petruzziello, Maddalena Schioppa, Gregorio Goffredi, Francesca Marciano, Michele Caraglia, Alessia Cossu, Marianna Scrima
EPI_ISL_935415, EPI_ISL_935416, EPI_ISL_935417, EPI_ISL_935418, EPI_ISL_935419, EPI_ISL_935420, EPI_ISL_935421, EPI_ISL_935422, EPI_ISL_935423, EPI_ISL_935424, EPI_ISL_935425, EPI_ISL_935426, EPI_ISL_935427, EPI_ISL_935428, EPI_ISL_935429, EPI_ISL_935430, EPI_ISL_935431, EPI_ISL_935432, EPI_ISL_935433, EPI_ISL_935434, EPI_ISL_935435, EPI_ISL_935436, EPI_ISL_935437, EPI_ISL_935438, EPI_ISL_935439, EPI_ISL_935440, EPI_ISL_935441, EPI_ISL_935442, EPI_ISL_935443, EPI_ISL_935444, EPI_ISL_935445, EPI_ISL_935446, EPI_ISL_935447, EPI_ISL_935448, EPI_ISL_935449, EPI_ISL_935450, EPI_ISL_935451, EPI_ISL_935452, EPI_ISL_935453, EPI_ISL_935454, EPI_ISL_935455, EPI_ISL_935456, EPI_ISL_935457, EPI_ISL_935458, EPI_ISL_935459, EPI_ISL_935460, EPI_ISL_935461, EPI_ISL_935462, EPI_ISL_935463			
see above	Florida Bureau of Public Health Laboratories	Florida Bureau of Public Health Laboratories	Sarah Schmedes, Jason Blanton
EPI_ISL_935881, EPI_ISL_935900, EPI_ISL_935904, EPI_ISL_935908, EPI_ISL_935919, EPI_ISL_935920, EPI_ISL_935922, EPI_ISL_935925, EPI_ISL_935926, EPI_ISL_935927, EPI_ISL_935928, EPI_ISL_935929, EPI_ISL_935931, EPI_ISL_935932, EPI_ISL_935934, EPI_ISL_935935, EPI_ISL_935936, EPI_ISL_935937,			

EPI_ISL_935938, EPI_ISL_935939, EPI_ISL_935940, EPI_ISL_935941, EPI_ISL_935942, EPI_ISL_935943, EPI_ISL_935944, EPI_ISL_935945, EPI_ISL_935946, EPI_ISL_935947, EPI_ISL_935948, EPI_ISL_935949, EPI_ISL_935950, EPI_ISL_935951, EPI_ISL_935952			
see above	Cadham Provincial laboratory	National Microbiology Laboratory (NML)	Anna Majer, Shari Tyson, Grace Seo, Philip Mabon, Elsie Grudeski, Rhiannon Huzarewich, Russell Mandes, Anneliese Landgraff, Jennifer Tanner, Natalie Knox, Morag Graham, Gary Van Domselaar, Paul Van Caeseele, Jared Bullard, David Alexander, Kerry Dust, Nathalie Bastien, Yan Li, Timothy Booth, Darian Hole, Madison Chapel, Kirsten Biggar, CanCOGeN's metadata curation team, Public Health Agency of Canada CanCOGeN team
EPI_ISL_936613, EPI_ISL_936615, EPI_ISL_936616, EPI_ISL_936617, EPI_ISL_936618	Northwestern Memorial Hospital	Ozer Lab	Ramon Lorenzo-Redondo, Lacy M. Simons, Chad J. Achenbach, Lawrence J. Jennings, Michael G. Ison, Judd F. Hultquist, Egon A. Ozer
EPI_ISL_940427, EPI_ISL_940451	Hôpital Bichat Claude Bernard, Laboratoire de Virologie	IAME UMR1137 Inserm, Université de Paris, Hôpital Bichat	Antoine Bridier-Nahmias, Amélie Recoing, Quentin Le Hingrat, Lena Daniel, Siham Hamri, Gilles Collin, Alexandre Storto, Mélanie Bertine, Charlotte Charpentier, Nadhira Houhou-Fidouh, Diane Descamps, Benoit Visseaux
EPI_ISL_940855	Vaccines and Infectious Diseases Analytics Research Unit (VIDA)	KRISP, KZN Research Innovation and Sequencing Platform	Baillie Vicky, du Plessis Jeanine, Giandhari Jennifer, Pillay Sureshnee, Naidoo Yeshnee, Tegally Houriiyah, de Oliveira Tulio, Madhi Shabir
EPI_ISL_941206	Servicio de Microbiología. Hospital Clínico Universitario de Valencia	SeqCOVID-SPAIN consortium/IBV(CSIC)	David Navarro Ortega, Eliseo Albert Vicent, Ignacio Torres and SeqCOVID-SPAIN consortium
EPI_ISL_941936, EPI_ISL_941937	Florida Bureau of Public Health Laboratories	Florida Bureau of Public Health Laboratories	Sarah Schmedes, Jason Blanton
EPI_ISL_942454, EPI_ISL_942455, EPI_ISL_942456, EPI_ISL_942457, EPI_ISL_942459, EPI_ISL_942460, EPI_ISL_942461, EPI_ISL_942462, EPI_ISL_942463, EPI_ISL_942464, EPI_ISL_942465, EPI_ISL_942466, EPI_ISL_942467, EPI_ISL_942468, EPI_ISL_942469, EPI_ISL_942470, EPI_ISL_942471, EPI_ISL_942472, EPI_ISL_942473, EPI_ISL_942474, EPI_ISL_942475, EPI_ISL_942476, EPI_ISL_942477, EPI_ISL_942478, EPI_ISL_942479			
see above	Gundersen Molecular Diagnostics Laboratory	Kabara Cancer Research Institute	Craig S. Richmond, Paraic A. Kenny
EPI_ISL_943602, EPI_ISL_943603, EPI_ISL_943605, EPI_ISL_943606, EPI_ISL_943608, EPI_ISL_943610, EPI_ISL_943612	Lacen_RS	State Center for Health Surveillance. Rio Grande do Sul State Secretary of Health	Aline Campos, Amanda da Silva, Anelise Schaurich, Claudia Dornelles, Cynthia Molina, Fernanda Godinho, Lara Crescente, Leticia Garay, Regina Barcellos, Richard Salvato, Tatiana Gregianini, Vagner Fonseca
EPI_ISL_947265	RS Sari Asih Ciledug	Eijkman Institute for Molecular Biology, Ministry of Research and Technology/National Agency for Research and Innovation	Frilasita A Yudhaputri, Hidayat Trimarsanto, Iskandar Adnan, Lydia V. Panggalo, Sukma Oktavianthi, Willy Agustine, Edison Johar, Safarina G Malik, Khin Saw Myint, Amin Soebandrio
EPI_ISL_947268	RSU Anggrek Mas	Eijkman Institute for Molecular Biology, Ministry of Research and Technology/National Agency for Research and Innovation	Frilasita A Yudhaputri, Hidayat Trimarsanto, Iskandar Adnan, Lydia V. Panggalo, Sukma Oktavianthi, Willy Agustine, Edison Johar, Safarina G Malik, Khin Saw Myint, Amin Soebandrio
EPI_ISL_947270	RS Mitra Keluarga Gading Serpong	Eijkman Institute for Molecular Biology, Ministry of Research and Technology/National Agency for Research and Innovation	Frilasita A Yudhaputri, Hidayat Trimarsanto, Iskandar Adnan, Lydia V. Panggalo, Sukma Oktavianthi, Willy Agustine, Edison Johar, Safarina G Malik, Khin Saw Myint, Amin Soebandrio
EPI_ISL_949347, EPI_ISL_949349, EPI_ISL_949350, EPI_ISL_949351, EPI_ISL_949352, EPI_ISL_949353, EPI_ISL_949354, EPI_ISL_949355, EPI_ISL_949356, EPI_ISL_949358, EPI_ISL_949359, EPI_ISL_949360, EPI_ISL_949361, EPI_ISL_949362, EPI_ISL_949363, EPI_ISL_949364, EPI_ISL_949365, EPI_ISL_949366, EPI_ISL_949367, EPI_ISL_949368, EPI_ISL_949369, EPI_ISL_949370, EPI_ISL_949371, EPI_ISL_949372, EPI_ISL_949373, EPI_ISL_949374, EPI_ISL_949375, EPI_ISL_949376, EPI_ISL_949377, EPI_ISL_949378, EPI_ISL_949379, EPI_ISL_949380, EPI_ISL_949381, EPI_ISL_949382, EPI_ISL_949383, EPI_ISL_949384, EPI_ISL_949385, EPI_ISL_949386, EPI_ISL_949387, EPI_ISL_949388, EPI_ISL_949389, EPI_ISL_949390			
see above	University of Birmingham	COVID-19 Genomics UK (COG-UK) Consortium	Institute of Microbiology, University of Birmingham: Claire McMurray, Joanne Stockton, Samuel Nicholls, Radoslaw Poplawski, Will Rowe, Josh Quick, Nicholas Loman. University of Birmingham Testing Laboratory: Celina M Whalley, Andrew Bosworth, Charlotte Poxon, Kasun Wanigasooriya, Oliver Pickles, Mike Kidd, Alex Richter, Andrew D Beggs PHE Heartlands Lab: Husam Osman, Andrew Bosworth. Queen Elizabeth Hospital: Anna Casey
EPI_ISL_952431	Centre for Enzyme Innovation, University of Portsmouth / Translational Research Laboratory, Portsmouth Hospitals NHS Trust	COVID-19 Genomics UK (COG-UK) Consortium	Angela Beckett,Salman Goudarzi,Christopher Fearn,Kate Cook,Katie Loveson,Sharon Glaysher,Scott Elliott,Samuel Robson
EPI_ISL_955165, EPI_ISL_955166	Institute for Biocides and Medical Ecology	Institute of microbiology and Immunology, Faculty of Medicine, University of Belgrade	Knezevic,A., Jankovic,M., Vidanovic,D., Milicevic,O., Tesovic,B., Sekler,M., Jovanovic,T.
EPI_ISL_955189, EPI_ISL_955190, EPI_ISL_955191, EPI_ISL_955194, EPI_ISL_955195, EPI_ISL_955197, EPI_ISL_955198, EPI_ISL_955199, EPI_ISL_955200, EPI_ISL_955201, EPI_ISL_955202, EPI_ISL_955203, EPI_ISL_955204, EPI_ISL_955205, EPI_ISL_955206, EPI_ISL_955207, EPI_ISL_955208, EPI_ISL_955209			
see above	Instituto Nacional de Medicina Genomica	Instituto Nacional de Medicina Genomica	Hidalgo-Miranda A, Mendoza-Vargas A, Reyes-Grajeda JP, Cisneros-Villanueva M, Cedro-Tanda A,Peñaloza-Figueroa F, Herrera-Montalvo LA
EPI_ISL_955939, EPI_ISL_955946, EPI_ISL_955947, EPI_ISL_955948, EPI_ISL_955950, EPI_ISL_955954, EPI_ISL_955955, EPI_ISL_955960, EPI_ISL_955961, EPI_ISL_955962, EPI_ISL_955963, EPI_ISL_955964, EPI_ISL_955965, EPI_ISL_955966, EPI_ISL_955967, EPI_ISL_955968, EPI_ISL_955969, EPI_ISL_955970, EPI_ISL_955971, EPI_ISL_955976, EPI_ISL_955977, EPI_ISL_955978			
see above	Division of Emerging Infectious Diseases, Bureau of Infectious Diseases Diagnosis Control, Korea Disease Control and Prevention Agency	Division of Emerging Infectious Diseases, Bureau of Infectious Diseases Diagnosis Control, Korea Disease Control and Prevention Agency	Ae Kyung Park, Il-Hwan Kim, Heui Man Kim, Jeong-Min Kim, Namjoo Lee, Chae Young Lee, Sang Hee Woo, Eun-Jin Kim
EPI_ISL_959302, EPI_ISL_959303	Centre de Recerca en Salut Animal (IRTA/CRESA)	IrsiCaixa - Can Ruti CovidSeq	Marc Noguera-Julian, Mariona Parera, Maria Casadellà, Pilar Armengol, Francesc Catala-Moll, Roger Paredes, Bonaventura Clotet J. Segalés, M. Puig, J. Rodon, C. Avila-Nieto, J. Carrillo, G. Cantero, M.T. Terrón, S. Cruz, N. Izquierdo-Useros, E. Vidal, J. Blanco, B. Clotet, J. Vergara-Alert
EPI_ISL_959479, EPI_ISL_959480, EPI_ISL_959481, EPI_ISL_959482, EPI_ISL_959501, EPI_ISL_959506	Division of Emerging Infectious Diseases, Bureau of Infectious Diseases Diagnosis Control, Korea Disease Control and Prevention Agency	Division of Emerging Infectious Diseases, Bureau of Infectious Diseases Diagnosis Control, Korea Disease Control and Prevention Agency	Ae Kyung Park, Il-Hwan Kim, Heui Man Kim, Jeong-Min Kim, Namjoo Lee, Chae Young Lee, Sang Hee Woo, Eun-Jin Kim
EPI_ISL_960343, EPI_ISL_960352, EPI_ISL_960375, EPI_ISL_960381, EPI_ISL_960386	University of Wisconsin-Madison AIDS Vaccine Research Laboratories	University of Wisconsin-Madison AIDS Vaccine Research Laboratories	Gage Moreno, Katarina Braun, et al. AIDS Vaccine Research Laboratories
EPI_ISL_960445, EPI_ISL_960450, EPI_ISL_960483, EPI_ISL_960484, EPI_ISL_960485, EPI_ISL_960502, EPI_ISL_960557, EPI_ISL_960568, EPI_ISL_960569, EPI_ISL_960578, EPI_ISL_960579, EPI_ISL_960580, EPI_ISL_960646, EPI_ISL_960660, EPI_ISL_960661			
see above	Istituto Zooprofilattico Sperimentale del Mezzogiorno	TIGEM	Patrizia Annunziata, Andrea Ballabio, Valentina Bouche, Davide Cacchiarelli, Pellegrino Cerino, Chiara Colantuono, Maria Concetta Cuomo, Denise Di Concilio, Lucio Di Filippo, Antonio Grimaldi, Antonio Limone, Anna Manfredi, Francesco Panariello, Biancamaria Pierri, Marcello Salvi
EPI_ISL_961667	Hôpital Georges L. Dumont	National Microbiology Laboratory (NML)	Anna Majer, Shari Tyson, Grace Seo, Philip Mabon, Elsie Grudeski, Rhiannon Huzarewich, Russell Mandes, Anneliese Landgraff, Jennifer Tanner, Natalie Knox, Morag Graham, Gary Van Domselaar, Richard Garceau, Guillaume Desnoyers, Nathalie Bastien, Yan Li, Timothy Booth, Darian Hole, Madison Chapel, Kirsten Biggar, CanCOGeN's metadata curation team, Public Health Agency of Canada CanCOGeN team
EPI_ISL_962208, EPI_ISL_962209, EPI_ISL_962210, EPI_ISL_962211, EPI_ISL_962212, EPI_ISL_962213, EPI_ISL_962214, EPI_ISL_962215, EPI_ISL_962216, EPI_ISL_962217, EPI_ISL_962218, EPI_ISL_962219, EPI_ISL_962220, EPI_ISL_962221, EPI_ISL_962222, EPI_ISL_962223			
see above	Seattle Flu Study	Seattle Flu Study	Deborah A. Nickerson, Chris D. Frazar, Jover Lee, Benjamin Pelle, Erica Ryke, Matthew Richardson, Amanda Adler, Elisabeth Brandstetter, Peter D. Han, Kairsten Fay, Misja Ilcisin, Kirsten Lacombe, Thomas R. Sibley, Melissa Truong, Caitlin R. Wolf, Karen Cowgill, Stephanie Schrag, Jeff Duchin, Michael Boeckh, Janet A. Englund, Michael Famulare, Barry R. Lutz, Mark J. Rieder, Lea M. Starita, Matthew Thompson, Helen Y. Chu, Trevor Bedford, Jay Shendure
EPI_ISL_962771, EPI_ISL_962775, EPI_ISL_962781, EPI_ISL_962782, EPI_ISL_962789, EPI_ISL_962794, EPI_ISL_962798, EPI_ISL_962804	San Diego County Public Health Laboratory	Andersen lab at Scripps Research	SEARCH Alliance San Diego with Tracy Basler, Jovan Shephard, Brett Austin
EPI_ISL_965341, EPI_ISL_965344, EPI_ISL_965347, EPI_ISL_965348, EPI_ISL_965349, EPI_ISL_965353, EPI_ISL_965354, EPI_ISL_965360, EPI_ISL_965363, EPI_ISL_965366, EPI_ISL_965367, EPI_ISL_965368, EPI_ISL_965369, EPI_ISL_965370, EPI_ISL_965372, EPI_ISL_965373, EPI_ISL_965374, EPI_ISL_965385, EPI_ISL_965391, EPI_ISL_965461, EPI_ISL_965464			
see above	University of Liège COVID-19 testing center	GIGA Medical Genomics	Keith Durkin, Maria Artesi, Bouchra Boujemla, Emmanuel André, Marc Van Ranst, Fabrice Bureau, Laurent Gillet, Wouter Coppieters, Vincent Bours
EPI_ISL_965532, EPI_ISL_965543,	Dutch COVID-19 response team	Medical Microbiology, Maastricht University Medical Centre	Jozef Dingemans*, Brian van der Veer*, Erik Beuken, Carmen Reumkens, Lieke van Alphen, Christian Hoebe, Paul Savelkoul

EPI_ISL_965663			
EPI_ISL_968199, EPI_ISL_968200, EPI_ISL_968201, EPI_ISL_968202, EPI_ISL_968203, EPI_ISL_968204	Arizona State Public Health Laboratory	Arizona State Public Health Laboratory	Trung Huynh, Jessica Escobar, Katherine Fullerton, Nobuko Fukushima, Stacy White, Linda Getsinger, Victor Waddell
EPI_ISL_968808, EPI_ISL_968810, EPI_ISL_968925, EPI_ISL_968926, EPI_ISL_968927, EPI_ISL_968993	KEMRI-Wellcome Trust Research Programme/KEMRI-CGMR-C Kilifi	KEMRI-Wellcome Trust Research Programme/KEMRI-CGMR-C Kilifi	Githinji et al
EPI_ISL_975495, EPI_ISL_975496, EPI_ISL_975497, EPI_ISL_975498, EPI_ISL_975499, EPI_ISL_975500, EPI_ISL_975501, EPI_ISL_975502, EPI_ISL_975503, EPI_ISL_975504, EPI_ISL_975505, EPI_ISL_975506, EPI_ISL_975507, EPI_ISL_975508, EPI_ISL_975509, EPI_ISL_975510, EPI_ISL_975511, EPI_ISL_975512, EPI_ISL_975513, EPI_ISL_975514, EPI_ISL_975515, EPI_ISL_975516, EPI_ISL_975517, EPI_ISL_975518, EPI_ISL_975519, EPI_ISL_975520, EPI_ISL_975521, EPI_ISL_975522, EPI_ISL_975523, EPI_ISL_975524, EPI_ISL_975525, EPI_ISL_975526, EPI_ISL_975527, EPI_ISL_975528, EPI_ISL_975529, EPI_ISL_975530, EPI_ISL_975531, EPI_ISL_975532, EPI_ISL_975533, EPI_ISL_975534, EPI_ISL_975535, EPI_ISL_975536, EPI_ISL_975537, EPI_ISL_975538, EPI_ISL_975539, EPI_ISL_975540, EPI_ISL_975541, EPI_ISL_975542, EPI_ISL_975543, EPI_ISL_975544, EPI_ISL_975545, EPI_ISL_975546, EPI_ISL_975547, EPI_ISL_975548, EPI_ISL_975549, EPI_ISL_975550, EPI_ISL_975551, EPI_ISL_975552, EPI_ISL_975553, EPI_ISL_975554, EPI_ISL_975555, EPI_ISL_975556, EPI_ISL_975557, EPI_ISL_975558, EPI_ISL_975559, EPI_ISL_975560, EPI_ISL_975561, EPI_ISL_975562, EPI_ISL_975563, EPI_ISL_975564, EPI_ISL_975565, EPI_ISL_975566, EPI_ISL_975567, EPI_ISL_975568, EPI_ISL_975569, EPI_ISL_975570, EPI_ISL_975571, EPI_ISL_975572, EPI_ISL_975573, EPI_ISL_975574, EPI_ISL_975575, EPI_ISL_975576, EPI_ISL_975577, EPI_ISL_975578, EPI_ISL_975579, EPI_ISL_975580, EPI_ISL_975581, EPI_ISL_975582, EPI_ISL_975583, EPI_ISL_975584, EPI_ISL_975585, EPI_ISL_975586, EPI_ISL_975587, EPI_ISL_975588, EPI_ISL_975589, EPI_ISL_975590, EPI_ISL_975591, EPI_ISL_975592, EPI_ISL_975593, EPI_ISL_975594, EPI_ISL_975595, EPI_ISL_975596, EPI_ISL_975597, EPI_ISL_975598, EPI_ISL_975599, EPI_ISL_975600, EPI_ISL_975601, EPI_ISL_975602, EPI_ISL_975603, EPI_ISL_975604, EPI_ISL_975605, EPI_ISL_975606, EPI_ISL_975607, EPI_ISL_975608, EPI_ISL_975609, EPI_ISL_975610, EPI_ISL_975611, EPI_ISL_975612, EPI_ISL_975613, EPI_ISL_975614, EPI_ISL_975615, EPI_ISL_975616, EPI_ISL_975617, EPI_ISL_975618, EPI_ISL_975619, EPI_ISL_975620, EPI_ISL_975621, EPI_ISL_975622, EPI_ISL_975623, EPI_ISL_975624, EPI_ISL_975625, EPI_ISL_975626, EPI_ISL_975627, EPI_ISL_975628, EPI_ISL_975629, EPI_ISL_975630, EPI_ISL_975631, EPI_ISL_975632, EPI_ISL_975633, EPI_ISL_975634, EPI_ISL_975635, EPI_ISL_975636, EPI_ISL_975637, EPI_ISL_975638, EPI_ISL_975639, EPI_ISL_975640, EPI_ISL_975641, EPI_ISL_975642, EPI_ISL_975643, EPI_ISL_975644, EPI_ISL_975645, EPI_ISL_975646, EPI_ISL_975647, EPI_ISL_975648, EPI_ISL_975649, EPI_ISL_975650, EPI_ISL_975651, EPI_ISL_975652, EPI_ISL_975653, EPI_ISL_975654, EPI_ISL_975655, EPI_ISL_975656, EPI_ISL_975657, EPI_ISL_975658, EPI_ISL_975659, EPI_ISL_975660			
see above	BCCDC Public Health Laboratory	BCCDC Public Health Laboratory	Prystajecky Natalie, Linda Hoang, Dan Fornika, John Tyson, Shannon Russell, Kim Macdonald, Kimia Kamelian, Ana Pacagnella, Corrinne Ng, Loretta Janz, Robert Azana Terry Snutch, Mel Krajden
EPI_ISL_981176	Johns Hopkins Hospital Department of Pathology	Johns Hopkins Hospital Department of Pathology	C. Paul Morris, Chun Huai Luo, Adannaya Amadi, Matthew Schwartz, Nicholas Gallagher, Heba H. Mostafa
EPI_ISL_982771, EPI_ISL_982772, EPI_ISL_982773, EPI_ISL_982774, EPI_ISL_982775	Kentucky State Public Health Lab	Kentucky State Public Health Lab	Stephanie Lunn, Karim George, Joshua Tobias, William Grooms, Vaneet Arora, Matthew Johnson, Rachel Zinner, Rhonda Lucas
EPI_ISL_983699, EPI_ISL_983700	Essentia Health-St. Mary's Medical Center	Minnesota Department of Health, Public Health Laboratory	Alexandra Lorentz, Jacob Garfin, Matt Plumb, and Xiong Wang
